# The Monogenean Which Lost Its Clamps

**DOI:** 10.1371/journal.pone.0079155

**Published:** 2013-11-22

**Authors:** Jean-Lou Justine, Chahrazed Rahmouni, Delphine Gey, Charlotte Schoelinck, Eric P. Hoberg

**Affiliations:** 1 UMR 7138 “Systématique, Adaptation, Évolution”, Muséum National d'Histoire Naturelle, CP 51, Paris, France; 2 UMS 2700 Service de Systématique moléculaire, Muséum National d'Histoire Naturelle, Paris, France; 3 Molecular Biology, Aquatic Animal Health, Fisheries and Oceans Canada, Moncton, Canada; 4 United States National Parasite Collection, United States Department of Agriculture, Agricultural Research Service, Beltsville, Maryland, United States of America; Institut national de la santé et de la recherche médicale - Institut Cochin, France

## Abstract

Ectoparasites face a daily challenge: to remain attached to their hosts. Polyopisthocotylean monogeneans usually attach to the surface of fish gills using highly specialized structures, the sclerotized clamps. In the original description of the protomicrocotylid species *Lethacotyle fijiensis*, described 60 years ago, the clamps were considered to be absent but few specimens were available and this observation was later questioned. In addition, genera within the family Protomicrocotylidae have either clamps of the “gastrocotylid” or the “microcotylid” types; this puzzled systematists because these clamp types are characteristic of distinct, major groups. Discovery of another, new, species of the genus *Lethacotyle*, has allowed us to explore the nature of the attachment structures in protomicrocotylids. *Lethacotyle vera* n. sp. is described from the gills of the carangid *Caranx papuensis* off New Caledonia. It is distinguished from *Lethacotyle fijiensis*, the only other species of the genus, by the length of the male copulatory spines. Sequences of 28S rDNA were used to build a tree, in which *Lethacotyle vera* grouped with other protomicrocotylids. The identity of the host fish was confirmed with COI barcodes. We observed that protomicrocotylids have specialized structures associated with their attachment organ, such as lateral flaps and transverse striations, which are not known in other monogeneans. We thus hypothesized that the clamps in protomicrocotylids were sequentially lost during evolution, coinciding with the development of other attachment structures. To test the hypothesis, we calculated the surfaces of clamps and body in 120 species of gastrocotylinean monogeneans, based on published descriptions. The ratio of clamp surface: body surface was the lowest in protomicrocotylids. We conclude that clamps in protomicrocotylids are vestigial organs, and that occurrence of “gastrocotylid” and simpler “microcotylid” clamps within the same family are steps in an evolutionary sequence, leading to the absence of these attributes in species of *Lethacotyle*.

## Introduction

Monogeneans are Platyhelminthes, mostly ectoparasites on fish. Although the monophyly of the Monogenea is dubious [1], [2], there is no doubt that each of the two components of the monogeneans, namely the Polyopisthocotylea and the Monopisthocotylea, are each monophyletic and members of the Neodermata, the parasitic and terminal group of Platyhelminthes, together with the Cestoda and the Trematoda [3]–[5]. Members of both monogenean groups deal with a major issue of parasitic life, attachment to the host, by a posterior organ named the haptor (or opisthaptor) which possesses specialized attachment structures [6]–[8].

In the Polyopisthocotylea (the name means “many sucker-cups at the rear” [9]) the posterior haptor includes suckers or clamps [6], and the latter are considered one of the major morphological synapomorphies of the group [10]. These clamps, ranging in number from a few to hundreds, are highly specialized structures, often armed with sclerotised elements [6], [11]–[13]. Clamps attach to the host's surface (generally the gill of a marine fish) and thus allow the worm to resist the flow of water running through the gill chamber and to maintain position on its host [14]. The anterior body of the monogenean is deformable and allows it to feed from blood sucked from the gill [9].

Although all known polyopisthocotyleans have suckers or clamps, a single exception is represented by the species *Lethacotyle fijiensis* Manter & Prince, 1953 [15]. This worm is a parasite on the gills of an unnamed carangid fish off Fiji, a South Pacific island. The species was described, however, from only two specimens (among which only one is still in a museum collection) and the authors mentioned that there was a possibility that the clamps could have been lost - this is not an unusual phenomenon when specimens are not collected in optimal conditions. Hargis (1957) [16] also expressed doubt over the accuracy of the original description and considered that the complete absence of clamps was “unique and puzzling.” Later Ramalingam (1966, 1968) [17], [18] found other specimens of *Lethacotyle* Manter & Prince, 1953 on a carangid off the Andaman Islands, and confirmed the absence of clamps in adult and juvenile worms. However, Ramalingam's papers [17], [18] were largely ignored, i.e. by Llewellyn (1971) [19] who commented that “such extraordinary occurrences deserve re-examination.”

**Figure 1:**
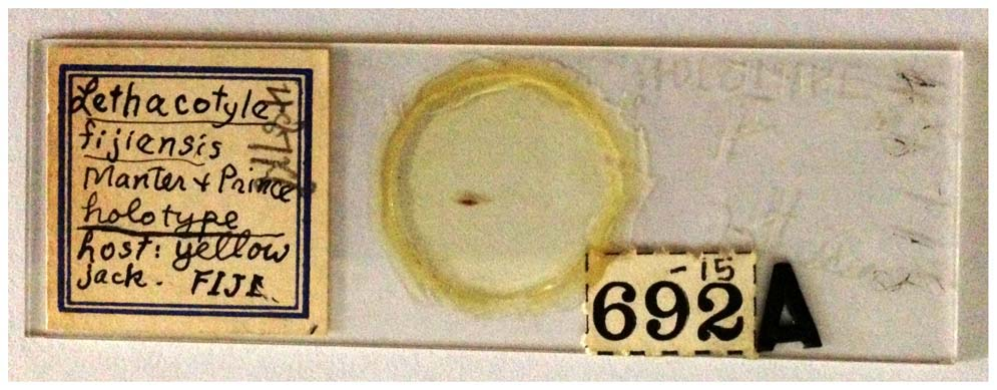
The single specimen of *Lethacotyle* available for study before this paper. The slide containing the single specimen of *Lethacotyle* available for study before this paper: holotype of *Lethacotyle fijiensi*s Manter & Prince, 1953 (urn:lsid:zoobank.org:act:DA367684-AAC2-44D0-A8E8-64894AFA647A), slide USNPC 48718. Our study is another example of the importance of Museum collections for modern research [86], [87].

Thus, in all, our current knowledge of *Lethacotyle*, in spite of its uniqueness and interest, is based on the observation of four specimens, three adults and one juvenile, in which only one has been kept in a museum and is available to study (Figure 1). No work has been published on *Lethacotyle* during the past 40 years and the doubts concerning the absence of clamps, expressed in the original description [15] and subsequent comments [16], [19], have remained problematic.

We collected off New Caledonia, another South Pacific island, a series of specimens of a previously unrecognized species of *Lethacotyle*. Specimens were collected in perfect condition for morphological study and were submitted to modern molecular analysis; the new species is described herein.

Further, during our current studies of *Lethacotyle* including comparisons among related monogeneans, we noted that clamps in species of protomicrocotylids were relatively small in comparison to the body. Thus, although individual clamps were not especially small, all clamps together occupied a small surface area of the body in comparison to other species of polyopisthocotylean monogeneans. Our observations suggest that clamps are reduced, or vestigial, in this family, an assertion based on the ubiquitous distribution of these attributes among basal polyopisthocotyleans and the putative phylogenetic relationships for the Protomicrocotylidae Johnston & Tiegs, 1922 [4], [6]. To test this hypothesis, we explored the phylogenetic placement of the Protomicrocotylidae and we compared the ratio for surface of clamps: surface of body in 120 monogenean species belonging to the Gastrocotylinea Lebedev, 1972. We found that the protomicrocotylids had the lowest ratio. Finally, we discuss the evolutionary significance of the absence of clamps in *Lethacotyle* spp., a unique feature among polyopisthocotylean monogeneans.

## Materials and Methods

### Hosts

Five specimens of *Caranx papuensis* Alleyne & MacLeay, 1877 were obtained in Nouméa City, New Caledonia, from amateur fishermen fishing from the piers of the harbour, or were bought at the fishmarket, from commercial fishmongers. The latter host specimens came from professional fisherman who specialize on mackerels, fish close to Nouméa City and bring back their catch within hours from the nearby fishing-grounds. Fish specimens are detailed in Table 1 with registration number, date, locality, length, weight and availability of photographs. Accurate identification of marine fish is often a problem in the South Pacific [20]–[24], and photographs of the fish were used to determine species identity by several ichthyologists. In addition, fish tissues were collected, stored in 95% or 100% ethanol, and processed for molecular identification. Specimens of *Caranx sexfasciatus* Quoy & Gaimard, 1825 from the same locality were examined and provided specimens of the monogenean *Neomicrocotyle* sp. used for comparison of morphology and molecules.

**Table 1:** Specimens of *Caranx papuensis* examined, specimens of *Lethacotyle vera* n. sp., and results.

JNC	Date	Locality	Fork Length (mm)	Weight (g)	Photo	Fish Sequences	Parasites	Parasite Sequences
JNC1185	05-07-2004	Nouméa harbour	372	843	Yes	-	3 specimens on slides; 2 adults, 1 juvenile	-
JNC1189	06-07-2004	Nouméa harbour	413	1,250	No	-	4 adult specimens on slides	-
JNC1988	04-10-2006	Nouméa fish market	275	350	Yes	COI: KF378585	1 adult specimen on slide	-
JNC3188	17-06-2010	Nouméa fish market	345	749	Yes	COI: KF378583	3 specimens: 1 juvenile on slide JNC3188A1; 1 adult, cut, anterior on slide JNC3188A2c, sequenced; 1 adult JNC3188A3c, sequenced	Specimen JNC3188A2c: 28S: KF378588
JNC3209	16-07-2010	Nouméa fish market	>500	unknown	Yes	COI: KF378584	1 adult specimen on slide	-

### Parasites

Monogeneans were collected alive or recently dead, flattened in cold ethanol, and routinely processed, including staining with carmine and mounting on a microscopic slide in Canada balsam [25]. Drawings were made using an Olympus BH2 microscope equipped with a drawing tube and differential interference contrast (DIC) optics. Measurements were made from pencil drawings with the help of a custom-made transparent rule, previously calibrated with a stage micrometer. Drawings were scanned and redrawn on a computer with Adobe Illustrator. All measurements are given in micrometres unless otherwise indicated. In the text and Tables, “juvenile” designates specimens with incomplete development of genital organs, especially of characteristic sclerotised organs.

### Museum specimens

The following museum slides were examined: *Bilaterocotyle novaeguineae* Rohde, 1977, paratype, USNPC 74800 (1 slide) (current status: *Bilaterocotyloides novaeguineae* (Rohde, 1977) Lebedev, 1986); *Neomicrocotyle* sp. from *Caranx sexfasciatus* off New Caledonia, MNHN JNC3242; *Protomicrocotyle celebesensis* Yamaguti, 1953, MNHN HEL80, HEL81; *Protomicrocotyle mannarensis* Ramalingam, 1960, USNPC 74798, BMNH 1978.6.15.6; *Protomicrocotyle manteri* Bravo-Hollis, 1966, paratype, USNPC 75514; *Protomicrocotyle mirabilis* (MacCallum, 1918) Johnston & Tiegs, 1922, BMNH 2002.8.12.3-4, BMNH 2007.7.25.34, 2007.7.25.30-33 (2 slides); *Protomicrocotyle pacifica* Meserve, 1938, USNPC 100122 (3 slides) (current status: *Neomicrocotyle pacifica* (Meserve, 1938) Yamaguti, 1968 [26]); *Protomicrocotyle* sp., BMNH 1985.11.8.48-47, BMNH 1985.11.8.48-52 (2 slides). The following slides could not be shipped but photographs were taken by curators: *Protomicrocotyle celebesensis*, MPM 22909 (SY6739); *Neomicrocotyle carangis* Yamaguti, 1968, holotype, USNPC 63672, and MPM 15660 (B2421-2423); *Lethacotyle fijiensis*, holotype, USNPC 48718 (Figure 1); *Protomicrocotyle pacifica*, holotype, USNPC 9166. Names in the above list are those from the original labels, sometimes updated with correct taxonomy and current usage. Patricia Pilitt (USNPC) and Eileen Harris (BMNH) are thanked for arranging specimen loans.

### Nomenclatural acts

The electronic edition of this article conforms to the requirements of the amended International Code of Zoological Nomenclature, and hence the new names contained herein are available under that Code from the electronic edition of this article. This published work and the nomenclatural acts it contains have been registered in ZooBank, the online registration system for the ICZN. The ZooBank LSIDs (Life Science Identifiers) can be resolved and the associated information viewed through any standard web browser by appending the LSID to the prefix “http://zoobank.org/”. The LSID for this publication is: urn:lsid:zoobank.org:pub:596C3FF5-CD24-4733-95FD-CC060A7FF0EE. The electronic edition of this work was published in a journal with an ISSN, and has been archived and is available from the following digital repositories: PubMed Central, LOCKSS.

### Molecular sequences

Fish DNA was extracted from tissue samples of three specimens (Table 1) using NucleoSpin 96 tissue kit (Macherey-Nagel) following the manufacturer's instructions. The 5′ region of the cytochrome oxidase I (COI) mitochondrial gene was amplified using the primers FishF1 (5′-TCAACCAACCACAAAGACATTGGCAC-3′) and FishR1 (5′-TAGACTTCTGGGTGGCCAAAGAATCA-3′) [27]. Species identification was confirmed using the BOLD identification engine [28].

One monogenean was cut in two parts: the anterior part, including the key sclerotised reproductive organs, was mounted, using routine methods, on a microscopic slide [25] as for whole worms, and the posterior part was used for DNA extraction. Thanks to this method, perfect traceability was insured between morphological and molecular methods (i.e. both were performed on the same monogenean individual); in addition, for host-parasite traceability, the individual host fish of the same individual monogenean was used for sequencing (Table 1). DNA was also extracted from another, whole individual monogenean and provided the same sequence.

For monogeneans, as little tissue was available, DNA was extracted using NucleoSpin 96 tissue kit with a modified protocol: the NucleoSpin 96 Tissue Binding Plate was replaced by the Plasmid Binding Plate (Macherey-Nagel) and elution was performed in 60 µL. A 28S rDNA fragment of 700 bp was amplified using the universals primers C1′ (5′-ACCCGCTGAATTTAAGCAT-3′) and D2 (3′-TCCGTGTTTCAAGACGG-5′) [29]. PCR reactions were performed in final volume of 20 µl, containing: 1 ng of DNA, 1× CoralLoad PCR buffer, 3 mM MgCl2, 66 µM of each dNTP, 0.15 µM of each primer, and 0.5 units of Taq DNA polymerase (Qiagen). Thermocycles consisted in an initial denaturation step at 94°C for 4′, followed by 38 cycles of denaturation at 94°C for 30″, annealing at 60°C, for 30″, and extension at 72°C for 1′. The final extension was conducted at 72°C for 7′. PCR products were visualized on a 1.5% agarose gel, purified and directly sequenced in both directions on 3730xl DNA Analyzer 96-capillary sequencers (Applied Biosystems) at Genoscope (Évry, France). Sequences were edited and assembled using CodonCode Aligner software (CodonCode Corporation, Dedham, MA, USA). Sequences were deposited in GenBank under the accession numbers KF378583–KF378585 (fish) and KF378588–KF378589 (monogeneans).

### Phylogenetic analysis of polyopisthocotylean monogeneans

The data matrix was built from the published alignment of Olson & Littlewood [30] (available from http://ebi.edu.au/ftp/databases/embl/align/ALIGN_000150.dat), restricted to the Polyopisthocotylea excluding Polystomatidae and Sphyranuridae (this corresponds to the group designated as Oligonchoinea in [30]; for equivalences of monogenean terminology, see Table 1 in [4]) to which were added two newly obtained sequences of 28S: one from *Lethacotyle vera* n. sp. (KF378588), and one from an unidentified species of *Neomicrocotyle* Ramalingam, 1960 (KF378589) from *Caranx sexfasciatus*. There is general agreement that the Oligonchoinea are monophyletic and that the Hexabothriidae are basal among them [10], [30]–[32], so the two hexabothriids of the original alignment were chosen as an outgroup.

Phylogenetic reconstruction was computed using the GTR+I+Γ model, selected as the best-fitting model of nucleotide evolution for 28S marker with ModelTest [33], in conjunction with PAUP 4.0b10 [34], following the AIC criterion. Trees were inferred using two probabilistic approaches: maximum likelihood with a non-parametric bootstrap (BP) using RaxML [35] and Bayesian Inference (BI) using MrBayes version 3.1.2 [36]. Maximum likelihood (ML) analyses were carried out online on the CIPRES Science Gateway (The CIPRES Portals. URL: http://www.phylo.org/sub_sections/portal) with RAxML-HPC BlackBox (7.2.7) [35]. BI analyses were performed using 1,000,000 generations with sampling every 100 generations and four Metropolis-coupled Markov chains Monte Carlo (MCMCMC) and other parameters by default. Two independent analyses were conducted to check for convergence of the results. The parameter estimates and convergence were checked using Tracer version 1.4 [37]. The first 25% of sampled trees were discarded prior to constructing a 50% majority rule consensus tree. Posterior probabilities (PP - Bayesian analysis) and Bootstrap values (BP - Maximum likelihood analysis) were used as indicators of node credibility and we used PP≥0.95 and BP≥75% as significant values.

### Analysis of relative importance of clamps in gastrocotylinean monogeneans

We compared the structure and the taxonomic distribution of clamps across the major group, the Gastrocotylinea Lebedev, 1972 [38], which contains the protomicrocotylids [39]. This is one of the largest groups of polyopisthocotylean monogeneans, which is characterized by a common, complex clamp structure known as “gastrocotylid” [19], [40] (but see below for changes of this structure in some protomicrocotylids).

Figures in the global literature were extracted from published PDF files or scanned from printed papers with a table top scanner with a 600 dpi resolution. The outlines of the body and of individual clamps were drawn with Adobe Illustrator and then filled in black. Drawings were exported in JPG format and area measurements (whole body including clamps and total of all clamps) were taken with ImageJ [41] on digital files. We checked against WoRMS [42] (date: 14 May 2013), the list of species of Gastrocotylinea for which we could obtain illustrations of sufficient quality. Our database includes 120 species; 9 of these species were not in WoRMS; the remaining 111 species represented 78% of the 142 species included in WoRMS. The 120 figures are available in a Supplementary File. The statistical significance of differences between families was tested with Mann & Whitney U test.

### Ethics statement

Fish used for collection of parasites were dead at the time we acquired them for study, having been commercially caught, and available for purchase at the Nouméa fish market; no permits were required for the described study, which complied with all relevant regulations.

### Abbreviations

Parasitological collections: BMNH, NHMUK: British Museum (Natural History), London, UK; MNHN, Muséum National d'Histoire Naturelle, Paris, France; MPM, Meguro Parasitological Museum, Tokyo, Japan; USNPC, United States National Parasite Collection, Beltsville, USA.

## Results

### Morphology of available specimens

**Figure 2:**
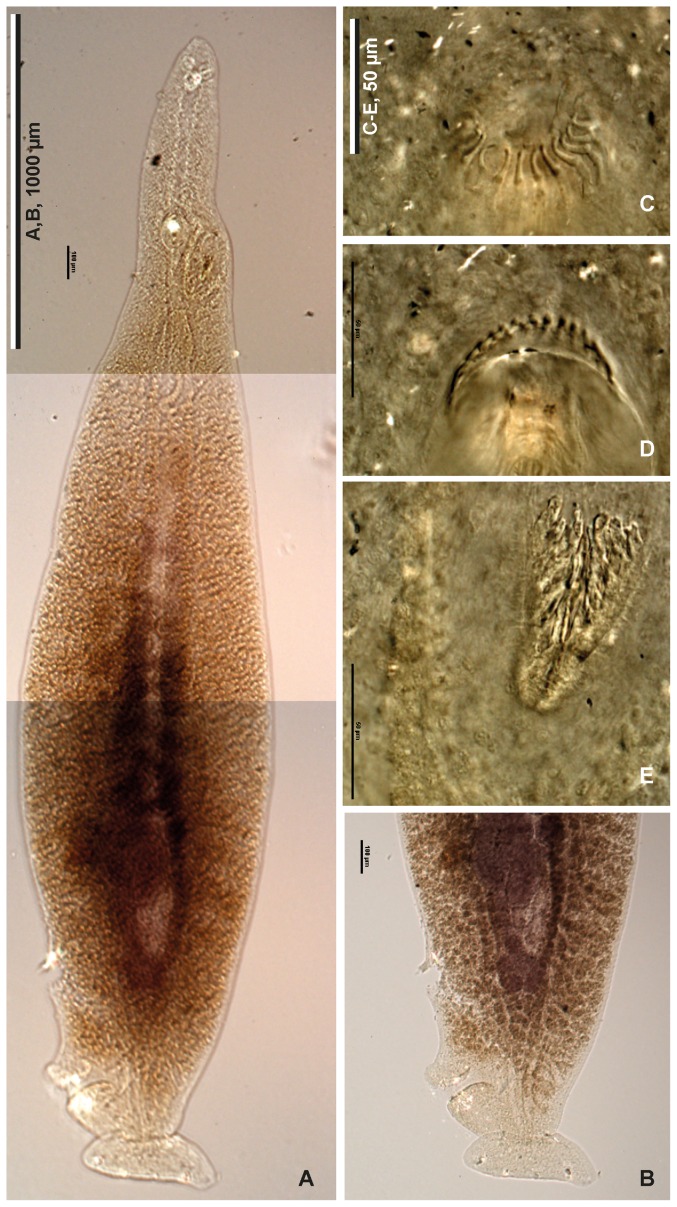
Photograph of the holotype of *Lethacotyle fijiensis* Manter & Prince, 1953. *Lethacotyle fijiensis* Manter & Prince, 1953 (urn:lsid:zoobank.org:act:DA367684-AAC2-44D0-A8E8-64894AFA647A). Holotype, slide USNPC 48718. A, body. B, posterior part of body, different focus. C, D, spines of male copulatory organ, two different focuses. E, sclerotised vagina. Original photographs taken by Patricia Pilitt, USNPC.

Museum specimens (or sometimes photographs of specimens) of species of protomicrocotylids, belonging to the genera *Lethacotyle*, *Protomicrocotyle*, *Neomicrocotyle*, and *Bilaterocotyle* were examined for the presence of clamps and other structures on the haptor. The number of clamps was found to be consistent with the published descriptions of species; particularly, we found no specimen with an incomplete number of clamps (i.e. only 5 clamps when 6 were described for the species). The single specimen of *Lethacotyle fijiensis* has no clamp (Figure 2), as emphasized in its original description [15]; the same is true for all specimens of our new species (formally described below).

**Figure 3:**
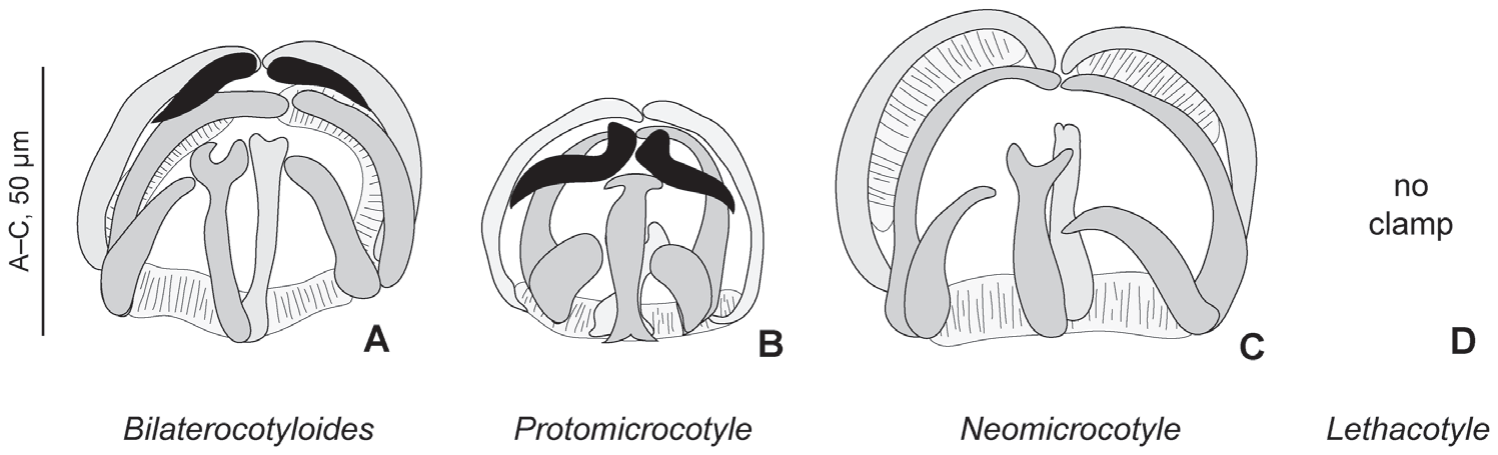
Clamps in various genera of Protomicrocotylidae. Examples of clamps in various genera of Protomicrocotylidae. A, *Bilaterocotyloides novaeguineae* (Rohde, 1977) Lebedev, 1986 (USNPC 74800). B, *Protomicrocotyle* sp. (MNHN JNC1163A5). C, *Neomicrocotyle* sp. (MNHN JNC3242A4). Black: additional sclerite, characteristic of the “gastrocotylid” clamp. *Bilaterocotyloides* and *Protomicrocotyle* have clamps of the “gastrocotylid” type, *Neomicrocotyle* has clamps of the “microcotylid” type, and *Lethacotyle* has no clamp.

Two types of clamps were found in specimens of protomicrocotylids (Figure 3), i.e. “gastrocotylid” type (with additional sclerite) and “microcotylid” type (without the sclerite).

In addition, we looked for striations on the haptor in specimens, or sought for the mention of striations in the descriptions. Table 2 shows that haptoral striations were often, but not always, mentioned in the descriptions of protomicrocotylids. Such striations are apparently not recorded (or observed) in other members of the Gastrocotylinea (and in polyopisthocotylean monogeneans as well), with the possible exception of a pseudodiclidophorid [43].

**Table 2:** Striations and other structures mentioned in protomicrocotylids.

Species	Observation	Reference
Subfamily Protomicrocotylinae		
*Lethacotyle vera* n. sp.	Figure 2	This paper
*Lethacotyle fijiensis*	“Dorsal surface of haptor with fine transverse striations” p. 105	[15]
*Lethacotyle* sp. from Andaman I. (as *L. fijiensis*)	Description of flaps pp. 108–109 (see discussion of present article);	[18]
*Protomicrocotyle mirabilis*	«Les faces ventrales et dorsales du hapteur et de la languette postérieure possèdent des stries transversales» (p. 320);	[55]
*Protomicrocotyle mirabilis* (as *Acanthodiscus mirabile)*	“body towards posterior disc transversally striated and spiny along dorsal surface” (p. 93); Figure 49	[64]
*Protocotyle celebensis*	“The caudal lobe is distinctly striated transversely like the posterior end of the body proper, giving a serrate appearance in profile”; Fig. 45	[65]
*Bilaterocotyle chirocentrosus*	Transversal striations not described, but well visible on Figs. 14, 15	[66]
*Neomicrocotyle indicus*	“The posterior portion of the body and the dumb-bell shaped haptor show transverse striations which give a spiny appearance to the surface of the worm”; Fig. 1	[67]
*Bilaterocotyle lucknowensis*	Fig. 7.52 (left Fig. and Fig. G)	[68]
*Bilaterocotyle mamaevi*	“Lappet two discs, each lappet lamellated”. Fig. 7.53 (left Fig. and Fig. G)	[68]
Subfamily Vallisiopsiinae		
*Youngiopsis australis*	Fig. 42D	[39]
*Vallisiopsis contorta*	“La partie élargie rayée du corps” ; Fig. 1	[40]

### Relative importance of clamps in gastrocotylinean monogeneans

**Figure 4:**
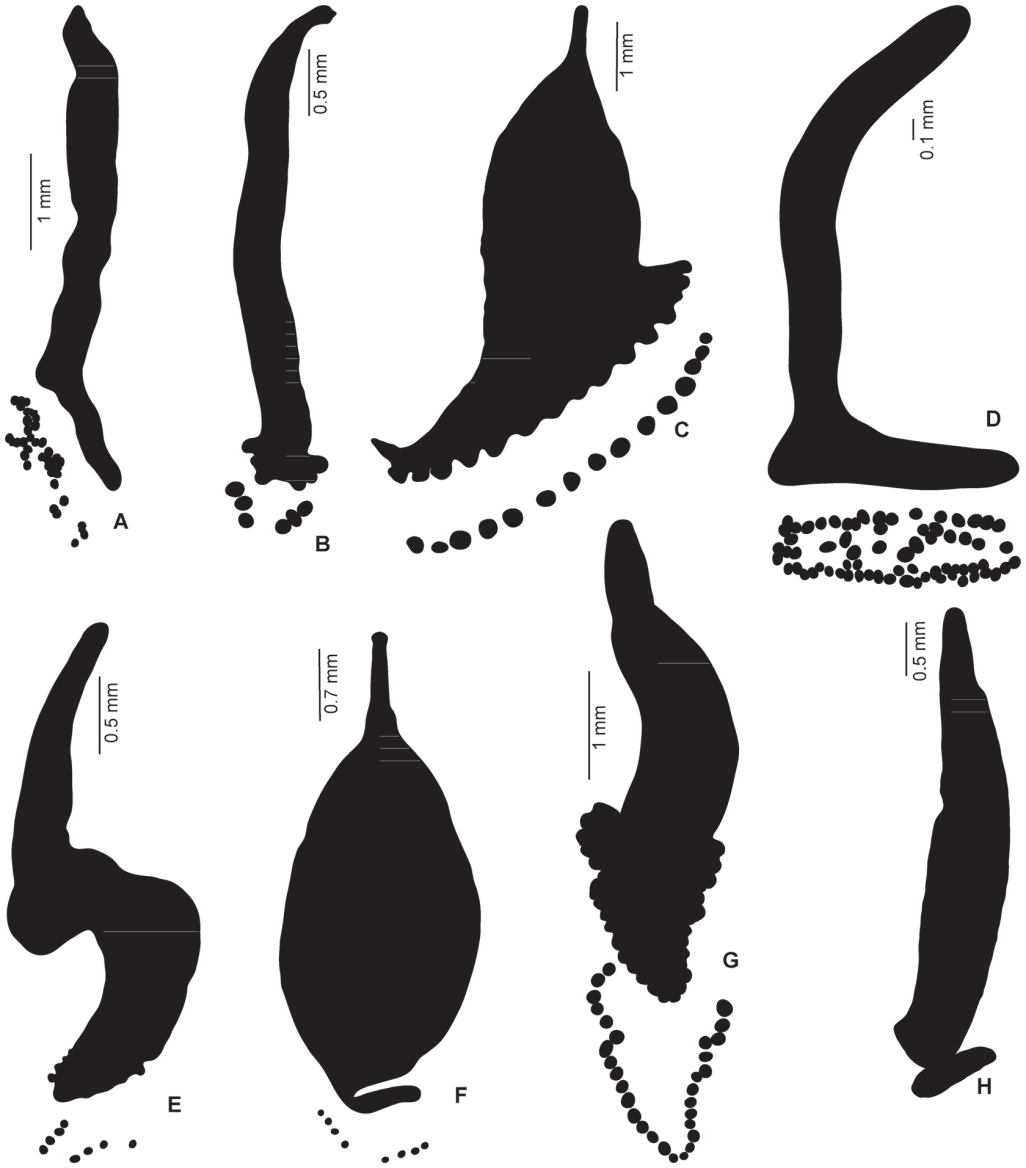
Body and clamp surfaces: examples of line drawings in 8 families. Body and clamp surfaces: examples of line drawings used for data extraction in each of the 8 families of the Gastrocotylinea. All species drawn to same body length. A, Gotocotylidae, *Gotocotyla niphonii*. B, Bychowskicotylidae, *Tonkinopsis transfretanus*. C, Gastrocotylidae, *Allopseudaxinoides euthynni*. D, Neothoracocotylidae, *Pricea minimae*. E, Allodiscocotylidae, *Metacamopia indica*. F, Pseudodiclidophoridae, *Allopseudodiclidophora opelu*. G, Chauhaneidae, *Cotyloatlantica mediterranea*. H, Protomicrocotylidae, *Lethacotyle vera* n. sp (no clamps). Details in Table 3.

**Figure 5:**
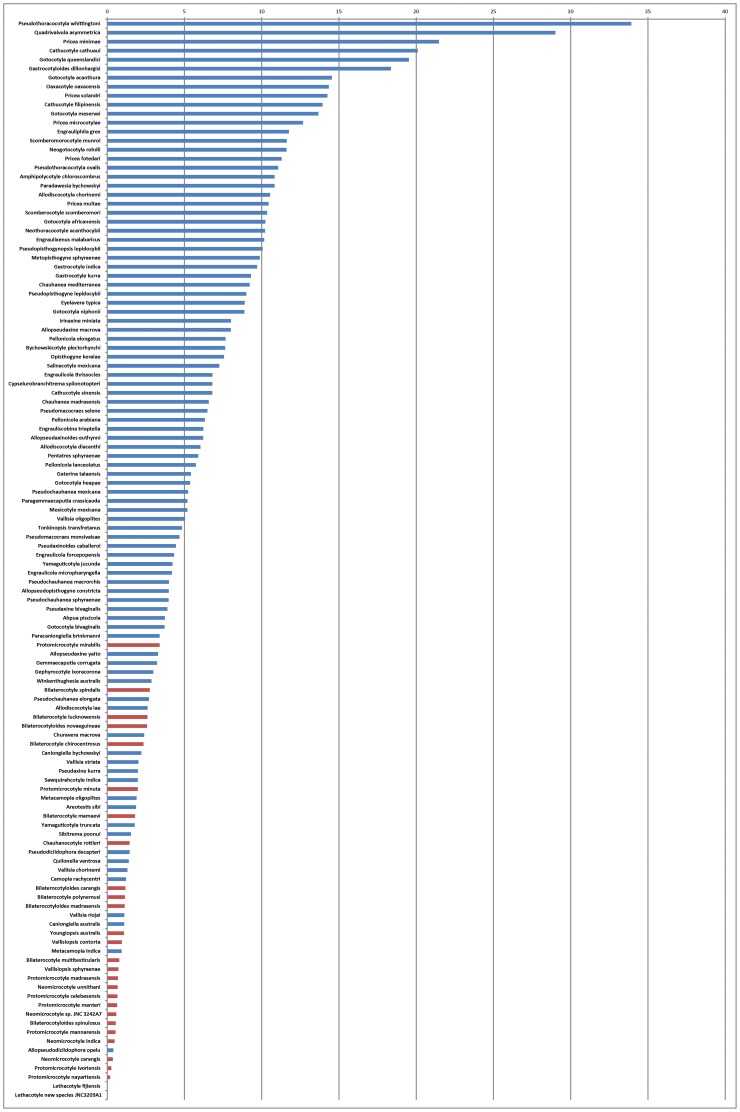
Ratio between clamp surface and body surface in species of gastrocotylinean monogeneans. Ratio between clamp surface and body surface in species of gastrocotylinean monogeneans. Ratios are ordered in decreasing sequence. Red: protomicrocotylids; blue: species of other families.

**Figure 6:**
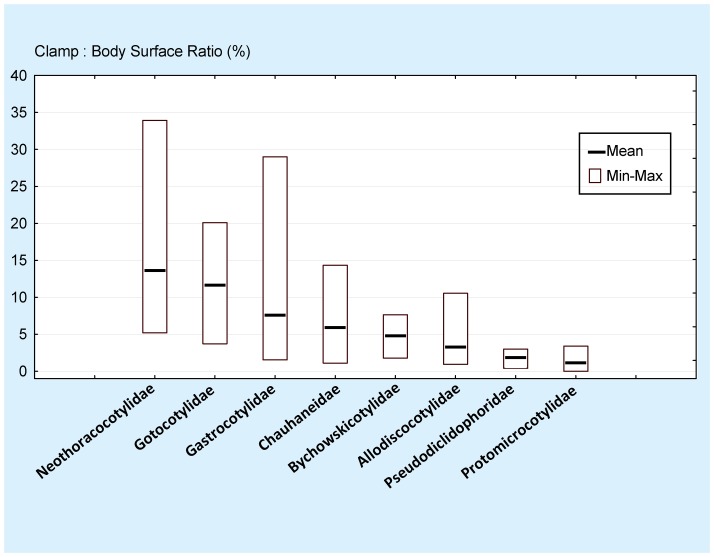
Ratio between clamp surface and body surface in families of gastrocotylinean monogeneans. Ratio between clamp surface and body surface in families of gastrocotylinean monogeneans. Ratios are ordered in decreasing order of mean. Protomicrocotylids have the lowest mean and lowest minimum. For significance see Table 4.

Examples of line drawings are shown in Figure 4; all 120 drawings are in the supplementary file. Data are in Table 3. Results of the comparison are presented in Figure 5 (data shown for all 120 species) and Figure 6 (data grouped by families). Among the 25 species with the smallest clamp: body ratios, 21 (84%) are protomicrocotylids (Figure 5). The clamp: body ratio in protomicrocotylids is the smallest of all families (Figure 6); ratios are smaller in protomicrocotylids than in each of the other families, and the differences are significant, except for the pseudodiclidophorids (Table 4).

**Table 3:** Surface of clamps and body in species of gastrocotylinean monogeneans.

Species	Family	Body surface	Clamp surface	Ratio	Reference, page
		(µm^2^)	(µm^2^)	%	
*Allodiscocotyla chorinemi* Yamaguti, 1953	Allodiscocotylidae	221,079	23,301	10.54	[8] p. 547
*Allodiscocotyla diacanthi* Unnithan, 1962	Allodiscocotylidae	891,326	53,724	6.03	[8] p. 547
*Allodiscocotyla lae* Yamaguti, 1968	Allodiscocotylidae	525,572	13,728	2.61	[69] p. 251
*Camopia rachycentri* Lebedev, 1970	Allodiscocotylidae	12,492,318	150,559	1.21	[39] p. 152
*Hargicola oligoplites* (Hargis, 1957) Lebedev, 1970as *Vallisia oligoplites* Hargis, 1957	Allodiscocotylidae	4,944,569	248,805	5.03	[16] p. 7
*Metacamopia chorinemi* (Yamaguti, 1953) Lebedev, 1984as *Vallisia chorinemi* Yamaguti, 1953	Allodiscocotylidae	1,278,926	16,737	1.31	[65] p. 65
*Metacamopia indica* (Unnithan, 1962) Lebedev, 1972	Allodiscocotylidae	1,488,158	13,866	0.93	[39] p. 154
*Metacamopia oligoplites* Takemoto, Amato & Luque, 1996	Allodiscocotylidae	3,273,506	62,243	1.90	[70] p. 167
*Vallisia riojai* Caballero & Bravo-Hollis, 1963	Allodiscocotylidae	971,416	10,768	1.11	[71] p. 175
*Vallisia striata* Parona & Perugia, 1890	Allodiscocotylidae	18,448,597	373,475	2.02	[72] p. 19
*Bychowskicotyle plectorhynchi* Lebedev, 1969	Bychowskicotylidae	857,260	65,536	7.64	[39] p. 100
*Gaterina talaensis* Lebedev, 1969	Bychowskicotylidae	619,337	33,586	5.42	[39] p. 101
*Tonkinopsis transfretanus* Lebedev, 1972	Bychowskicotylidae	1,236,435	59,827	4.84	[39] p. 104
*Yamaguticotyla jucunda* (Lebedev, 1969) Lebedev, 1984	Bychowskicotylidae	1,189,653	50,151	4.22	[39] p. 103
*Yamaguticotyla truncata* (Goto, 1894)	Bychowskicotylidae	4,924,003	87,494	1.78	[39] p. 60
*Ahpua piscicola* Caballero & Bravo-Hollis, 1973	Chauhaneidae	10,832	404	3.73	[73] p. 39
*Allopseudopisthogyne constricta* Yamaguti, 1965	Chauhaneidae	4,293,278	170,812	3.98	[74] p. 75
*Caniongiella australis* (Young, 1968) Lebedev, 1976	Chauhaneidae	624,748	7,033	1.1	[39] p. 127
*Caniongiella bychowskyi* Lebedev, 1976	Chauhaneidae	1,204,269	26,531	2.20	[39] p. 126
*Chauhanea madrasensis* Ramalingam, 1953	Chauhaneidae	2,631,446	172,925	6.57	[39] p. 120
*Cotyloatlantica mediterranea* (Euzet & Trilles, 1960)as *Chauhanea mediterranea* Euzet & Trilles, 1960	Chauhaneidae	3,309,353	305,081	9.22	[40] p. 190
*Gemmaecaputia corrugata* Tripathi, 1959	Chauhaneidae	404,195	13,015	3.22	[8] p. 487
*Metopisthogyne sphyraenae* Yamaguti, 1966	Chauhaneidae	3,415,824	337,042	9.87	[74] p. 426
*Oaxacotyle oaxacensis* (Caballero & Bravo, 1964) Lebedev, 1984	Chauhaneidae	397,303	56,968	14.34	[39] p. 138
*Opisthogyne keralae* Unnithan, 1962	Chauhaneidae	262,328	19,843	7.56	[44] p. 318
*Paracaniongiella brinkmanni* (Unnithan, 1962) Lebedev, 1976	Chauhaneidae	236,647	8,027	3.39	[68] p. 359
*Paragemmaecaputia crassicauda* Ramalingam, 1960	Chauhaneidae	189,399	9,836	5.19	[68] p. 357
*Pentatres sphyraenae* Euzet & Razarihelisoa, 1959	Chauhaneidae	497,849	29,309	5.89	[39] p. 132
*Pseudochauhanea elongata* Kritsky, Bilqees & Leiby, 1972	Chauhaneidae	593,000	16,000	2.70	[39] p. 122
*Pseudochauhanea macrorchis* Lin, Liu & Zhang in Zhang, Yang & Liu, 2001	Chauhaneidae	1,037,805	41,361	3.99	[75] p. 261
*Pseudochauhanea mexicana* Lamothe, 1967	Chauhaneidae	2,354,237	123,241	5.23	[39] p. 120
*Pseudochauhanea sphyraenae* Yamaguti, 1965	Chauhaneidae	2,795,654	110,955	3.97	[69] p. 251
*Pseudomazocraes monsivaisae* Caballero & Bravo Hollis, 1955	Chauhaneidae	810,544,311	37,813,692	4.67	[76] p. 108
*Pseudomazocraes selene* Hargis, 1957	Chauhaneidae	771,077	49,995	6.48	[16] p. 7
*Pseudopisthogyne lepidocybii* Yamaguti, 1965	Chauhaneidae	2,106,993	189,587	9.00	[57] p. 75
*Pseudopisthogynopsis lepidocybii* Yamaguti, 1965	Chauhaneidae	8,160,745	821,312	10.06	[39] p. 117
*Salinacotyle mexicana* (Caballero & Bravo-Hollis, 1963) Lebedev, 1984	Chauhaneidae	1,425,544	103,400	7.25	[39] p. 138
*Allopseudaxine macrova* (Unnithan, 1957) Yamaguti, 1963	Gastrocotylidae	4,858,980	388,611	8.00	[8] p. 265
*Allopseudaxine yaito* Yamaguti, 1968	Gastrocotylidae	14,764,474	485,975	3.29	[69] p. 251
*Allopseudaxinoides euthynni* Yamaguti, 1965	Gastrocotylidae	11,587,179	720,066	6.21	[57] p. 84
*Amphipolycotyle chloroscombrus* Hargis, 1957	Gastrocotylidae	209,204	22,656	10.83	[16] p. 5
*Areotestis sibi* Yamaguti, 1965	Gastrocotylidae	33,887,590	629,933	1.86	[57] p. 79
*Churavera macrova* Unnithan, 1968	Gastrocotylidae	2,844,619	67,889	2.39	[68] p. 368
*Cypselurobranchitrema spilonotopteri* Yamaguti, 1966	Gastrocotylidae	204,576	13,906	6.80	[74] p. 432
*Engraulicola forcepopensis* George, 1960	Gastrocotylidae	303,387	13,117	4.32	[68] p. 366
*Engraulicola micropharyngella* Unnithan, 1967	Gastrocotylidae	293,283	12,295	4.19	[77] p. 212
*Engraulicola thrissocles* (Tripathi, 1959) Lebedev, 1971	Gastrocotylidae	1,014,407	69,075	6.81	[39] p. 70
*Engrauliphila grex* Unnithan, 1967	Gastrocotylidae	208,190	24,488	11.76	[77] p. 218
*Engrauliscobina triaptella* Unnithan, 1967	Gastrocotylidae	1,465,577	91,211	6.22	[77] p. 221
*Engraulixenus malabaricus* Unnithan, 1967	Gastrocotylidae	556,699	56,562	10.16	[77] p. 215
*Eyelavera typica* Unnithan, 1968	Gastrocotylidae	9,500,350	844,481	8.89	[39] p. 74
*Gastrocotyle indica* Subhapradha, 1951	Gastrocotylidae	281,048	27,274	9.70	[68] p. 361
*Gastrocotyle kurra* Unnithan, 1968	Gastrocotylidae	2,864,859	266,456	9.30	[68] p. 362
*Gastrocotyloides dillonhargisi* Lebedev, 1980	Gastrocotylidae	1,273,059	233,778	18.36	[39] p. 72
*Irinaxine miniata* Ghichenok, 1980	Gastrocotylidae	741,990	59,441	8.01	[39] p. 60
*Pellonicola arabiana* Khan & Karyakarte, 1977	Gastrocotylidae	1,059,163	66,909	6.32	[68] p. 367
*Pellonicola elongatus* Unnithan, 1967	Gastrocotylidae	353,696	27,079	7.66	[77] p. 225
*Pellonicola lanceolatus* Kritsky & Bilqees, 1973	Gastrocotylidae	1,785,588	102,492	5.74	[78] p. 198
*Pseudaxine bivaginalis* Dillon & Hargis, 1965	Gastrocotylidae	1,137,648	44,201	3.89	[79] p. 276
*Pseudaxine kurra* Unnithan, 1968	Gastrocotylidae	1,909,954	37,999	1.99	[75] p. 268
*Pseudaxinoides caballeroi* Lebedev, 1977	Gastrocotylidae	2,096,832	93,128	4.44	[39] p. 57
*Quadrivalvula asymmetrica* Ghichenok, 1980	Gastrocotylidae	3,430,344	994,784	29.00	[39] p. 77
*Sibitrema poonui* Yamaguti, 1966	Gastrocotylidae	15,402,407	235,741	1.53	[74] p. 430
*Cathucotyle cathuaui* Lebedev, 1968	Gotocotylidae	1,303,159	261,873	20.10	[80] p. 450
*Cathucotyle filipinensis* Hayward & Rohde, 1999	Gotocotylidae	2,971,409	413,969	13.93	[80] p. 453
*Cathucotyle sinensis* Hayward & Rohde, 1999	Gotocotylidae	22,961,031	1,562,400	6.80	[80] p. 455
*Gotocotyla acanthura* (Parona & Perugia, 1896) Meserve, 1938	Gotocotylidae	3,209,235	466,446	14.53	[80] p. 431
*Gotocotyla africanensis* Hayward & Rohde, 1999	Gotocotylidae	2,445,683	250,315	10.23	[80] p. 438
*Gotocotyla bivaginalis* (Ramalingam, 1961) Rohde, 1976	Gotocotylidae	7,414,296	274,996	3.71	[80] p. 440
*Gotocotyla elagatis* Meserve, 1938as *Gotocotyla meservei* Yamaguti, 1953	Gotocotylidae	1,314,267	179,701	13.67	[65] p. 56
*Gotocotyla heapae* Hayward & Rohde, 1999	Gotocotylidae	1,291,877	69,182	5.36	[80] p. 443
*Gotocotyla niphonii* Hayward & Rohde, 1999	Gotocotylidae	1,932,305	171,386	8.87	[80] p. 445
*Gotocotyla queenslandici* Hayward & Rohde, 1999	Gotocotylidae	1,321,566	258,123	19.53	[80] p. 447
*Neogotocotyla rohdii* Hadi & Bilqees, 2010	Gotocotylidae	2,991,506	347,296	11.61	[81] p. 22
*Mexicotyle mexicana* (Meserve, 1938) Lebedev, 1984	Neothoracocotylidae	2,566,693	133,237	5.19	[39] p. 90
*Neothoracocotyle acanthocybii* (Meserve, 1938) Hargis, 1956	Neothoracocotylidae	126,796	12,940	10.21	[39] p. 88
*Pricea fotedari* Gupta & Sharma, 1979	Neothoracocotylidae	2,219,395	250,628	11.29	[68] p. 383
*Pricea microcotylae* Chauhan, 1945	Neothoracocotylidae	31,523	3,995	12.67	[66] p. 148
*Pricea minimae* Chauhan, 1945	Neothoracocotylidae	796,183	170,955	21.47	[66] p. 146
*Pricea solandri* Gupta & Channa, 1977	Neothoracocotylidae	29,180	4,158	14.25	[68] p. 382
*Pseudothoracocotyla ovalis* (Tripathi, 1956) Yamaguti, 1963	Neothoracocotylidae	1,104,406	122,155	11.06	[82] p. 164
*Pseudothoracocotyla whittingtoni* Hayward & Rohde, 1999	Neothoracocotylidae	6,151,442	2,086,478	33.92	[82] p. 167
*Scomberocotyle scomberomori* (Koratha, 1955) Hargis, 1956	Neothoracocotylidae	2,837,224	293,771	10.35	[39] p. 89
*Thoracocotyle crocea* MacCallum, 1913as *Paradawesia bychowskyi* Bravo & Lamothe, 1976	Neothoracocotylidae	3,604,259	390,338	10.83	[39] p. 94
*Pricea multae* Chauhan, 1945	Neothoracocotylidae	3,561,945	371,996	10.44	[83] p. 173
*Scomberomorocotyle munroi* Rohde & Hayward, 1999	Neothoracocotylidae	640,210	74,389	11.62	[84] p. 5
*Chauhanocotyle rottleri* Khoche & Dad, 1975	Protomicrocotylidae	912,981	13,257	1.45	[68] p. 356
*Bilaterocotyle chirocentrosus* Chauhan, 1945	Protomicrocotylidae	670,131	15,726	2.35	[66] p. 138
*Bilaterocotyle lucknowensis* (Agrawal & Sharma, 1986) Pandey & Agrawal, 2008	Protomicrocotylidae	92,963	2,413	2.60	[68] p. 350
*Bilaterocotyle multitesticularis* Khan & Karyakarte, 1982	Protomicrocotylidae	1,180,417	9,192	0.78	[68] p. 349
*Bilaterocotyle polynemusi* Gupta & Krishna, 1980	Protomicrocotylidae	1,358,610	15,508	1.14	[68] p. 347
*Bilaterocotyle spindalis* Deo & Karyakarte, 1980	Protomicrocotylidae	1,659,994	45,651	2.75	[68] p. 348
*Bilaterocotyloides carangis* Ramalingam, 1961	Protomicrocotylidae	1,135,204	13,434	1.18	[39] p. 114
*Bilaterocotyloides madrasensis* Radha, 1966	Protomicrocotylidae	441,228	4,984	1.13	[39] p. 116
*Bilaterocotyle mamaevi* Agrawal, 1988	Protomicrocotylidae	27,004	485	1.80	[68] p. 352
*Bilaterocotyloides novaeguineae* (Rohde, 1977) Lebedev, 1986	Protomicrocotylidae	442,980	11,411	2.58	[39] p. 114
*Bilaterocotyloides spinulosus* Liu in Zhang, Yang & Liu, 2001	Protomicrocotylidae	2,197,940	11,976	0.54	[75] p. 247
*Lethacotyle fijiensis* Manter & Price, 1953	Protomicrocotylidae	2,788,607	0	0	[39] p. 117
*Lethacotyle vera* n. sp.	Protomicrocotylidae	2,562,639	0	0	This paper
*Neomicrocotyle carangis* Yamaguti, 1968	Protomicrocotylidae	4,287,184	15,571	0.36	[39] p. 110
*Neomicrocotyle indica* Ramalingam, 1960	Protomicrocotylidae	49,651	232	0.47	[67] p. 375
*Neomicrocotyle* sp. JNC 3242A7	Protomicrocotylidae	2,663,686	15,327	0.58	This paper
*Neomicrocotyle unnithani* Yamaguti, 1968	Protomicrocotylidae	2,019,641	13,440	0.67	[44] p. 344
*Protomicrocotyle celebesensis* Yamaguti, 1953	Protomicrocotylidae	1,791,383	11,869	0.66	[65] p. 56
*Protomicrocotyle ivoriensis* Wahl, 1972	Protomicrocotylidae	2,939,959	7,682	0.26	[55] p. 324
*Protomicrocotyle madrasensis* Ramalingam, 1960	Protomicrocotylidae	736,440	5,113	0.69	[67] p. 375
*Protomicrocotyle mannarensis* Ramalingam, 1960	Protomicrocotylidae	1,934,754	10,205	0.53	[67] p. 377
*Protomicrocotyle manteri* Bravo-Hollis, 1966	Protomicrocotylidae	1,608,092	10,245	0.64	[39] p. 106
*Protomicrocotyle minuta* Ramalingam, 1960	Protomicrocotylidae	334,808	6,589	1.97	[67] p. 377
*Protomicrocotyle mirabilis* (MacCallum, 1918) Johnston & Tiegs, 1922	Protomicrocotylidae	231,559	7,854	3.39	[55] p. 321
*Protomicrocotyle nayaritensis* Bravo-Hollis, 1979	Protomicrocotylidae	7,317,320	13,238	0.18	[85] p. 190
*Vallisiopsis contorta* Subhapradha, 1951	Protomicrocotylidae	29,848	281	0.94	[39] p. 17
*Vallisiopsis sphyraenae* Yamaguti, 1968	Protomicrocotylidae	6,746,717	48,813	0.72	[69] p. 251
*Youngiopsis australis* (Young, 1968) Lebedev, 1972	Protomicrocotylidae	1,561,104	16,863	1.08	[39] p. 117
*Allopseudodiclidophora opelu* Yamaguti, 1965	Pseudodiclidophoridae	5,244,324	20,471	0.39	[57] p. 73
*Gephyrocotyle ixoracorona* Unnithan, 1966	Pseudodiclidophoridae	482,140	14,380	2.98	[68] p. 340
*Pseudodiclidophora decapteri* Yamaguti, 1965	Pseudodiclidophoridae	1,163,641	16,908	1.45	[57] p. 70
*Quilonella ventrosa* Lebedev & Parukhin, 1970	Pseudodiclidophoridae	954,583	13,250	1.39	[39] p. 81
*Sawquirahcotyle indica* Lebedev, 1976	Pseudodiclidophoridae	2,316,402	45,956	1.98	[39] p. 85
*Winkenthughesia australis* Robinson, 1961	Pseudodiclidophoridae	8,558,914	244,842	2.86	[43] p. 261

The outlines of body and clamps were redrawn on computer from original publications or from our own drawings, and the surface was calculated using ImageJ. Names of species follow WoRMS [42]; if different, name used in publication also indicated. All computerized line drawings available as Supplementary Material. Data ordered in alphabetical order of families and species.

**Table 4:** Significant differences of clamp surface: body surface ratios in families of gastrocotylinean monogeneans.

Families	n	Minimum	Maximum	Mean	P value
	(Total = 118)	(%)	(%)	(%)	
Neothoracocotylidae	12	5.19	33.92	13.61	0.000483
Gotocotylidae	9	3.71	20.10	11.47	0.000483
Gastrocotylidae	26	1.53	29	7.60	0.001699
Chauhaneidae	22	1.13	14.34	5.89	0.007222
Bychowskicotylidae	5	1.78	7.64	4.78	0.001699
Allodiscocotylidae	10	0.93	10.54	3.27	0.004136
Pseudodiclidophoridae	6	0.39	2.98	1.84	0.209316
Protomicrocotylidae	28	0	3.39	1.12	-

Families are in decreasing order of ratio. P values correspond to Mann & Whitney U tests between each family and the Protomicrocotylidae; all families have a significantly greater ratio than the Protomicrocotylidae, except the Pseudodiclidophoridae.

### Description of the new species

#### *Lethacotyle vera* Justine, Rahmouni, Gey, Schoelinck & Hoberg n. sp

urn:lsid:zoobank.org:act:0B7ABE99-07AF-4088-97F3-1A154DBA614D

Type-host: *Caranx papuensis* Alleyne & MacLeay.

Molecular identification of hosts: The blast search processed on BOLD engine [28] for the fish specimens MNHN JNC1988, JNC3188, JNC3209 (Table 1), confirmed the species identification as *C. papuensis* based on comparisons to the 12 available specimens in the database.

Type-locality: Off Nouméa, New Caledonia.

Site: Gills.

Type-material: Holotype MNHH JNC3209A1, collected 16-07-2010, Nouméa fish market. Paratypes: MNHN, JNC1185, JNC1189, JNC1988, JNC3188 (whole specimens); NHMUK, 1 slide, 2013.10.8.1; USNPC, 1 slide, 107263. One paratype cut in two parts, anterior part on slide MNHN JNC3188A2c, posterior part used for sequencing.

Prevalence: 5/5 (100%); intensity 1–4 (Table 1).

Etymology: *vera*, Latin for true, meaning that *Lethacotyle*, a genus differentiated by absence of clamps, was based on true observations.

Comparative material examined. *Lethacotyle fijiensis* Manter & Prince, 1953, holotype, USNPC 48718; the holotype slide (Figure 1) could not be shipped but photographs were taken and are herein shown in Figure 2. Other protomicrocotylids: see Materials and Methods.

### Description (Figures 7–8)

**Figure 7:**
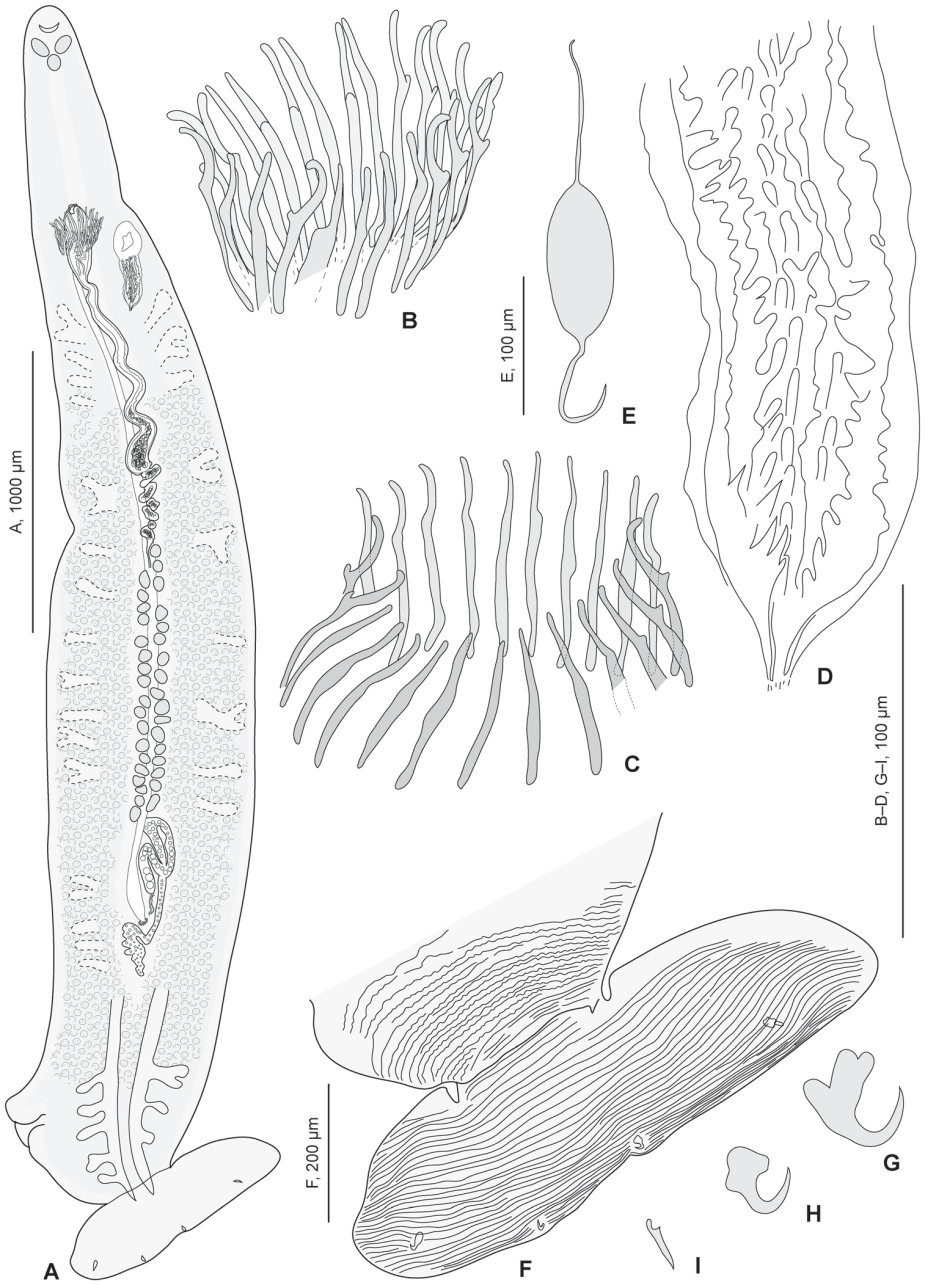
*Lethacotyle vera* n. sp. Adult and details. *Lethacotyle vera* n. sp (urn:lsid:zoobank.org:act:0B7ABE99-07AF-4088-97F3-1A154DBA614D). A, whole body; B, spines of male copulatory organ (MCO). C, spines of MCO in other specimen (paratype MNHN JNC1189A3). D, sclerotized vagina. E, egg, in utero. F, striations on posterior part of body; G, H, I, hooks (paratype MNHN JNC1185A3). A, B, D, F: holotype, MNHN JNC3209A1.

Body elongate, fusiform (Figure 7A). Tegument of body proper smooth; tegument of posterior part of haptor with parallel transverse striations.

Haptor devoid of clamps, slightly asymmetrical, comprising lateral pads and terminal lappet. Lateral pads two, anterior short, posterior long. Terminal lappet transversally elongate ovate, symmetrical, armed with 3 pairs of ventral sclerites (1 pair of hooks, 2 pairs of anchors). Lateral anchors located approximately at two thirds from centre of lappet; median anchors on posterior edge of lappet; hooks just external to corresponding median anchors. Lateral anchor with inner root partly divided medially (Figure 7G), outer root simple, strongly recurved point; median anchor with flattened triangular root and strongly recurved point (Figure 7H); hook with elongate, straight shaft (Figure 7I). Transverse striations on posterior part of haptor, including whole surface of terminal lappet and most terminal part of haptor, but not lateral flaps (Figure 7F). Pattern of striation similar on ventral and dorsal sides, 20–25 striations on terminal lappet, regularly parallel, 15–20 striations on body, less regular.

Mouth subterminal, ventral. Prohaptoral suckers ovoid, aseptate, lying diagonally in posterolateral wall of buccal cavity. Pharynx subovate, muscular, median and immediately posterior to prohaptoral suckers. Oesophagus long, devoid of diverticula, bifurcating to 2 intestinal caeca at level of genital atrium. Intestinal caecum in each lateral field of body proper, extending into haptor to anteriormost part of lappet; lateral intestinal diverticula numerous, branched, often indistinct; short diverticula in anterior haptor, no diverticula in lappet.

**Figure 8:**
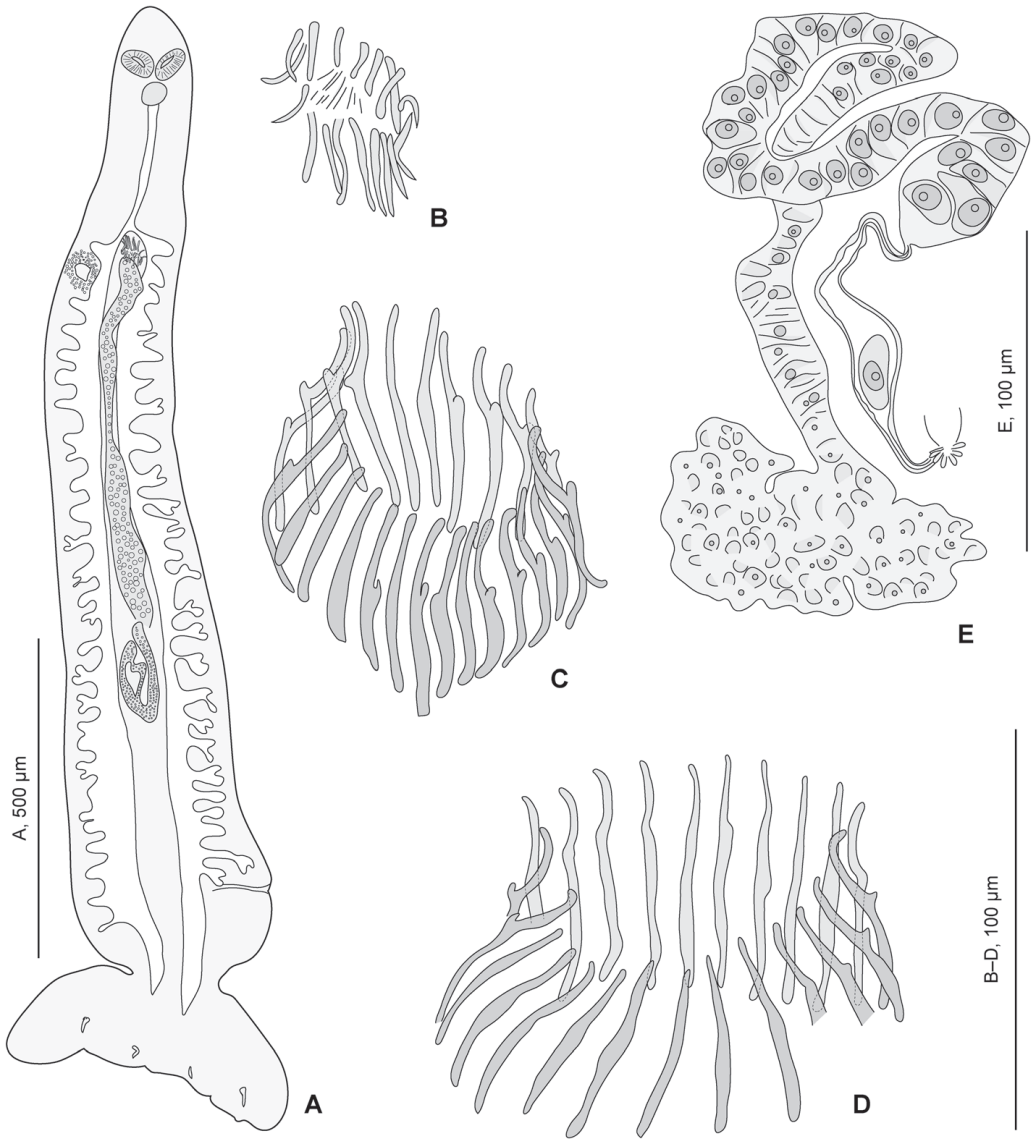
*Lethacotyle vera* n. sp. Juvenile and other details. *Lethacotyle vera* n. sp (urn:lsid:zoobank.org:act:0B7ABE99-07AF-4088-97F3-1A154DBA614D). A, juvenile (specimen MNHN JNC3188A1). B, spines of MCO in juvenile. C, spines of MCO in paratype MNHN JNC3188A2c (posterior part of body processed for molecular study); D, spines of MCO in paratype MNHN JNC1189A2. E, outline of ovary (paratype JNC1189A1).

Genital atrium unarmed, median. Testes ovoid, pregermarial, intercaecal, in 2 bilateral rows along body midline. Vas deferens expanding just anterior to anteriormost testis into seminal vesicle filled with sperm; seminal vesicle continued anteriorly by wide canal to male copulatory organ (MCO); vas efferentia and prostate not visible. MCO an elongate bulb, with muscular wall and internal coiled canal, armed with anterior spines; mass of bulb sometimes protruding anterior to spines. Spines arranged in a tight circle (“genital corona”), with tips directed outward and extending into genital atrium. Spines elongate, with blunt ends, elongate root, and thumb located at anterior third. General arrangement of spines of genital corona slightly variable with specimens (Figures 7B, 7C, 8C, 8D), but morphology of spines similar in all adult specimens.

Germarium intercaecal, with posterior immature mass, anteriorly directed branch, posteriorly directed looped mature branch (Figure 8E). Small coiled canal with visible wall from extremity of germarium to posterior part of ootype. Ootype elongate, median, with basal Mehlis' glands, continued anteriorly as uterus. Uterus linear, thin walled, extending up to genital atrium; superposed to seminal vesicle along part of its path. Median vitelline duct ventrally superposed to ootype (in holotype), anterior paired vitelline branches visible only on short distance.

Vaginal pore ventral, at midlength of MCO bulb level, on either side of body midline but opposite to that of haptoral pads. Vagina comprising anterior smooth part and posterior sclerotised part. Sclerotised part (Figure 7D) cone-shaped, with pointed extremity posterior; internal longitudinal crests with irregular spines; posterior end a small sclerotised conical canal. Smooth canal between sclerotised part and rest of female organs not seen.

Vitellarium in two lateral fields, never dense; anterior extremity at level of seminal vesicle; posterior extremity at level of haptoral anterior pad, i.e. anteriormost and posteriormost part of intestine not coextensive with vitellarium.

Egg elongate, with long anterior and posterior filaments (Figure 7E). *In utero*, egg length 220–225, width 82–95, filament length 412–467 (n = 2).

Juvenile specimens (Figures 8A, 8B). Two juvenile specimens briefly described for differential maturation of organs and sclerotised parts. One specimen (slide MNHN JNC3188A1, Figure 8A), 1300 in length, 320 in width: body almost symmetrical, haptoral pads barely visible; MCO spines incomplete, 22–24 in length, no thumbs on spines (Figure 8B); vagina a denser zone, no sclerotisation; germarium visible, testis zone an indistinct mass; haptoral hooks not well oriented. One specimen (slide MNHN JNC1185A3, not figured), 1700 in length, 350 in width, body symmetrical, MCO a dense mass without sclerotisation, all other genital organs indistinct; haptoral sclerotised parts well visible, morphology of lateral and median anchors similar to adult. Note that the longest juvenile specimen is apparently the less mature according to less differentiated sclerotised parts.

### Differential diagnosis

*Lethacotyle vera* n. sp. is similar to the single species described in the genus, *L. fijiensis*, based on the following characters (Table 5): body shape, and especially haptoral shape; total absence of clamps; body dimensions (mean 4340, 2300–5720) *vs* 3156–3759 in *L. fijiensis* [15]; presence of a circle of spines in MCO and shape of individual spines; number of spines 23 (17–27) *vs* 24–25 in *L. fijiensis*; shape of cone-like sclerotised vagina; shape and position of sclerotised haptoral parts.

**Table 5:** Measurements of *Lethacotyle* species.

*Lethacotyle*	*L. vera* n. sp.	*L. vera* n. sp.	*L. vera* n. sp.	*L. fijiensis*	*“L. fijiensis”*	*“L. fijiensis”*
	Holotype	Adults	Juveniles	Adult	Adult	Juvenile
Reference	This paper	This paper	This paper	Manter & Price, 1953 [15]	Ramalingam, 1968 [18]	Ramalingam, 1968 [18]
n	1	8	2	2	1	1
Body Length	5130	4340 (2300–5720, n = 8)	1300, 1700	3156–3759	1540	950
Body Width	750	973 (500–1270, n = 9)	320, 350	663–770	380	130
Pharynx Length	53	66 (53–75, n = 9)	38, 45	64	50	37
Pharynx Width	45	59 (45–70, n = 9)	38,43	50	33	25
Buccal Sucker Length	70	71 (50–83, n = 18)	60, 60	49–52 (diameter)	37	27
Buccal Sucker Width	42–57	59 (42–75, n = 18)	38, 50		25	25
Anterior-Genital Pore Distance	800	648 (360–803, n = 9)				
Number of Genital Corona Spines	24	24 (21–27, n = 9)		24–25	24	
Length of Genital Corona Spines	52 (43–66, n = 10)	50±5.7 (35–66, n = 138)		24	15	
Number of Testes	34	29 (21–34, n = 7)		30	31	
Testis Length	43±7.9 (25–55, n = 34)	52 (25–82, n = 57)				
Testis Width	42±7.9 (27–52, n = 34)	145 (63–262, n = 57)				
Testicular Mass Length	975	913 (588–1163, n = 6)			370	
Testicular Mass Width	125	352 (125–489, n = 6)				
Sclerotized Vagina Length	150	157 (125–175, n = 9)				
Sclerotized Vagina Width	85	81 (38–100, n = 9)				
Unsclerotised Vagina Length	175	170 (60–250, n = 9)				
Anterior-Vagina Pore Distance	850	739 (407–938, n = 9)				
Ovary Length	542	591 (114–935, n = 8)				
Ovary Width	192	340 (192–550, n = 8)				
Haptor Total Length	282	325 (245–400, n = 7)	207, 275		130	90
Haptor Total Width	850	764 (588–850, n = 6)	452, 525		260	280
Hamulus Length	24, 30	28 (24–33, n = 15)	27, 32	24	33	30
Posterior Hook Length	16, 16	18 (10–24, n = 13)	15, 16	16	18	22
Small Hook Length		14 (11–16, n = 8)		14	12	12

All measurements are in µm, in the form: mean (minimum–maximum), except for a few measurements with sample >30, for which measurements are in the form: mean ± standard deviation (minimum–maximum).

It differs in MCO spine length (mean 50±5.7, 35–66) *vs* 24 in *L. fijiensis* and shape of sclerotised vagina longitudinal crests, with irregular spines along length *vs* with minute terminal spines in *L. fijiensis*. The length of MCO spines in the holotype of *L. fijiensis* was ascertained by scaled photographs. Note that in specimens of “*L. fijiensis*” described by Ramalingam [17], [18] the length of the MCO spines was reported as 15 (*vs* 24 in original description [15]) and thus this might represent another species (see below); *L. vera* n. sp. is distinct from this putative species by the length of MCO spines.

### Phylogenetic position of the new species

**Figure 9:**
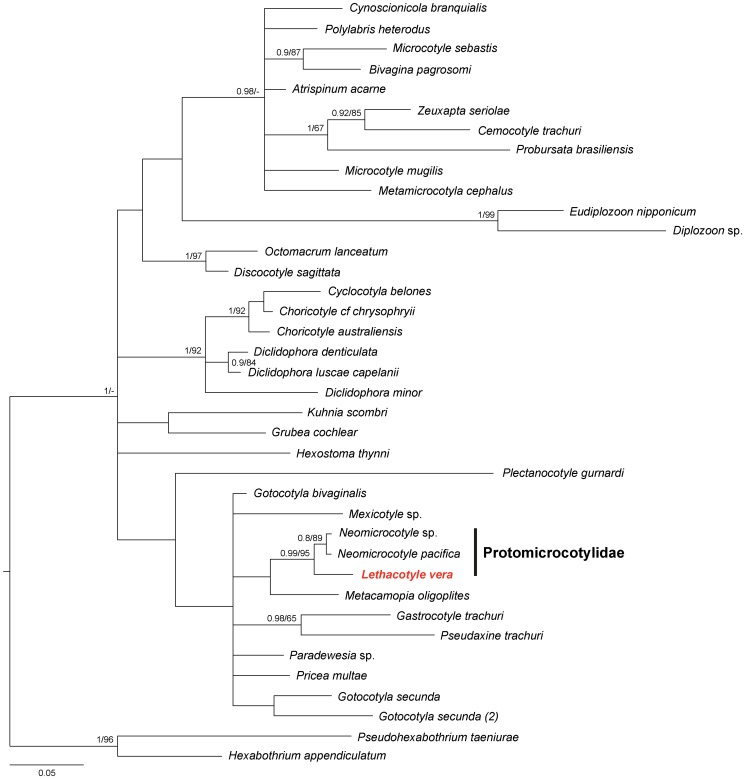
Tree of gastrocotylinean monogeneans. Tree of gastrocotylinean monogeneans, based on a phylogenetic analysis of 28S sequences.

A phylogenetic analysis of 28S sequences (Figure 9) show that the new species forms a clade (PP = 0.99, BP_ML_ = 95) with *Neomicrocotyle pacifica* (from *Caranx hippos* (Linnaeus, 1766) off Mexico [30]) and *Neomicrocotyle* sp. (our specimens from *Caranx sexfasciatus* off New Caledonia), the two other protomicrocotylids of the dataset.

## Discussion

### Taxonomic discussion of the new species

#### Classification of *Lethacotyle*

A diversity of taxonomic opinions illustrates the problematic nature and difficulty for classification of Lethacotyle and more generally for the Protomicrocotylidae. For example, Lethacotyle was classified within the family Discocotylidae Price, 1936, subfamily Vallisiinae Price, 1943 in the original description [15]; in Protomicrocotylidae Poche, 1926, Lethacotylinae Unnithan, 1962 by Unnithan (1962) [44] and in Protomicrocotylidae Johnson & Tiegs, 1922, Lethacotylinae Yamaguti, 1963, independently by Unnithan (1962) [44] and with a different definition of the subfamily, by Yamaguti (1963) [8]; in Gastrocotylidae Price, 1943, Valisiinae Price, 1943 by Hargis, 1957 [16]; and in Protomicrocotylidae (Johnston & Tiegs, 1922), Protomicrocotylinae Johnston & Tiegs, 1922 in the monograph by Lebedev (1986) [39]. The taxonomic confusion about the authority for the family Protomicrocotylidae in this list will not be commented upon here, but the challenge for classification clearly originates in the structure of the clamps (or their absence). The classification of polyopisthocotylean monogeneans is mainly based on clamp structure, but protomicrocotylids are unique in that this structure changes relative to each genus within the family: Protomicrocotyle has clamps of the gastrocotylid type, but Neomicrocotyle has clamps of the microcotylid type. In addition, the asymmetrical morphology of the haptor in protomicrocotylids has been considered as “extraordinary” [19]. Unfortunately, the genus Lethacotyle was not included in discussions of modern phylogenies of monogeneans [10], [45].

Our molecular phylogenetic analysis shows that *Lethacotyle vera* n. sp. groups with two species of *Neomicrocotyle* and thus confirms that the genus *Lethacotyle* belongs to the Protomicrocotylidae, in spite of the absence of clamps.

An hypothesis could be proposed, in which *Lethacotyle* would be a primitive species without clamps, with more derived species having clamps; our analyses clearly falsify this hypothesis, and demonstrate that the Protomicrocotylidae is not among the basal groups among the polyopisthocotyleans and the Gastrocotylinea.

### Species in *Lethacotyle*

Manter & Prince (1953) described *L. fijiensis* from two specimens from “yellow jack” [15]; the identification of the host fish is vague, as often with Manter's work (other cases: [46], [47]), and almost useless (many carangids are partly yellow). Only one monogenean specimen, the holotype of *L. fijiensis*, is kept in the USNPC collections (Figure 1).

Ramalingam [17], [18] described a species of *Lethacotyle* from “*Caranx sexfasciatus*” from off the Andaman Islands, and claimed it was the same species as *L. fijiensis*. No deposition of specimens in a curated collection or museum is mentioned in the papers. The MCO spines as described by Ramalingam are 15 µm in length. The host of the *Lethacotyle* species described by Ramalingam is “*C. sexfasciatus*” but the author mentioned that the carangids were 15 cm [18] and 5.2–26.5 cm [17] in length. Maturity of *C. sexfasciatus* is attained at 40 cm, common length is 60 cm, and maximum published weight is 18 kg [48]. Species identification of carangids, when they are adult, is often difficult, but the validity of species identification of the small specimens examined by Ramalingam is certainly dubious.

Therefore, we consider that: (a) the host of *L. fijiensis* in Fiji is an unknown carangid (due to insufficient host identification by Manter & Prince [15]); (b) the host of the *Lethacotyle* species described by Ramalingam is an unknown carangid, due to identification from immature fish specimens [17], [18], and we see no reason why it should be the same species as Manter & Prince's host fish. It might be *C. sexfasciatus*, as claimed by the author; however, we examined several *C. sexfasciatus* from off Australia and New Caledonia, and found no species of *Lethacotyle* [49]; (c) it is likely, based on collections from widely separated areas (Andaman Islands *vs* Fiji, which are separated by 9,000 km), the probability of different host species, and differences in measurements of the MCO spines (Table 5), that the species described by Ramalingam is distinct from both *L. fijiensis* and *L. vera* n. sp.; (d) and thus, *Lethacotyle* probably comprises, at least, three species.

Our species is the first referred to *Lethacotyle* with a precise host identification. We have examined a number of other carangids from several genera off New Caledonia [47], [49]–[53] and found *L. vera* n. sp. only on *C. papuensis*, suggesting that species of *Lethacotyle* are specific to *Caranx* species. It is likely that the “yellow jack” of Manter & Prince (1953) [15] and the carangid of Ramalingam [17], [18], both identified with suboptimal precision, were species of *Caranx*, but, as explained above, not necessarily conspecific.

### Clamps in protomicrocotylids vs other monogeneans

Our results (Figures 5, 6) show that the clamp surface is significantly smaller in species of the protomicrocotylids in comparison to other gastrocotylinean monogeneans. In addition, our description of *L. vera* n. sp. confirms that clamps are completely absent in members of the genus *Lethacotyle*. Clamps are an important and characteristic part of the anatomy of polyopisthocotylean monogeneans, and are clearly the main organ used for attachment to the host [6], [8], [11]–[13]. Protomicrocotylids, no less than other monogeneans, need to maintain attachment to their host. In a fluid environment maintenance of position on the external surfaces of the host represents a challenge, and one potentially heightened for protomicrocotylids that possess miniscule clamps, and for species of *Lethacotyle*, in which clamps are completely absent.

We hypothesize that other structures play a role in host attachment in protomicrocotylids, as habitat selected by these monogeneans (the fish gill) does not differ substantially from that characteristic of other gastrocotylineans which have fully developed clamps. Among protomicrocotylids, fixation may be attained by the combined action of the haptoral hooks, the lateral flaps of the haptor, and the striations on the posterior haptoral lappet. Hooks are relatively small in protomicrocotylids and are thus not considered of importance in attachment.

Ramalingam [18], apparently from a study of living specimens (although this is not clearly stated in his paper) described the flaps of the haptor and reported that “the gap between the flaps in the anteroposterior axis can be narrowed by the contraction of the body in this region as well as by the extensile power of the flaps thus bringing their free ends in contact with each other or may lead to overlapping condition”. He explained that the flaps “on coming into contact with the filaments may either press against them thus helping to hold on to them or after getting a hold around the filaments may adpress them against the body and thus effect a hold on to the gills”. He concluded “this mode of effecting attachment to the gills by means of outgrowths of body surface is unique in Monogenea. An adventious growth of the body surface as seen in this case is rather unique and possibly nothing parallel is known among the animal kingdom”.

Unfortunately, we cannot confirm Ramalingam's observations and hypotheses, having not observed living worms. Striations are visible on the posterior lappet of *L. vera* n. sp., and also on other protomicrocotylids (Table 5). Such transverse striations are rather unique among monogeneans. Some information about the precise habitat of protomicrocotylids are available; Rohde [54] stated that *Protomicrocotyle* sp. was only found on the posterior surface of the internal filaments of the first gill of *Caranx melampygus* Cuvier, 1833. Wahl described the position of specimens of *Protomicrocotyle ivoriensis* Wahl, 1972 and *P. mirabilis* according to their asymmetry and noted that the posterior lappet was intercalated between two gill lamellae ([55], p. 329). Indeed, transverse striations are probably efficient for attachment, by increasing friction, only when the posterior lappet is perpendicular to the longitudinal axis of the worm, and firmly applied against the gill surface.

It is apparent that development of a complex of organs associated with the haptor, and a reduction in the size and complexity of the clamps is associated with evolution of the Protomicrocotylidae. In this group, development of organs for the attachment on the host, including lateral flaps and posterior tegumental striations, or a combination of these two structures, apparently renders clamps of little significance for attachment. It is not clear which came first (reduction of the clamps or development of a complex of tegumental organs for attachment), and comprehensive phylogenetic analysis of all members of the family would be needed to resolve this question [56]. Given the overall phylogenetic placement of the family, and relative to other Gastrocotylinea, clamps must be considered vestigial organs in most protomicrocotylids (genera *Protomicrocotyle*, *Neomicrocotyle*, *Bilaterocotyle* and *Bilaterocotyloides*) and are absent in species of *Lethacotyle*. The existence of two major types of clamp structures (gastrocotylid type in *Protomicrocotyle*, microcotylid type in *Neomicrocotyle*) which puzzled systematists [8], [16], [19], is consistent with a secondary loss of the accessory sclerites in *Neomicrocotyle*, transforming the more complex gastrocotylid clamp into a simpler microcotylid-like clamp.

The Pseudodiclidophoridae also have a small clamp: body ratio, slightly higher than but not significantly different from the protomicrocotylids (Figure 6, Table 4). As our study concerns mainly the protomicrocotylids, we provide here only limited comments about pseudodiclidophorids. Only 5 pseudodiclidophorids were studied, and none has completely lost the clamps; one has transverse striations [43], and one, *Allopseudodiclidophora opelu* Yamaguti, 1965 (Figure 4F) has a “long anchor-bearing appendage” [57]; several have outstandingly wide posterior bodies that evoke the possibility of this part working as a sucker, as suggested for the microcotylid *Aspinatrium gallieni* Euzet & Ktari, 1971 [58]. These observations suggest that reduction in clamps, coincidental with development of secondary organs of attachment is a rare event, but has occurred in multiple lineages of phylogenetically disparate polyopisthocotyleans. Among some pseudodiclidophorids, evolution towards a reduced role of clamps has occurred without attaining the secondarily simplified microcotylid-like structure nor the total absence observed within the protomicrocotylids. Establishing phylogenetic context is a primary foundation necessary to differentiate between secondary loss (as proposed for these lineages of monogeneans) in contrast to plesiomorphic absence [56]. Additionally, the phylogenetic framework is critical for establishing the temporal association and sequence of evolutionary modification in complex attributes.

### Clamps of protomicrocotylids as vestigial organs

Vestigial organs are structures that have apparently lost their ancestral function in a species, and for which homologous and functional organs are known in related species. Typical examples are the loss or reduction of flight organs in some island-dwelling species (in insects or birds, independently), limbs bones in cetaceans, or the loss of eyes and pigmentation in cavern-dwelling species which have occurred under changing regimes for selection [59]–[62]. Parasites, in old anthropogenic interpretations, were considered “simpler” than free-living animals because they had lost certain organs (such as the intestine in cestodes)(e.g. [56]). More nuanced observations have demonstrated the considerable specialization and structural and biochemical complexity of helminths which often have complexes of novel organ systems in relation to parasitism, such as various sensory attributes in larvae, used to seek hosts [56], [63]. In *Lethacotyle* and protomicrocotylids, the loss and modification of organs concerns the haptor and clamps, body parts of the monogeneans which are clearly an adaptation to ectoparasitism. The occurrence of vestigial clamps or the complete absence of clamps, however, does not demonstrate that these parasites are “simplified”. In contrast, reduction has occurred in the evolutionary context for development of novel structures for attachment (flaps and striations) which are unique among any of the lineages of the monogeneans.

## Supporting Information

File S1 **PDF of all figures and measurements of clamp and body surfaces. Total number of figures: 120.**
(PDF)
