# Supplementary material for: The Monogenean Which Lost Its Clamps
Source: PLoS One. 2013 Nov 22;8(11):e79155. doi: 10.1371/journal.pone.0079155 (PMC3838368; doi:10.1371/journal.pone.0079155)

The monogenean which lost its clamps

Supplementary Information file

120 line drawings; for each drawing are indicated:

Family

Species

Body Surface (in μm^2)

Clamp Surface (in μm^2)

Ratio

Reference

Jean-Lou Justine [1 *], Chahrazed Rahmouni [1], Delphine Gey [2], Charlotte Schoelinck [1,3], Eric P. Hoberg [4]

[1] UMR 7138 "Systématique, Adaptation, Évolution", Muséum National d'Histoire Naturelle, CP 51, 55 rue Buffon, 75231 Paris cedex 05, France

[2] UMS 2700 Service de Systématique moléculaire, Muséum National d'Histoire Naturelle, CP 26, 57 rue Cuvier, 75231 Paris cedex 05, France

[3] Molecular Biology, Aquatic Animal Health, Fisheries and Oceans Canada, 343, Avenue de l'Université, Moncton N.B. E1C 9B6, Canada

[4] US National Parasite Collection, USDA, Agricultural Research Service, BARC East No. 1180, 10300 Baltimore Avenue, Beltsville, MD, 20705, USA

* Corresponding author. email: justine@mnhn.fr

Family: Allodiscocotylidae
Species: Allodiscocotyla chorinemi Yamaguti, 1953
Body Surface: 221,079
Clamp Surface: 23,301
Ratio: 10.54
Reference: Yamaguti, 1963, p. 547

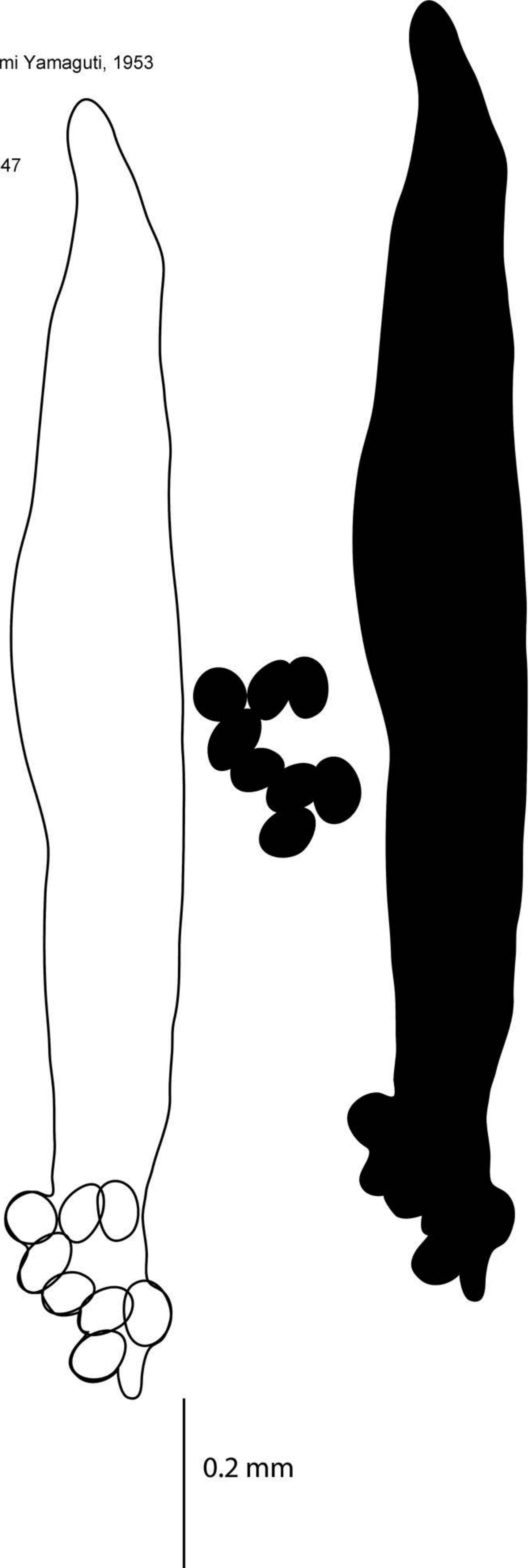

Family: Allodiscocotylidae
Species: *Allodiscocotyla diacanthi* Unnithan, 1962
Body Surface: 891,326
Clamp Surface: 53,724
Ratio: 6.03
Reference: Yamaguti,1963, p. 547

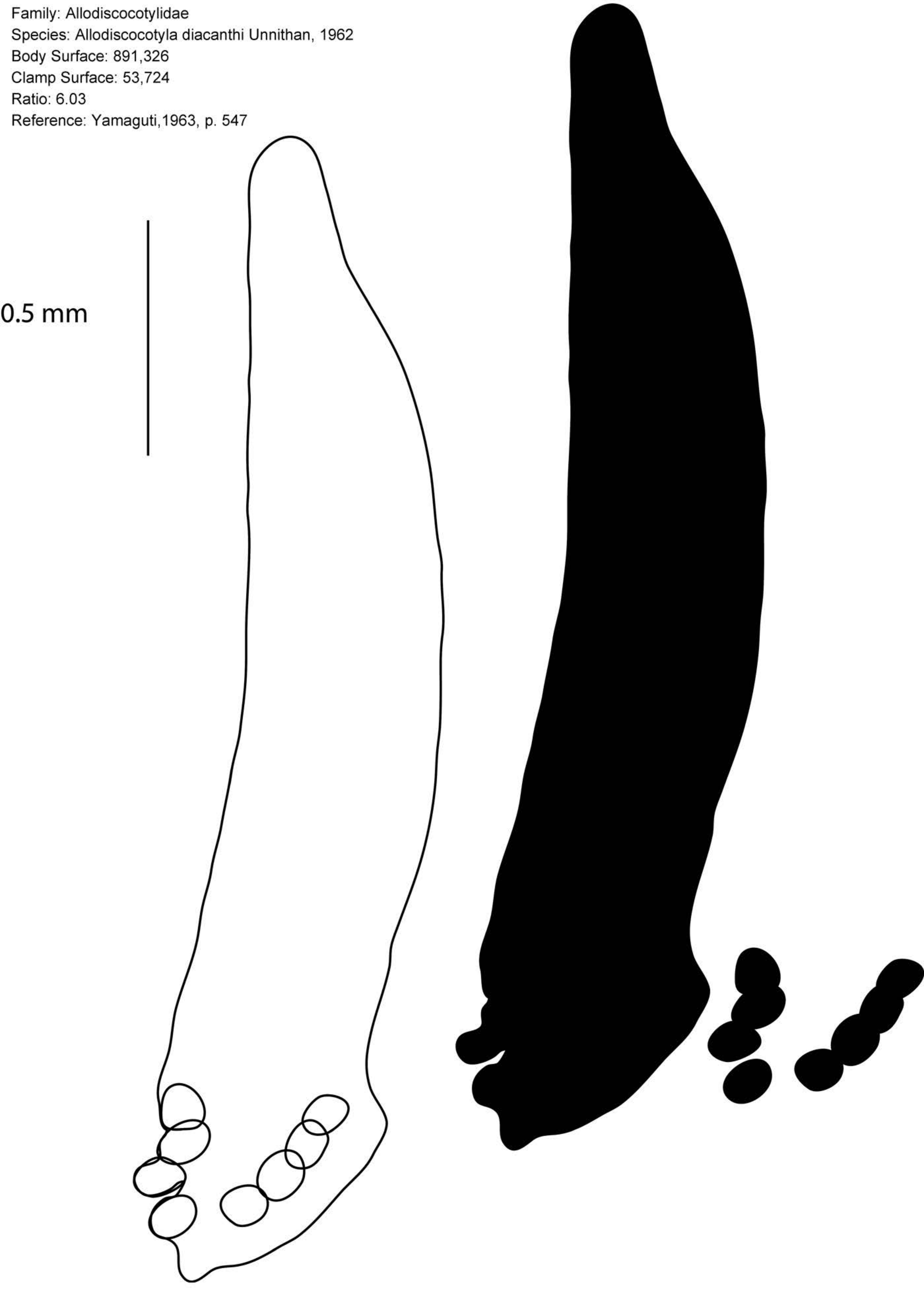

Family: Allodiscocotylidae
Species: Allodiscocotyla lae Yamaguti, 1968
Body Surface: 525,572
Clamp Surface:13,728
Ratio: 2.61
Reference: Yamaguti, 1968, p. 251

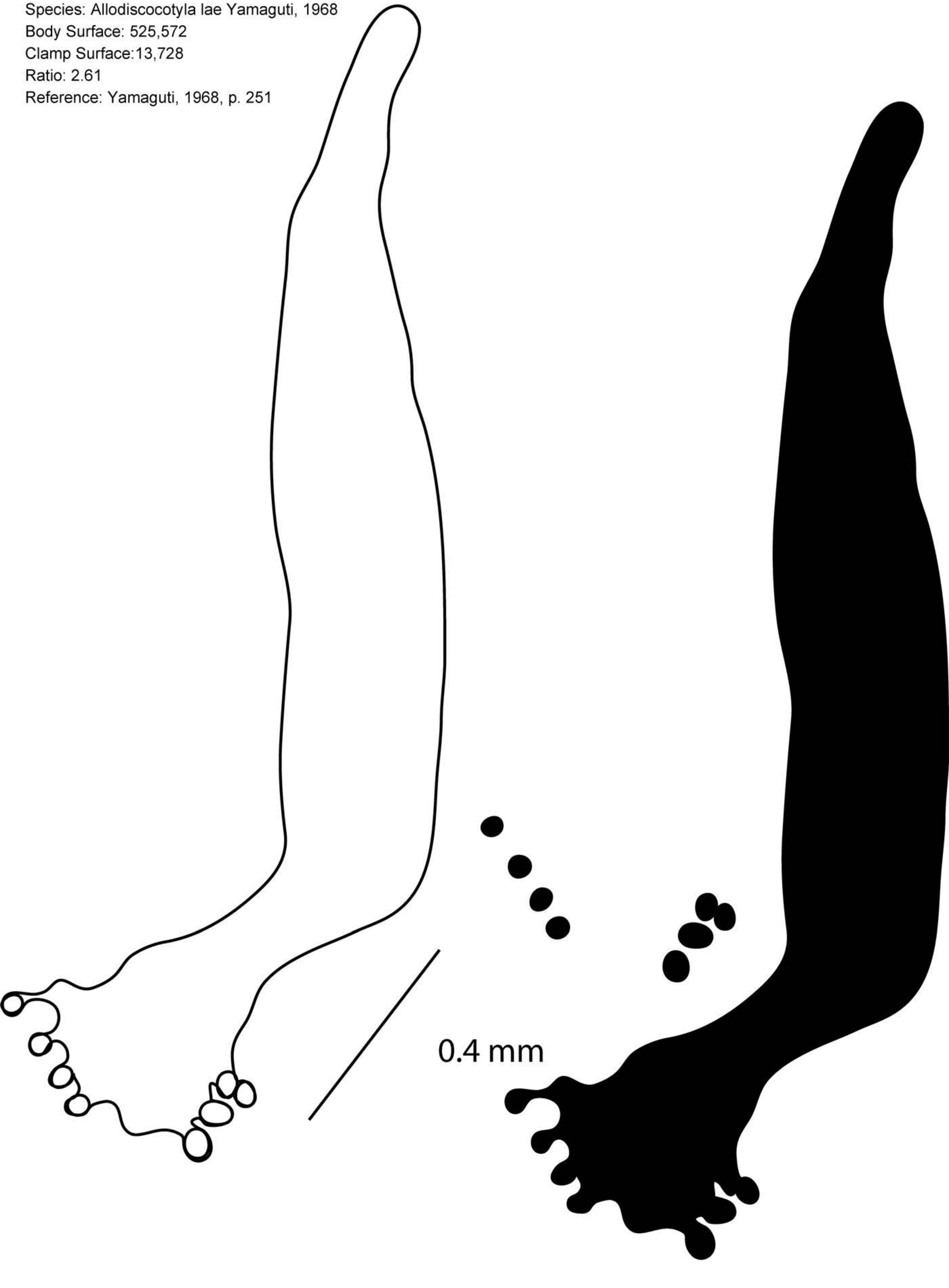

Family: Allodiscocotylidae
Species: Camopia rachycentri Lebedev, 1970
Body Surface: 12,492,318
Clamp Surface: 150,559
Ratio: 1.21
Reference: Lebedev, 1986, p. 152

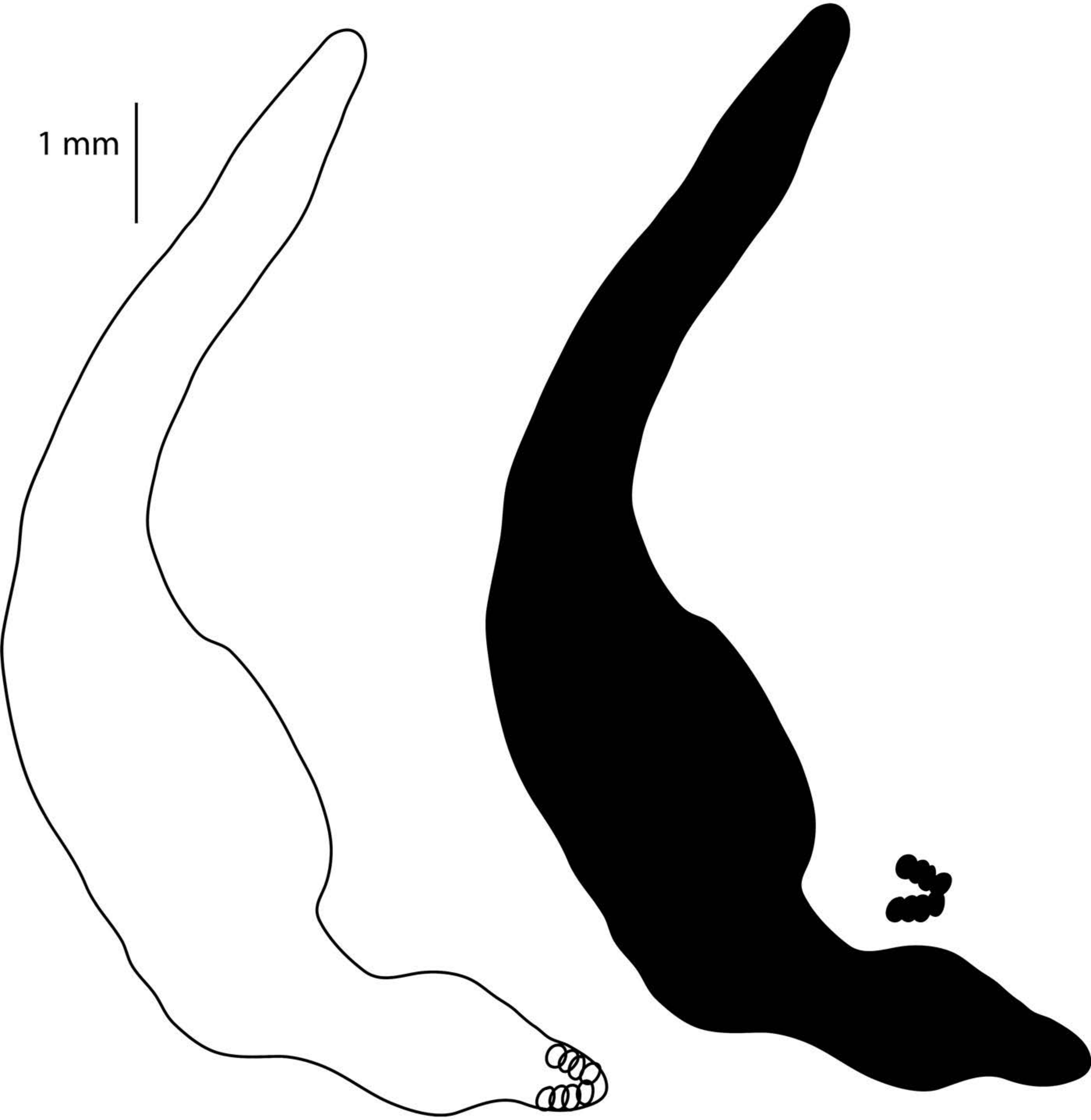

Family: Allodiscocotylidae
Species: Metacamopia indica (Unnithan, 1962) Lebedev, 1972
Body Surface: 1488158 ; Clamp Surface: 13866
Ratio: 0,93
Reference: Lebedev, 1986 , p154

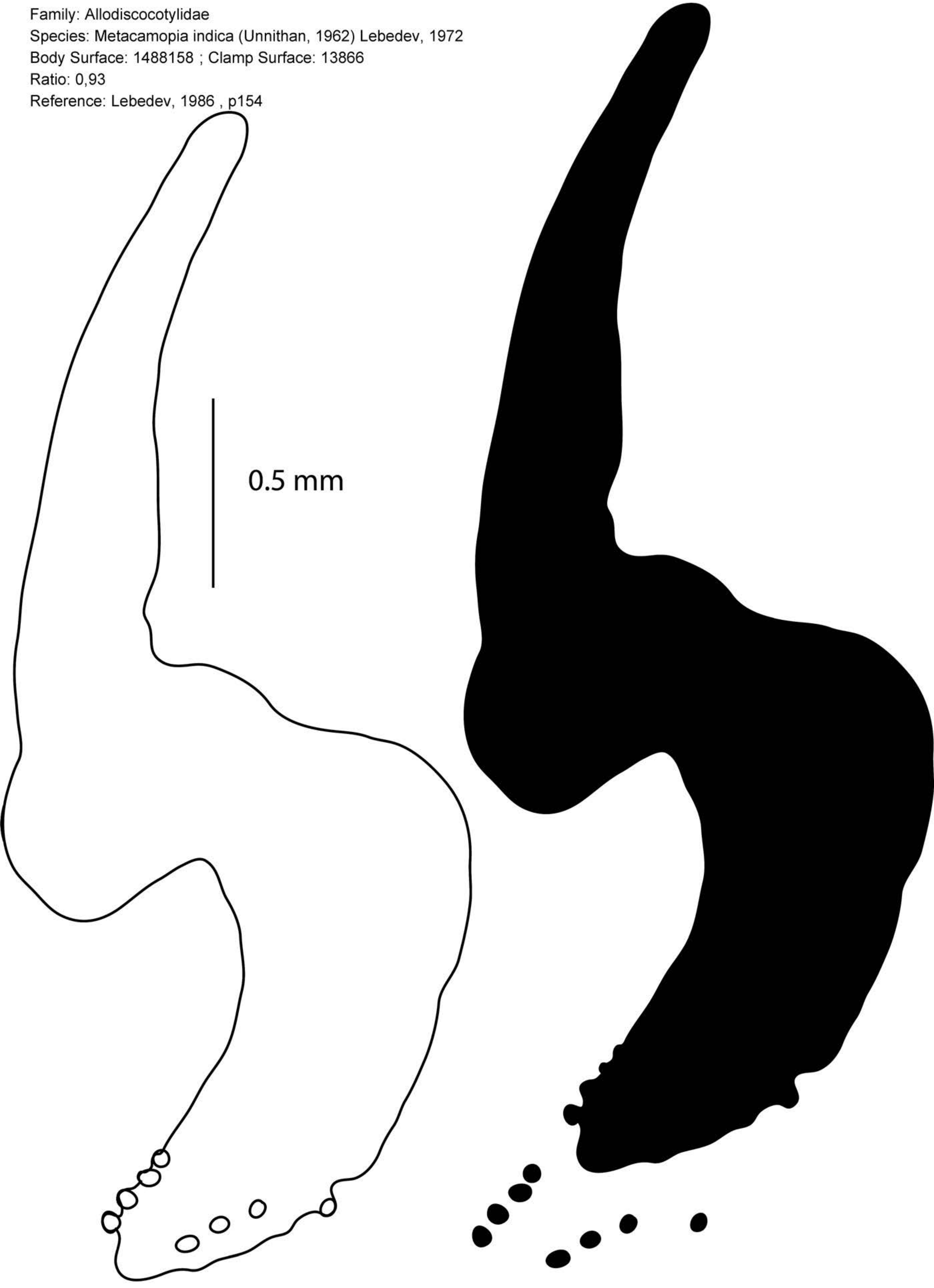

Family: Allodiscocotylidae
Species: Metacamopia oligoplites Takemoto, Amato & Luque, 1996
Body Surface: 3,273,506
Clamp Surface: 62,243
Ratio: 1.90
Reference: Takemoto, 1996, p. 167

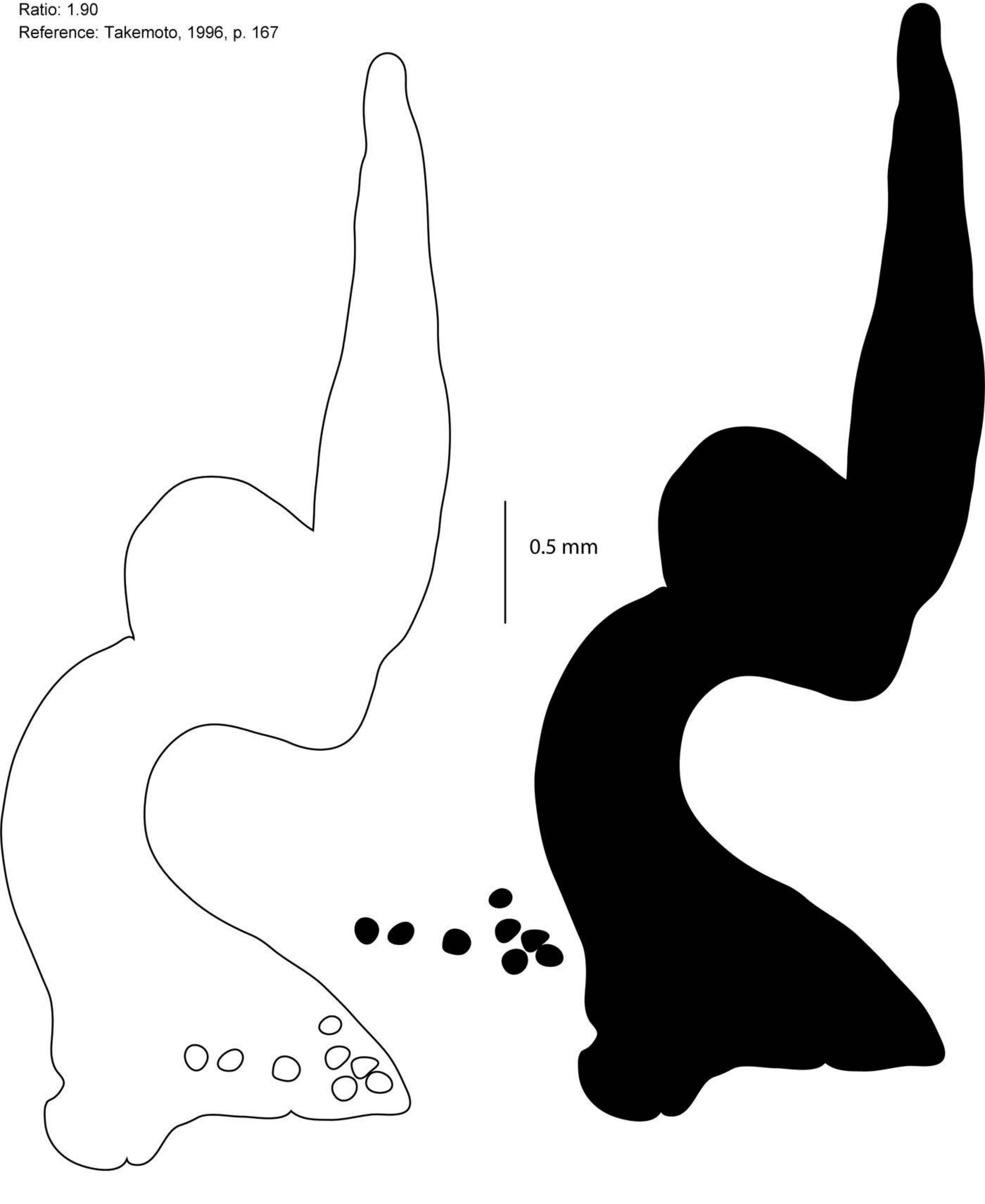

Family: Allodiscocotylidae
Species: Vallisia chorinemi Yamaguti, 1953
Body Surface:1,278,926
Clamps Surface: 16,737
Ratio: 1.31
Reference: Yamaguti, 1953, p. 65

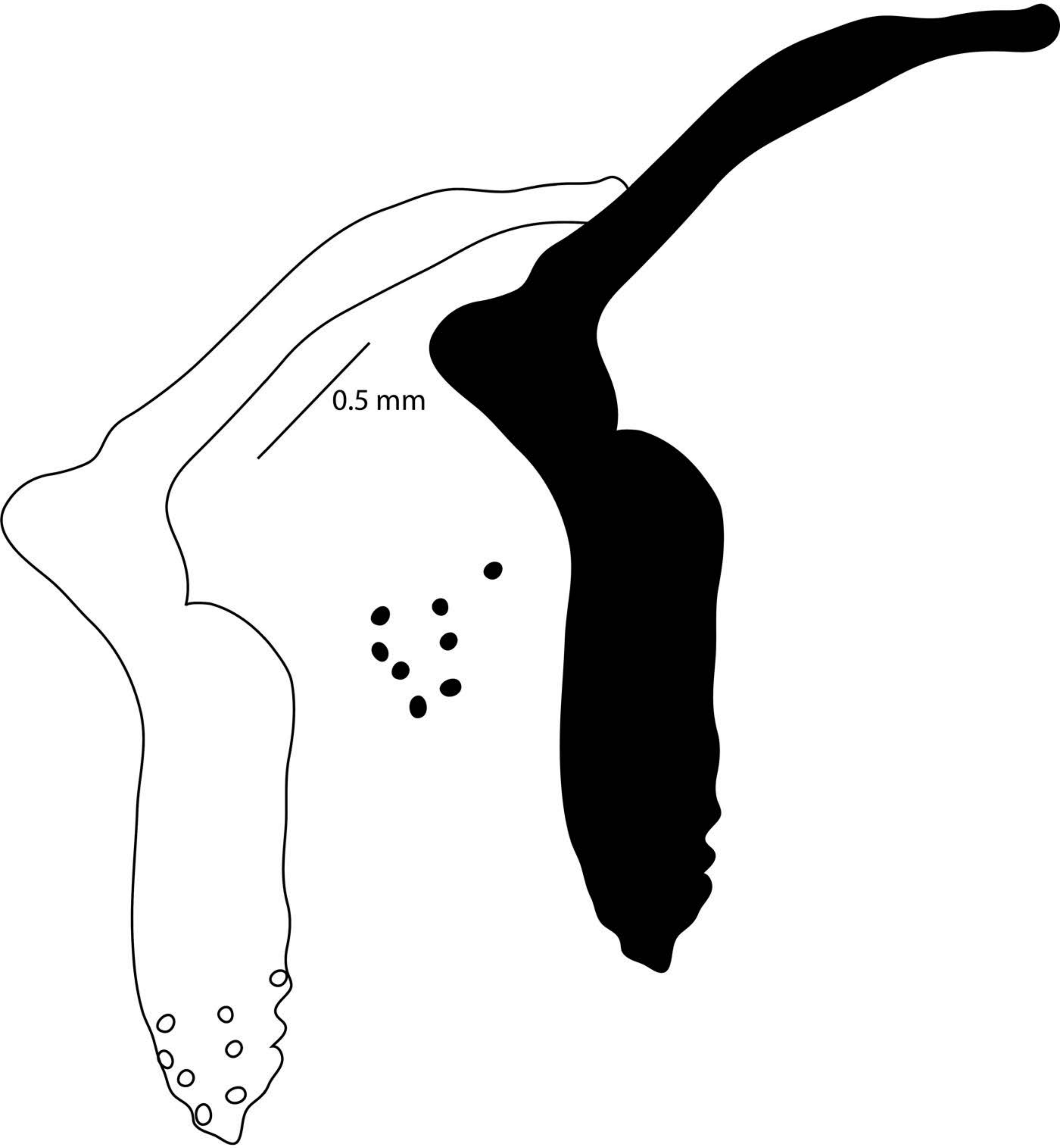

Family: Allodiscocotylidae
Species: Vallisia oligoplites Hargis, 1957
Body Surface: 4,944,569
Clamp Surface: 248,805
Ratio: 5.03
reference: Hargis, 1957, p. 7

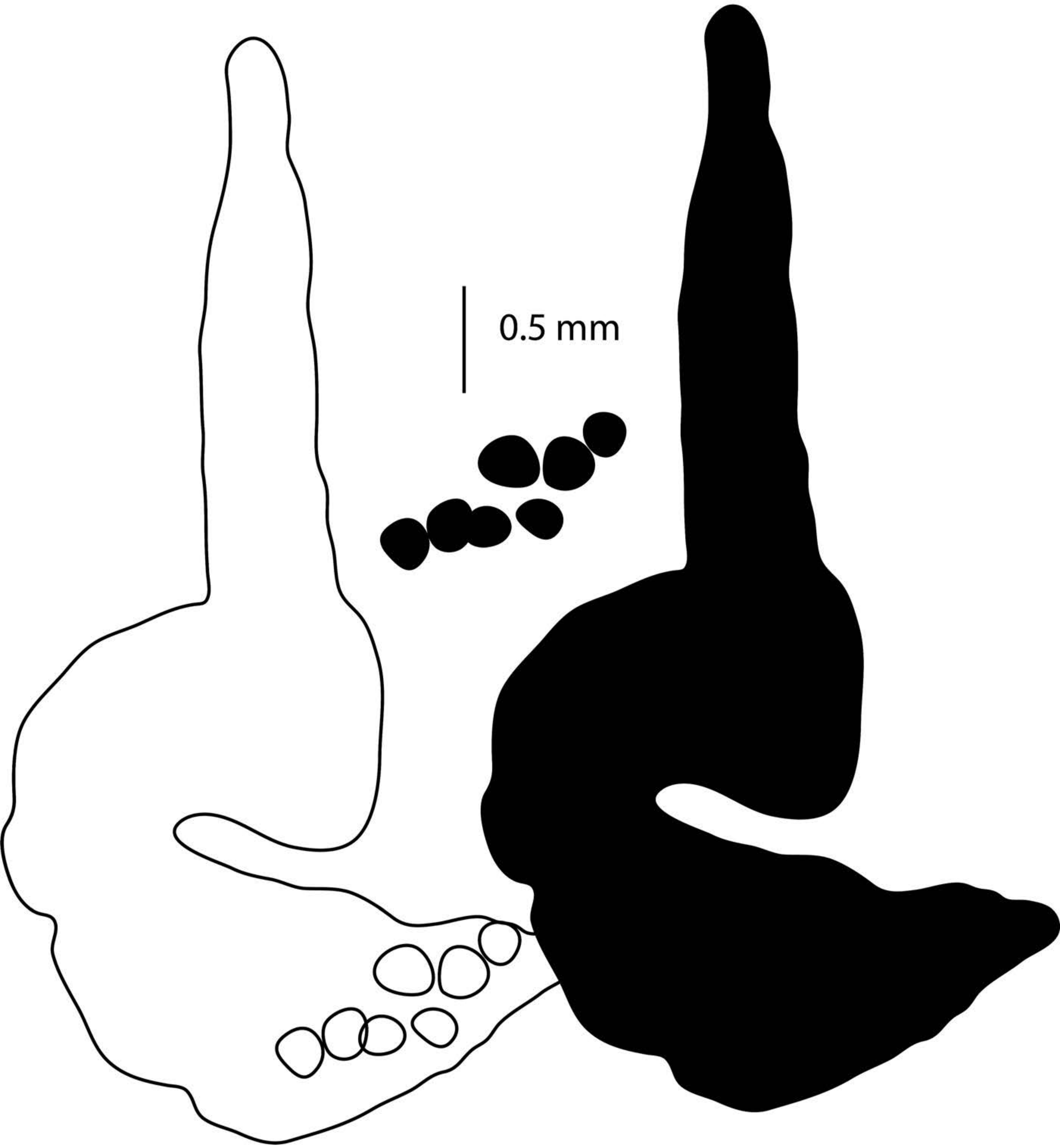

Family: Allodiscocotylidae
Species: Vallisia riojai Caballero & Bravo-Hollis, 1963
Body Surface: 971,416
Clamps Surface: 10,768
Ratio: 1.11
Reference: Caballero & Caballero, 1963, p. 175

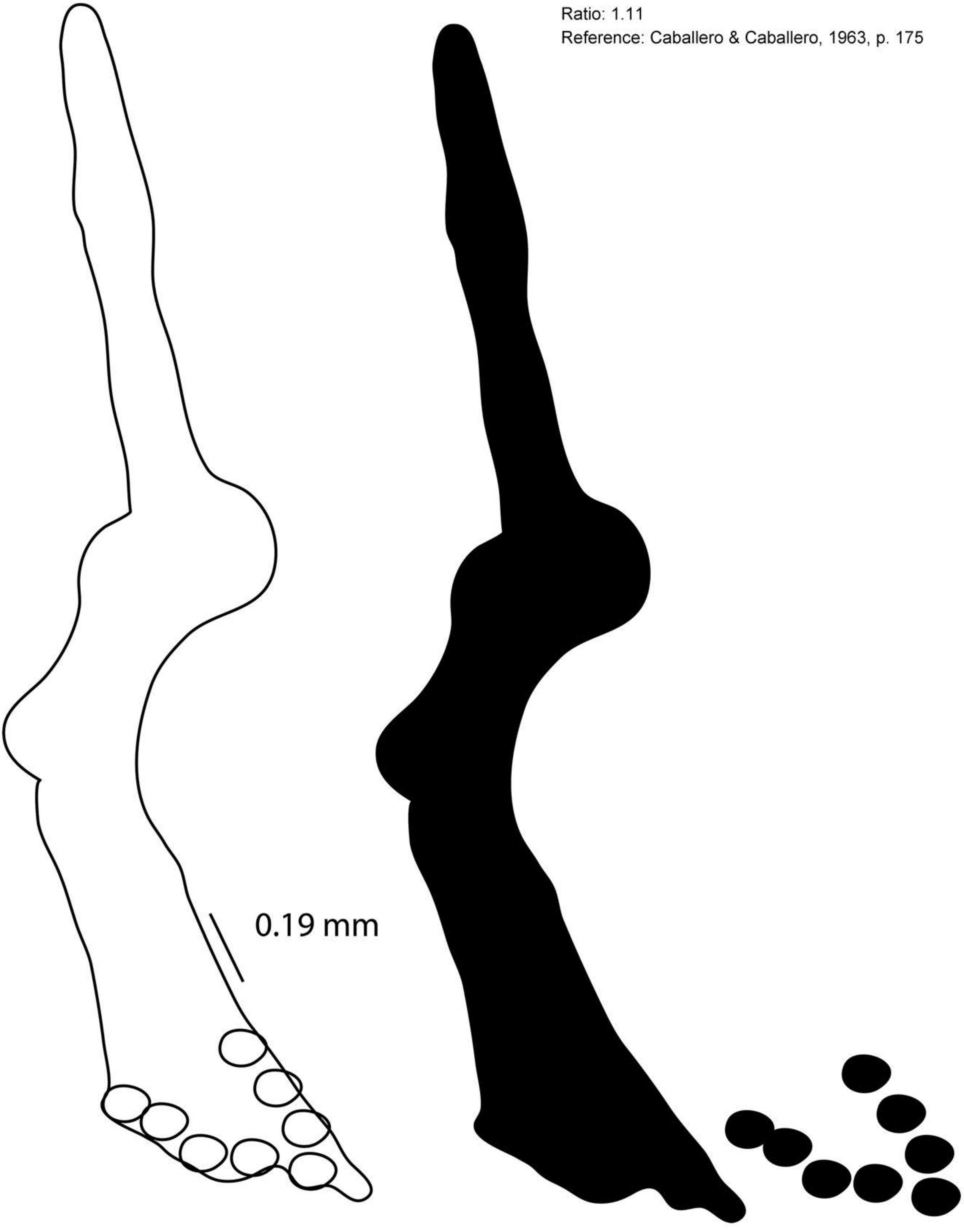

Family: Allodiscocotylidae
Species: Vallisia striata Parona & Perugia, 1890
Body Surface: 18,448,597
Clamp Surface: 373,475
Ratio: 2.02
Reference: Parona & Perugia, 1890, p. 19

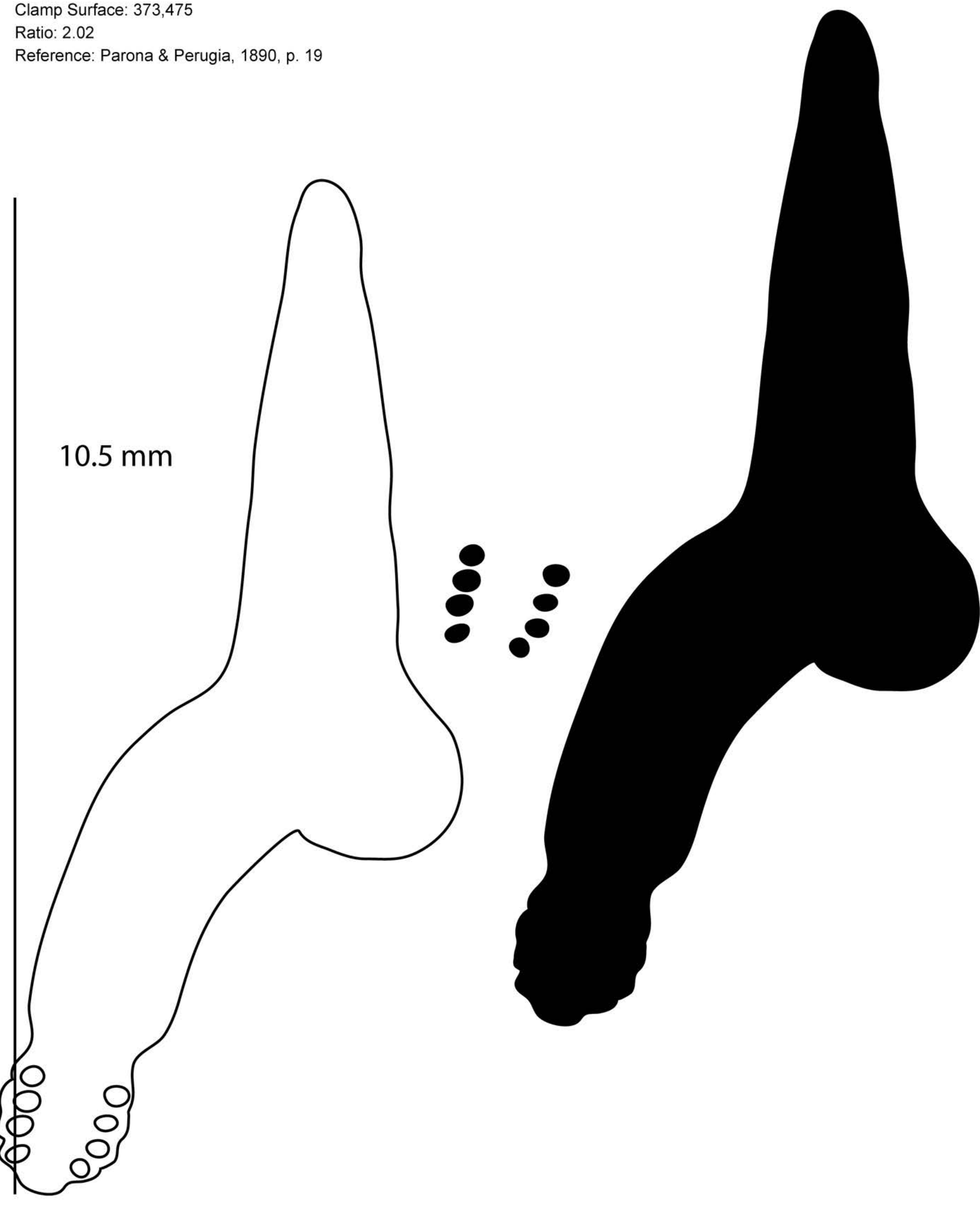

Family: Bychowskicotylidae
Species: Bychowskicotyle plectorhynchi Lebedev, 1969
Body Surface: 857,260
clamp Surface: 65,536
Ratio: 7.64
Reference: Lebedev, 1986, p. 100

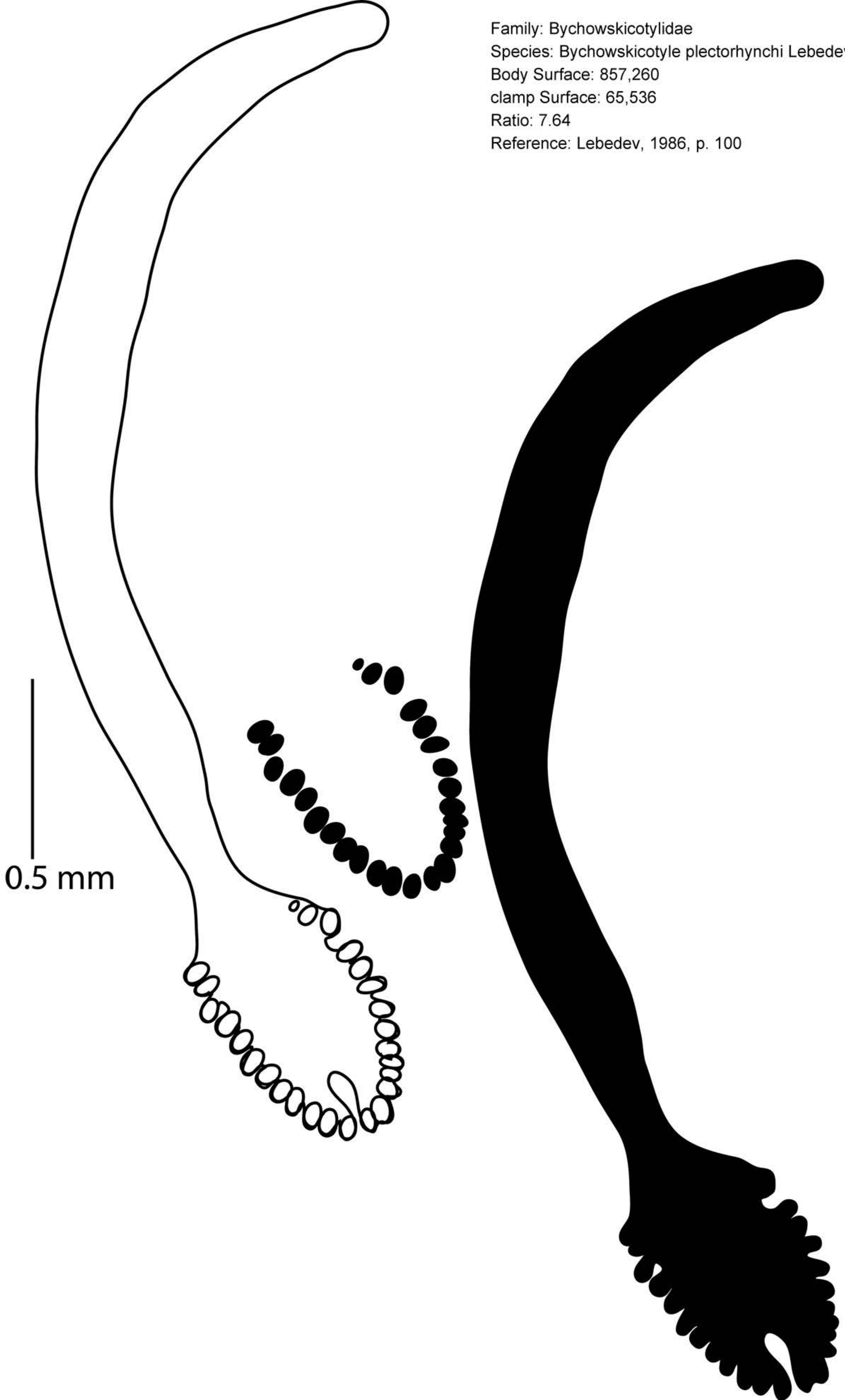

Family: Bychowskicotylidae
Species: Gaterina talaensis Lebedev, 1969
Body Surface: 619,337
Clamp Surface: 33,586
Ratio: 5.42
Reference: Lebedev, 1986, p. 101

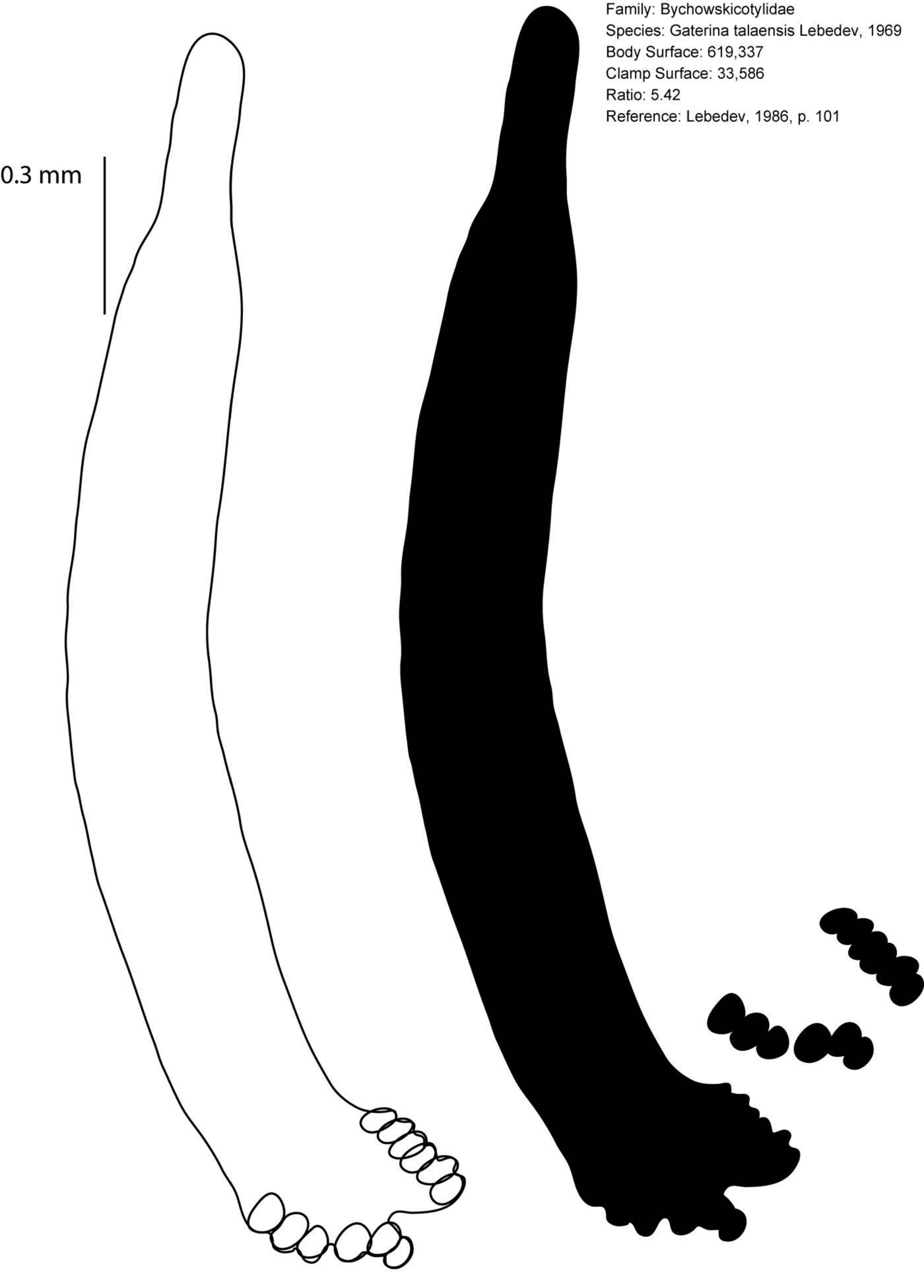

Family: Bychowskicotylidae
Species: Tonkinopsis transfretanus Lebedev, 1972
Body Surface: 1,236,435
Clamp Surface: 59,827
Ratio: 4.84
Reference: Lebedev, 1986, p. 104

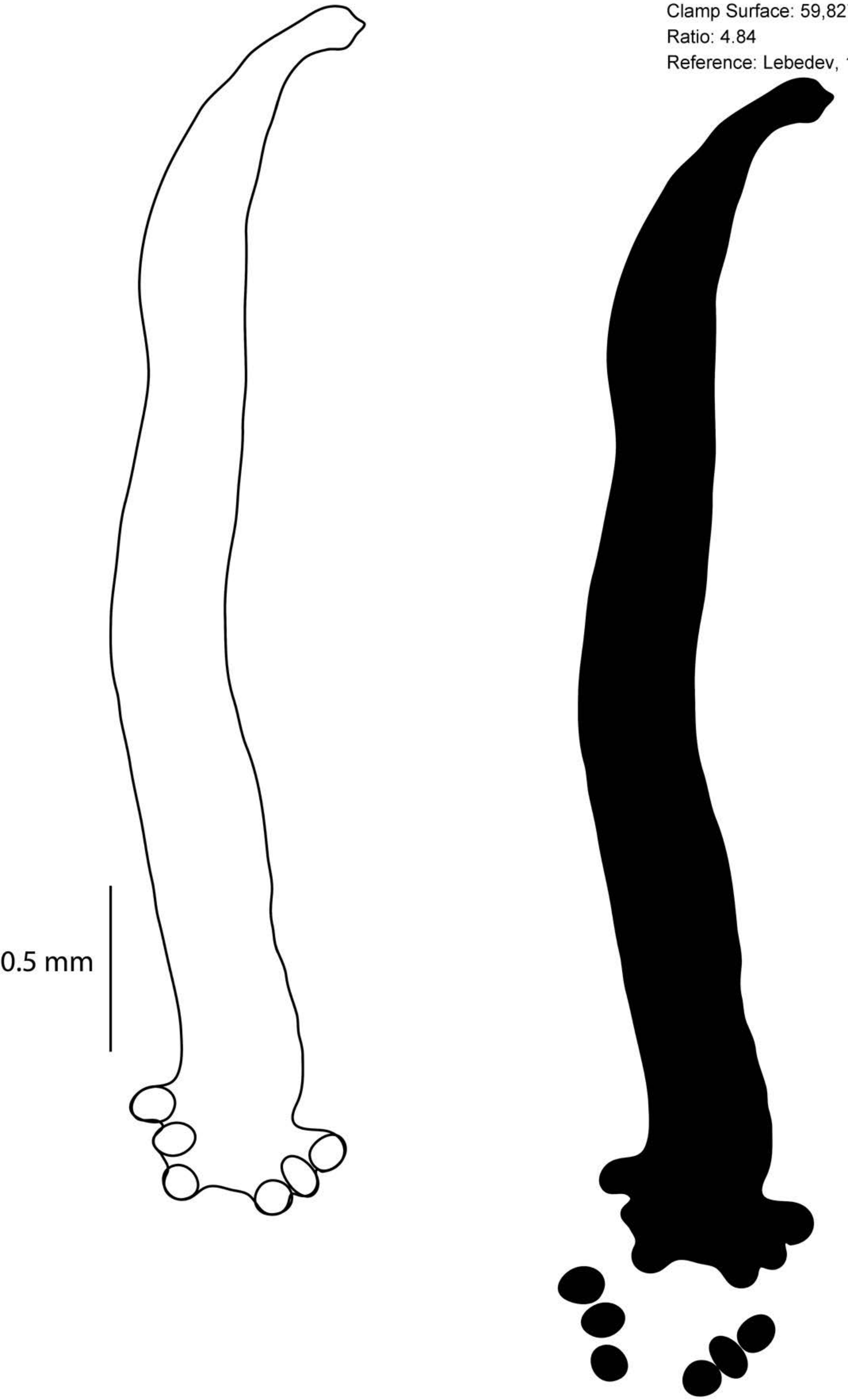

Family: Bychowskicotylidae
Species: Yamaguticotyla jucunda (Lebedev, 1969) Lebedev, 1984
Body Surface: 1,189,653
Clamp Surface: 50,151
Ratio: 4.22
Reference: Lebedev, 1986, p. 103

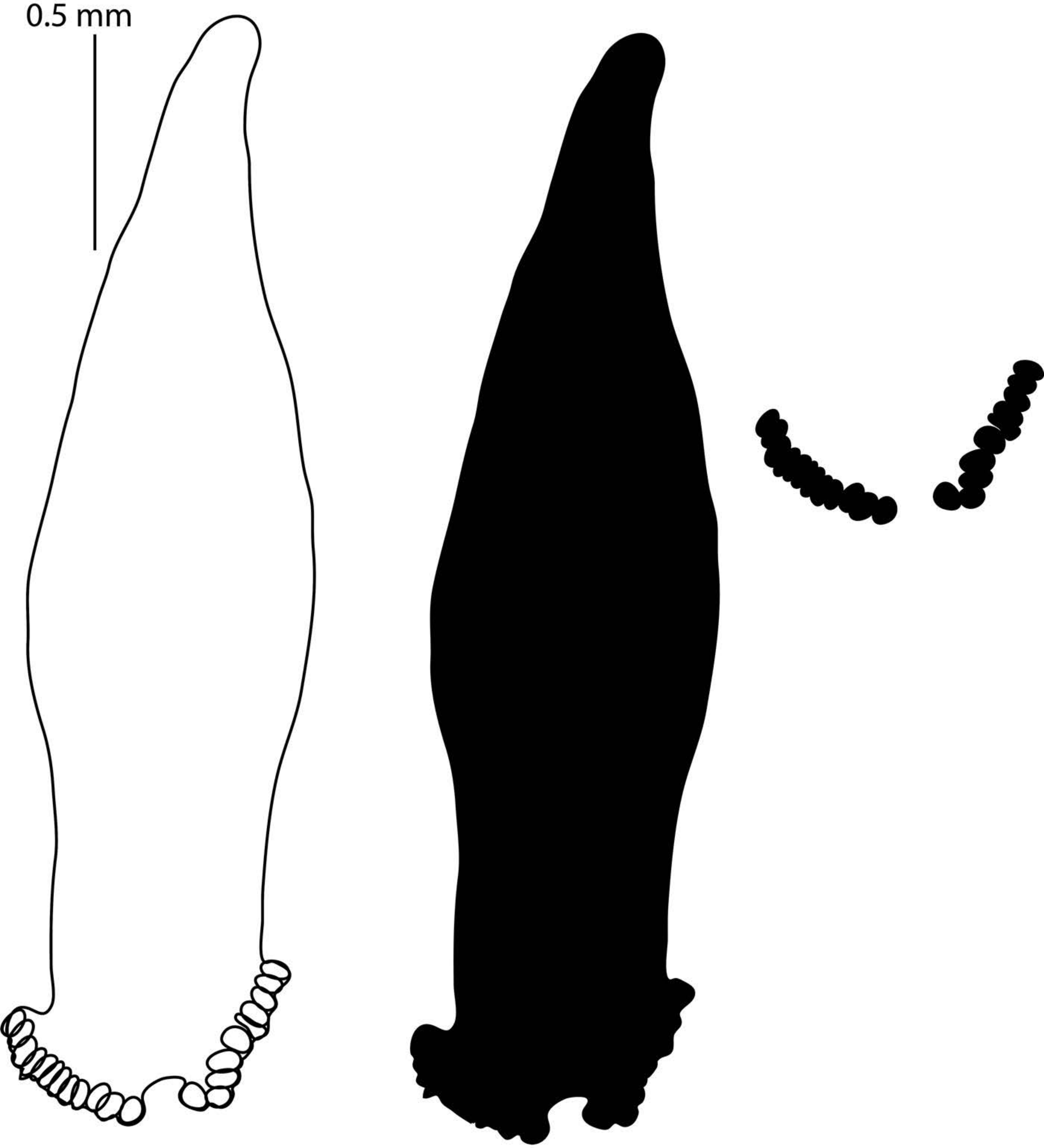

Family: Bychowskicotylidae

Species: Yamaguticotyla truncata (Goto, 1894)

Body Surface: 4,924,003 ;Clamp Surface: 87,494

Ratio: 1.78

Reference: Lebedev, 1986, p. 60

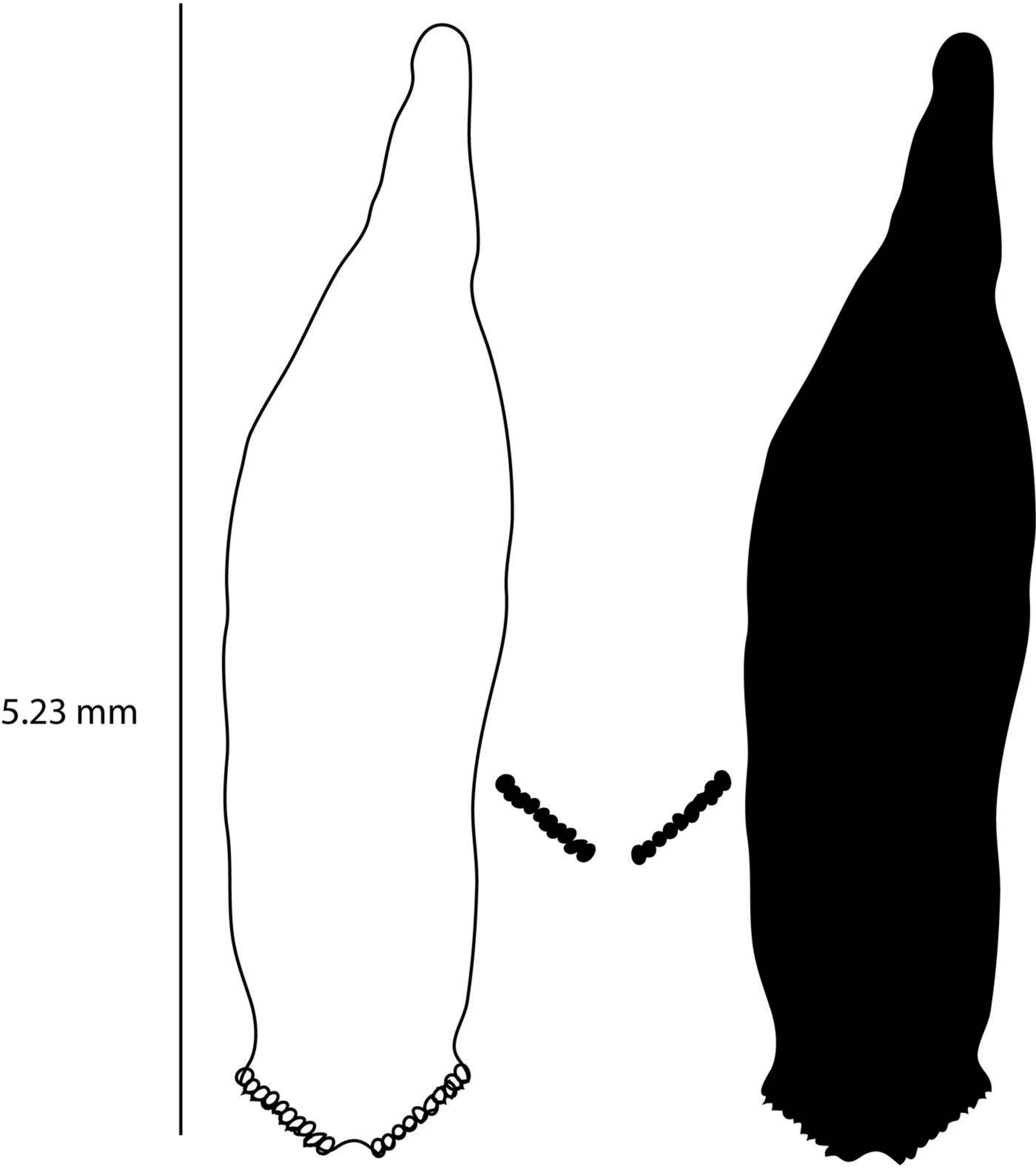

Family: Chauhaneidae
Species: Ahpua piscicola Caballero & Bravo-Hollis, 1973
Body Surface: 10,832
Clamp Surface: 404
Ratio: 3.73
Reference: Caballero & Caballero, 1973, p. 39

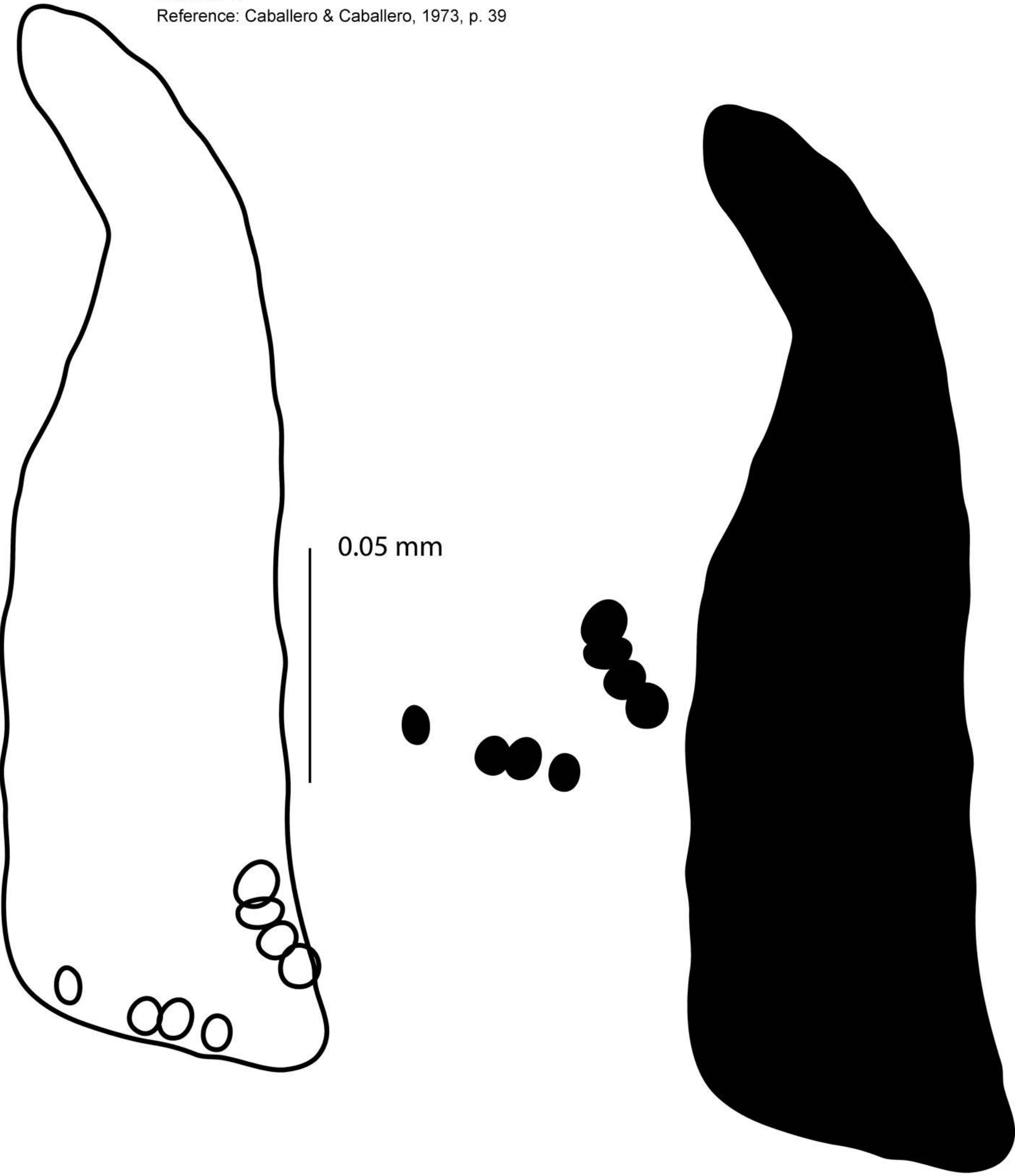

Family: Chauhaneidae
Species: Allopseudopisthogyne constricta Yamaguti, 1965
Body Surface: 4,293,278
Clamp Surface: 170,812
Ratio: 3.98
reference: Yamaguti, 1966, p. 75

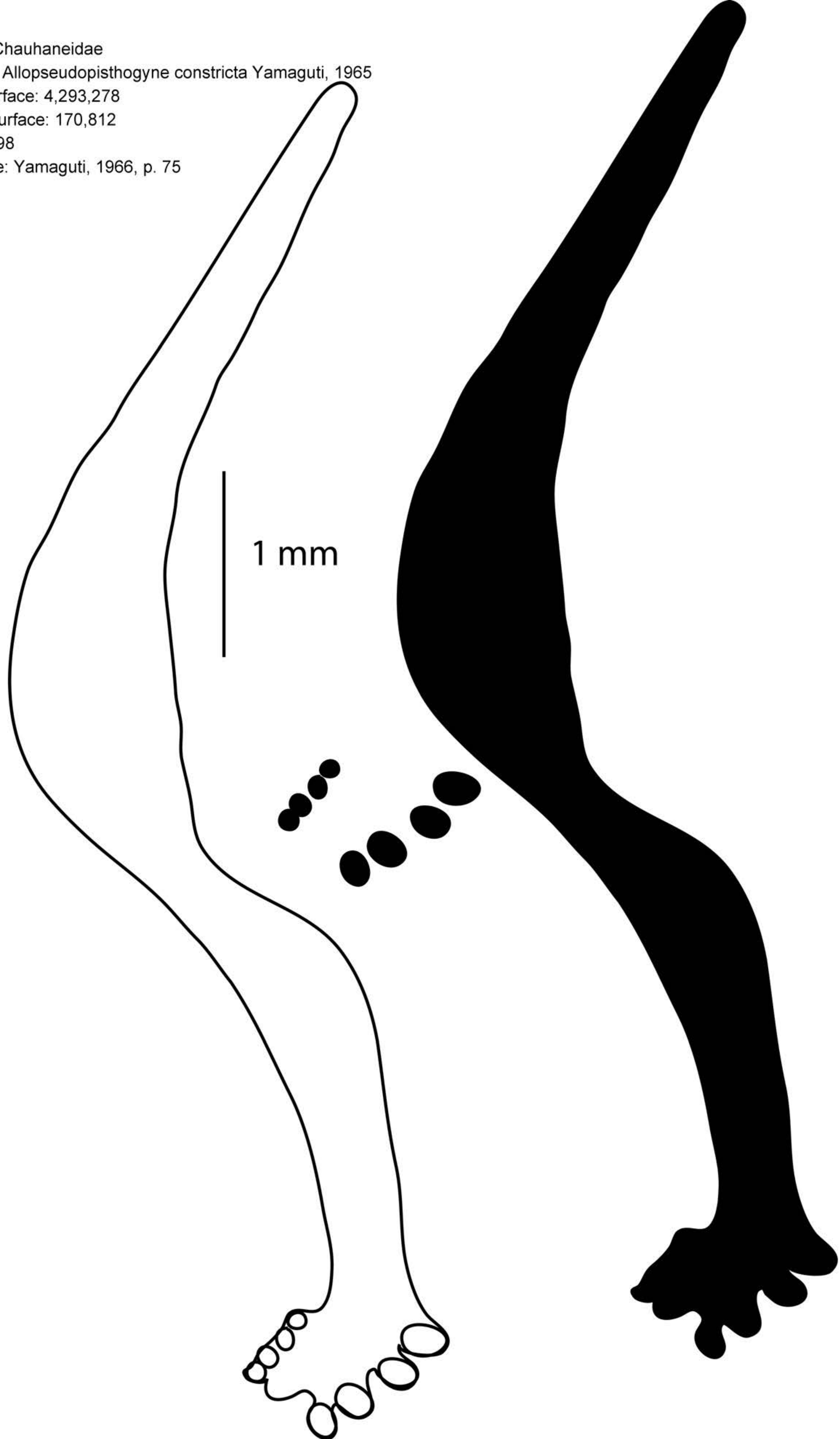

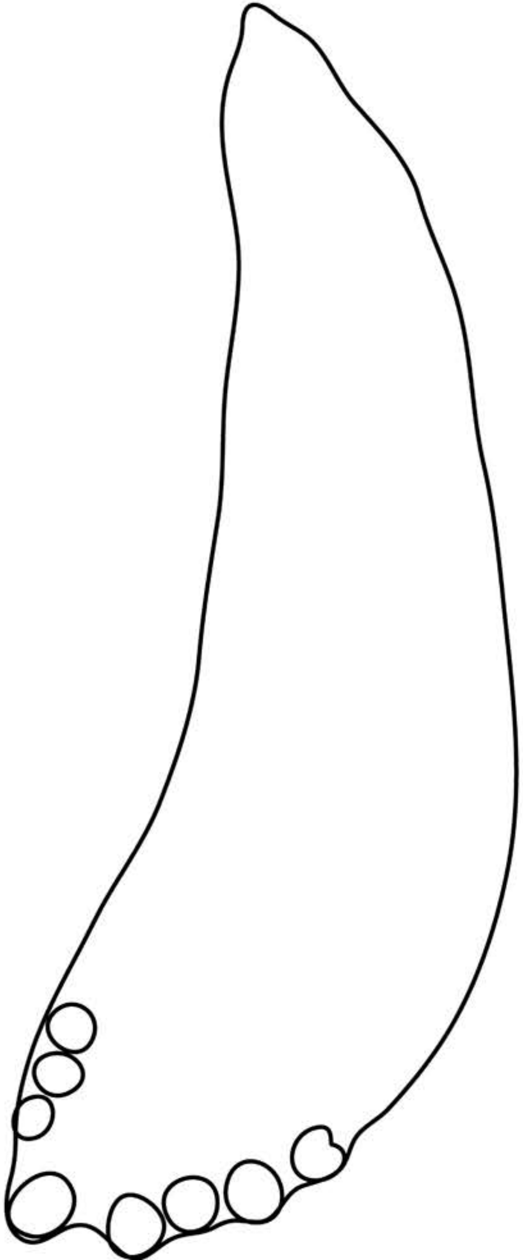

Family: Chauhaneidae
Species: Caniongiella australis (Young, 1968) Lebedev, 1976
Body Surface: 624,748
Clamp Surface: 7,033
Ratio: 1.1
Reference: Lebedev, 1986, p. 127

1.67 mm

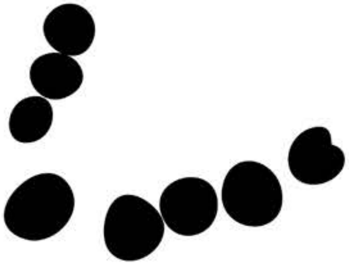

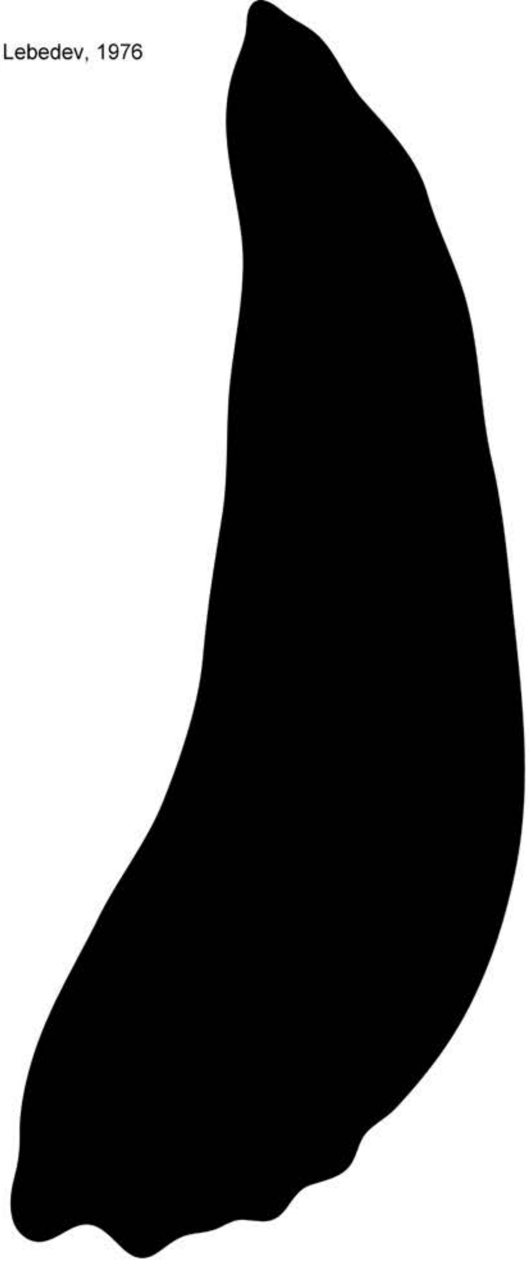

Family: Chauhaneidae
Species: *Caniongiella bychowskyi* Lebedev, 1976
Body Surface: 1,204,269
Clamp Surface: 26,531
Ratio: 2.20
Reference: Lebedev, 1986, p. 126

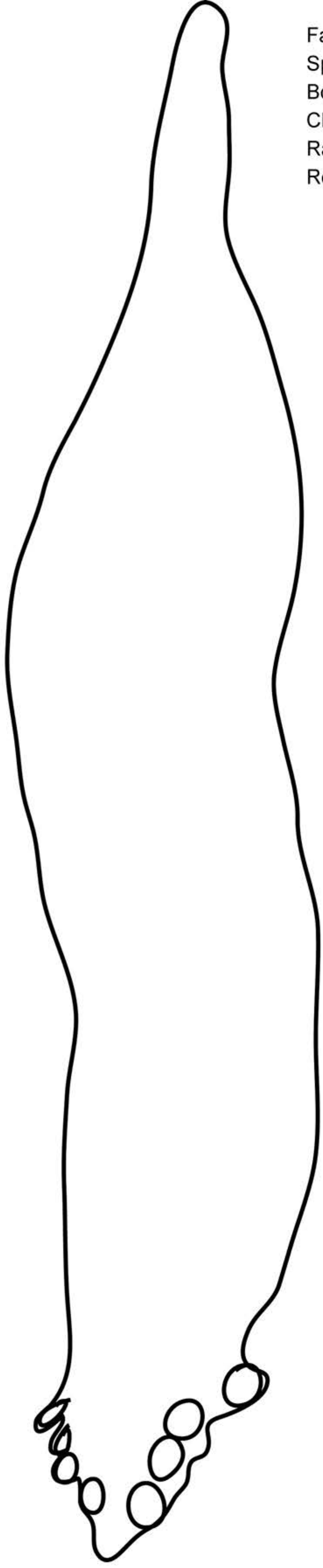

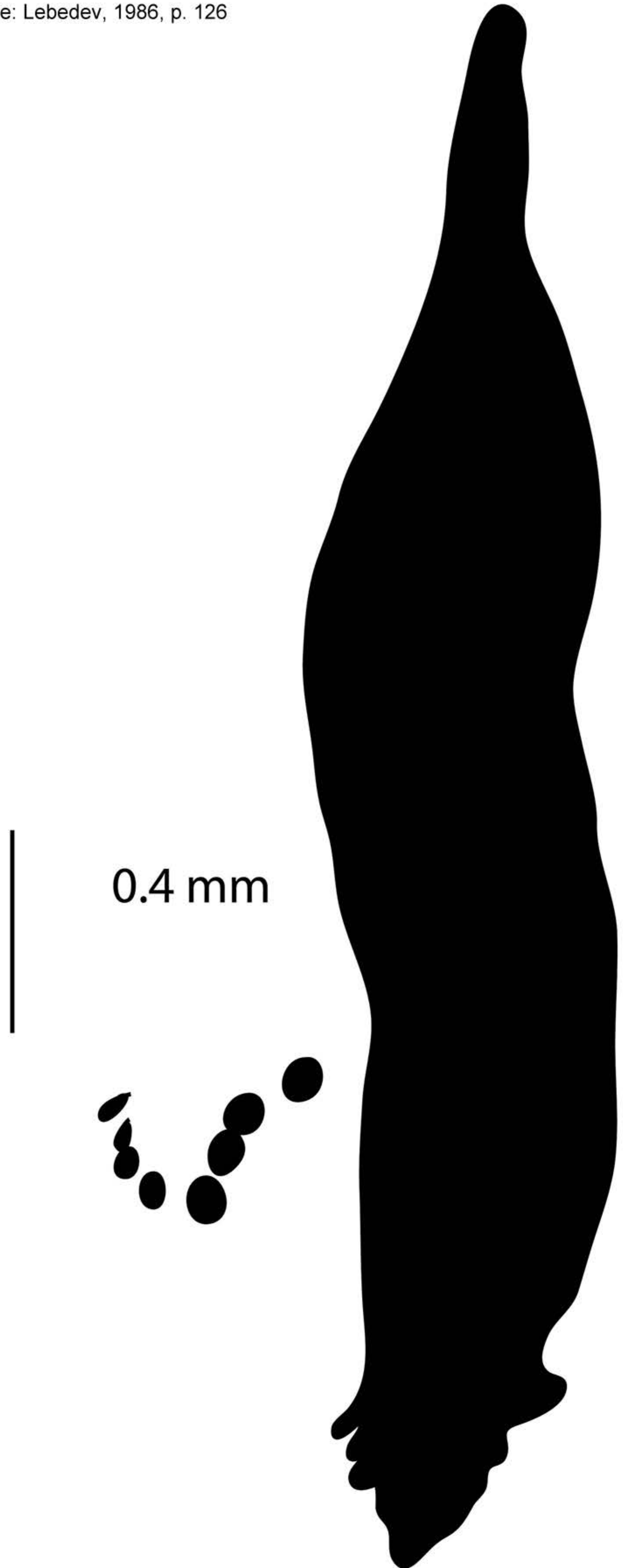

Family: Chauhaneidae
Species: *Chauhanea madrasensis* Ramalingam, 1953
Body Surface: 2,631,446
Clamp Surface: 172,925
Ratio: 6.57
Reference: Lebedev, 1986, p. 120

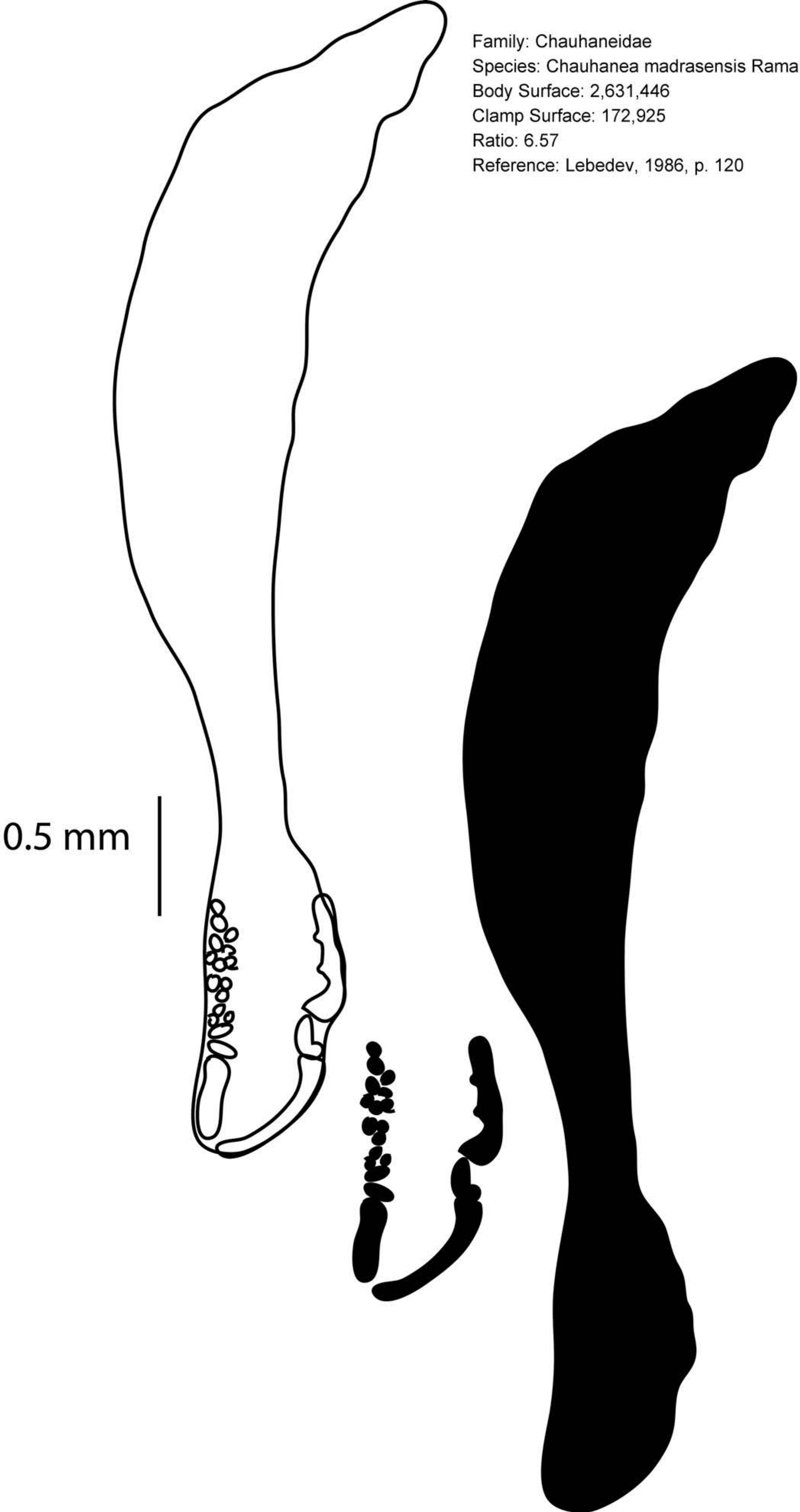

Family: Chauhaneidae
Species: *Chauhanea mediterranea* Euzet & Trilles, 1960
Body Surface: 3,309,353
Clamp Surface: 305,081
Ratio: 9.22
Reference: Euzet & Trilles, 1960, p. 190

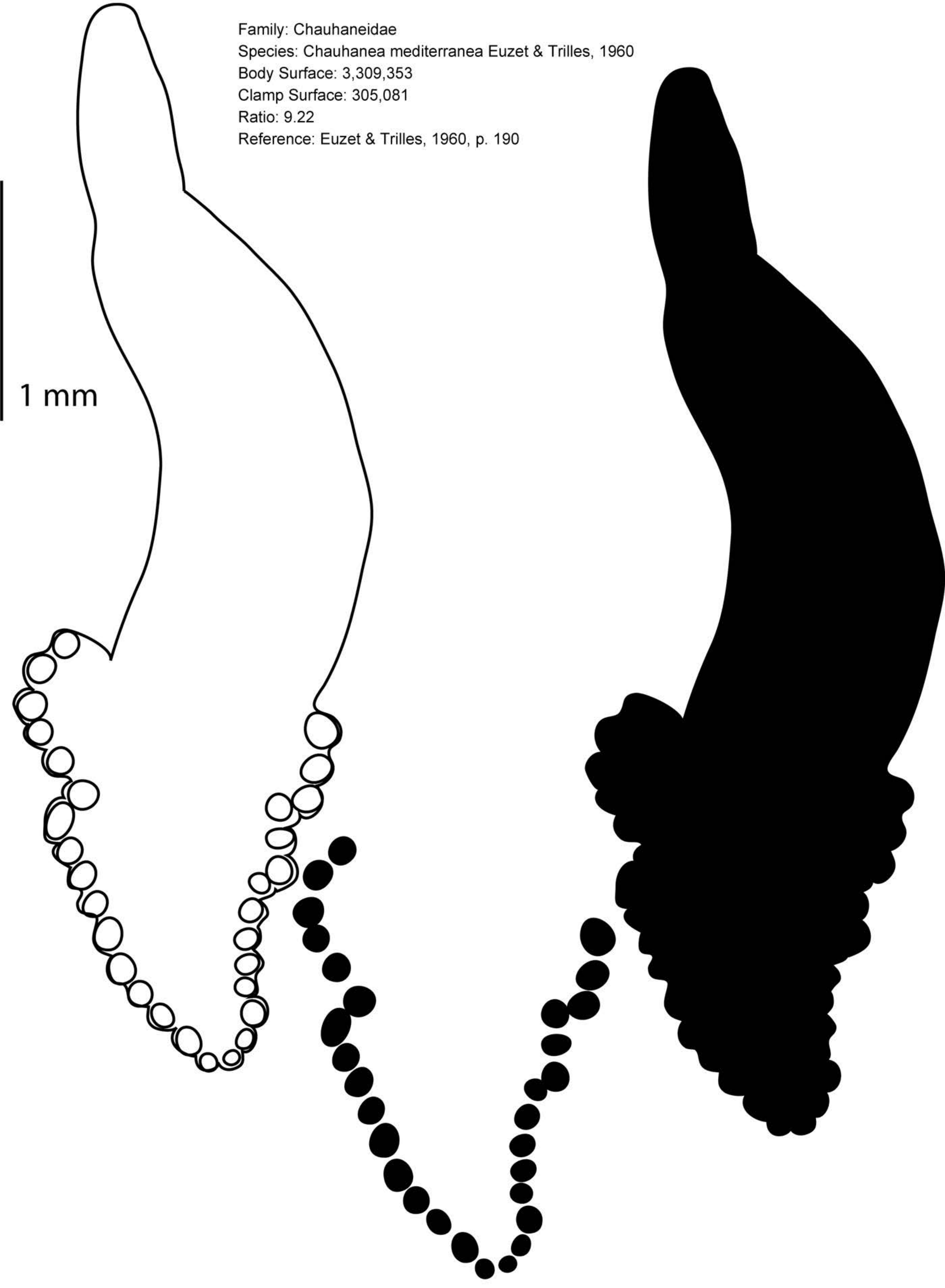

Family: Chauhaneidae
Species: *Gemmaecaputia corrugata*
Tripathi, 1959
Body Surface: 404,195
Clamp Surface: 13,015
Ratio: 3.22
Reference: Yamaguti, 1963, p. 487

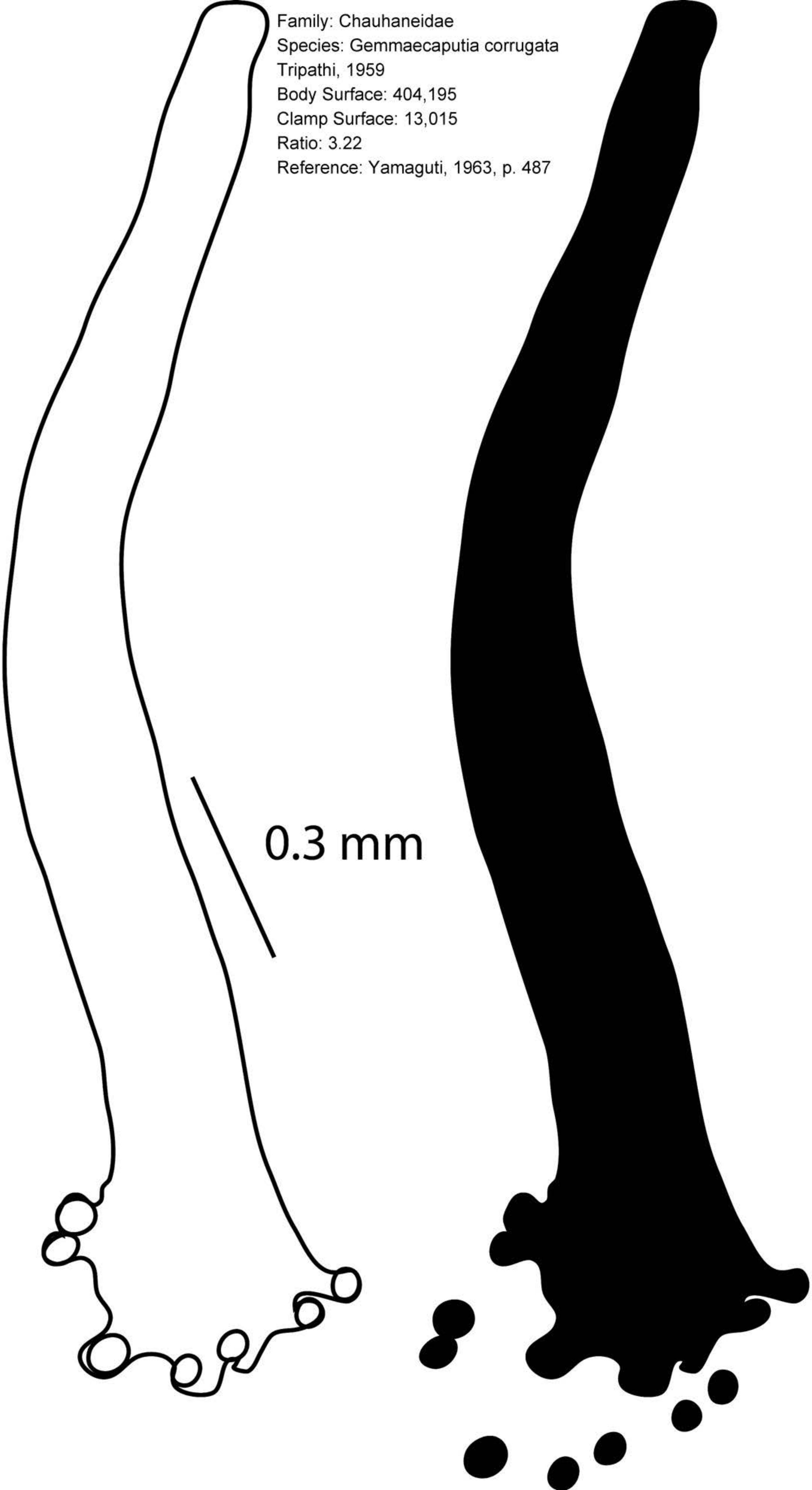

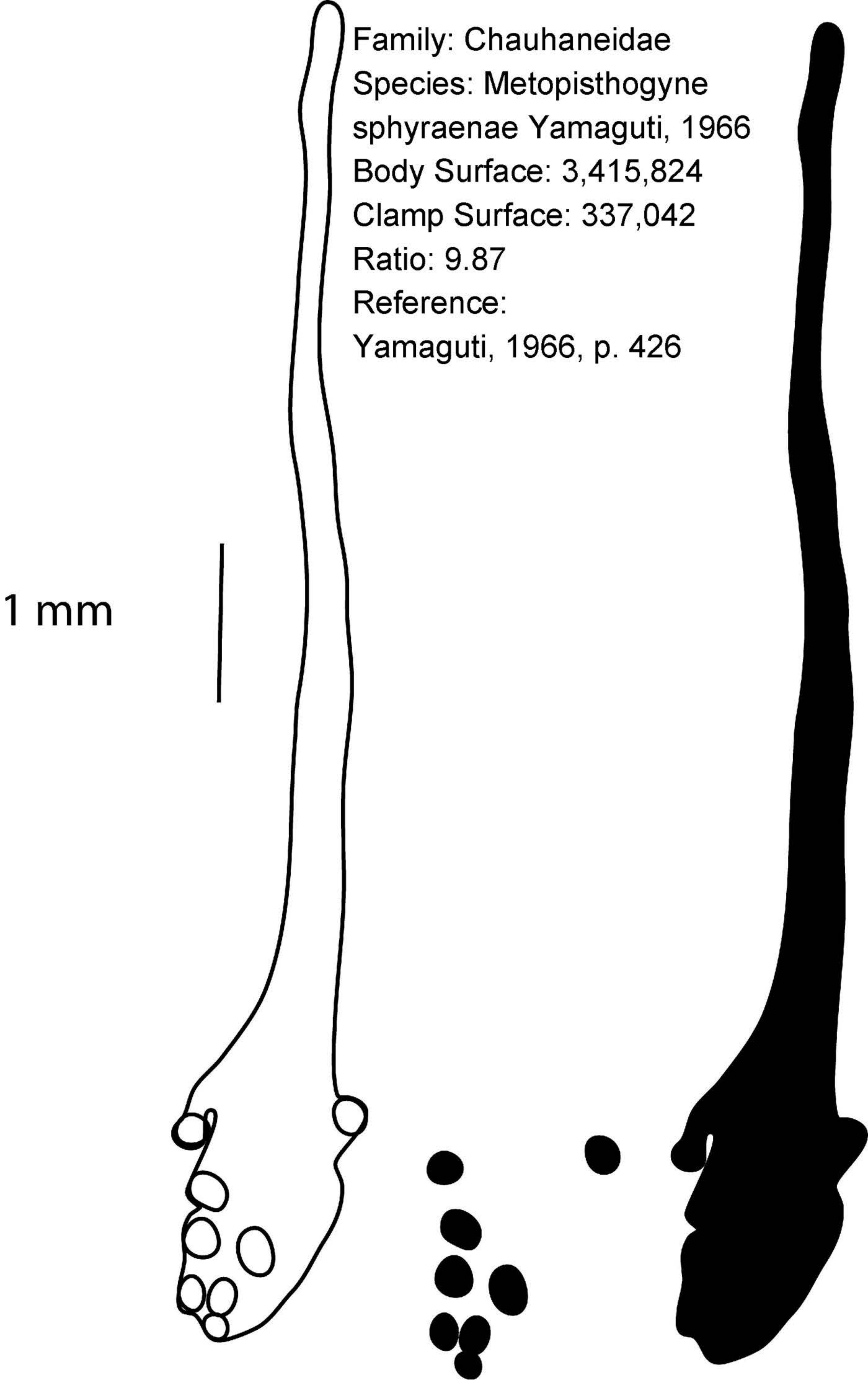
Family: Chauhaneidae
Species: Metopisthogyne
sphyraenae Yamaguti, 1966
Body Surface: 3,415,824
Clamp Surface: 337,042
Ratio: 9.87
Reference:
Yamaguti, 1966, p. 426
1 mm

Family: Chauhaneidae
Species: Oaxacotyle oaxacensis
(Caballero & Bravo, 1964) Lebedev, 1984
Body Surface: 397,303 ; Clamp Surface: 56,968
Ratio: 14.34
Reference: Lebedev, 1986, p. 138

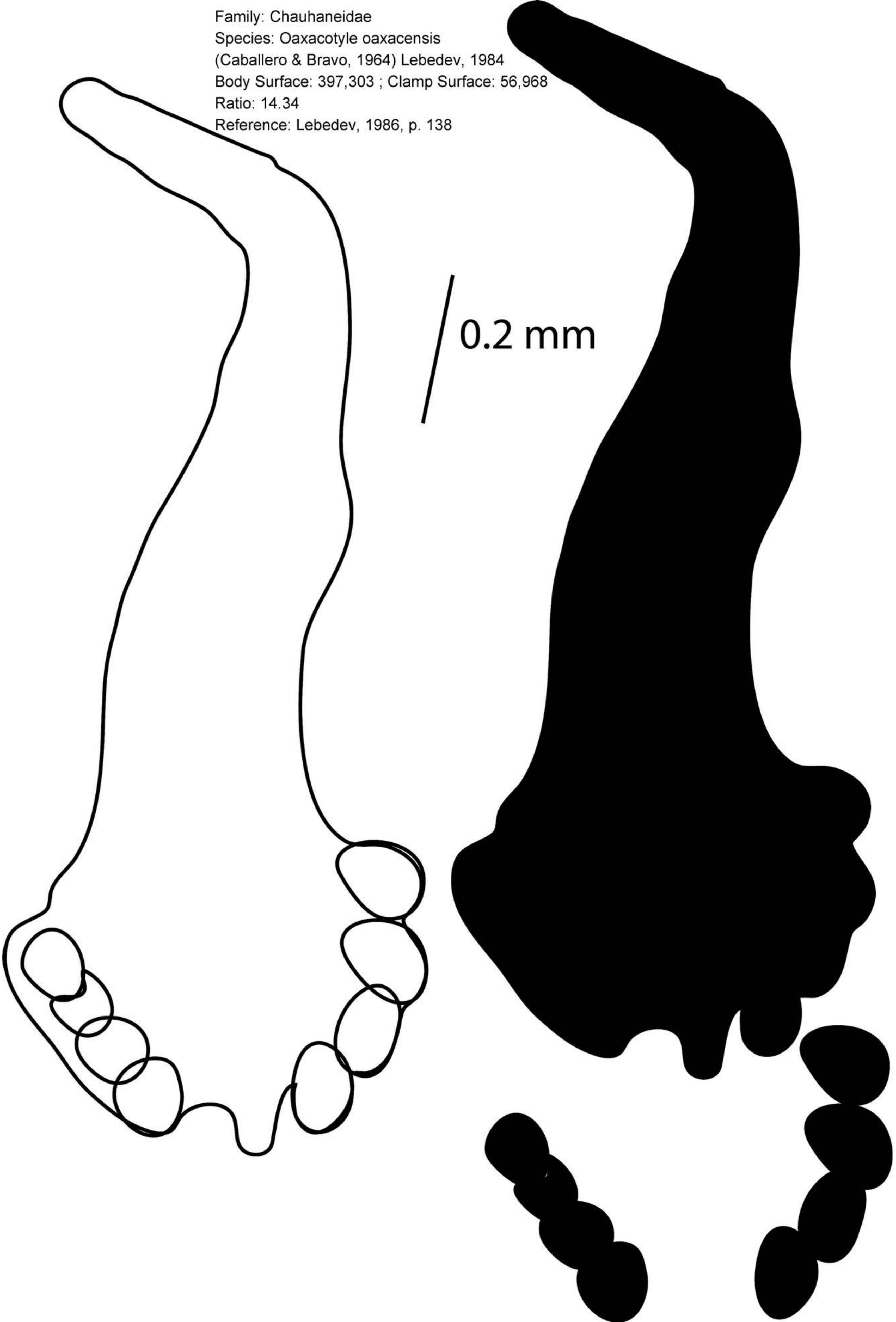

Family: Chauhaneidae
Species: *Opisthogyne keralae* Unnithan, 1962
Body Surface: 262,328
Clamp Surface:19,843
Ratio: 7.56
Reference: Unnithan, 1962, p. 318

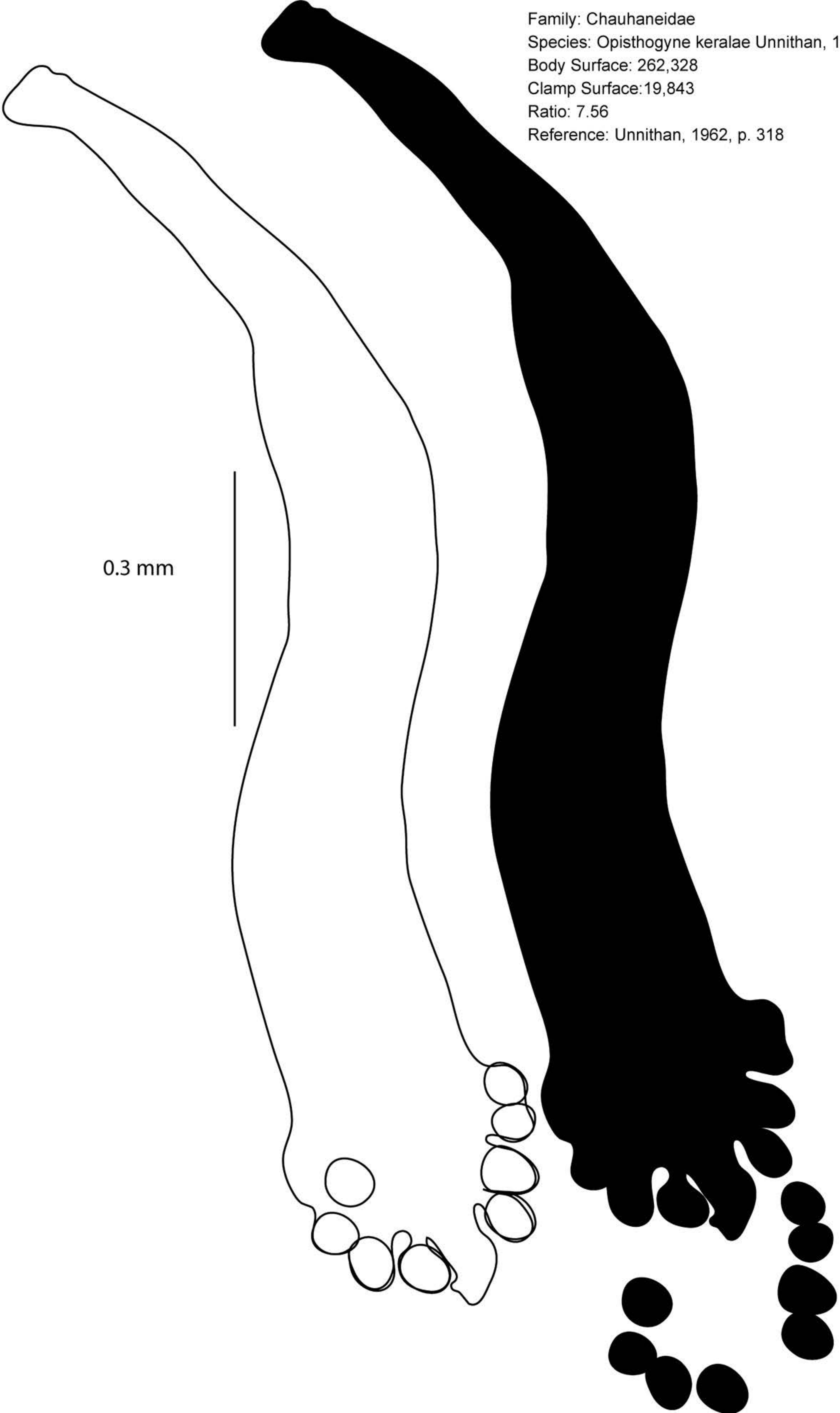

Family: Chauhaneidae
Species: Paracaniongiella brinkmanni (Unnithan, 1962) Lebedev, 1976
Body Surface: 236,647
Clamp Surface: 8,027
Ratio: 3.39
Reference: Pandey, 2008, p. 359

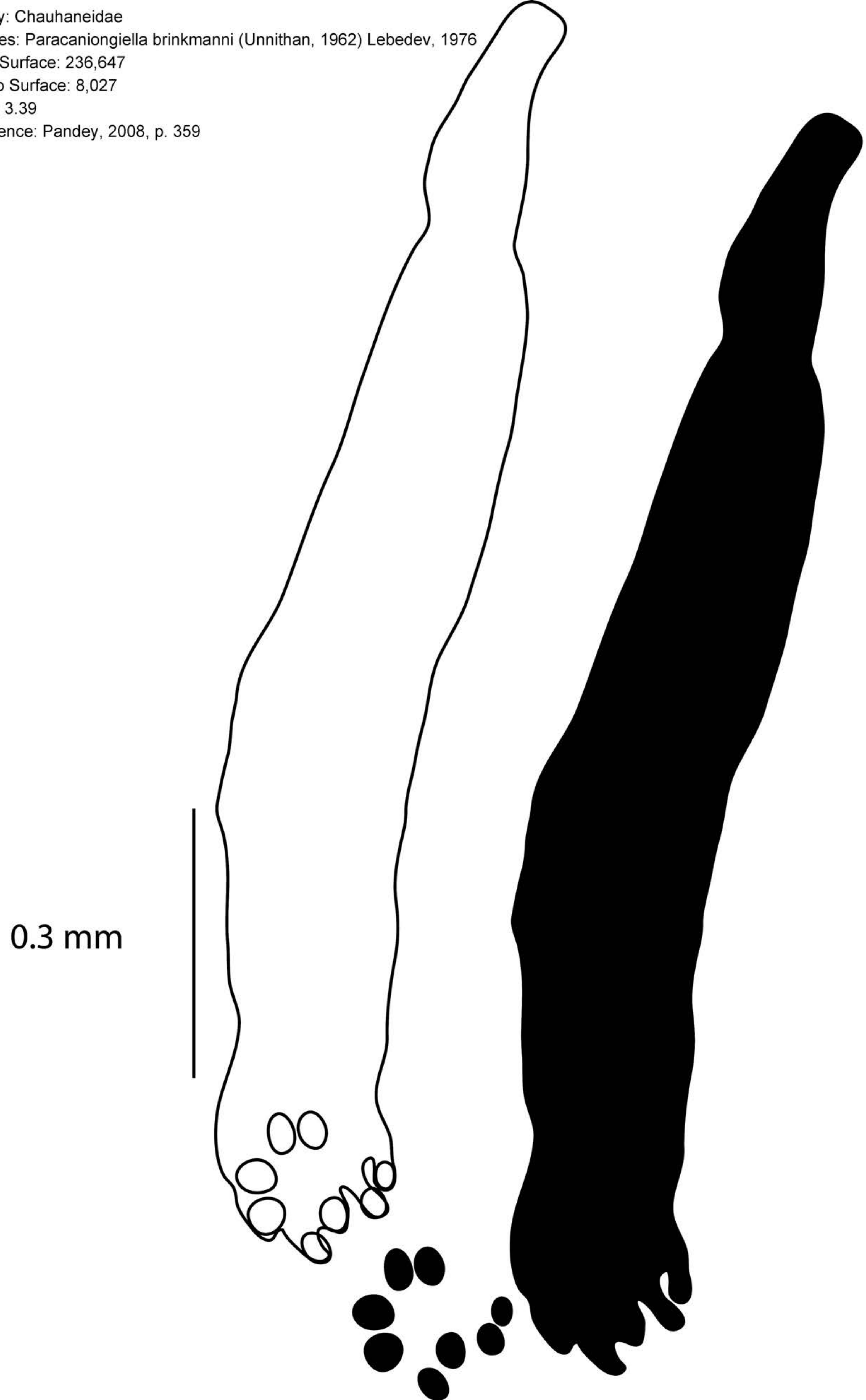

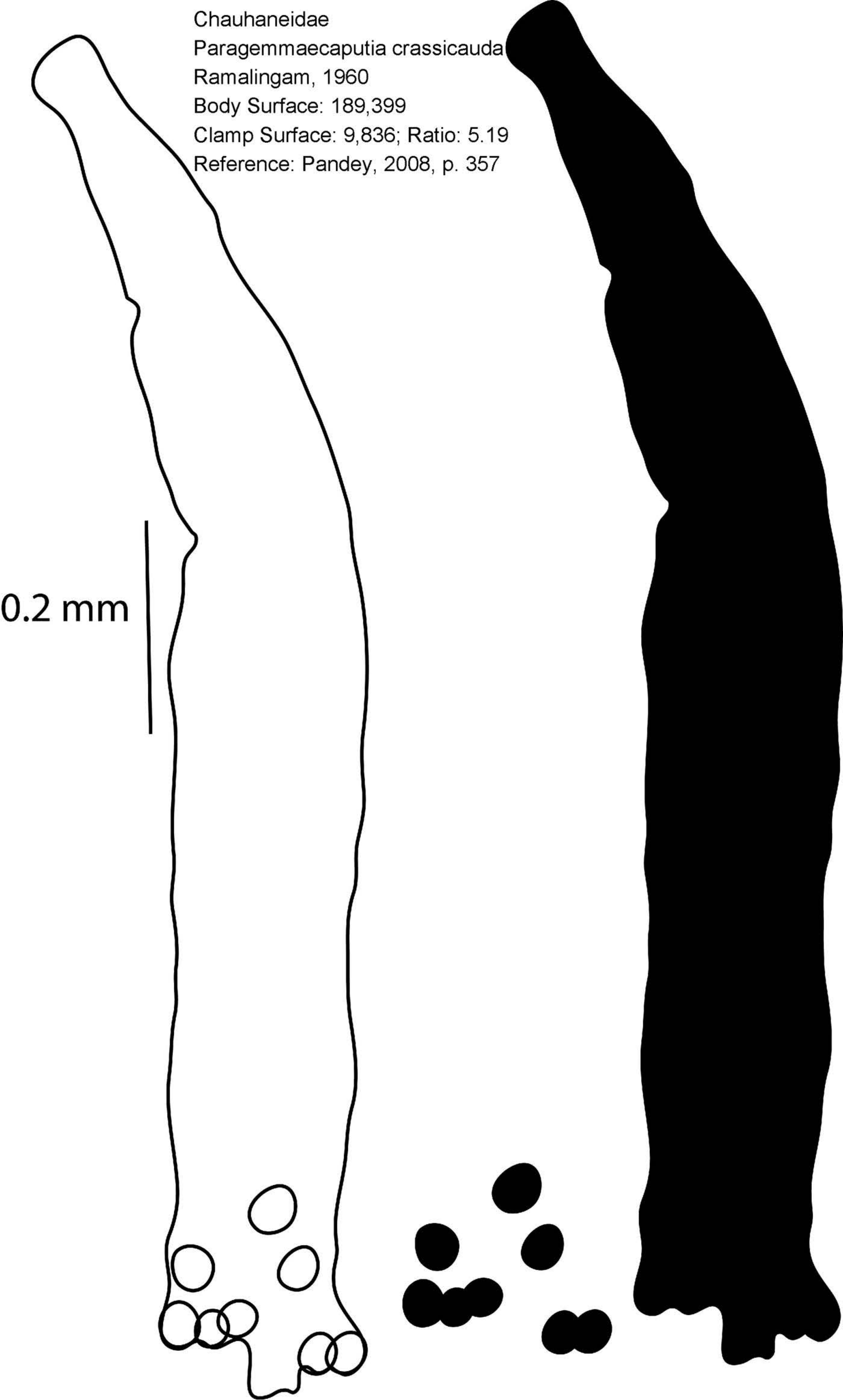
Chauhaneidae
Paragemmaecaputia crassicauda
Ramalingam, 1960
Body Surface: 189,399
Clamp Surface: 9,836; Ratio: 5.19
Reference: Pandey, 2008, p. 357
0.2 mm

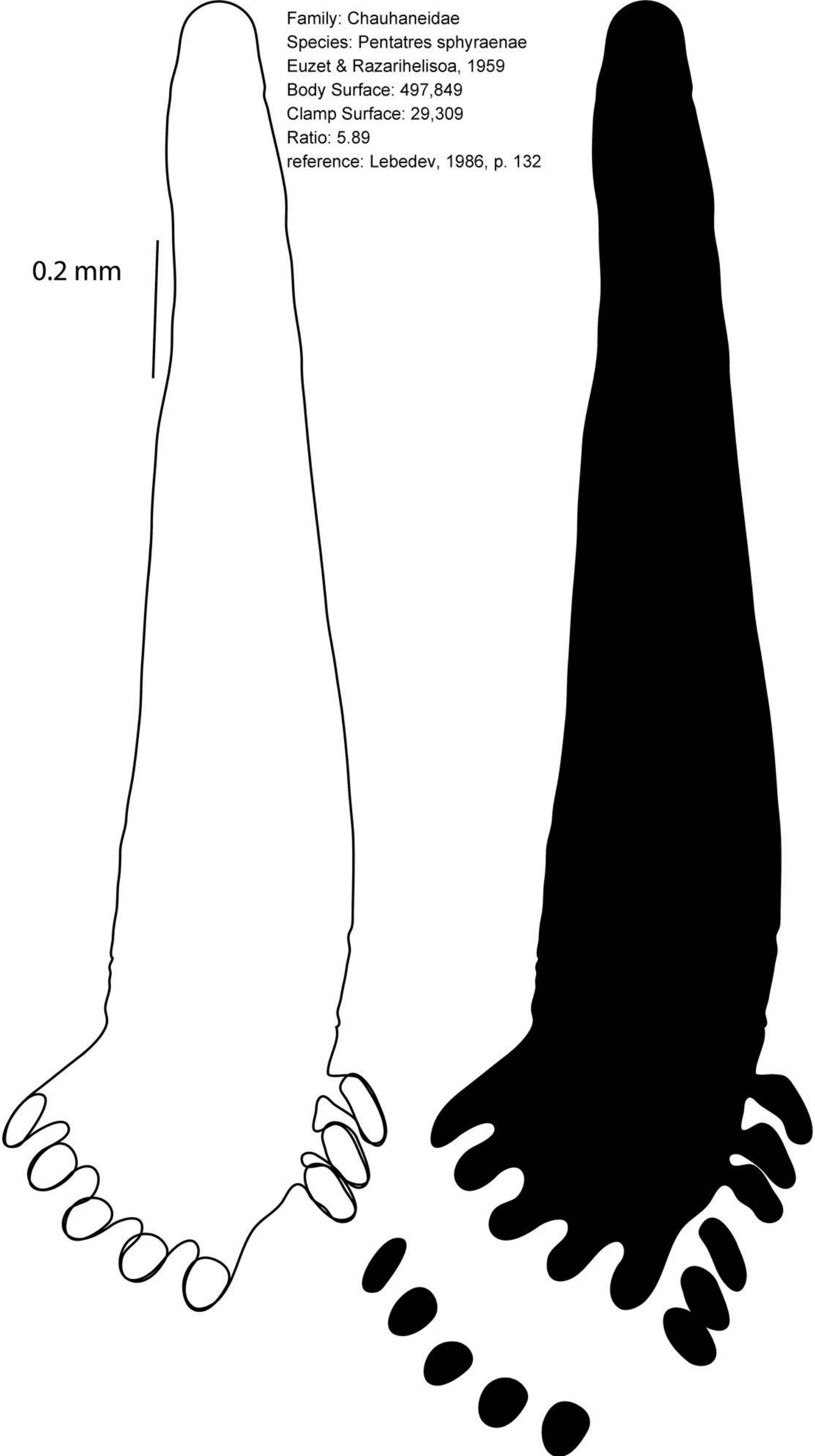
Family: Chauhaneidae
Species: Pentatres sphyraenae
Euzet & Razarihelisoa, 1959
Body Surface: 497,849
Clamp Surface: 29,309
Ratio: 5.89
reference: Lebedev, 1986, p. 132
0.2 mm

Family: Chauhaneidae
Species: Pseudochauhanea elongata Kritsky, Bilqees & Leiby, 1972
Body Surface: 593,000
Clamp Surface: 16,000
Ratio: 2.70
Reference: Lebedev, 1986, p. 122
Note: scale erroneous, should be 0.1 mm

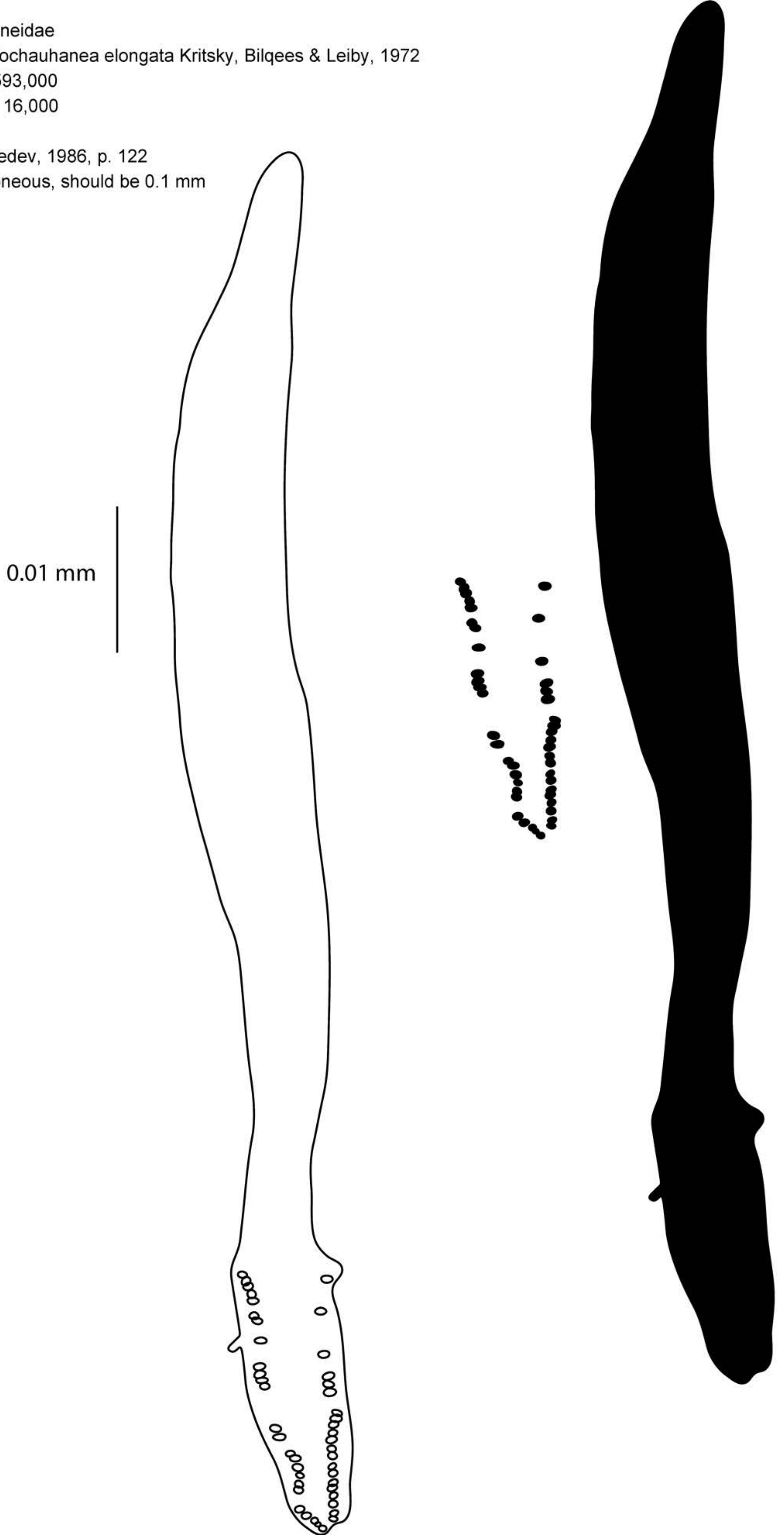

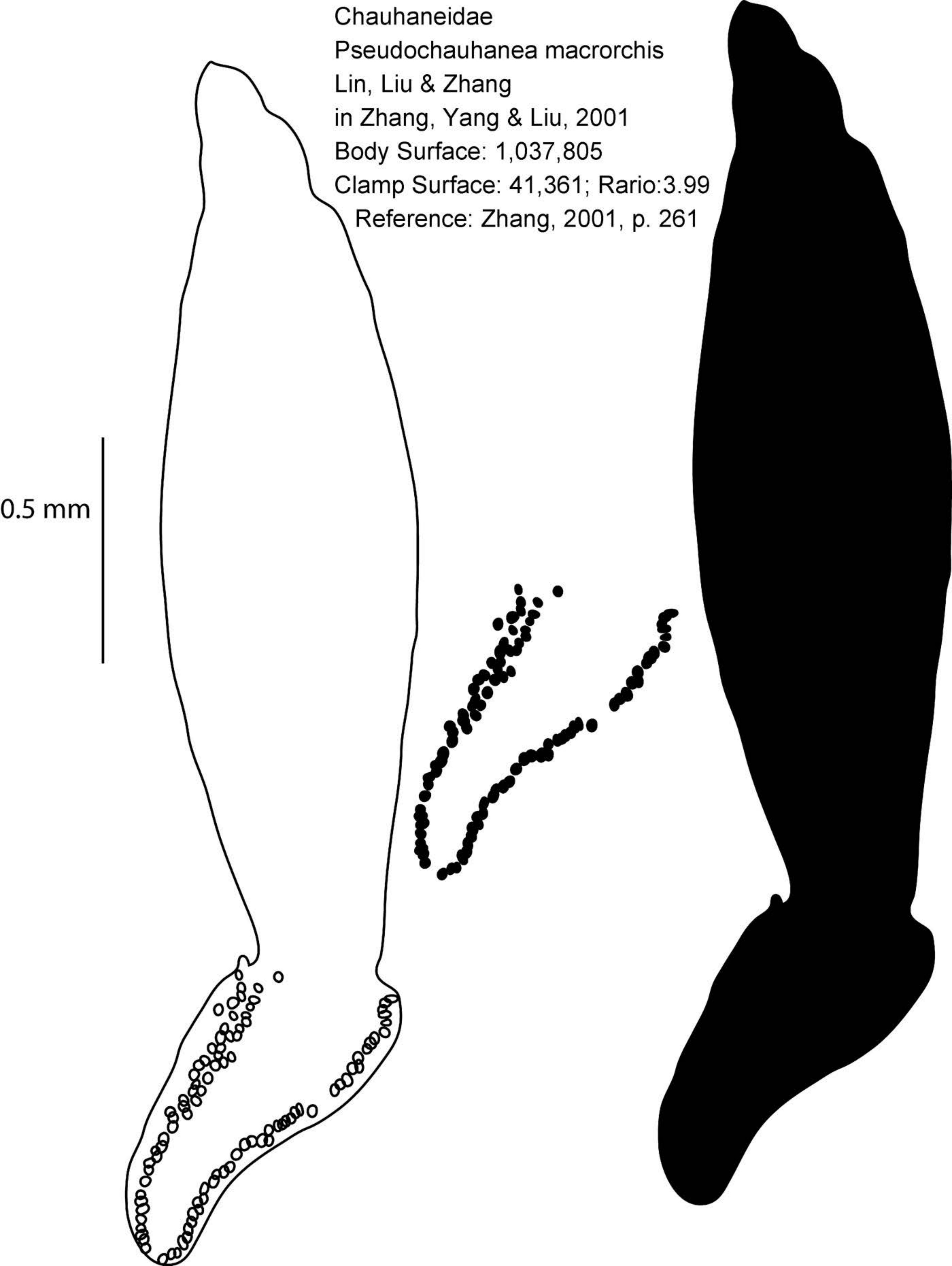
Chauhaneidae
Pseudochauhanea macrorchis
Lin, Liu & Zhang
in Zhang, Yang & Liu, 2001
Body Surface: 1,037,805
Clamp Surface: 41,361; Rario:3.99
Reference: Zhang, 2001, p. 261
0.5 mm

Family: Chauhaneidae
Species: Pseudochauhanea mexicana Lamothe, 1967
Body Surface: 2,354,237
Clamp Surface: 123,241
Ratio: 5.23
Reference: Lebedev, 1986, p. 120

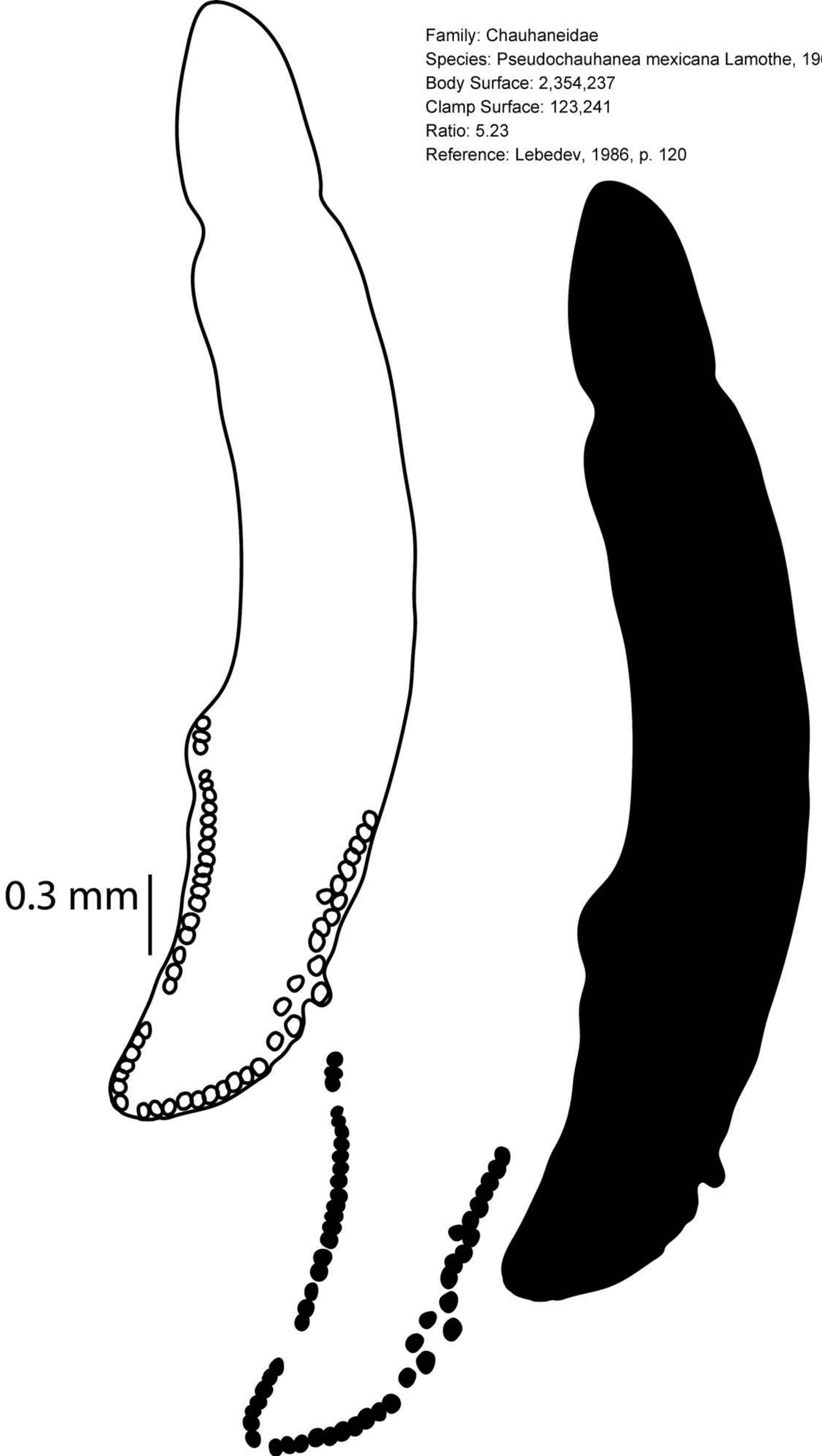

Chauhaneidae

Pseudochauhanea sphyraenae Yamaguti, 1965

Body Surface: 2,795,654

Clamp Surface: 110,955

Ratio: 3.97

Reference: Yamaguti, 1968, p. 251

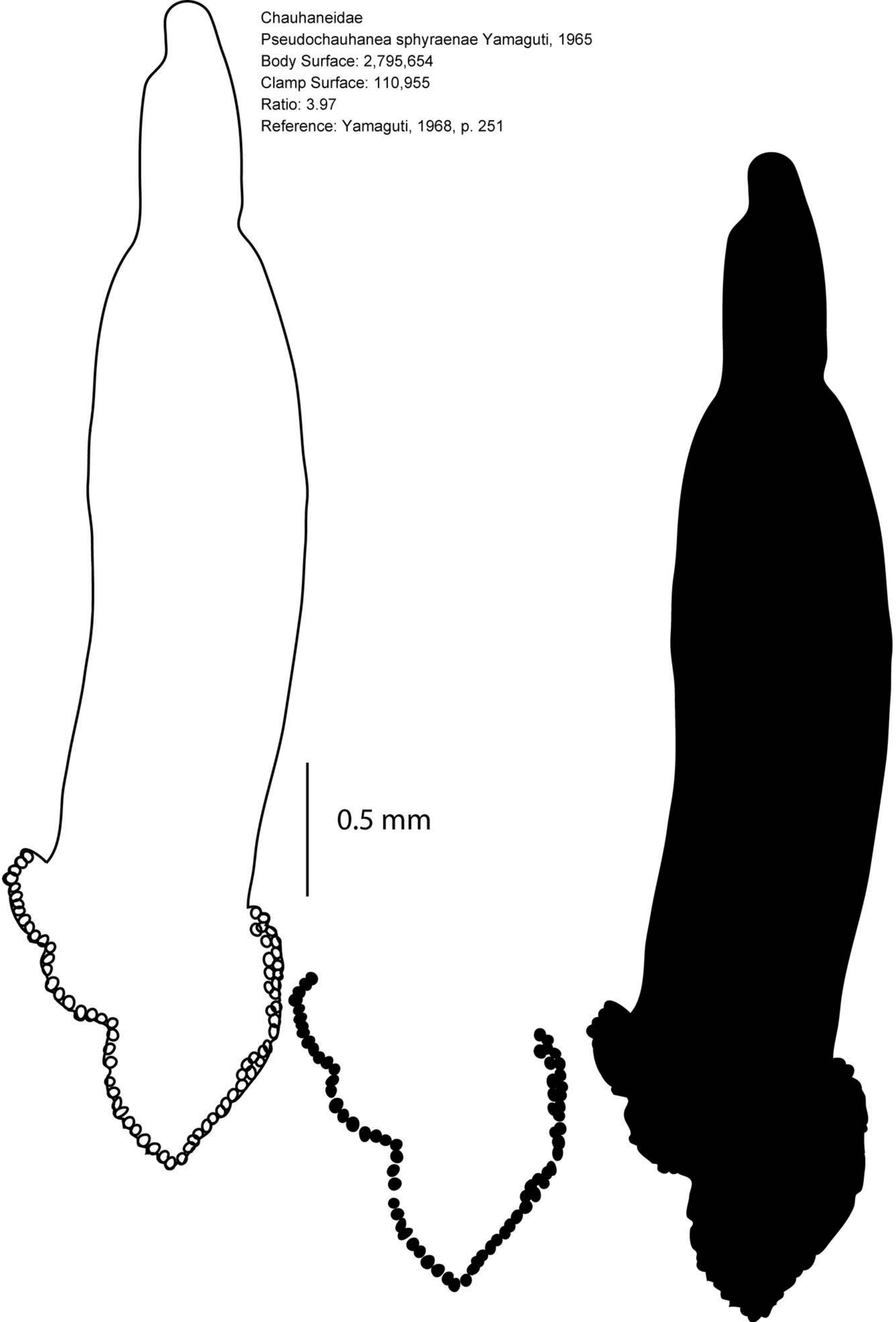

Chauhaneidae

Pseudomazocraes monsivaisae Caballero & Bravo Hollis, 1955

Body Surface: 811

Clamp Surface: 38

Ratio: 4.69

Reference: Caballero & Caballero, 1955, p. 108

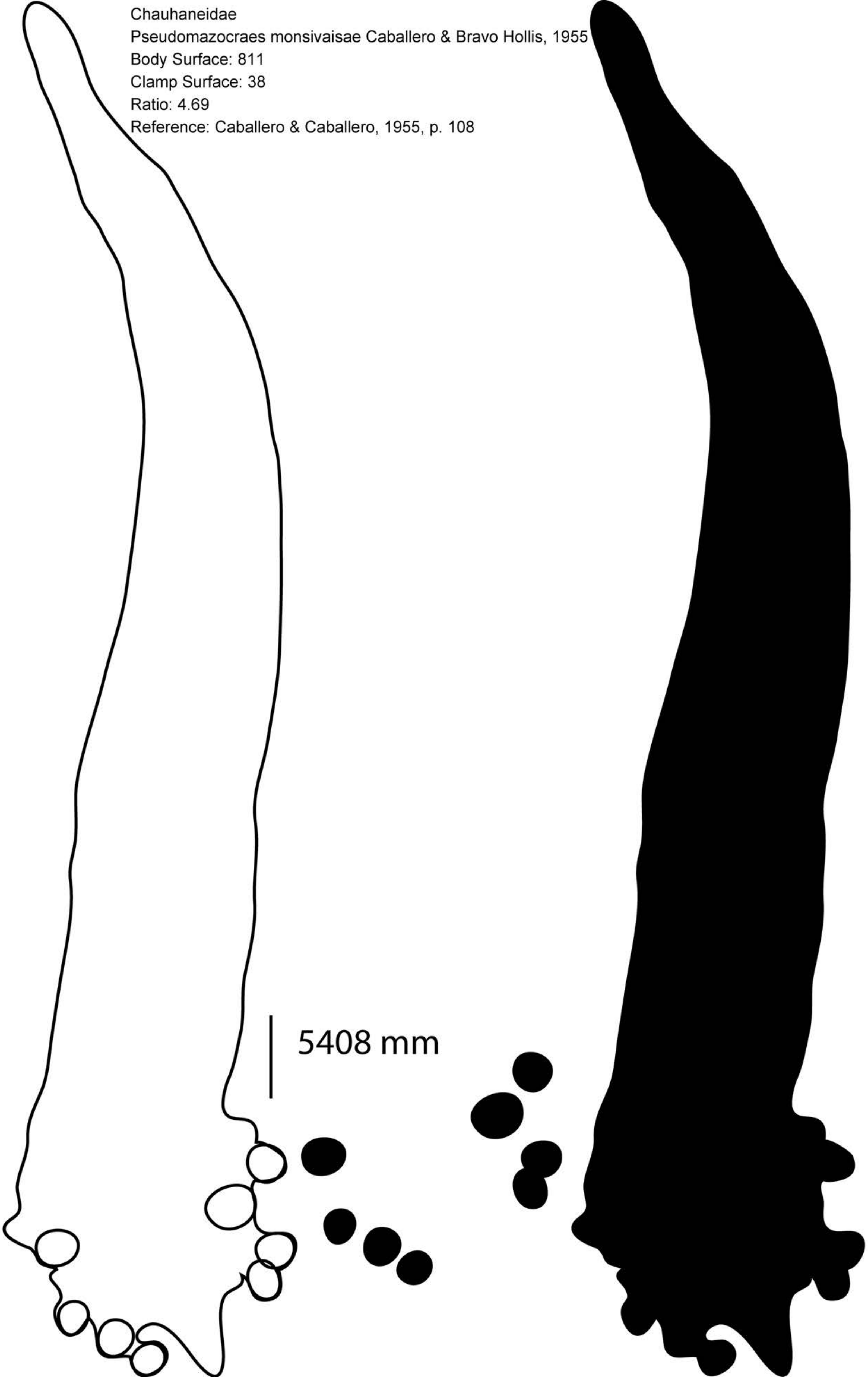

Family: Chauhaneidae
Species: Pseudomazocraes selene Hargis, 1957
Body Surface: 71,077
Clamp Surface: 49,995
Ratio: 6.48
Reference: Hargis, 1957, p. 7

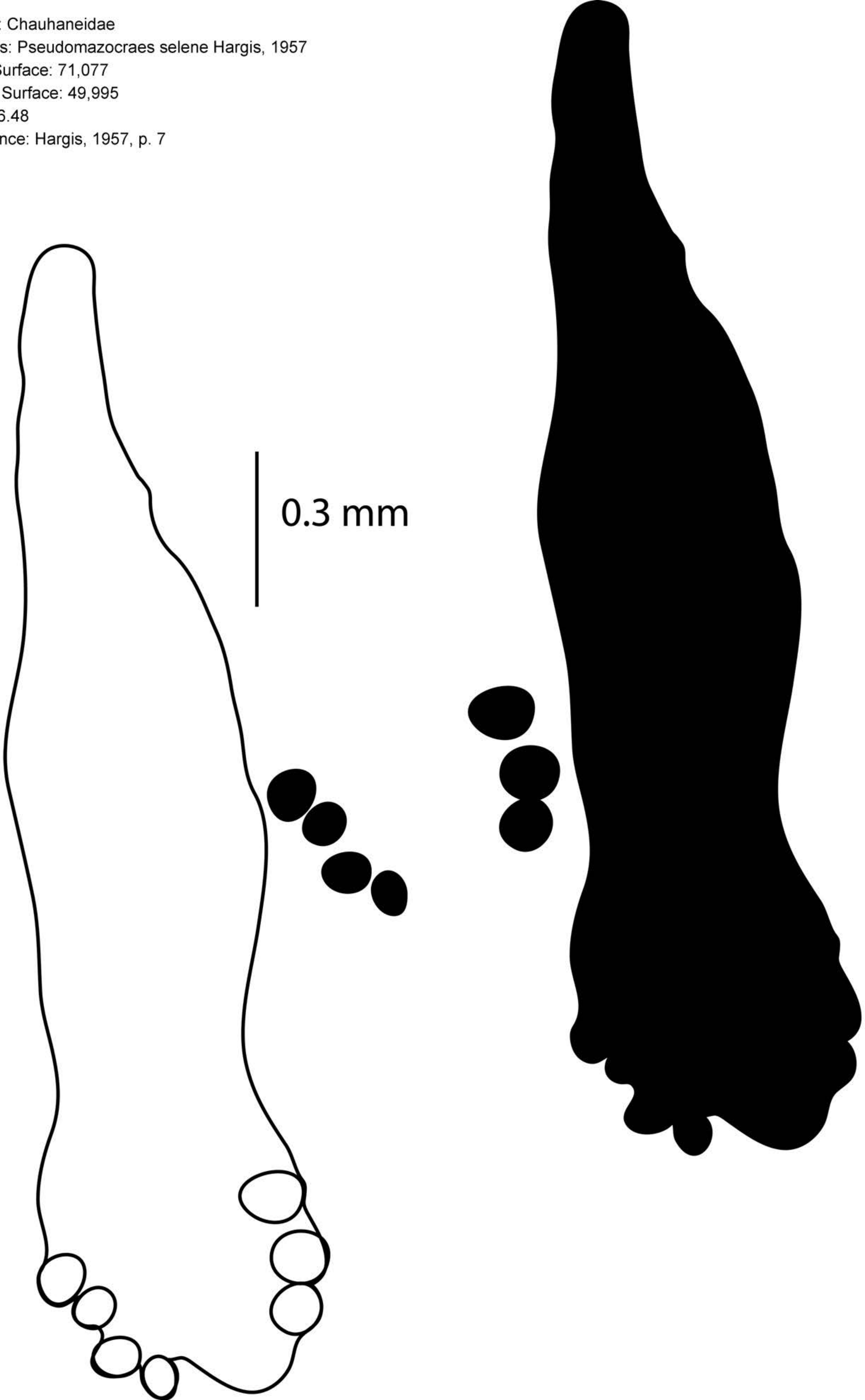

Family: Chauhaneidae
Species: Pseudopisthogynopsis lepidocybii Yamaguti, 1965
Body Surface: 8,160,745
Clamp Surface: 821,312
Ratio: 10.06
Reference: Lebedev, 1986, p. 117

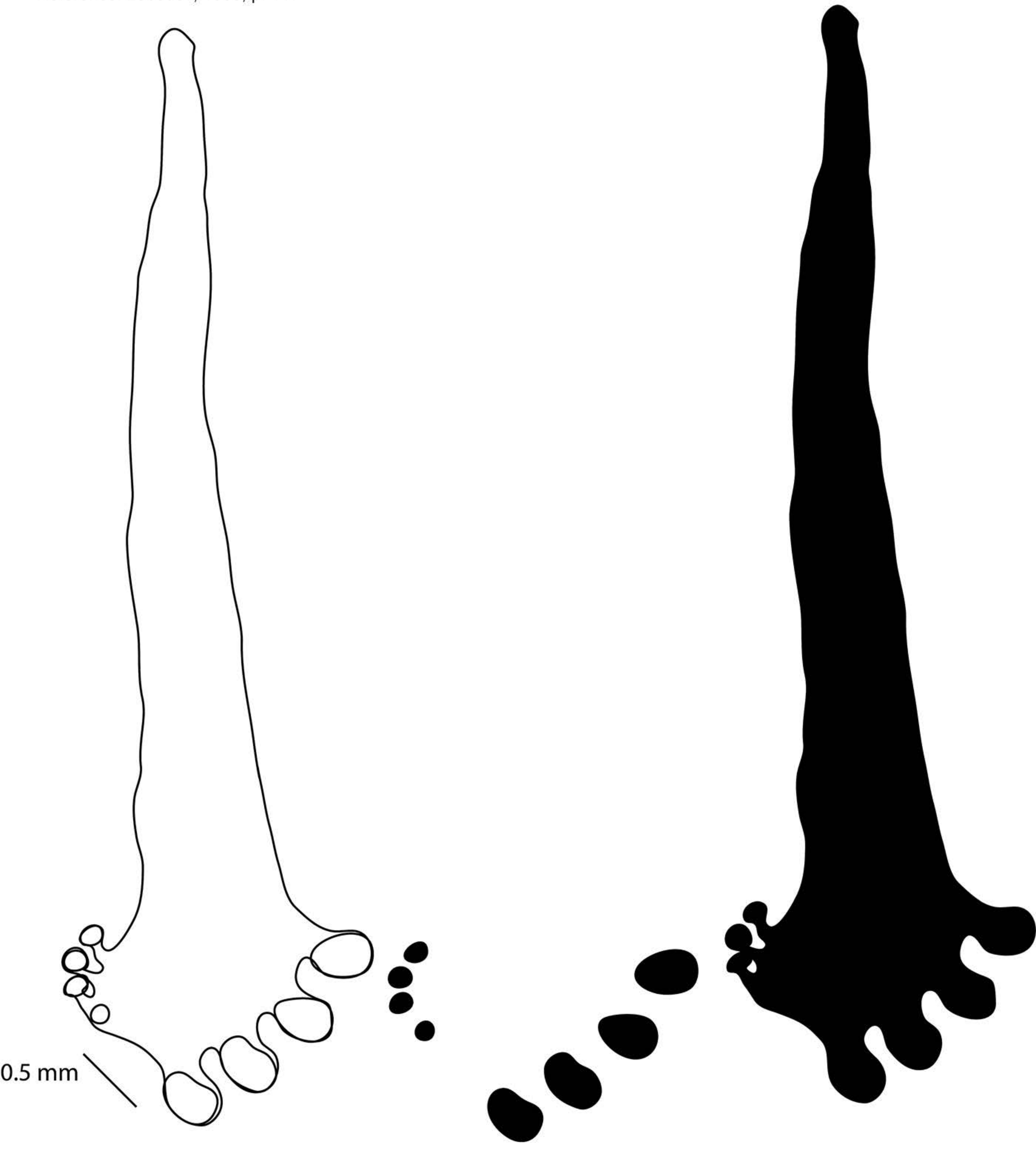

Family: Chauhaneidae
Species: Pseudopisthogyne lepidocybii
Yamaguti, 1965
Body Surface: 2,106,993
clamp Surface: 189,587
Ratio: 9.00
Reference: Yamaguti, 1965, p. 75

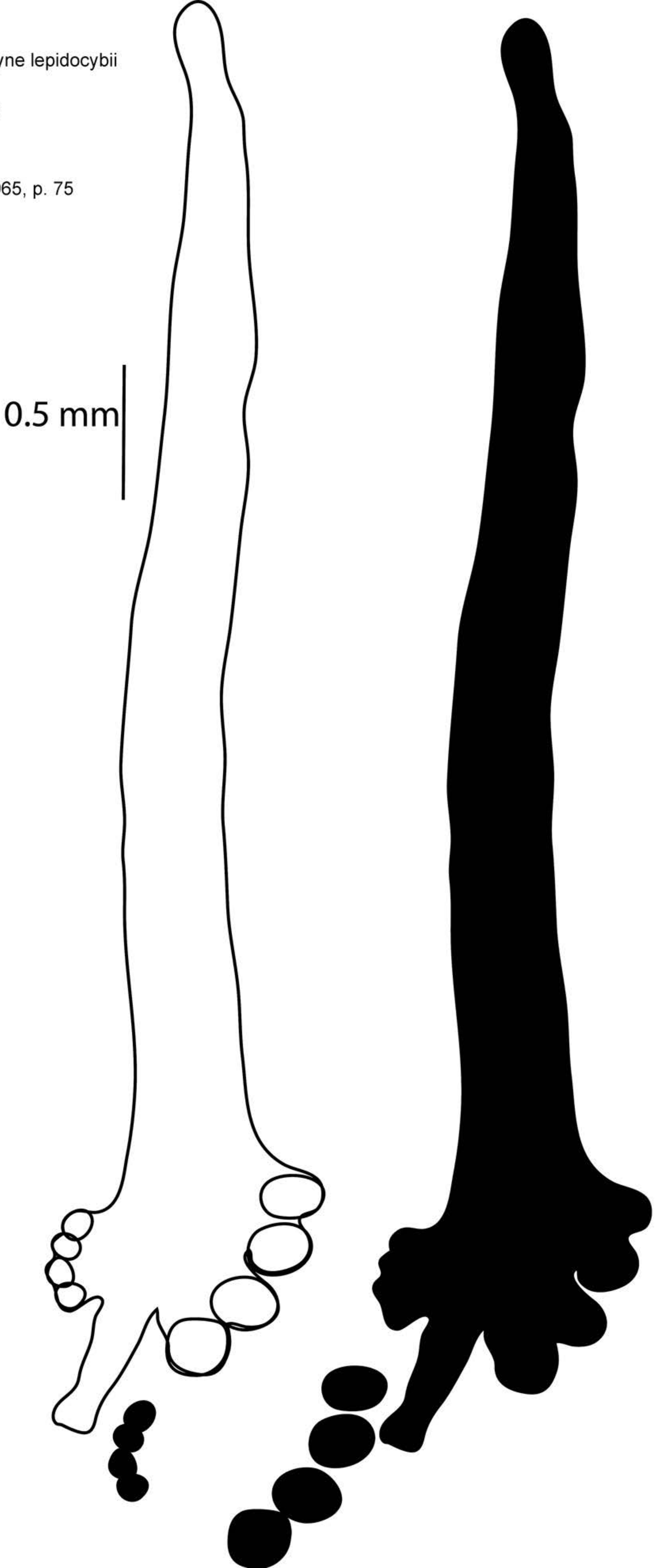

Family: Chauhaneidae
Species: Salinacotyle mexicana (Caballero & Bravo-Hollis, 1963) Lebedev, 1984
Body Surface: 1,425,544
Clamp Surface: 103,400
Ratio: 7.25
Reference: Lebedev, 1986, p. 138

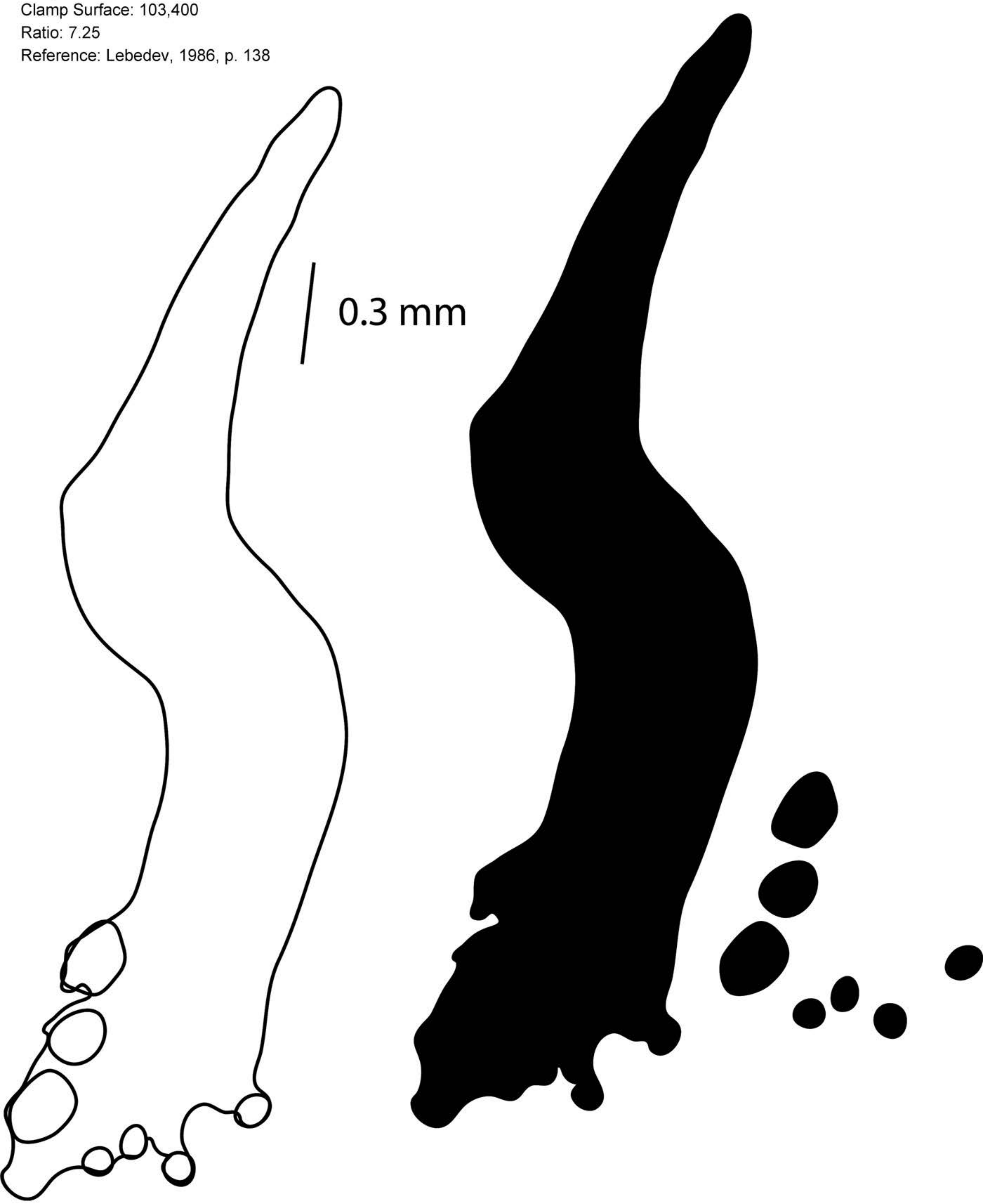

Family: Gastrocotylidae
Species: Allopseudaxine macrova (Unnithan, 1957) Yamaguti, 1963
Body Surface: 4,858,980
Clamp Surface: 388,611
Ratio: 8.00
Reference: Yamaguti, 1963, p. 265

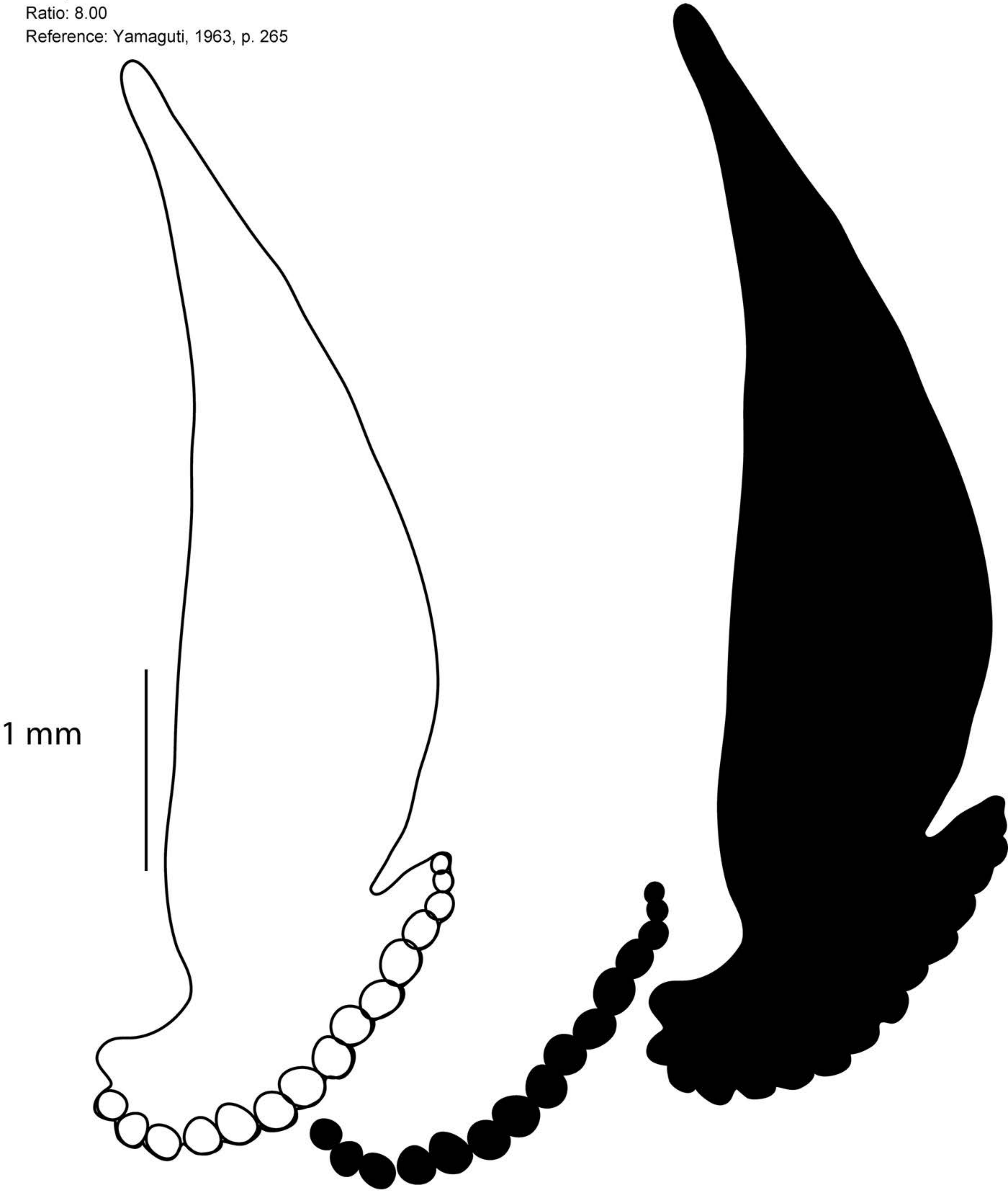

Family: Gastrocotylidae
Species: Allopseudaxine yaito Yamaguti, 1968
Body Surface: 14,764,474
Clamp Surface: 485,975
Ratio: 3.29
Reference: Yamaguti, 1968, p. 251

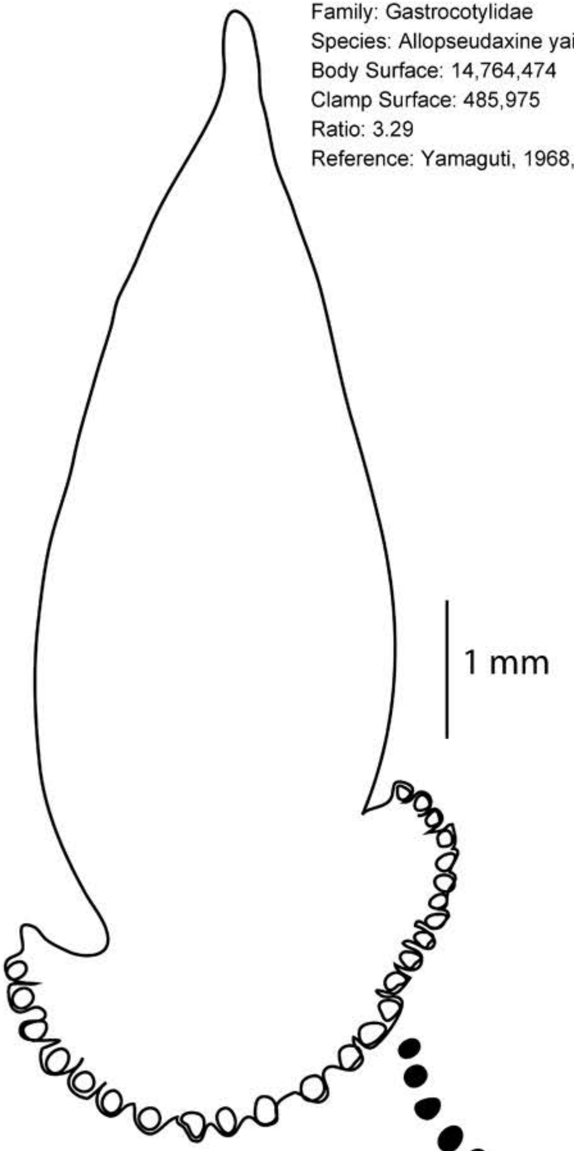

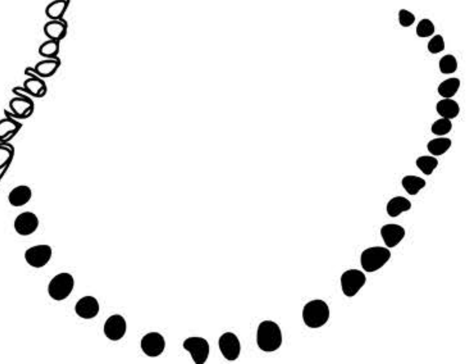

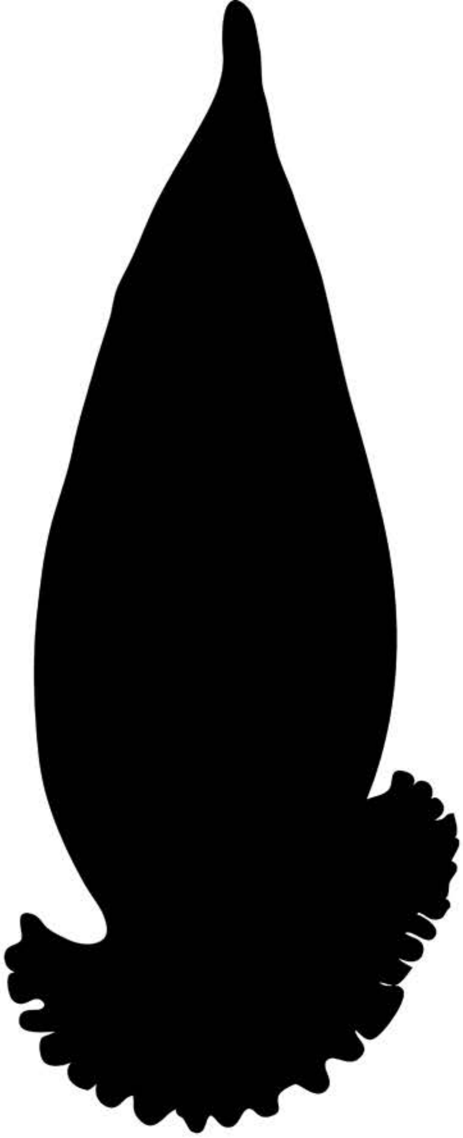

Family: Gastrocotylidae
Species: Allopseudaxinoides euthynni Yamaguti, 1965
Body Surface: 11,587,179
Clamp Surface: 720,066
Ratio: 6.21
Reference: Yamaguti, 1965, p. 84

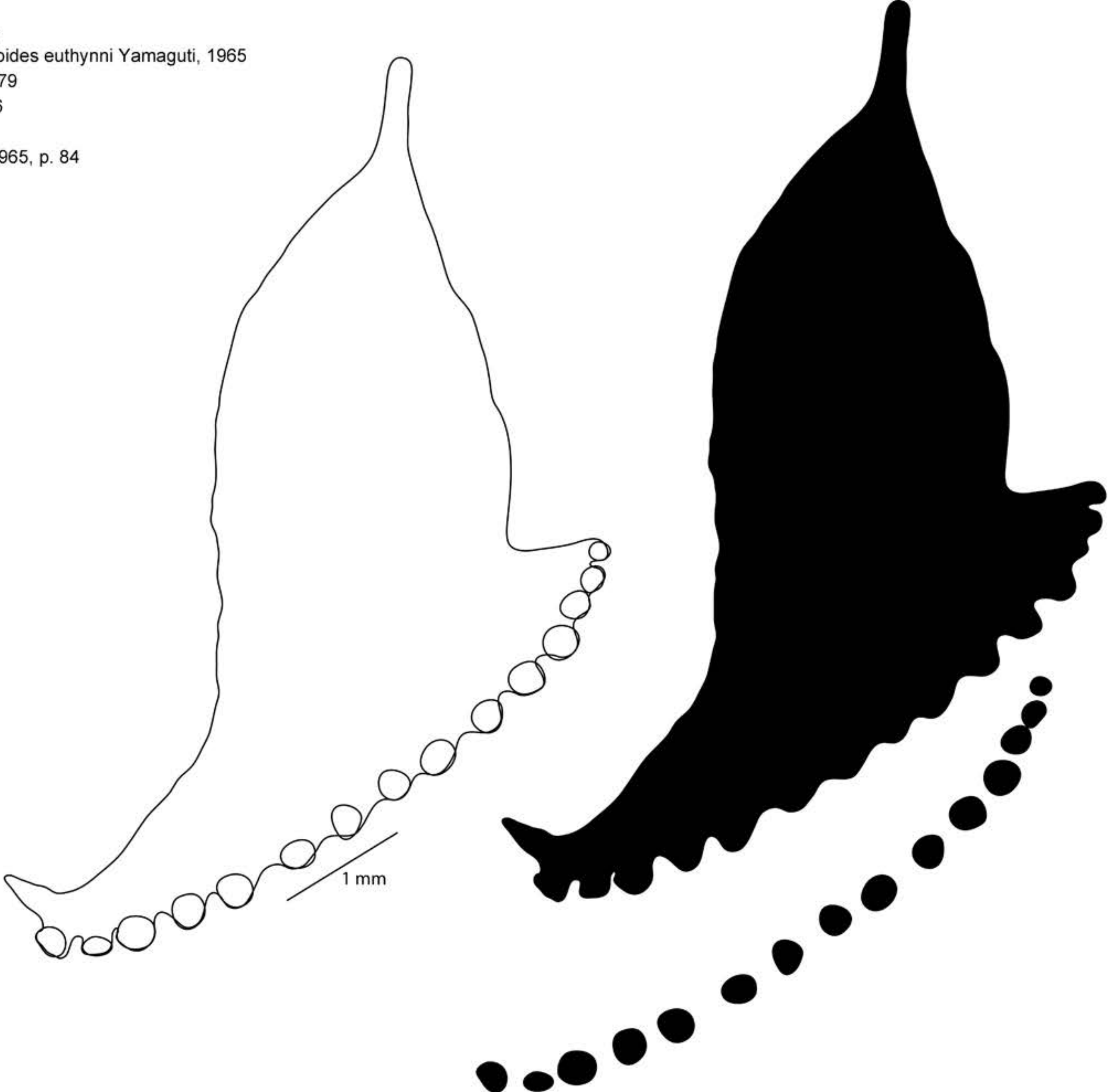

Family: Gastrocotylidae
Species: Amphipolycotyle chloroscombrus Hargis, 1957
Body Surface: 209,204
Clamp Surface: 22,656
Ratio: 10.83
Reference: Hargis, 1957, p. 5

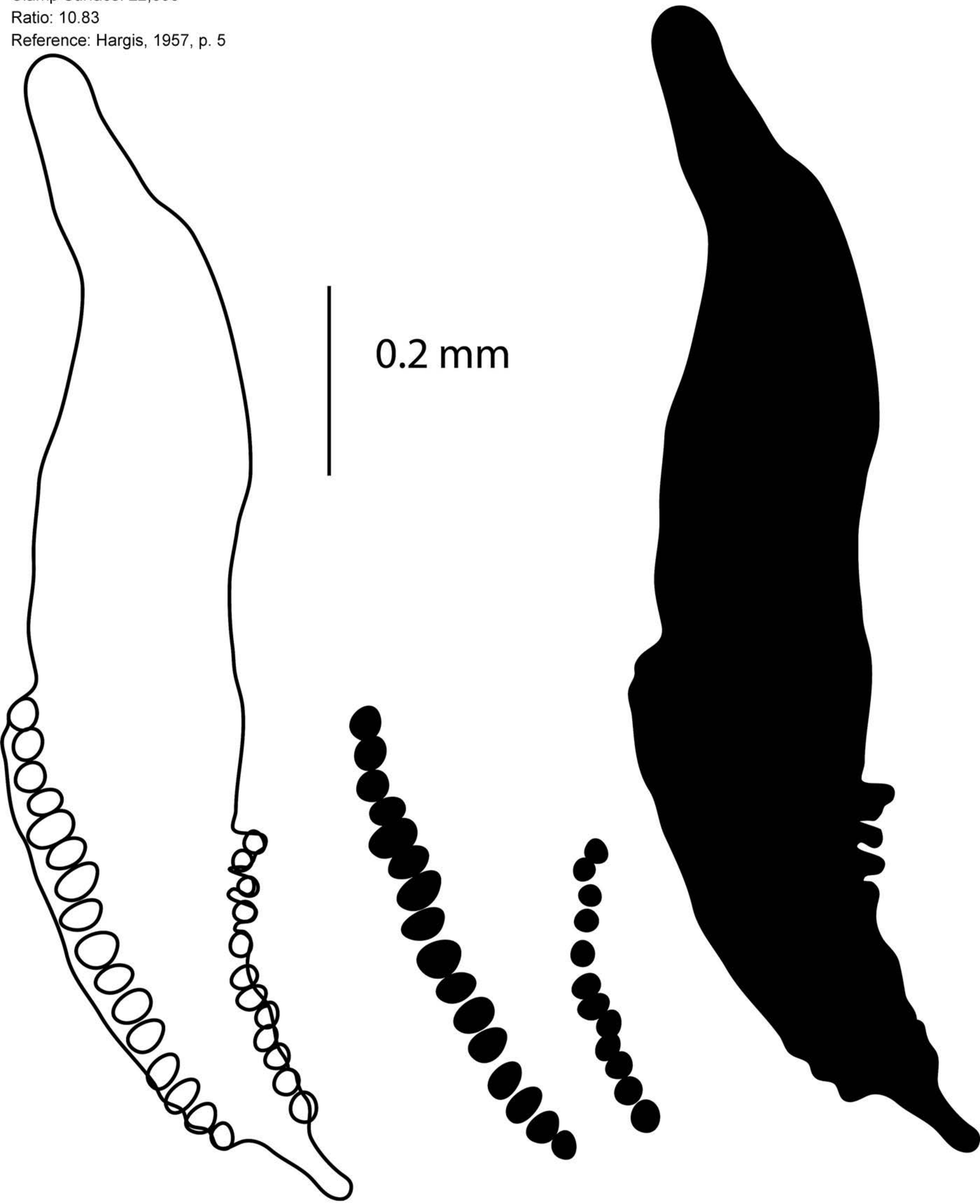

Family: Gastrocotylidae
Species: Areotestis sibi Yamaguti, 1965
Body Surface: 33,887,590
Clamp Surface: 629,933
Ratio: 1.86
Reference: Yamaguti, 1965, p. 79

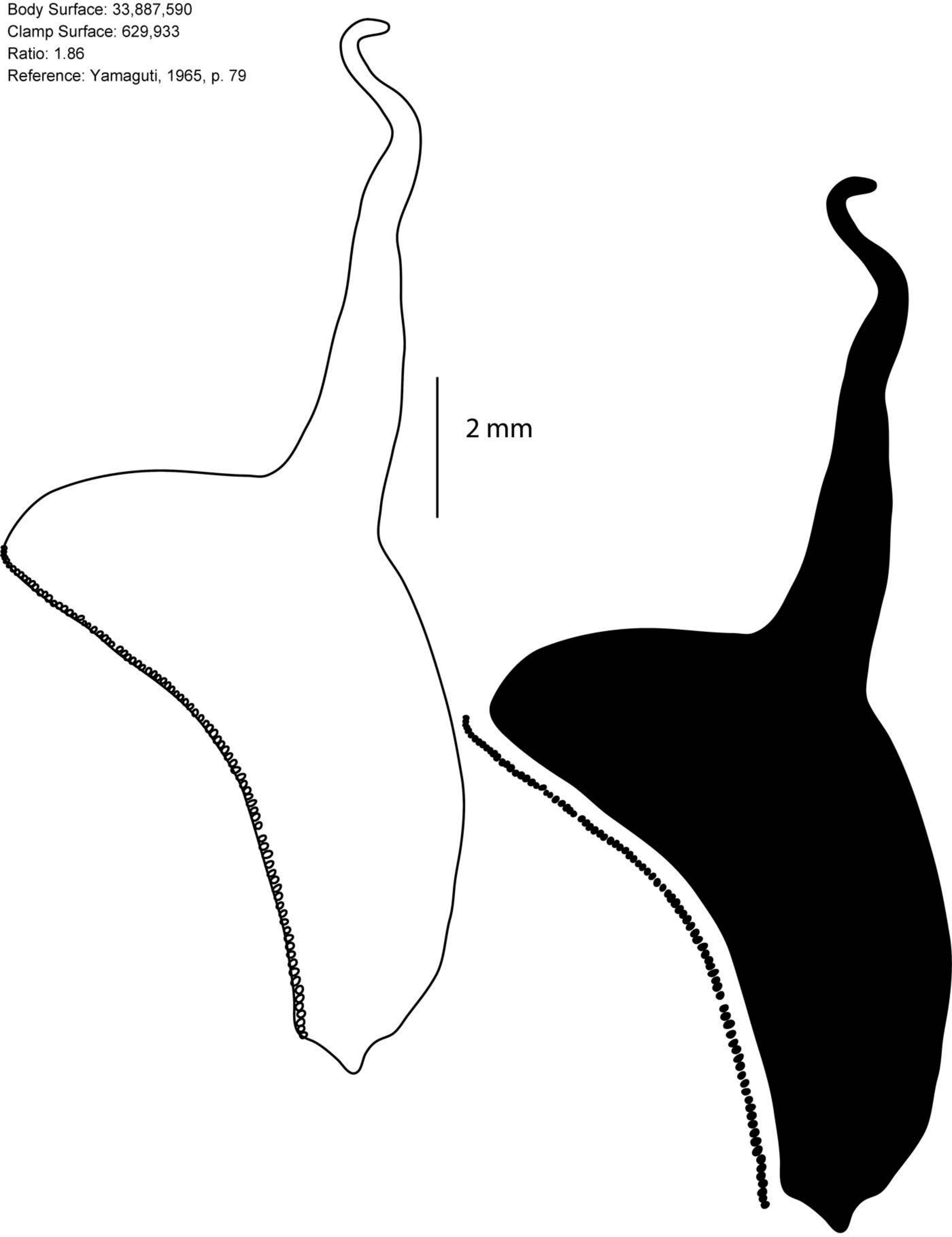

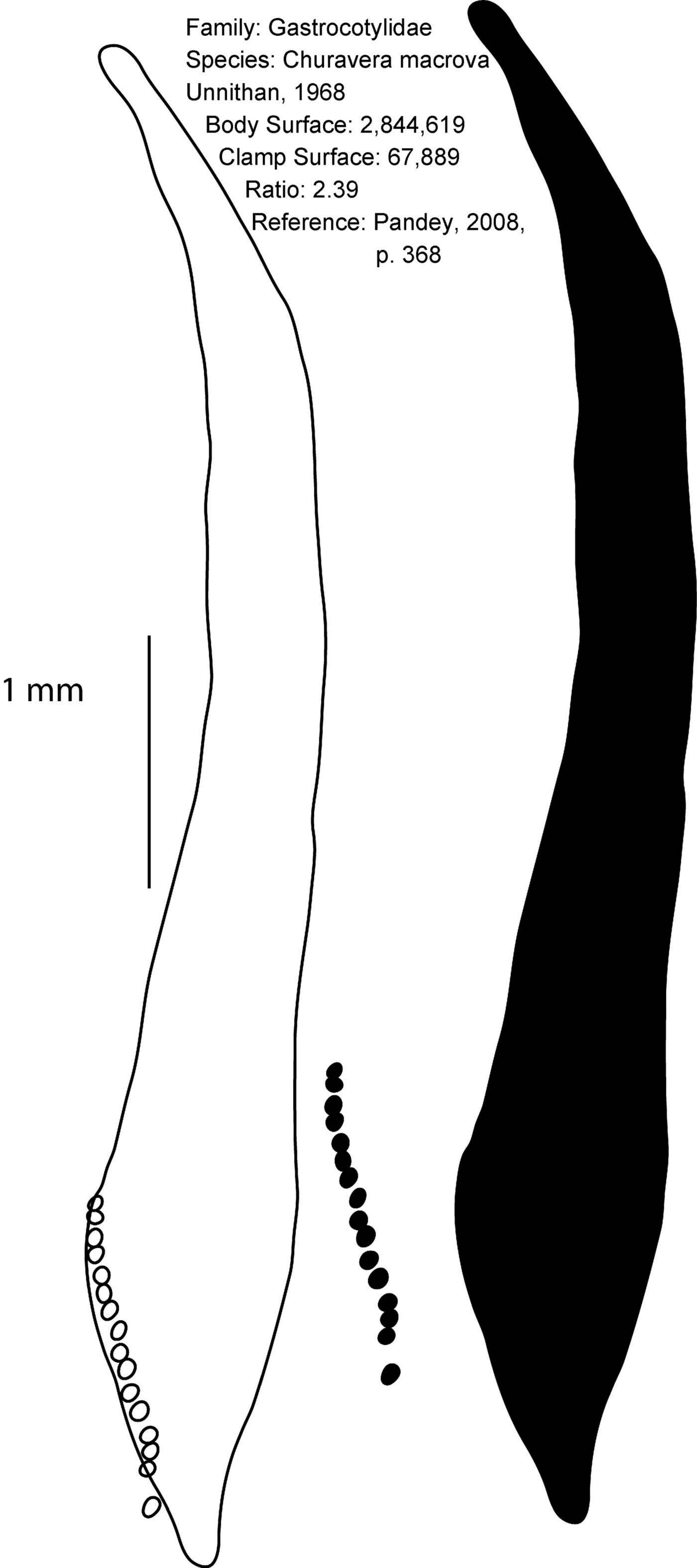
Family: Gastrocotylidae
Species: Churavera macrova
Unnithan, 1968
Body Surface: 2,844,619
Clamp Surface: 67,889
Ratio: 2.39
Reference: Pandey, 2008,
p. 368
1 mm

Family: Gastrocotylidae
Species: *Cypselurobranchitrema spilonotopteri* Yamaguti, 1966
Body Surface: 204,576
Clamp Surface: 13,906
Ratio: 6.80
Reference: Yamaguti, 1966, p. 432

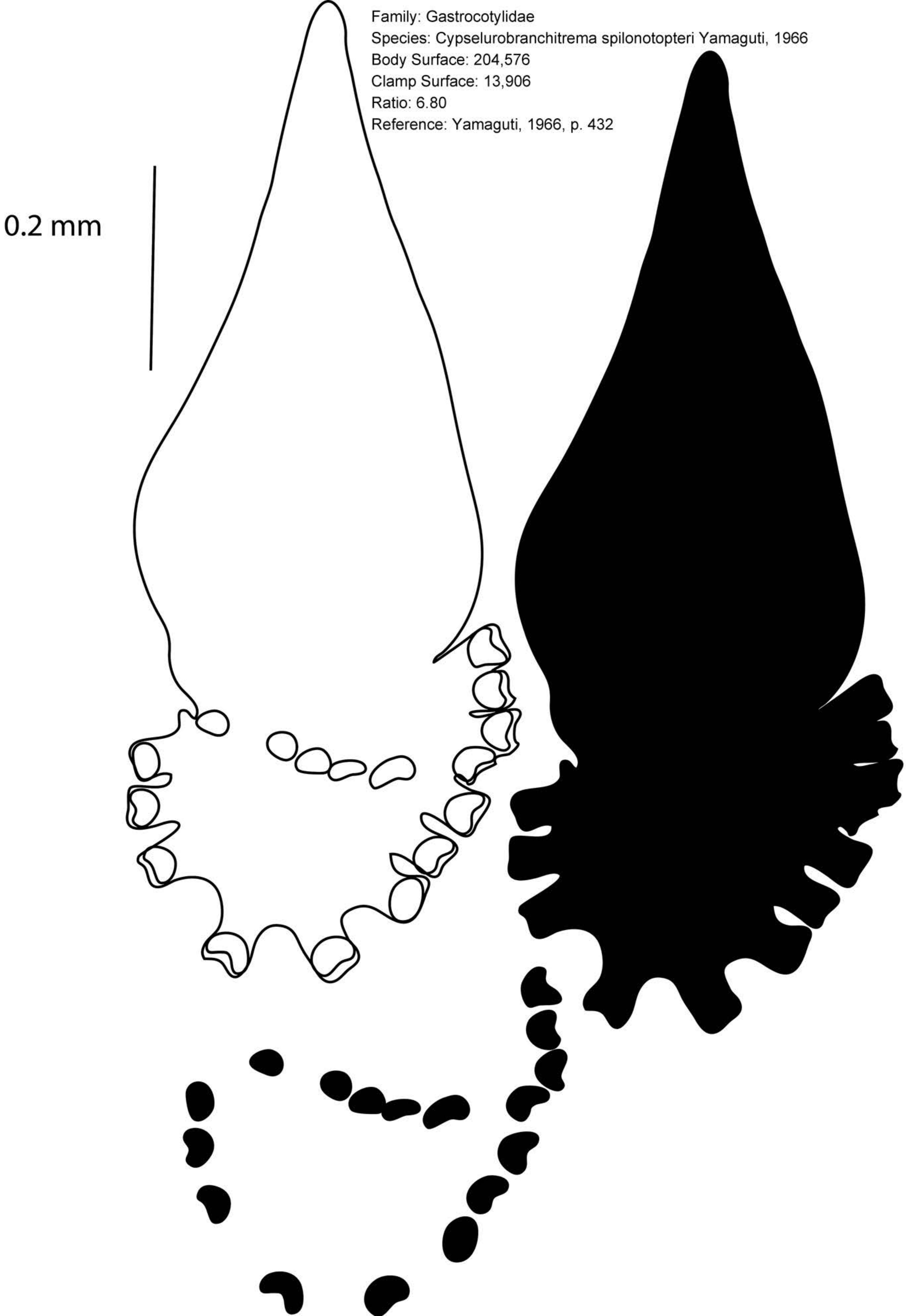

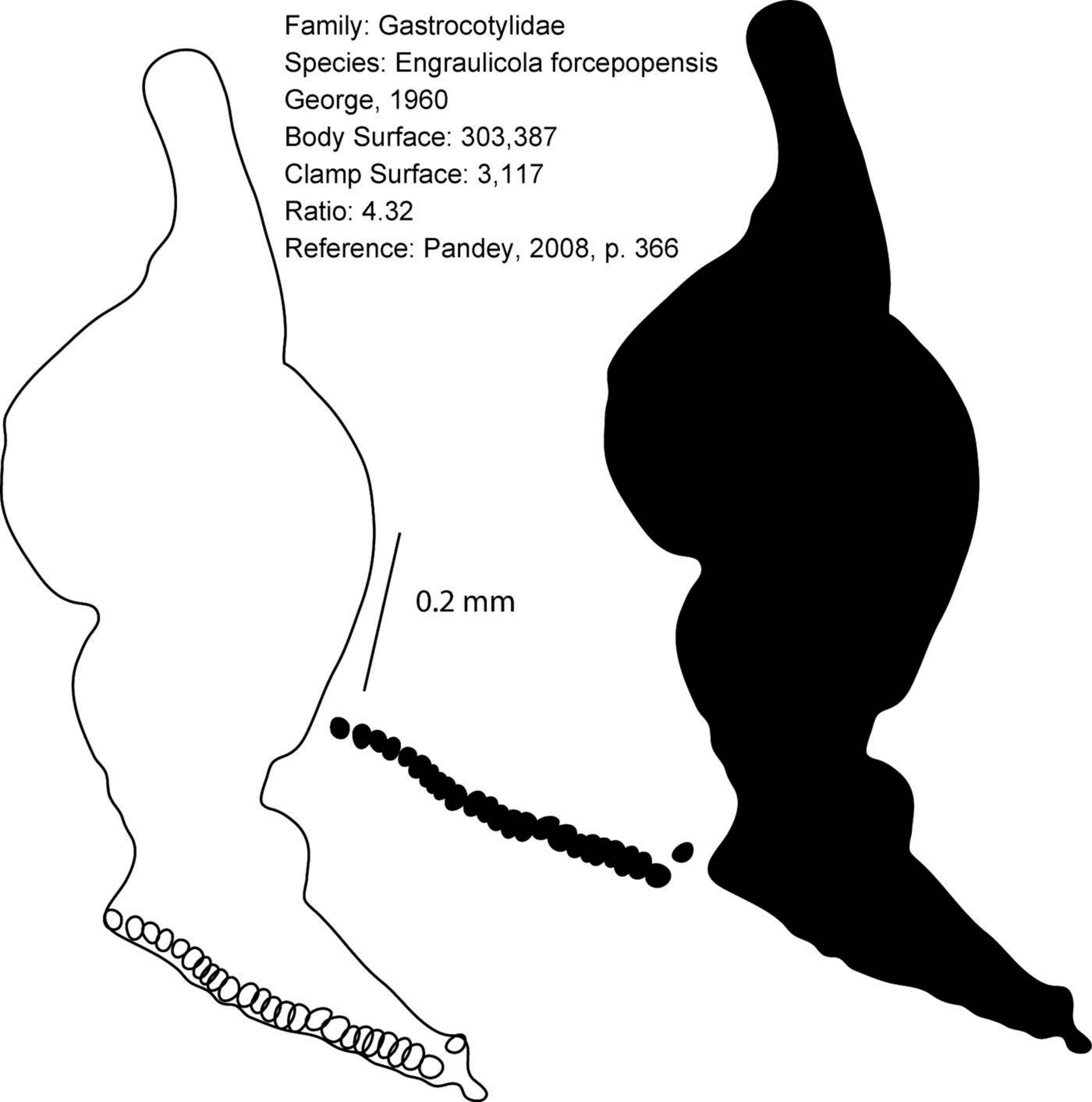
Family: Gastrocotylidae
Species: Engraulicola forcepopensis
George, 1960
Body Surface: 303,387
Clamp Surface: 3,117
Ratio: 4.32
Reference: Pandey, 2008, p. 366
0.2 mm

Family: Gastrocotylidae
Species: Engraulicola micropharyngella Unnithan, 1967
Body Surface: 293,283
Clamp Surface: 12,295
Ratio: 4.19
Reference: Unnithan, 1967, p. 212

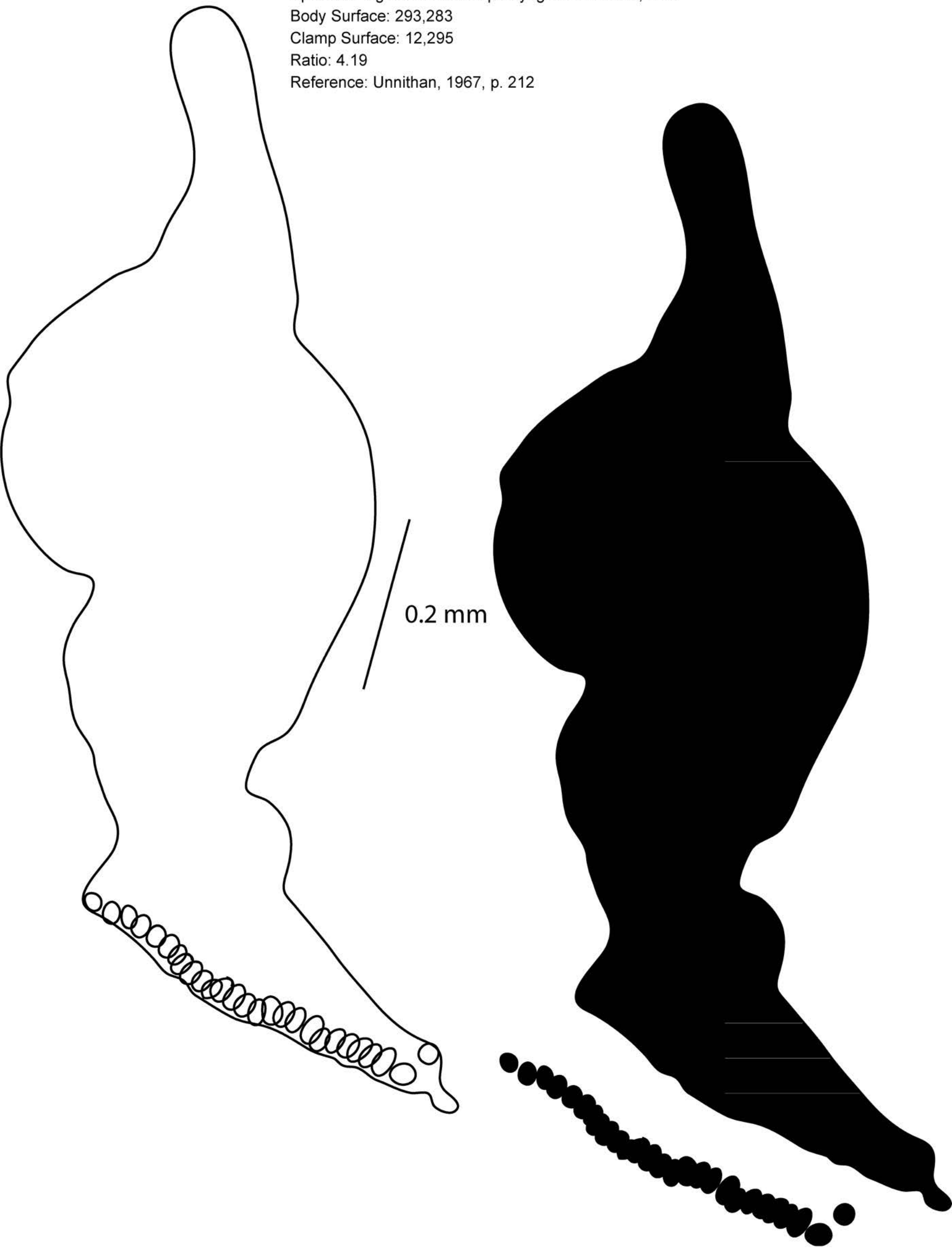

Family: Gastrocotylidae
Species: Engraulicola thrissocles (Tripathi, 1959) Lebedev, 1971
Body Surface: 1,014,407
Clamp Surface: 69,075
Ratio: 6.81
Reference: Lebedev, 1986, p. 70

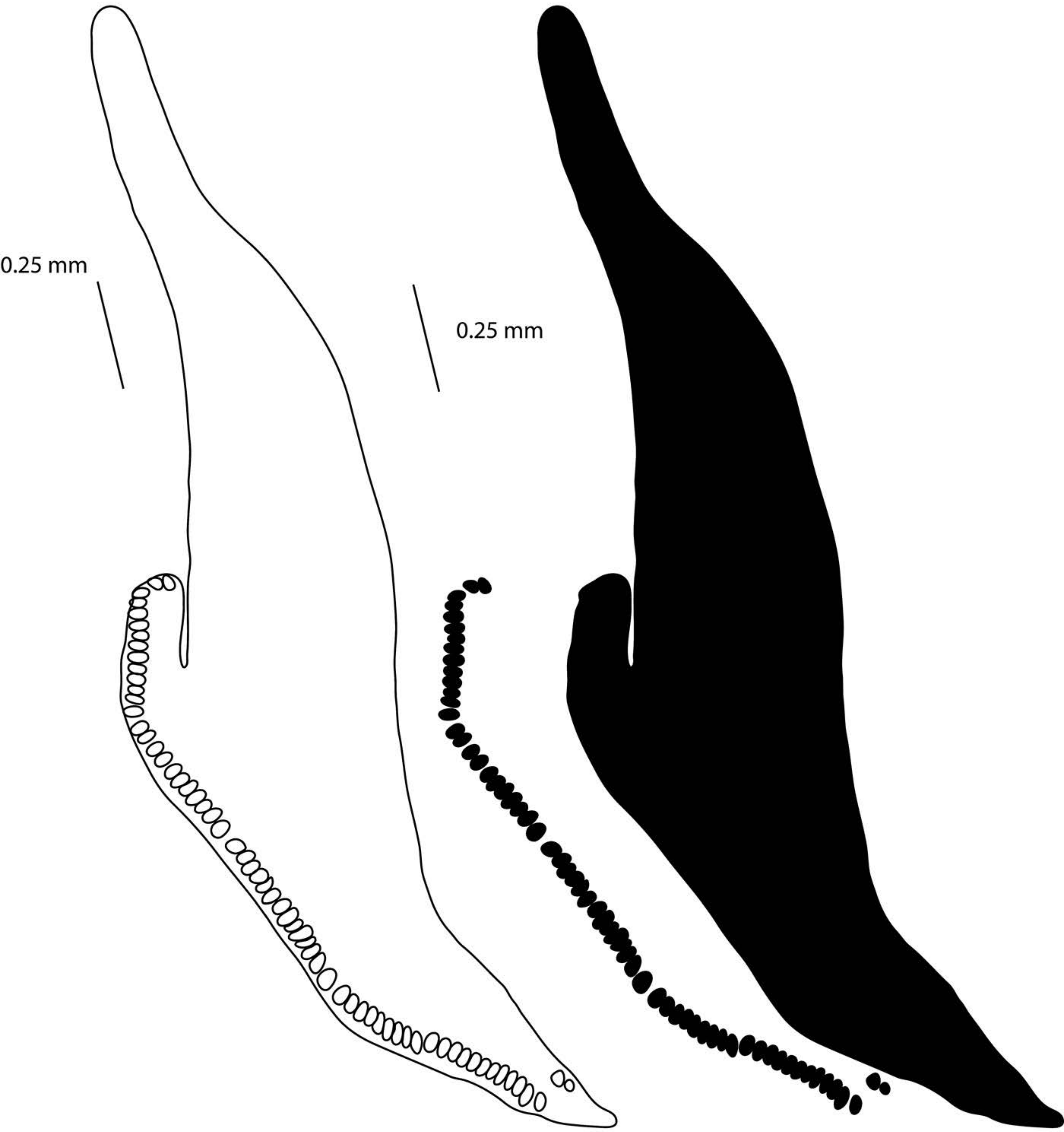

Family: Gastrocotylidae
species: Engrauliphila grex Unnithan, 1967
Body Surface: 208,190
Clamp Surface: 24,488
Ratio: 11.76
Reference: Unnithan, 1967, p. 218

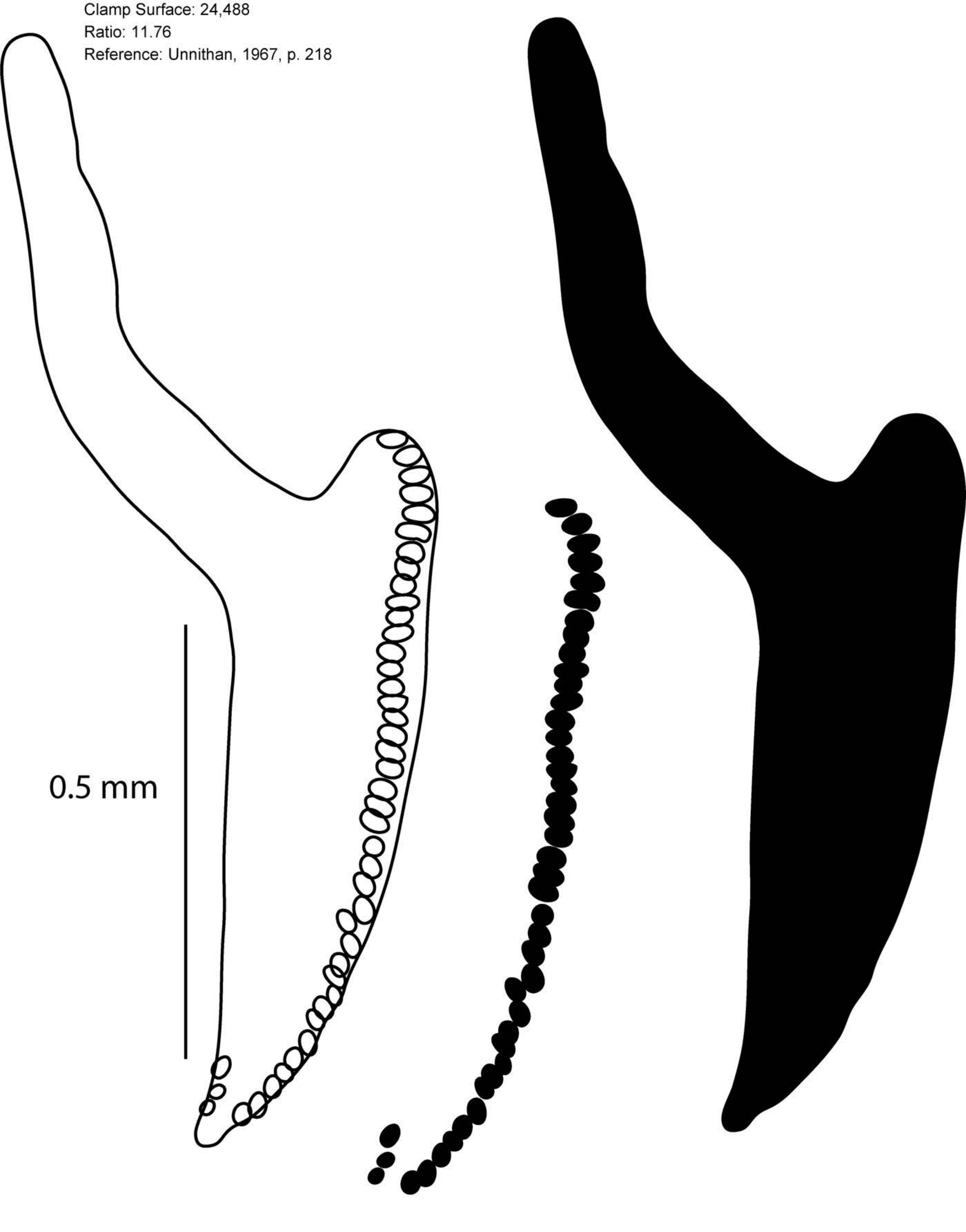

Family: Gastrocotylidae
Species: Engrauliscobina triaptella Unnithan, 1967
Body Surface: 1,465,577
Clamp Surface: 91,211
Ratio: 6.22
Reference: Unnithan, 1967, p. 221

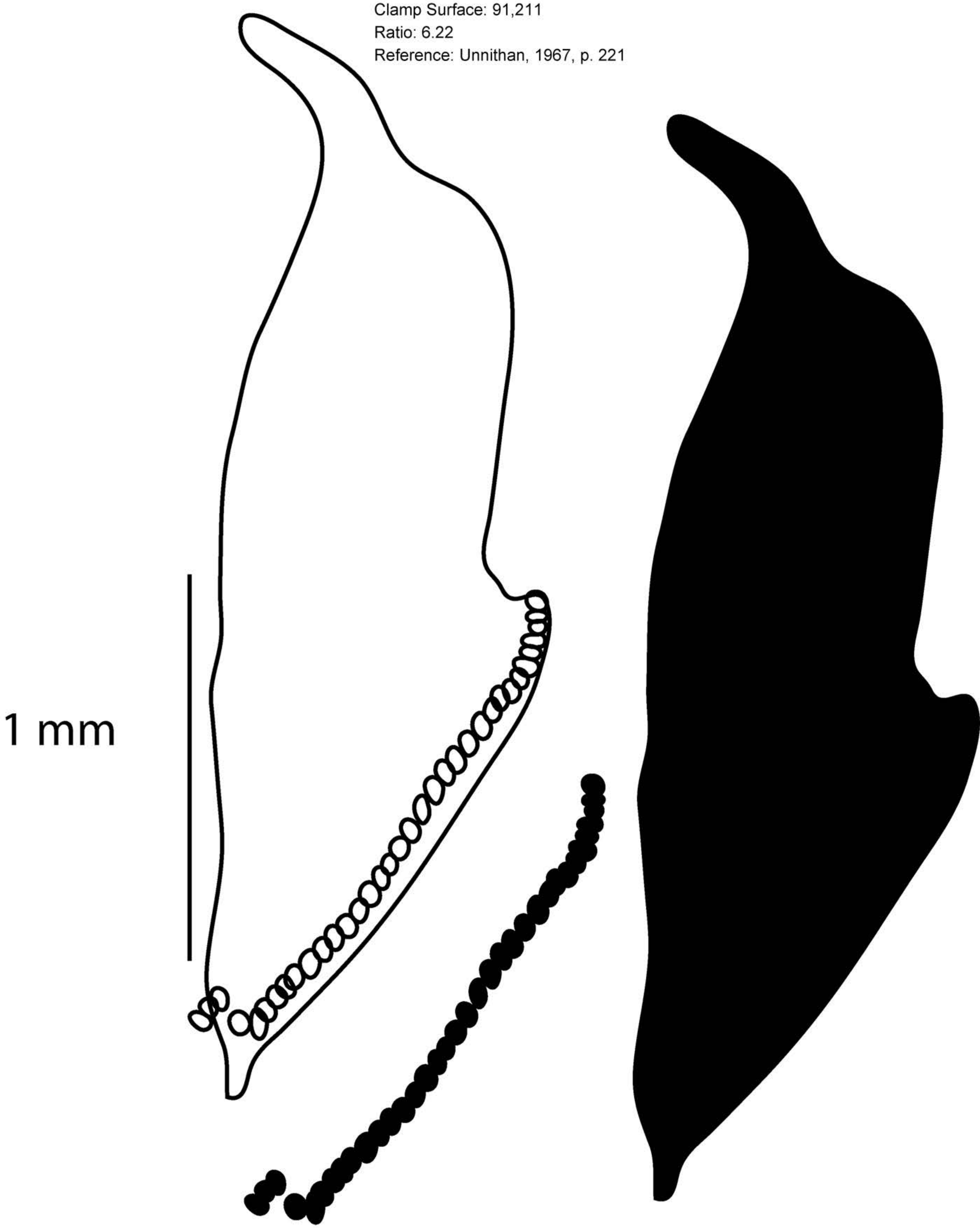

Family: Gastrocotylidae
Species: Engraulixenus malabaricus Unnithan, 1967
Body Surface: 556,699
Clamp Surface: 56,562
Ratio: 10.16
Reference: Unnithan, 1967, p. 215

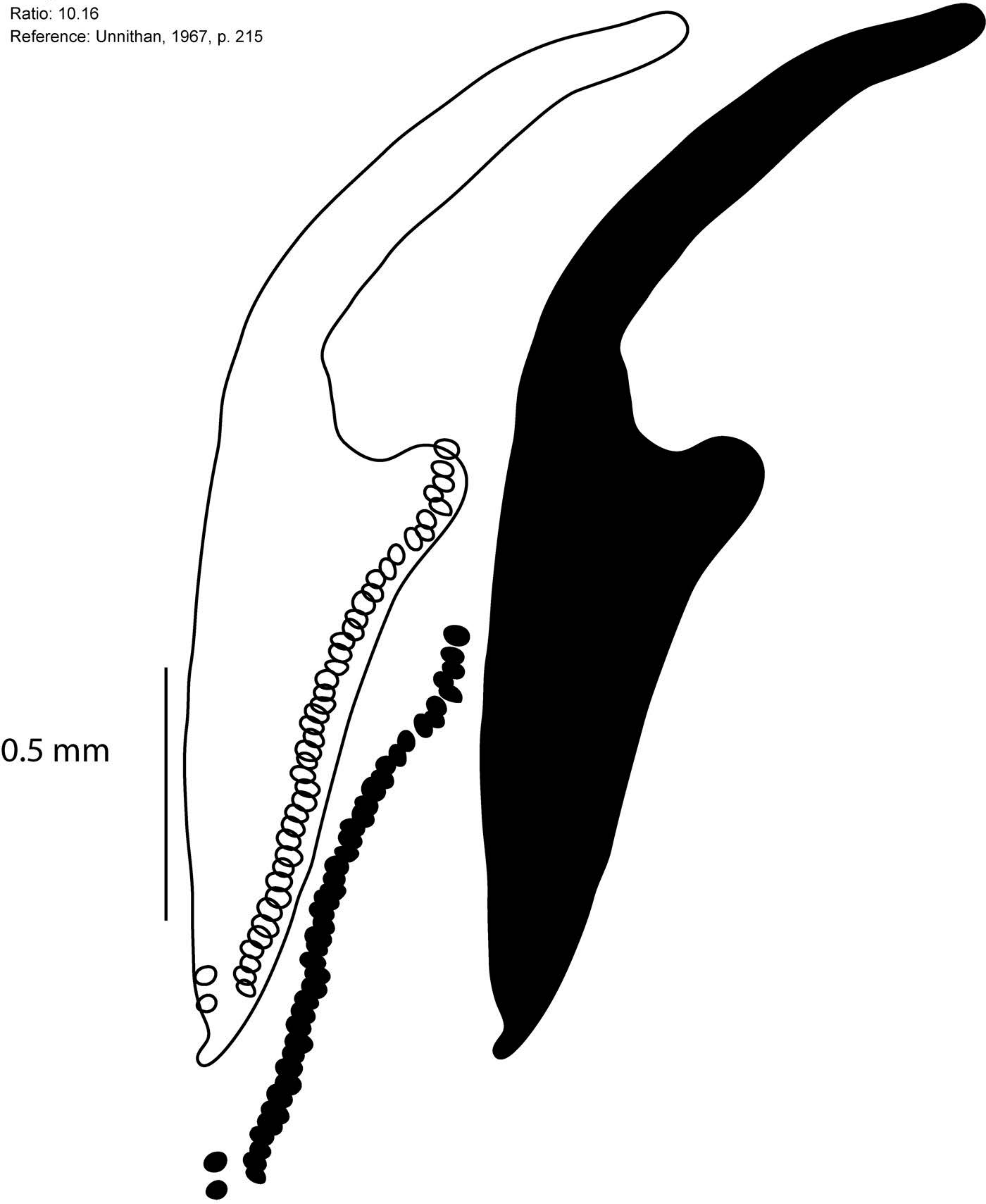

Family: Gastrocotylidae
Species: Eyelavera typica Unnithan, 1968
Body Surface: 9,500,350
Clamp Surface: 844,481
Ratio: 8.89
Reference: Lebedev, 1986, p. 74

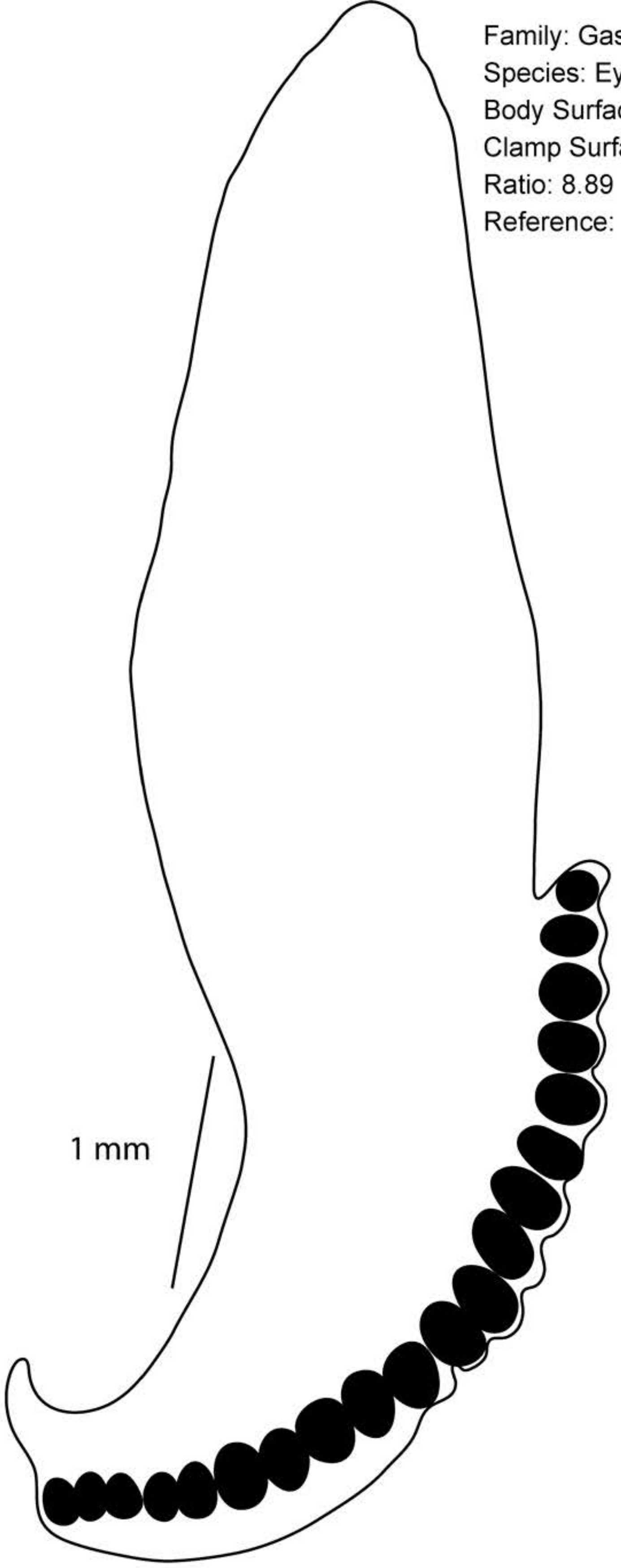

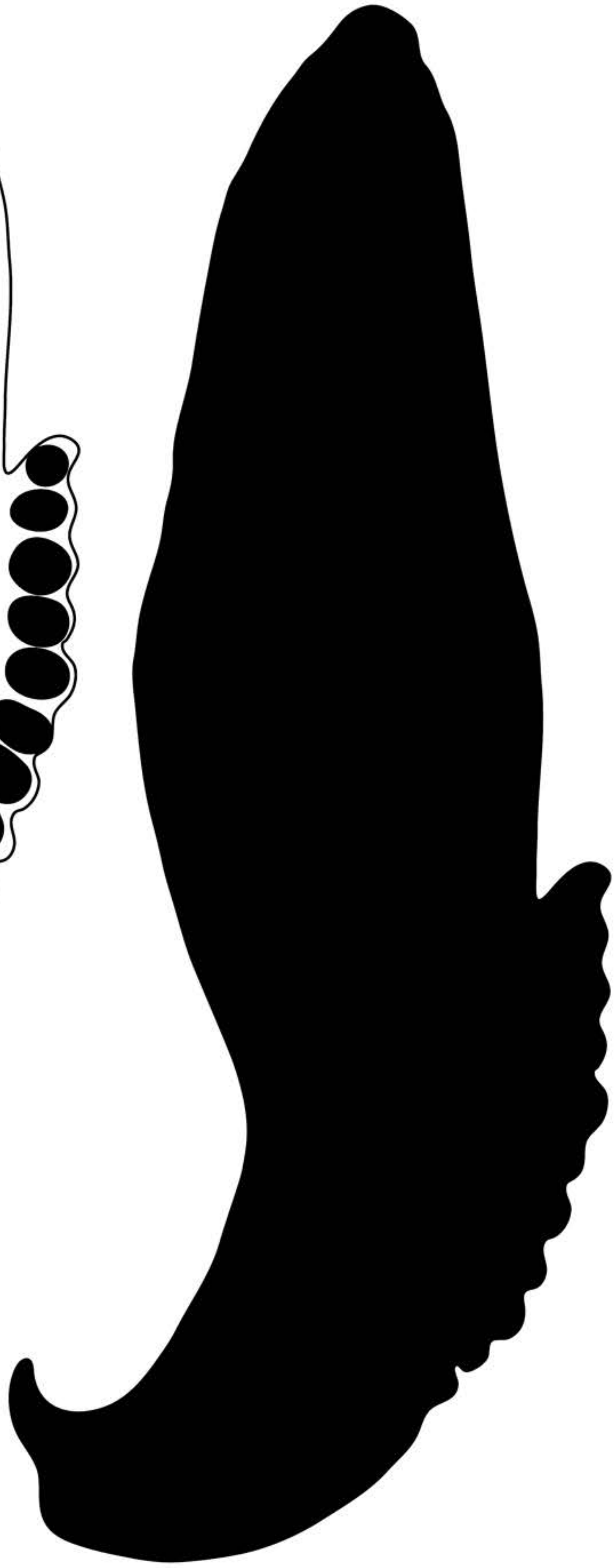

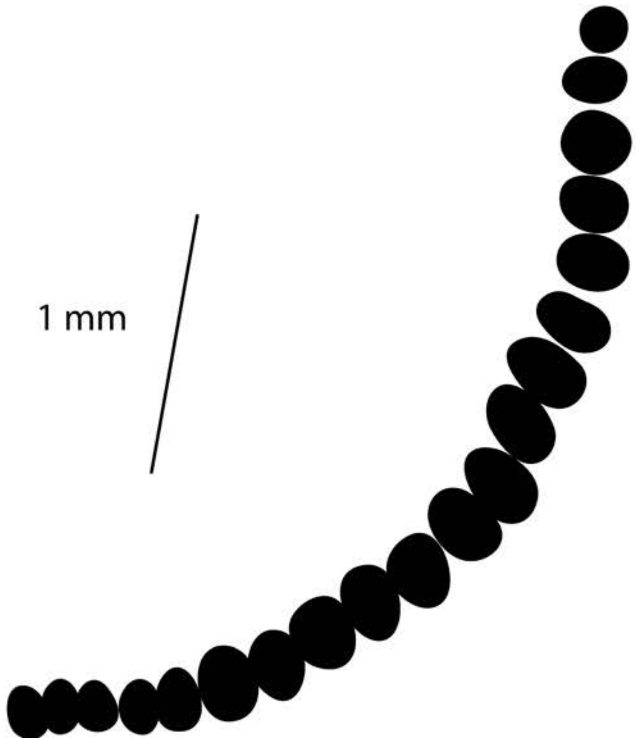

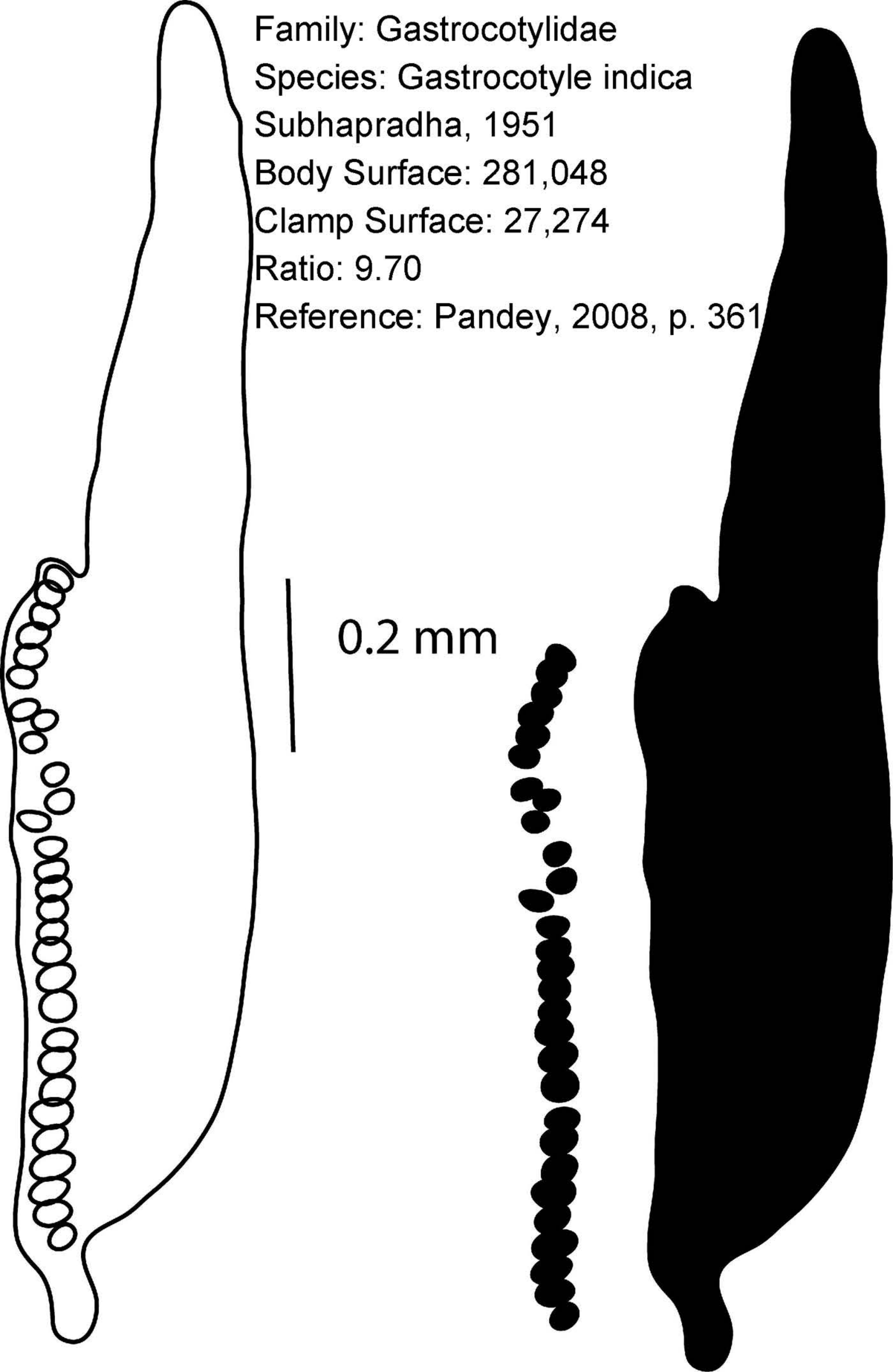
Family: Gastrocotylidae
Species: Gastrocotyle indica
Subhapradha, 1951
Body Surface: 281,048
Clamp Surface: 27,274
Ratio: 9.70
Reference: Pandey, 2008, p. 361
0.2 mm

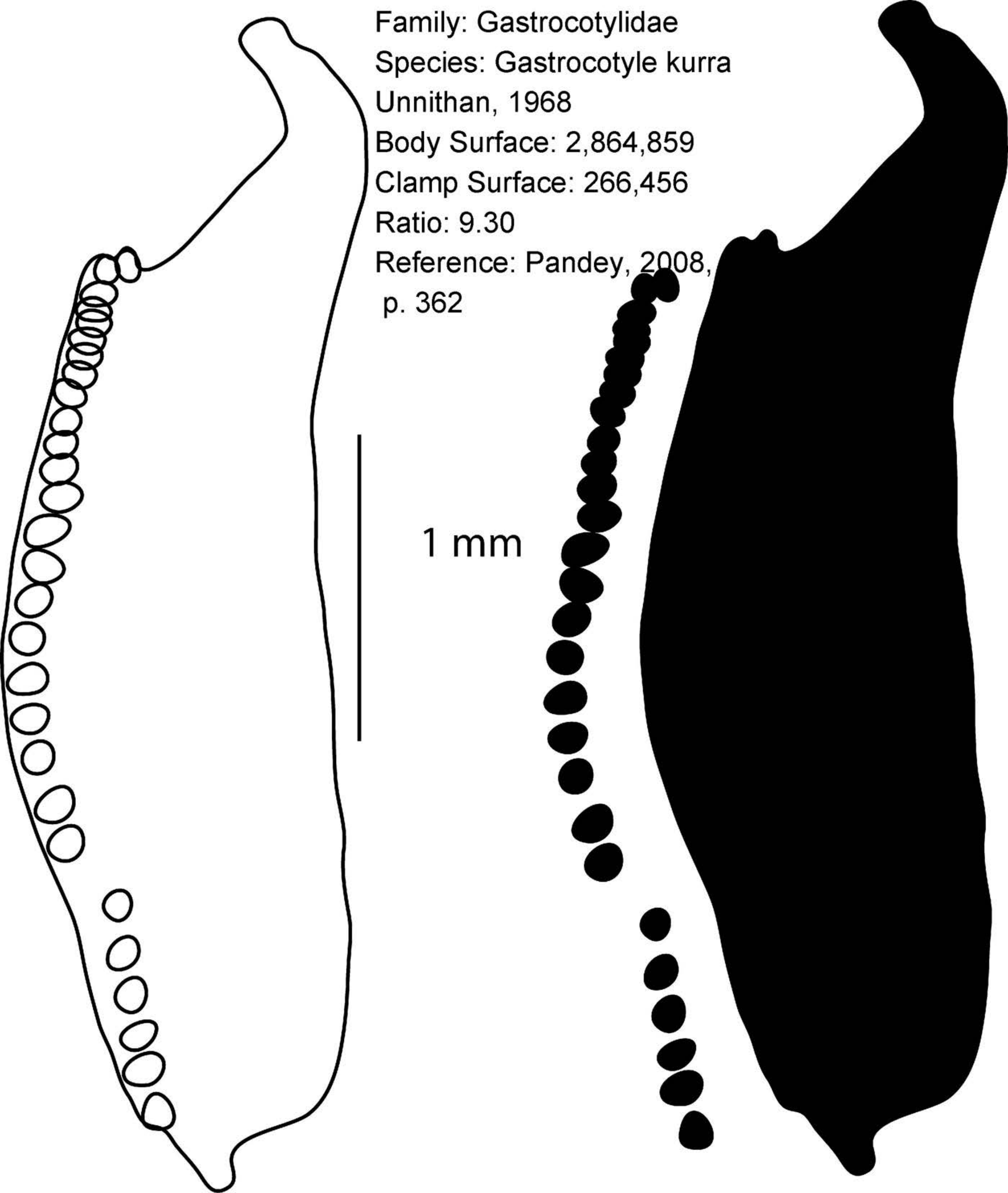
Family: Gastrocotylidae
Species: Gastrocotyle kurra
Unnithan, 1968
Body Surface: 2,864,859
Clamp Surface: 266,456
Ratio: 9.30
Reference: Pandey, 2008,
p. 362
1 mm

Family: Gastrocotylidae
Species: Gastrocotyloides dillonhargisi Lebedev, 1980
Body Surface: 1,273,059
Clamp Surface: 233,778
Ratio: 18.36
Reference: Lebedev, 1986, p. 72

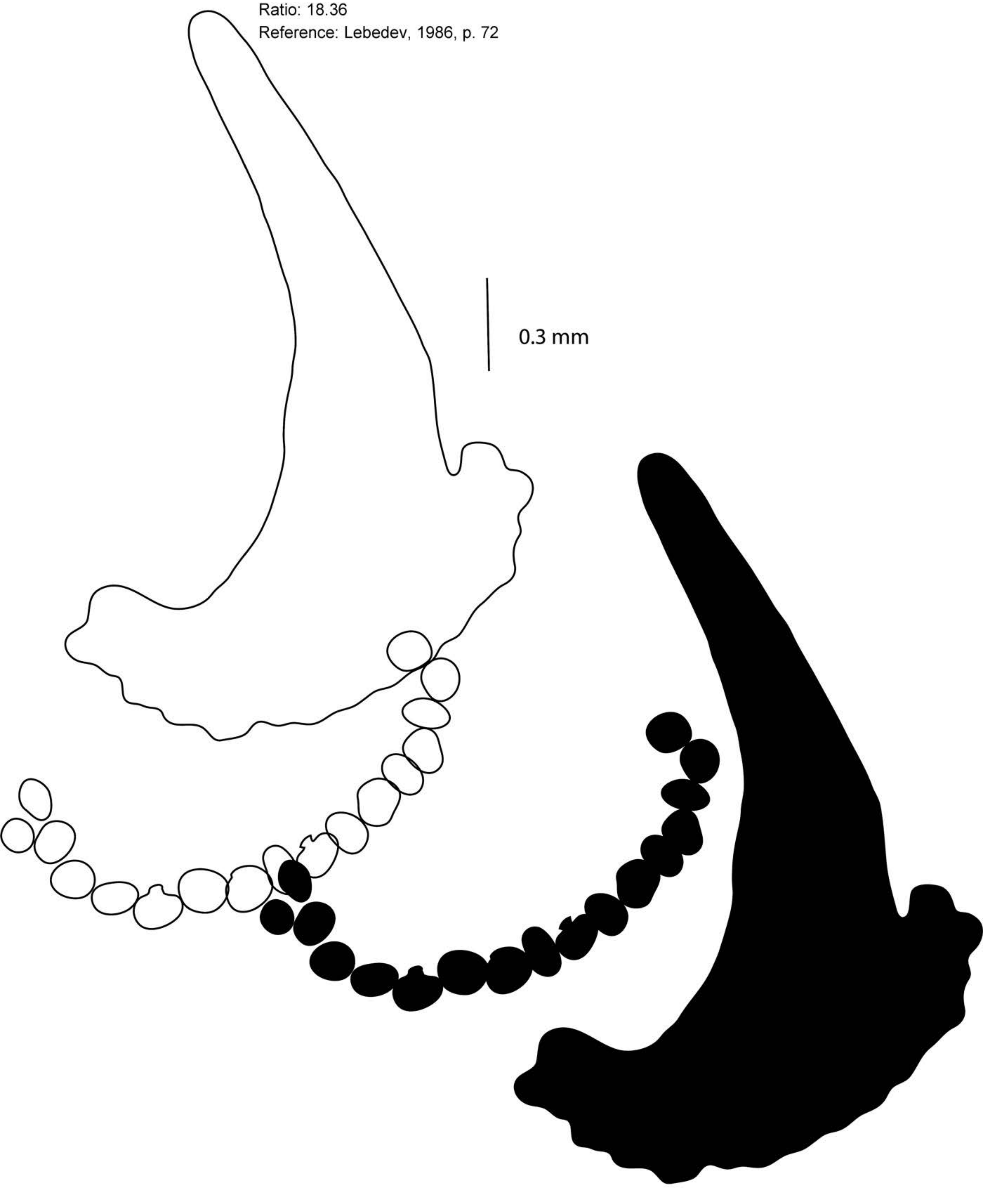

Family: Gastrocotylidae
Speceis : Irinaxine miniata Ghichenok, 1980
Body Surface: 741,990
Clamp Surface : 59,441
Ratio: 8.01
Reference: Lebedev, 1986, p. 60

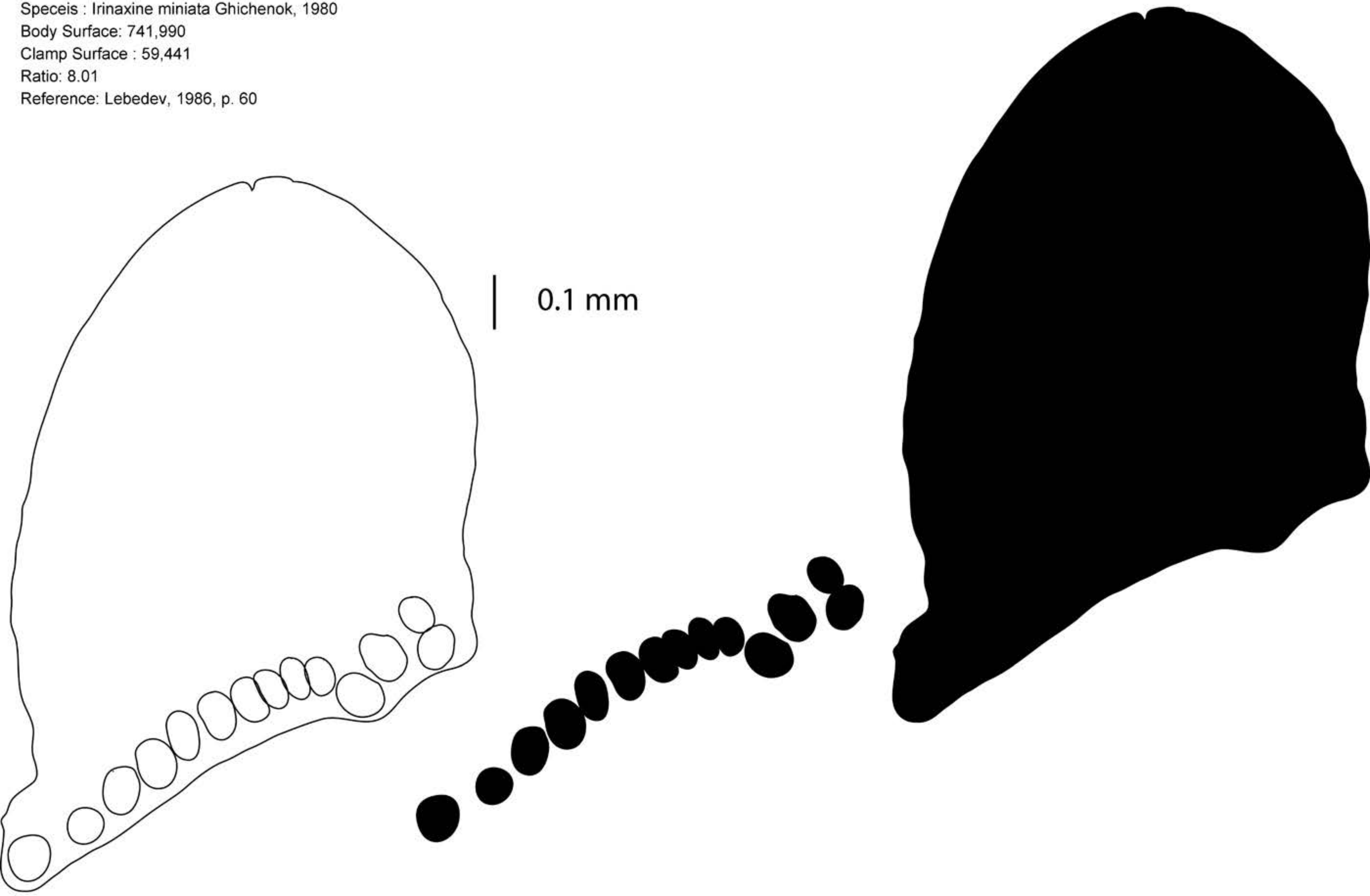

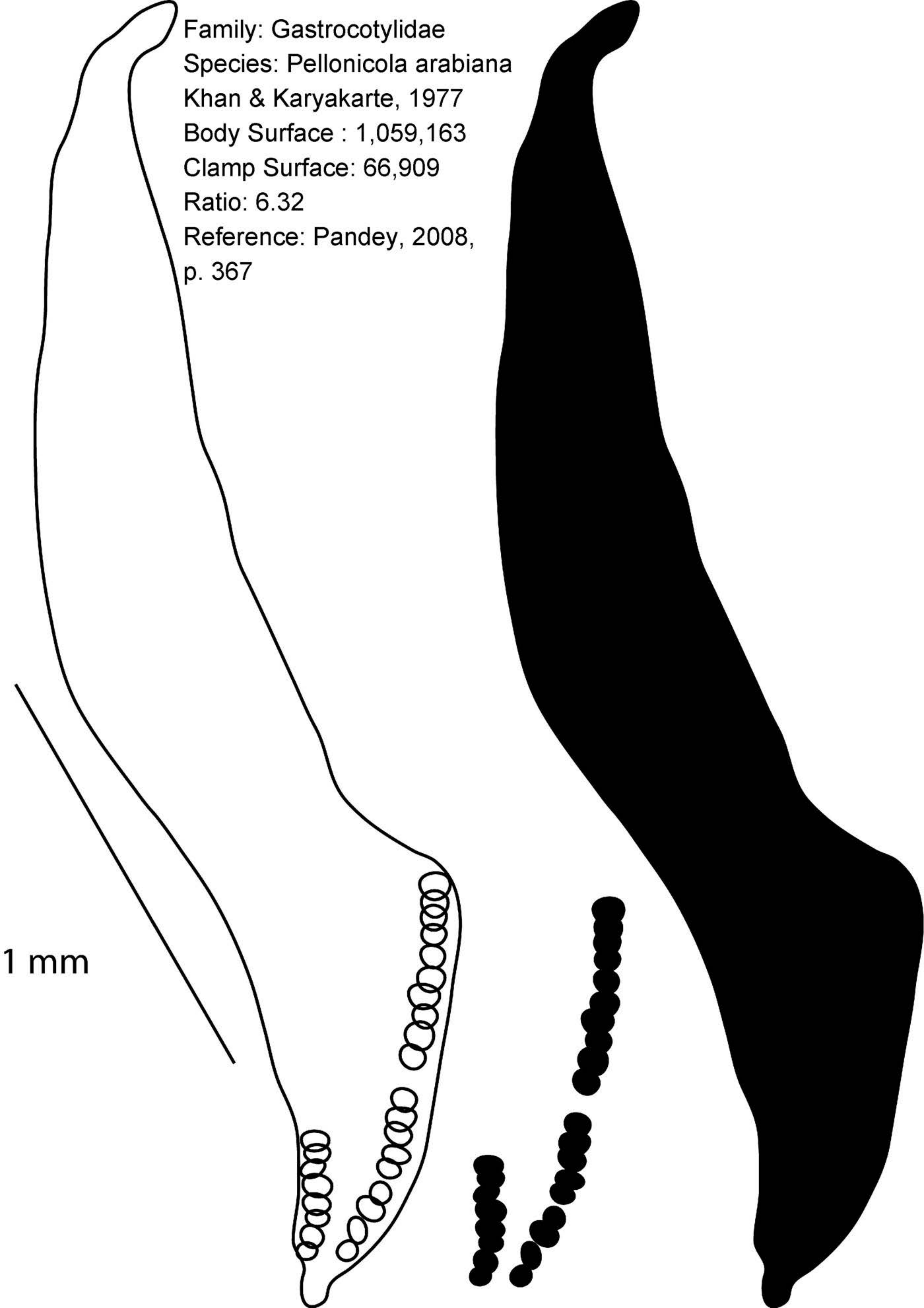
Family: Gastrocotylidae
Species: Pellonicola arabiana
Khan & Karyakarte, 1977
Body Surface : 1,059,163
Clamp Surface: 66,909
Ratio: 6.32
Reference: Pandey, 2008,
p. 367
1 mm

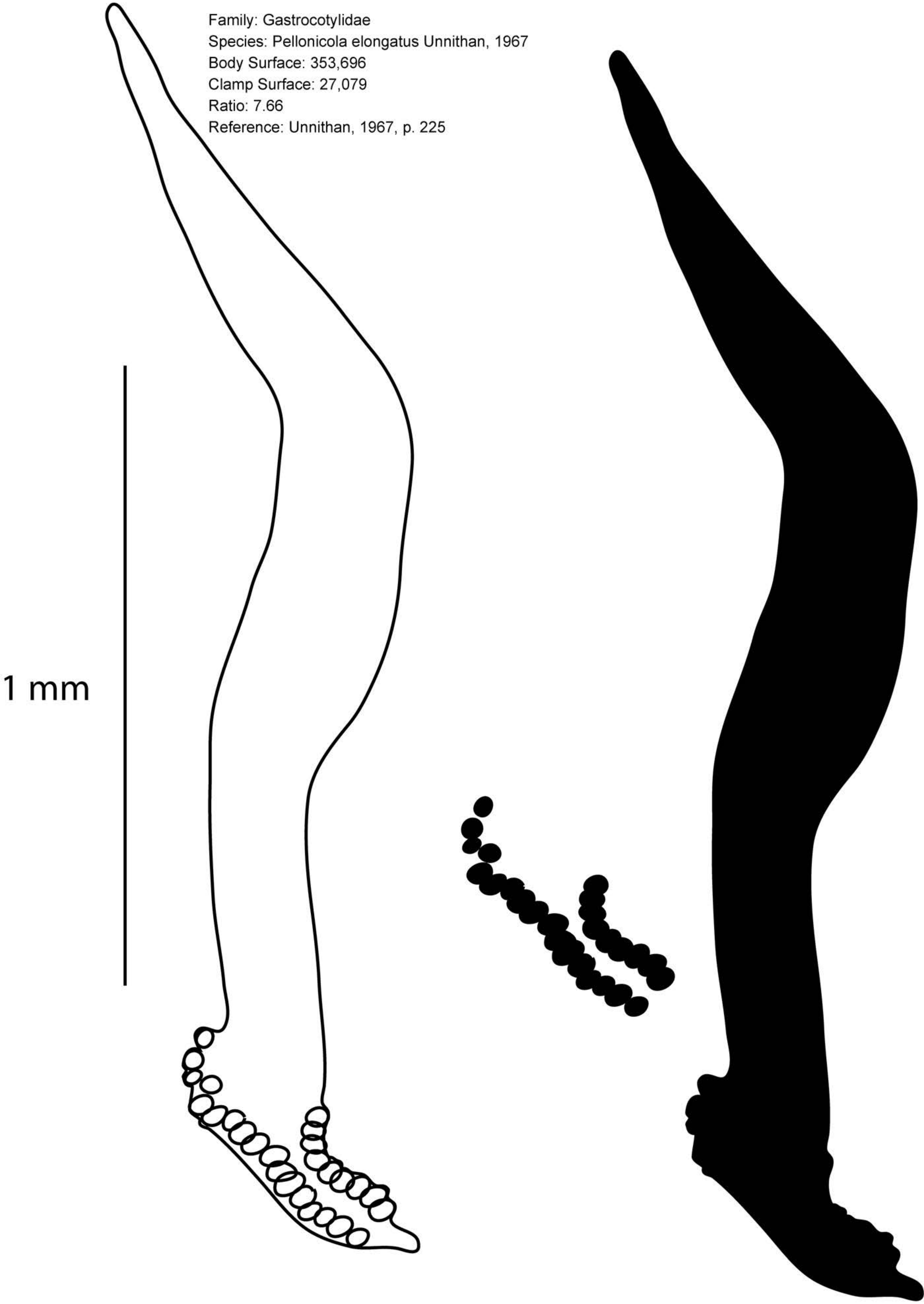
Family: Gastrocotylidae
Species: Pellonicola elongatus Unnithan, 1967
Body Surface: 353,696
Clamp Surface: 27,079
Ratio: 7.66
Reference: Unnithan, 1967, p. 225
1 mm

Family: Gastrocotylidae
Species: Pellonicola lanceolatus Kritsky & Bilqees, 1973
Body Surface: 1,785,588
Clamp Surface: 102,492
Ratio: 5.74
Reference: Kritsky, 1973, p. 198

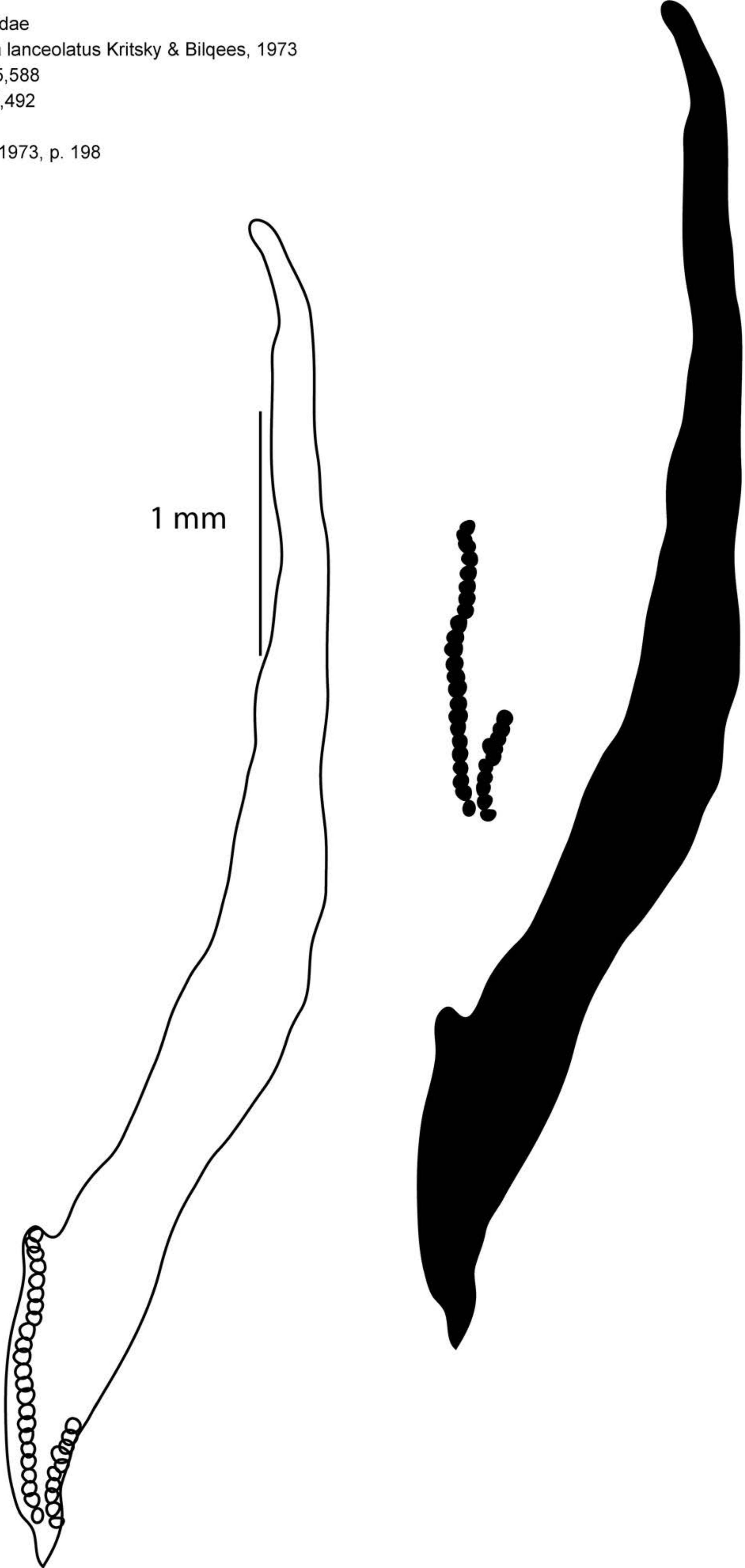

Family: Gastrocotylidae
Species: Pseudaxine bivaginalis
Dillon & Hargis, 1965
Body Surface: 1,137,648
Clamp Surface: 44,201
Ratio: 3.89
Reference: Dillon, 1965, p. 276

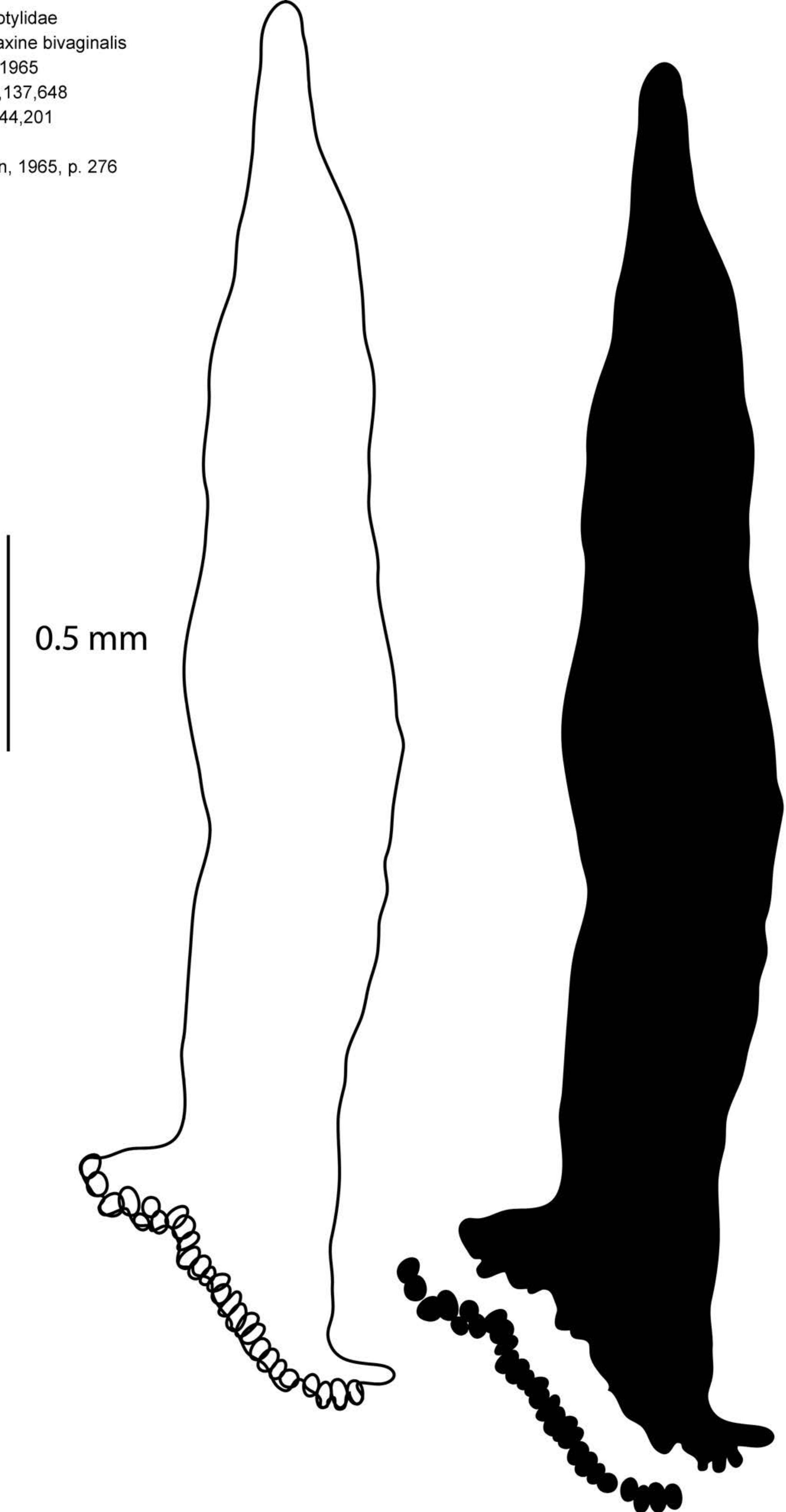

Family: Gastrocotylidae

Species: Pseudaxine kurra Unnithan, 1968

Body Surface: 1,909,954

Clamp Surface: 37,999

Ratio: 1.99

Reference: Zhang, 2001, p. 268

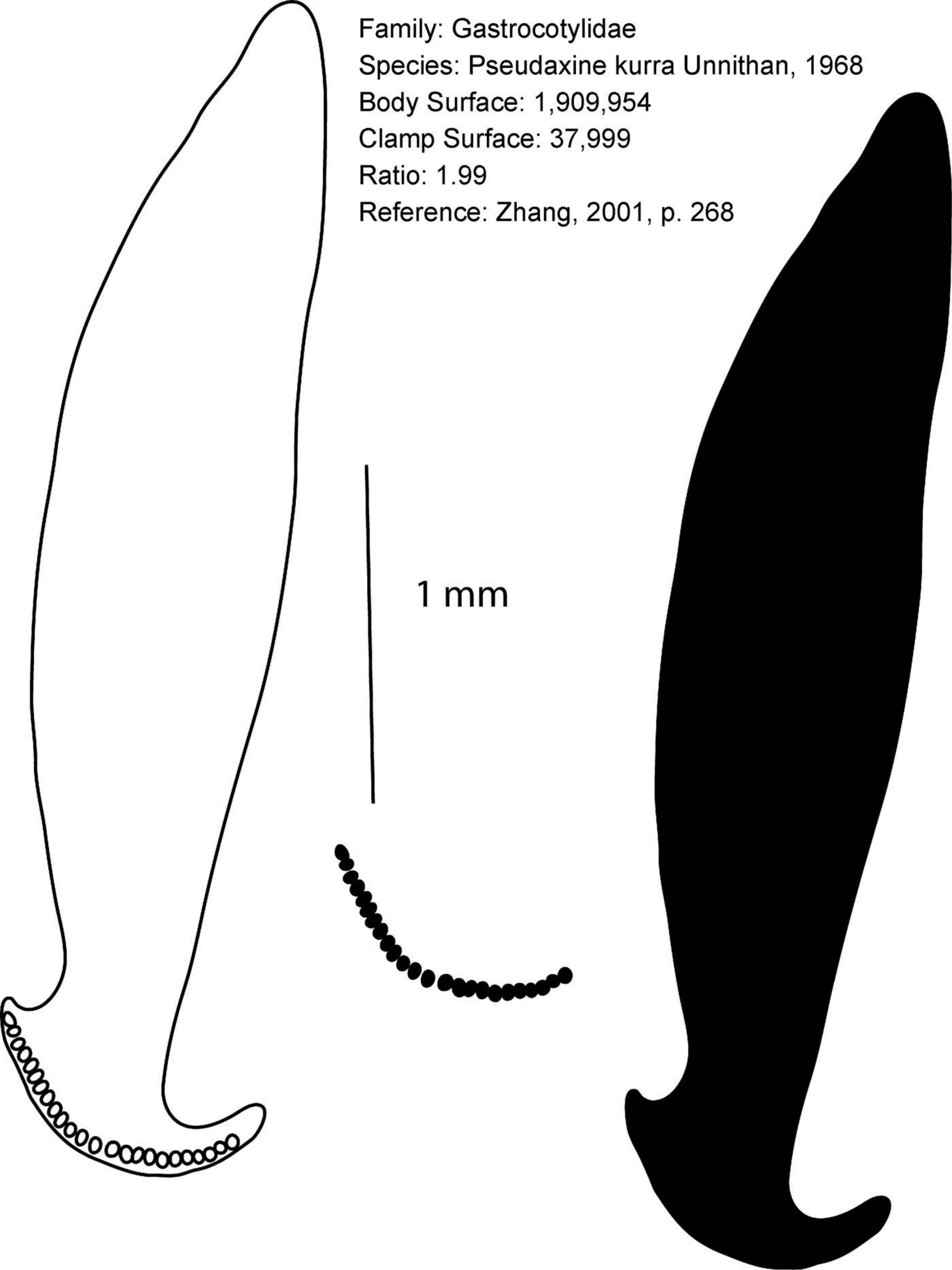

Family: Gastrocotylidae

Species: Pseudaxinoides caballeroi Lebedev, 1977

Body Surface: 2,096,832

Clamp Surface: 93,128

Ratio: 4.44

Reference: Lebedev, 1986, p. 57

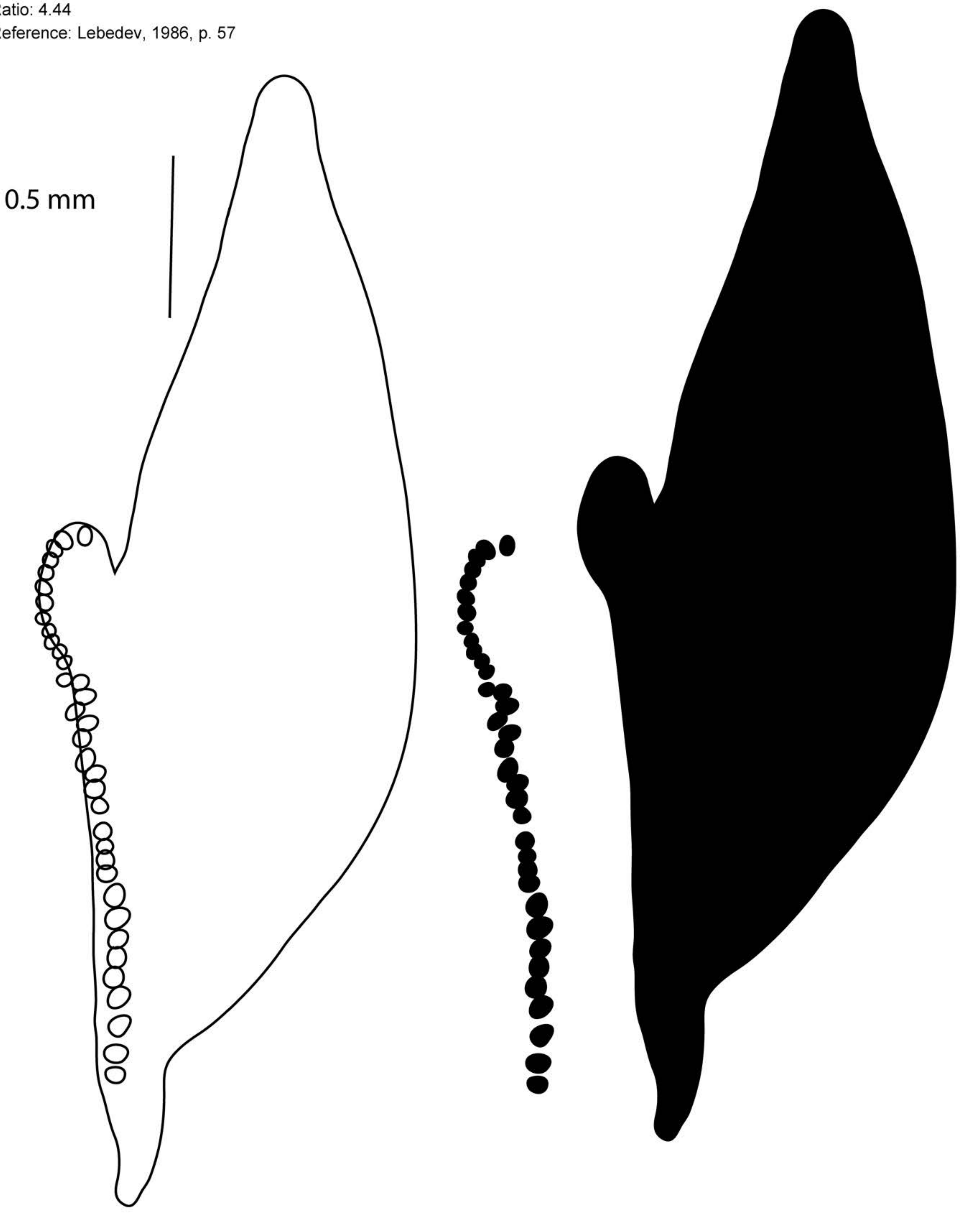

Family: Gastrocotylidae
Species: Quadrivalvula asymmetrica Ghichenok, 1980
Body surface: 3,430,344
Clamp surface: 994,784
Ratio: 29.00
Reference: Lebedev, 1986, p. 77

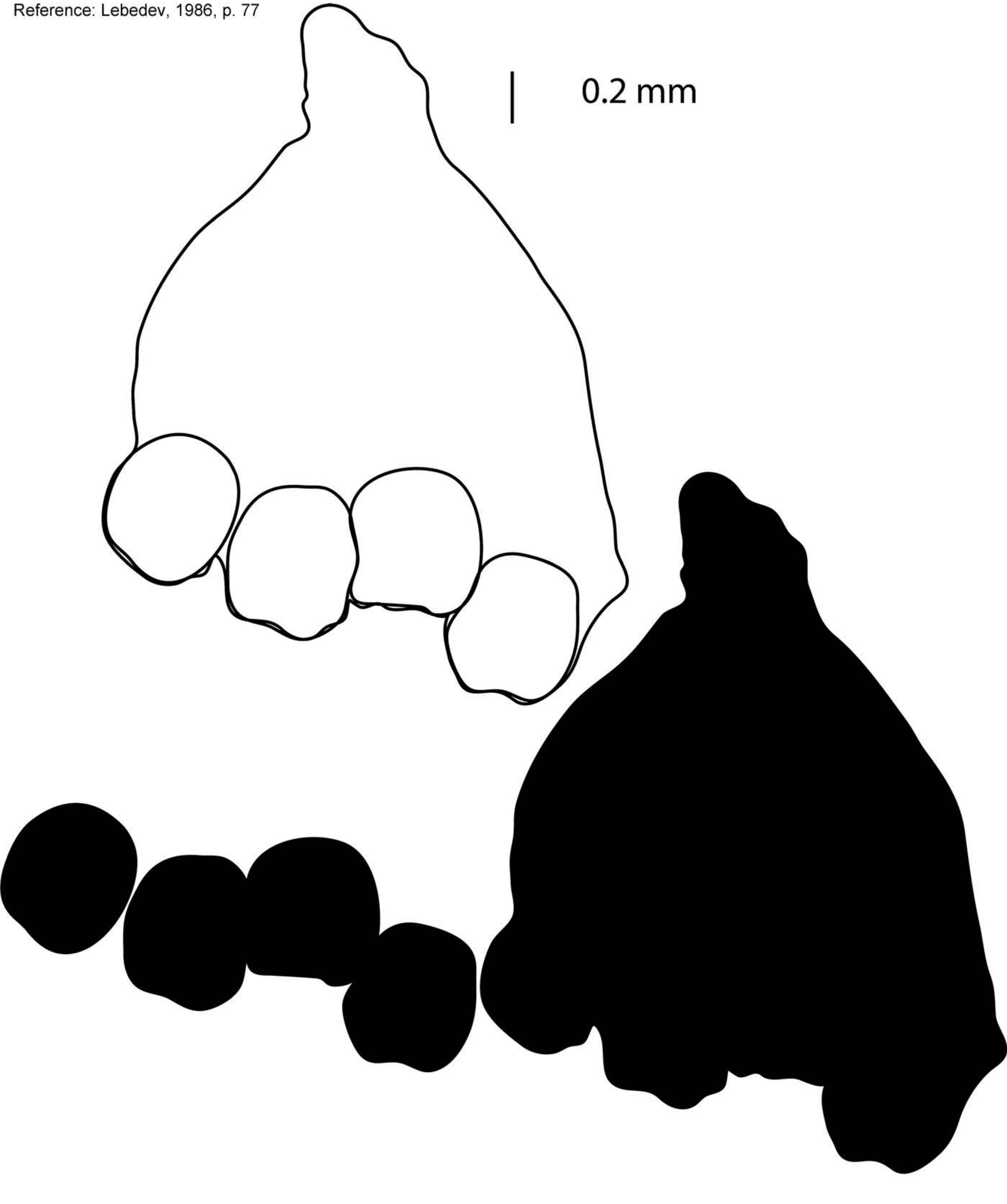

Family: Gastrocotylidae
Species: Sibitrema poonui Yamaguti, 1966
Body Surface: 15,402,407
Clamp Surface: 235,741
Ratio: 1.53
Reference: Yamaguti, 1966, p. 430

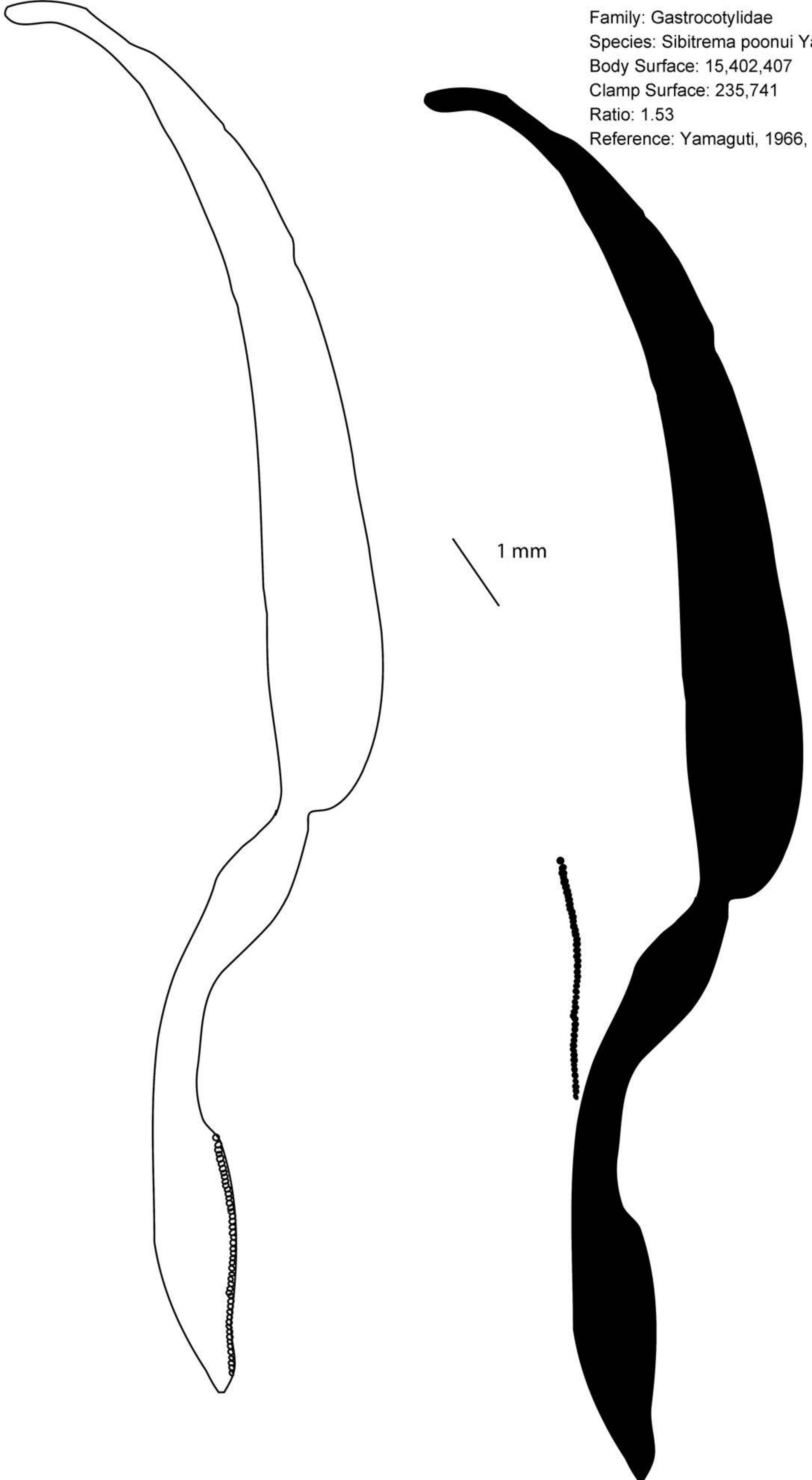

Family: Gotocotylidae
Species: Cathucotyle cathuaui Lebedev, 1968
Body Surface: 1,303,159
Clamp Surface: 261,873
Ratio: 20.10
Reference: Hayward, 1999, p. 450

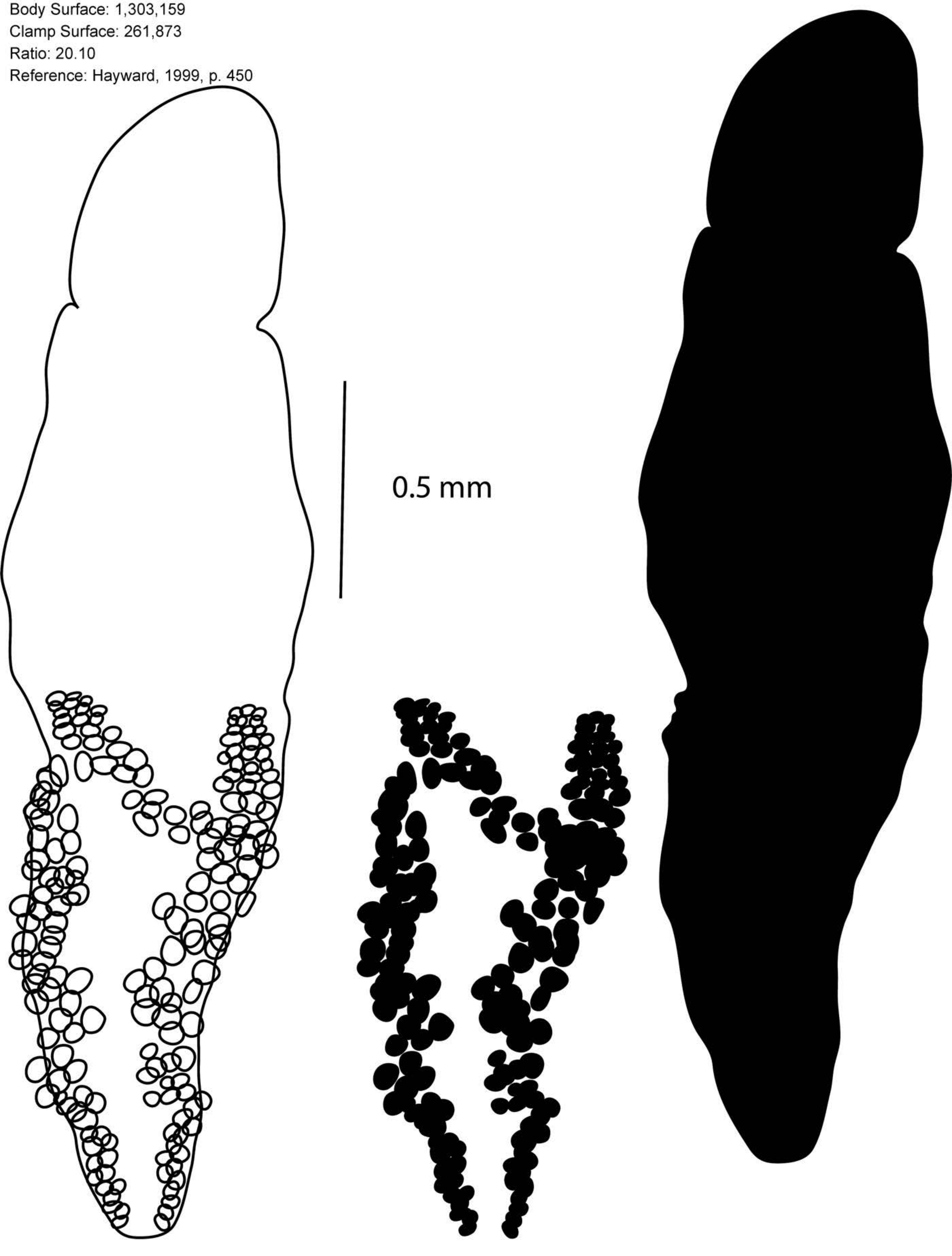

Family: Gotocotylidae
Species: Cathucotyle filipinensis
Hayward & Rohde, 1999
Body Surface: 2,971,409
Clamp Surface: 413,969
Ratio: 13.93
Reference: Hayward, 1999, p. 453

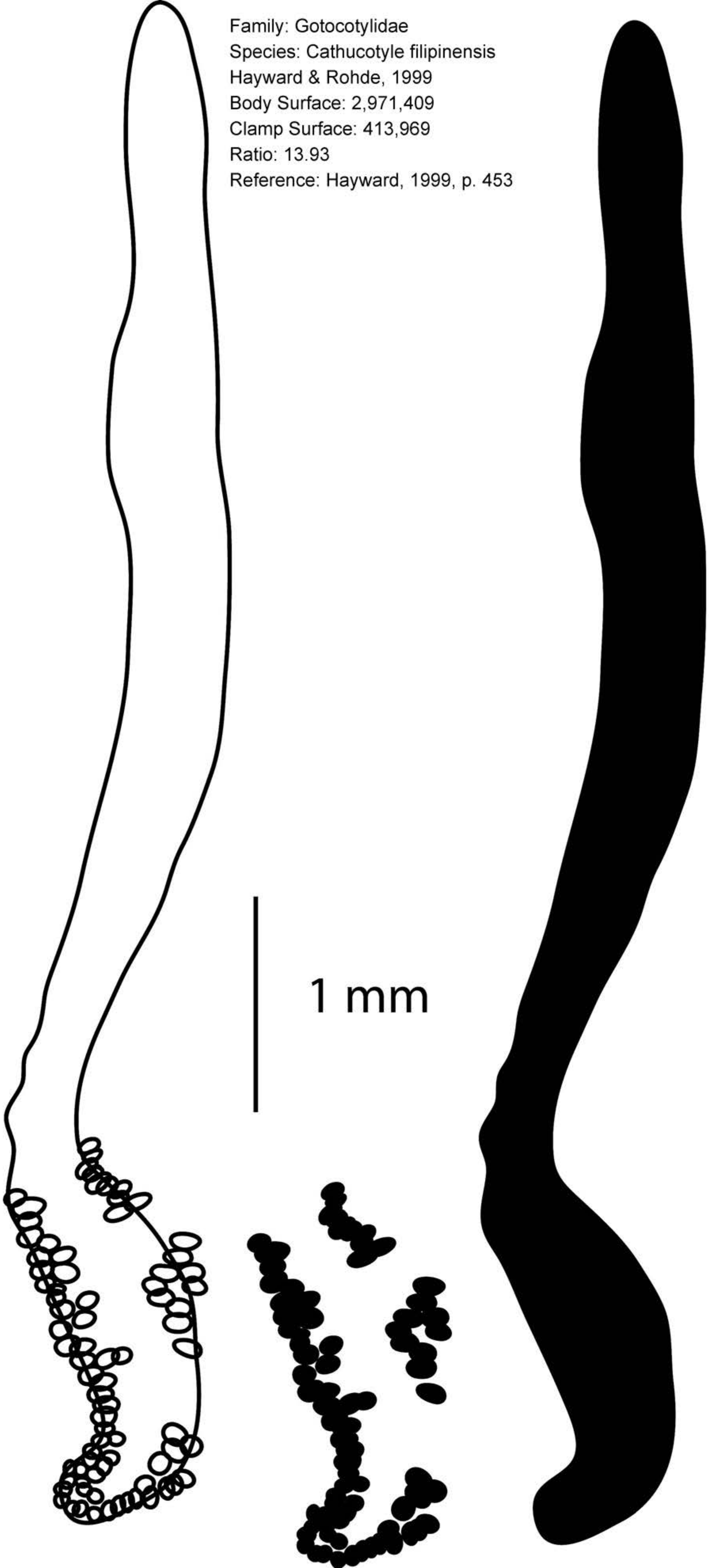

Family: Gotocotylidae
Species: Cathucotyle sinensis Hayward & Rohde, 1999
Body Surface: 22,961,031
Clamp Surface: 1,562,400
Ratio: 6.80
Reference: Hayward, 1999, p. 455

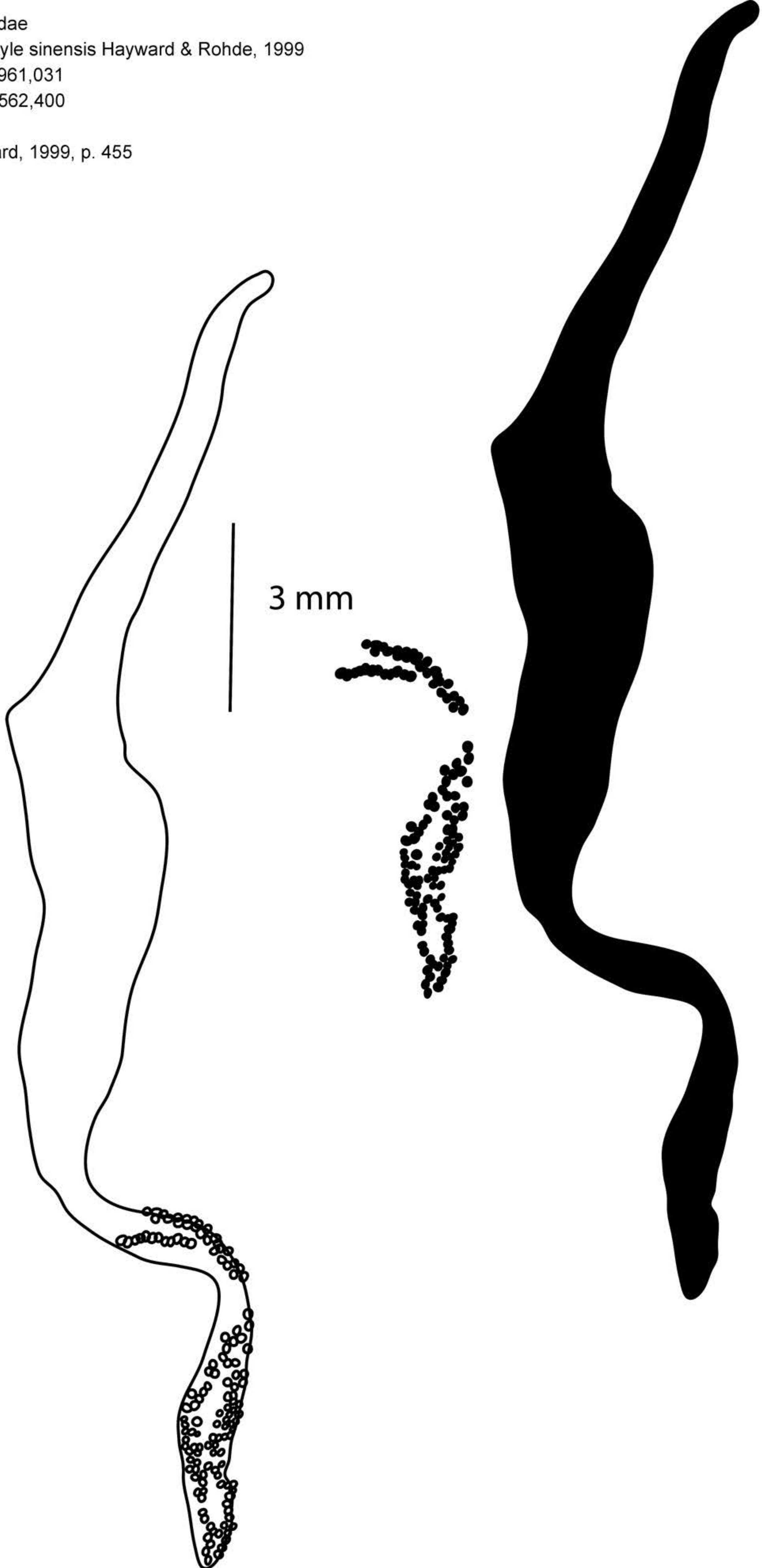

Family: Gotocotylidae

Species: Gotocotyla acanthura (Parona & Perugia, 1896) Meserve, 1938

Body Surface: 3,209,235

Clamp Surface: 466,446

Ratio: 14.53

Reference: Hayward, 1999, p. 431

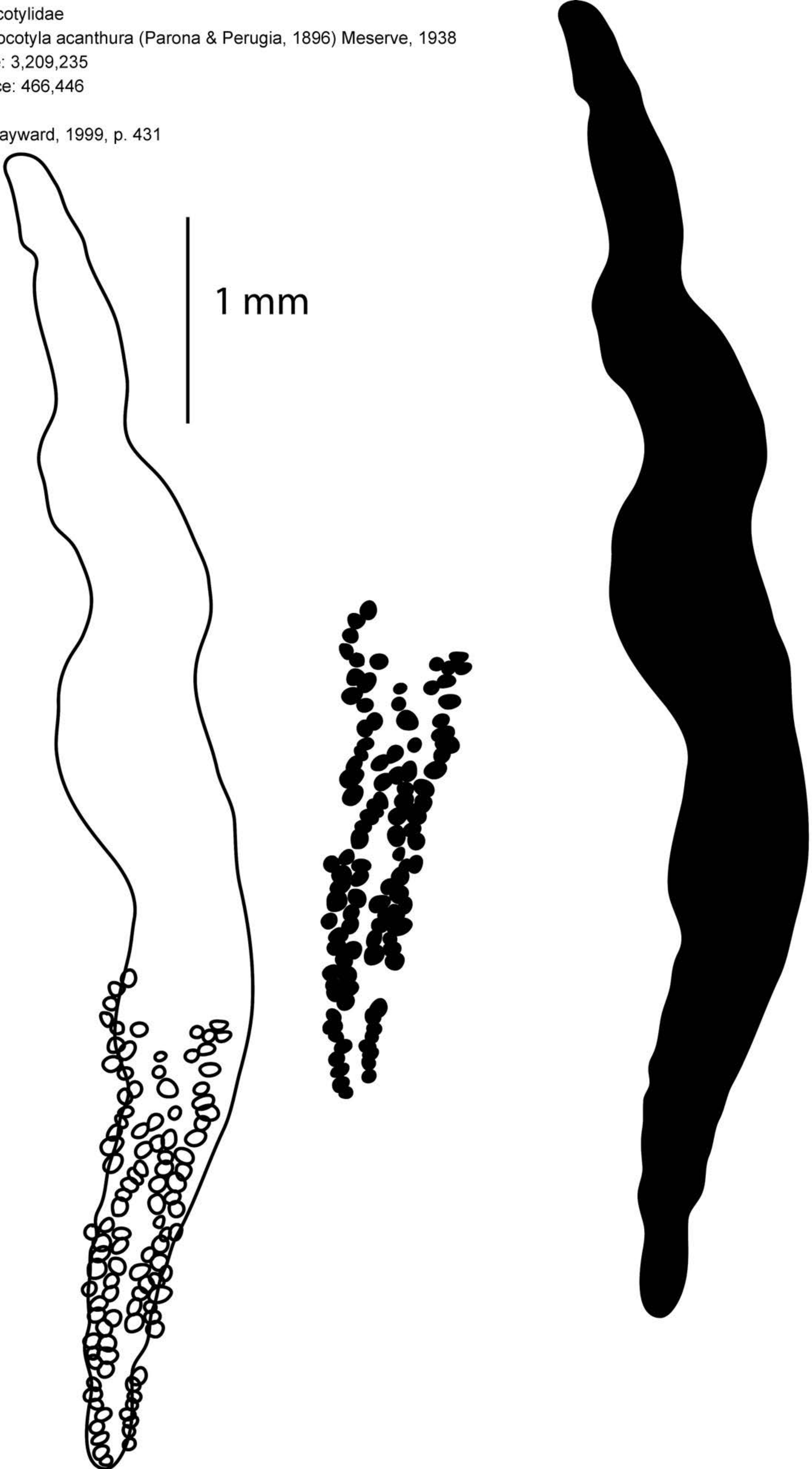

Family: Gotocotylidae
Species: Gotocotyla africanensis Hayward & Rohde, 1999
Body Surface: 2,445,683
Clamp Surface: 250,315
Ratio: 10.23
Reference: Hayward, 1999, p. 438

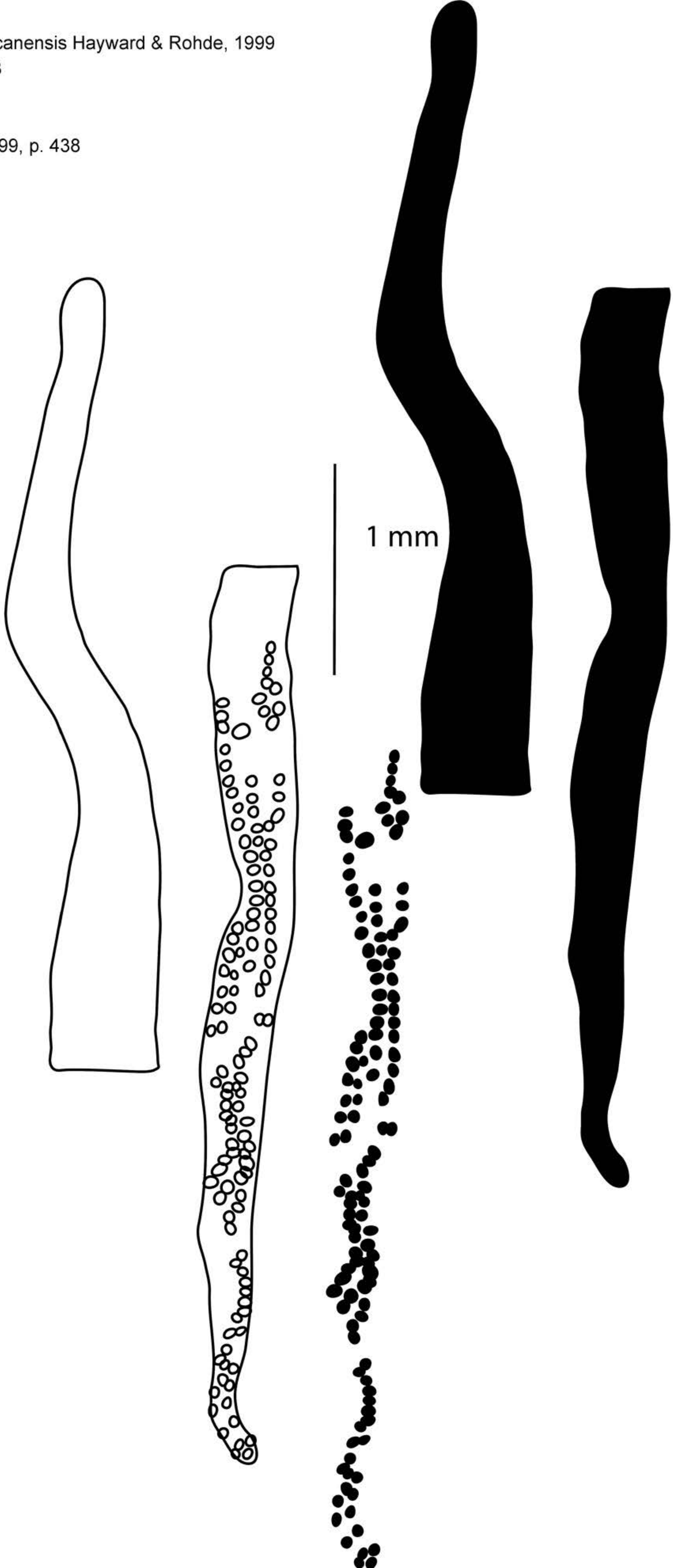

Family: Gotocotylidae
Species: Gotocotyla bivaginalis (Ramalingam, 1961) Rohde, 1976
Body Surface: 7,414,296
Clamp Surface: 274,996
Ratio: 3.71
Reference: Hayward, 1999, p. 440

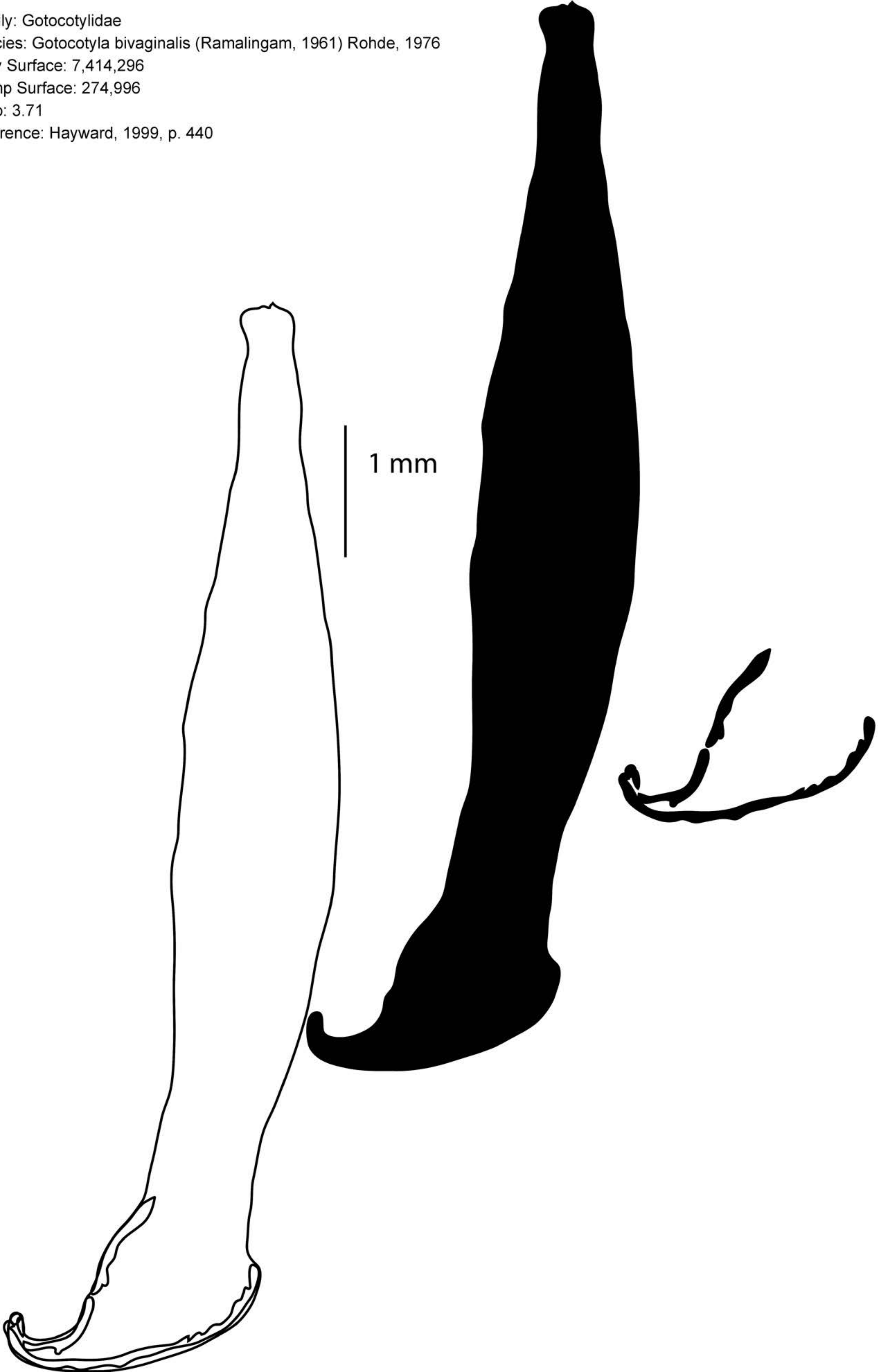

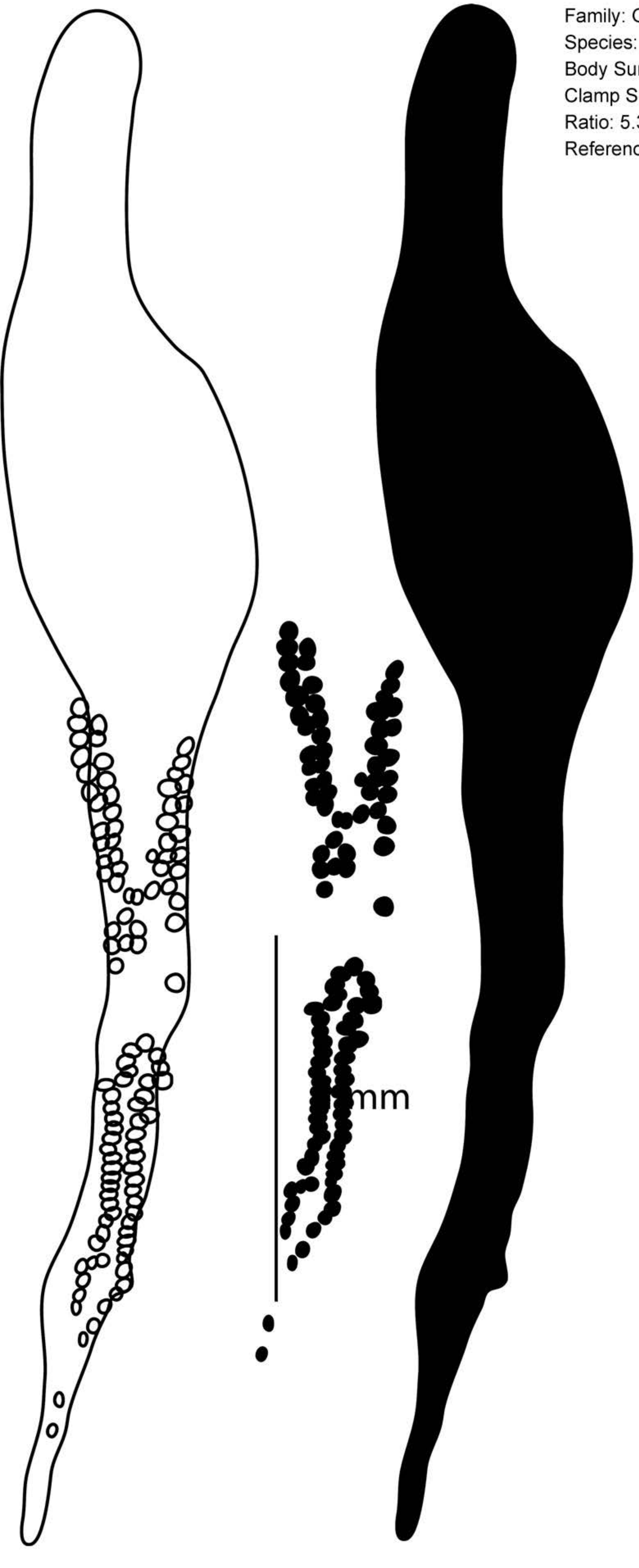

Family: Gotocotylidae

Species: Gotocotyla heapae Hayward & Rohde, 1999

Body Surface: 1,291,877

Clamp Surface: 69,182

Ratio: 5.36

Reference: Hayward, 1999, p. 443

Family: Gotocotylidae
Species: Gotocotyla meservei Yamaguti, 1953
Body Surface: 1,314,267
Clamp Surface: 179,701
Ratio: 13.67
Reference: Yamaguti, 1953, p. 56

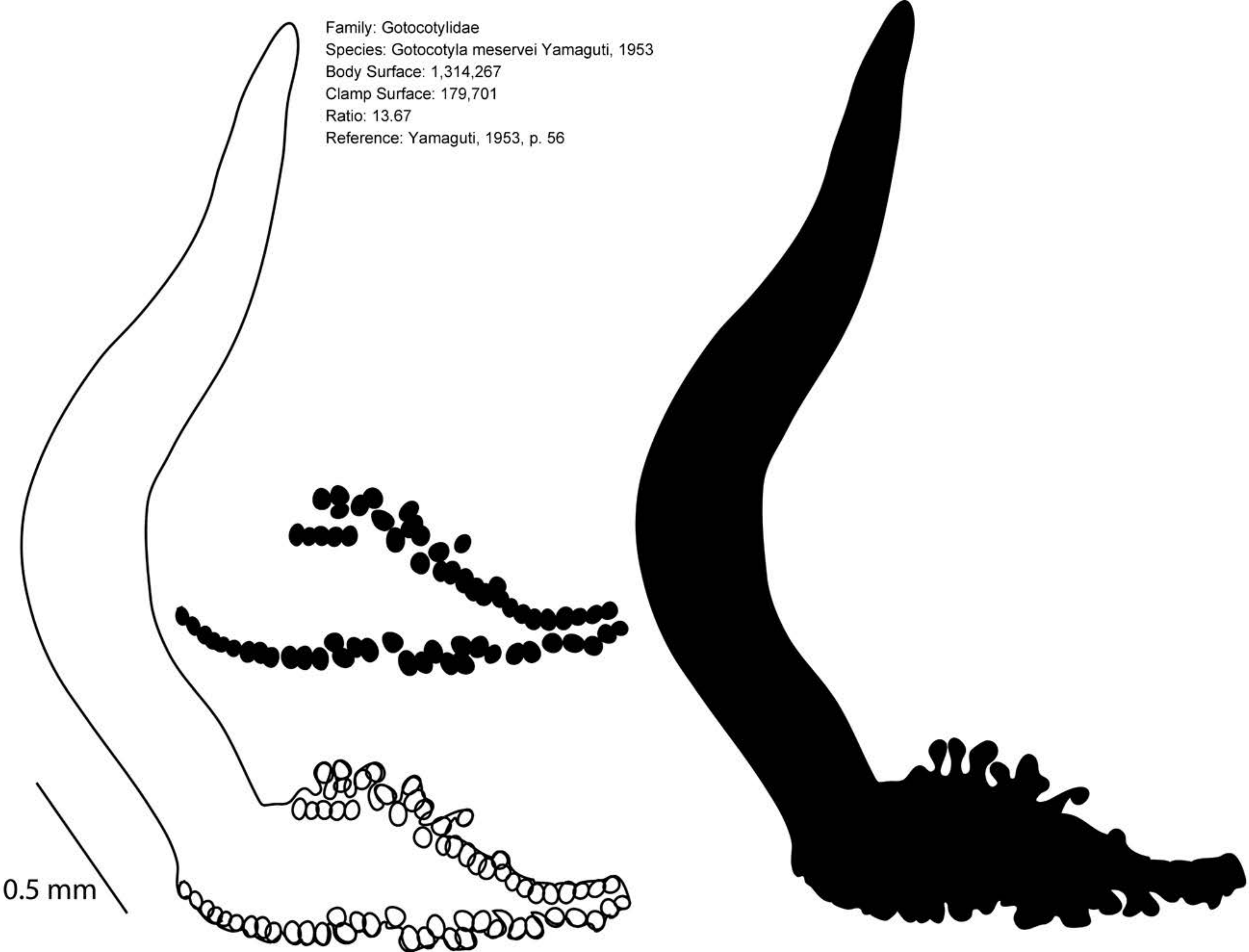

Family: Gotocotylidae
Species: Gotocotyla niphonii
Hayward & Rohde, 1999
Body Surface: 1,932,305
Clamp Surface: 171,386
Ratio: 8.87
Reference: Hayward, 1999, p. 445

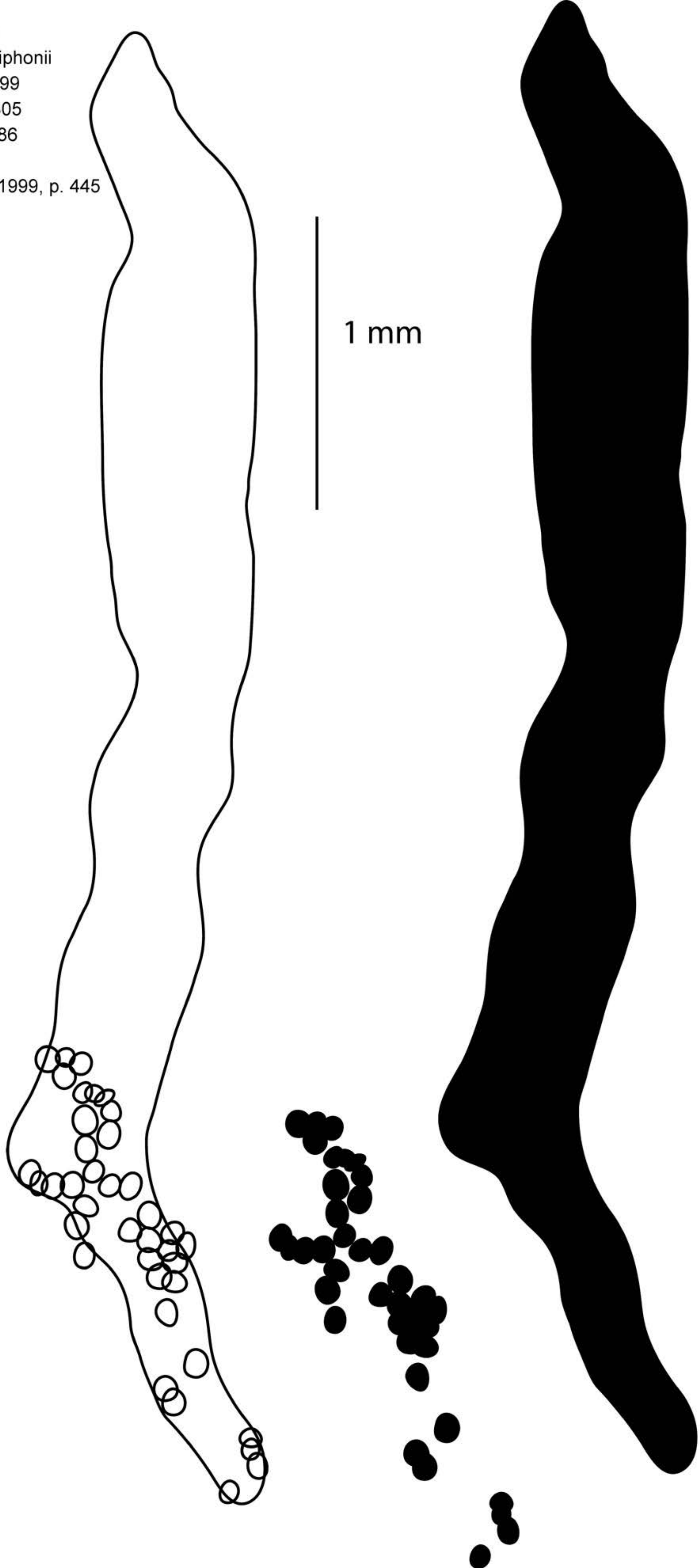

Family: Gotocotylidae
Species: Gotocotyla queenslandici Hayward & Rohde, 1999
Body Surface: 1,321,566
Clamp Surface: 258,123
Ratio: 19.53
Reference: Hayward, 1999, p. 447

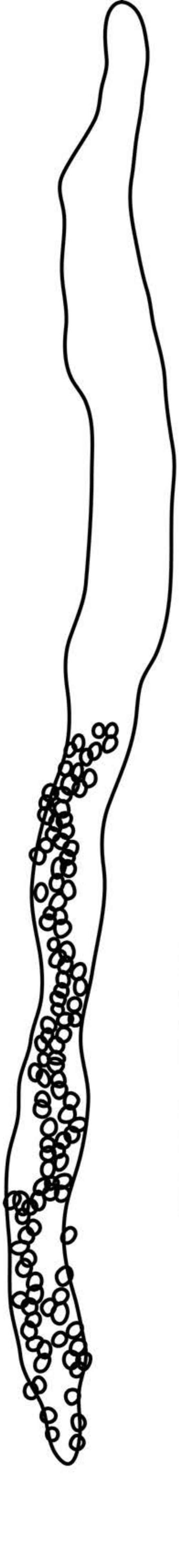

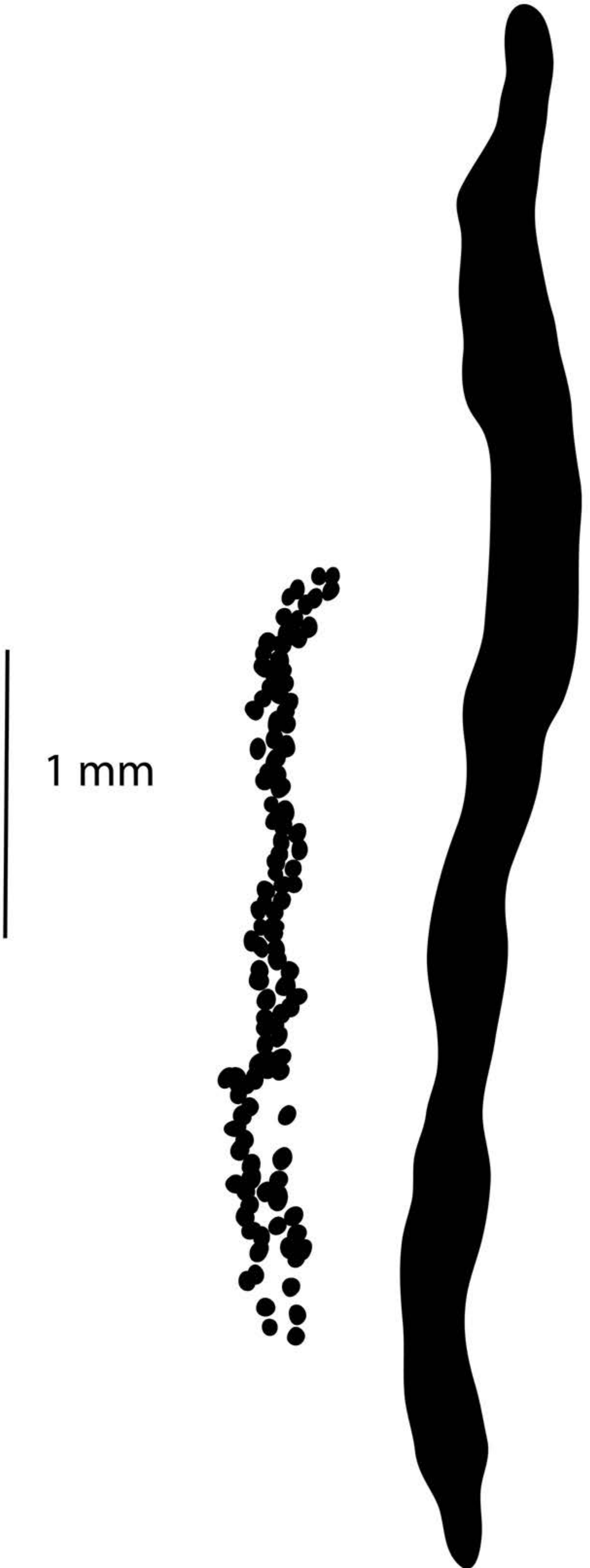

Family: Gotocotylidae
Species: Neogotocotyla rohdii Hadi & Bilqees, 2010
Body Surface: 2,991,506
Clamp Surface: 347,296
Ratio: 11.61
Reference: Hadi, 2010, p. 22

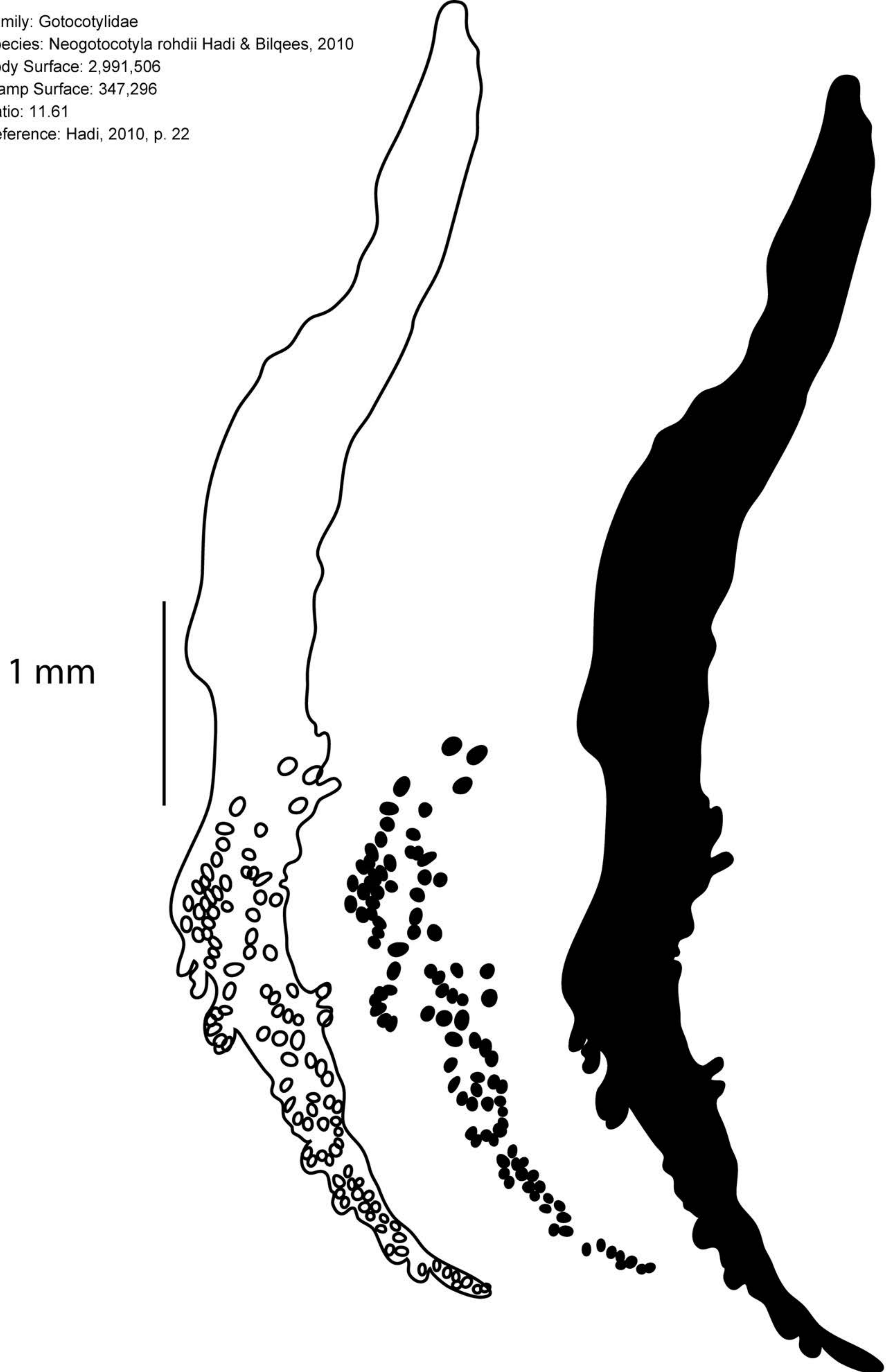

Family: Neothoracocotoylidae
Species: Mexicotyle mexicana (Meserve, 1938) Lebedev, 1984
Body Surface: 2,566,693
Clamp Surface: 133,237
Ratio: 5.19
Reference: Lebedev, 1986, p. 90

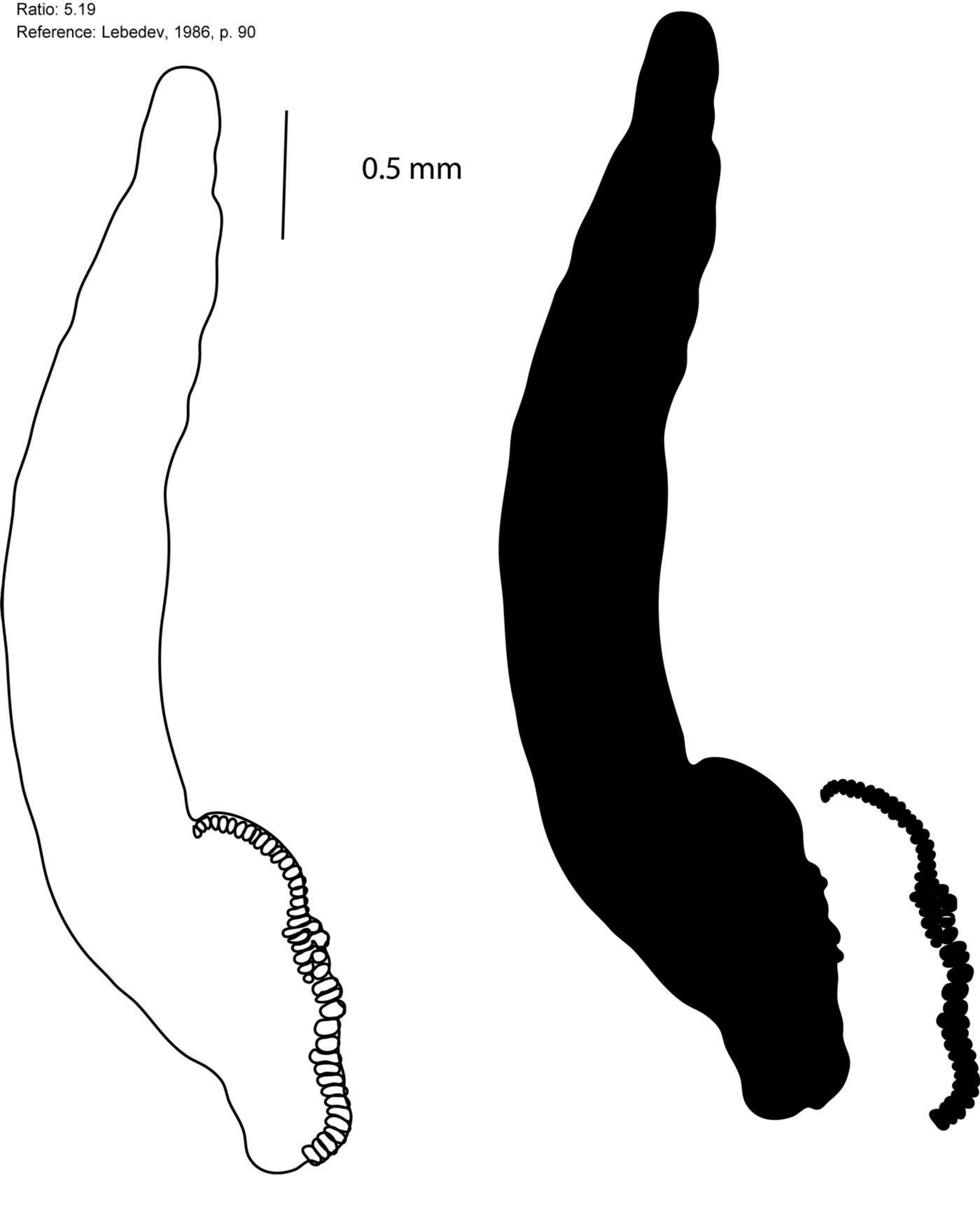

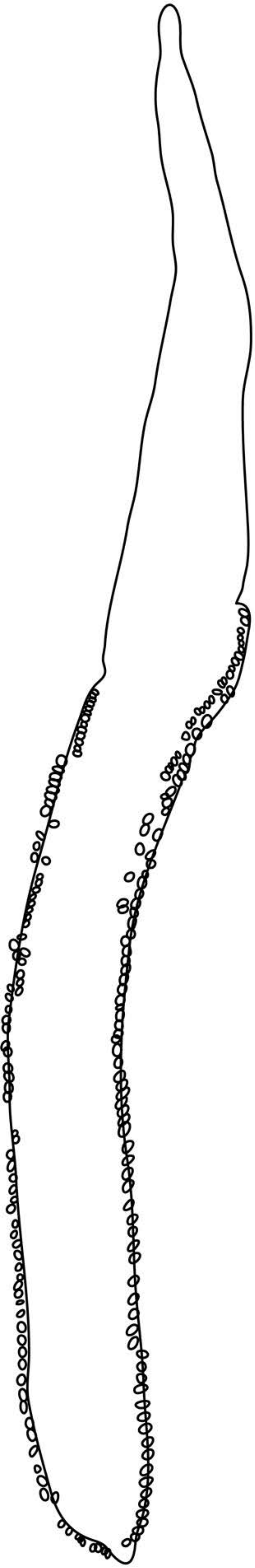

Family: Neothoracocotoylidae

Species: Neothoracocotyle acanthocybii (Meserve, 1938) Hargis, 1956

Body Surface: 126,796

Clamp Surface: 12,940

Ratio: 10.21

Reference: Lebedev, 1986, p. 88

14 mm

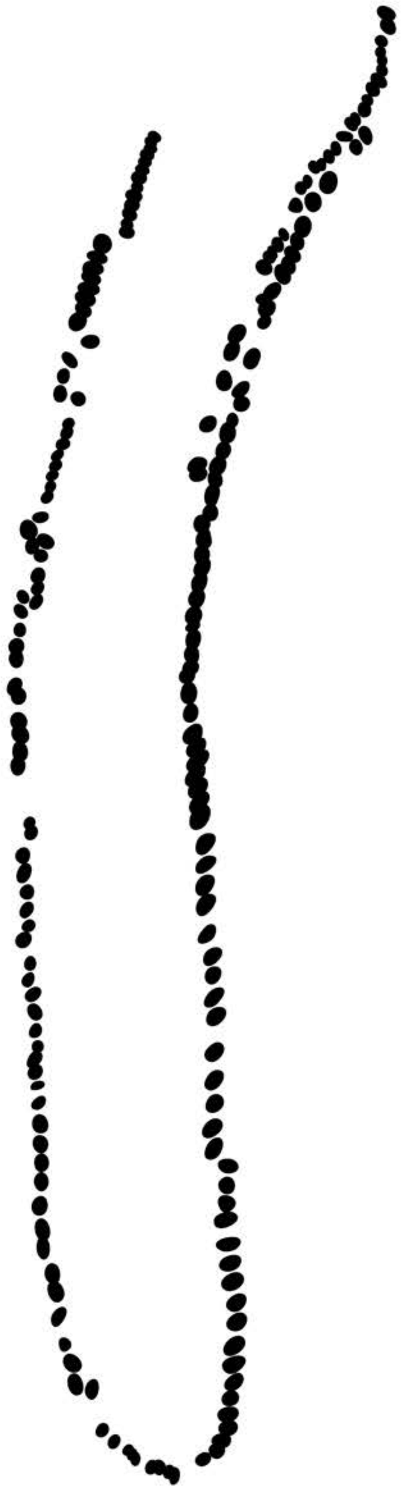

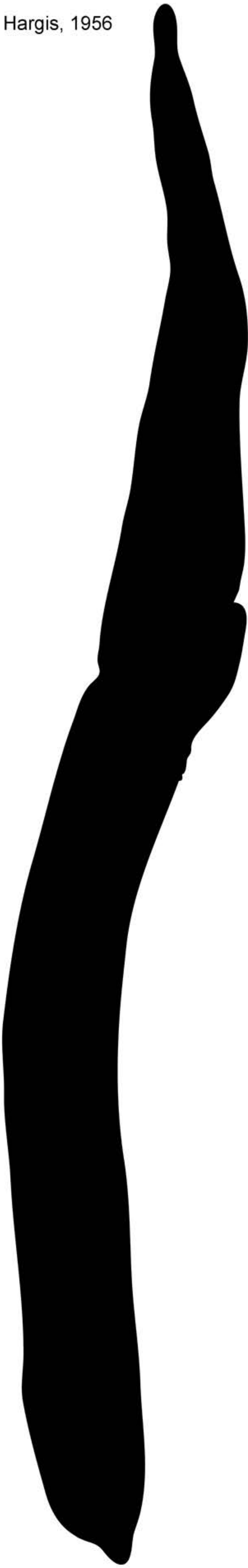

Family: Neothoracocotoylidae
Species:Paradawesia bychowskyi Bravo & Lamothe, 1976
Body Surface: 3,604,259
Clamp Surface: 390,338
Ratio: 10.83
Reference: Lebedev, 1986, p. 94

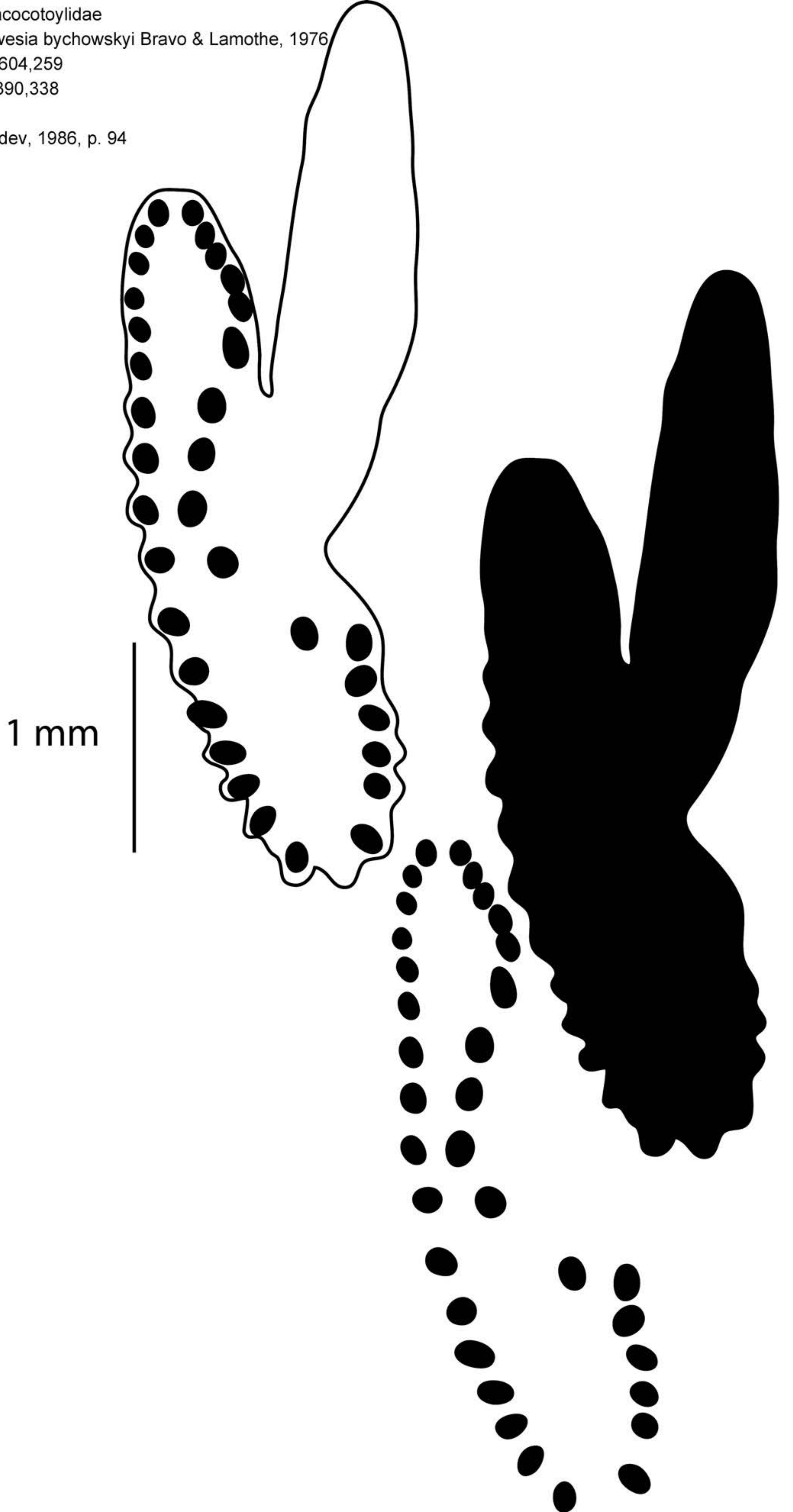

Family: Neothoracocotoylidae
Species: Pricea fotedari Gupta & Sharma, 1979
Body Surface: 2,219,395
Clamp Surface: 250,628
Ratio: 11.29
Reference: Pandey, 2008, p. 383

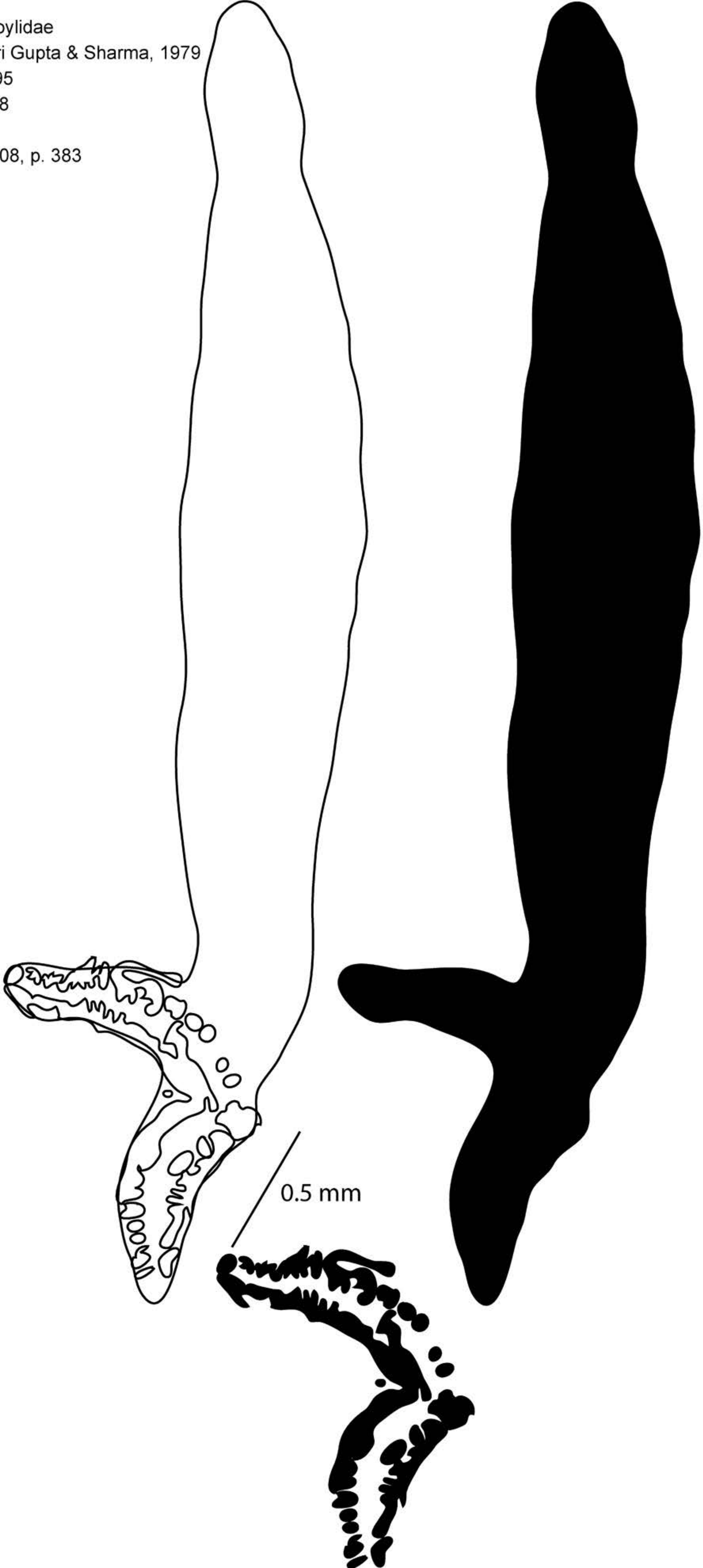

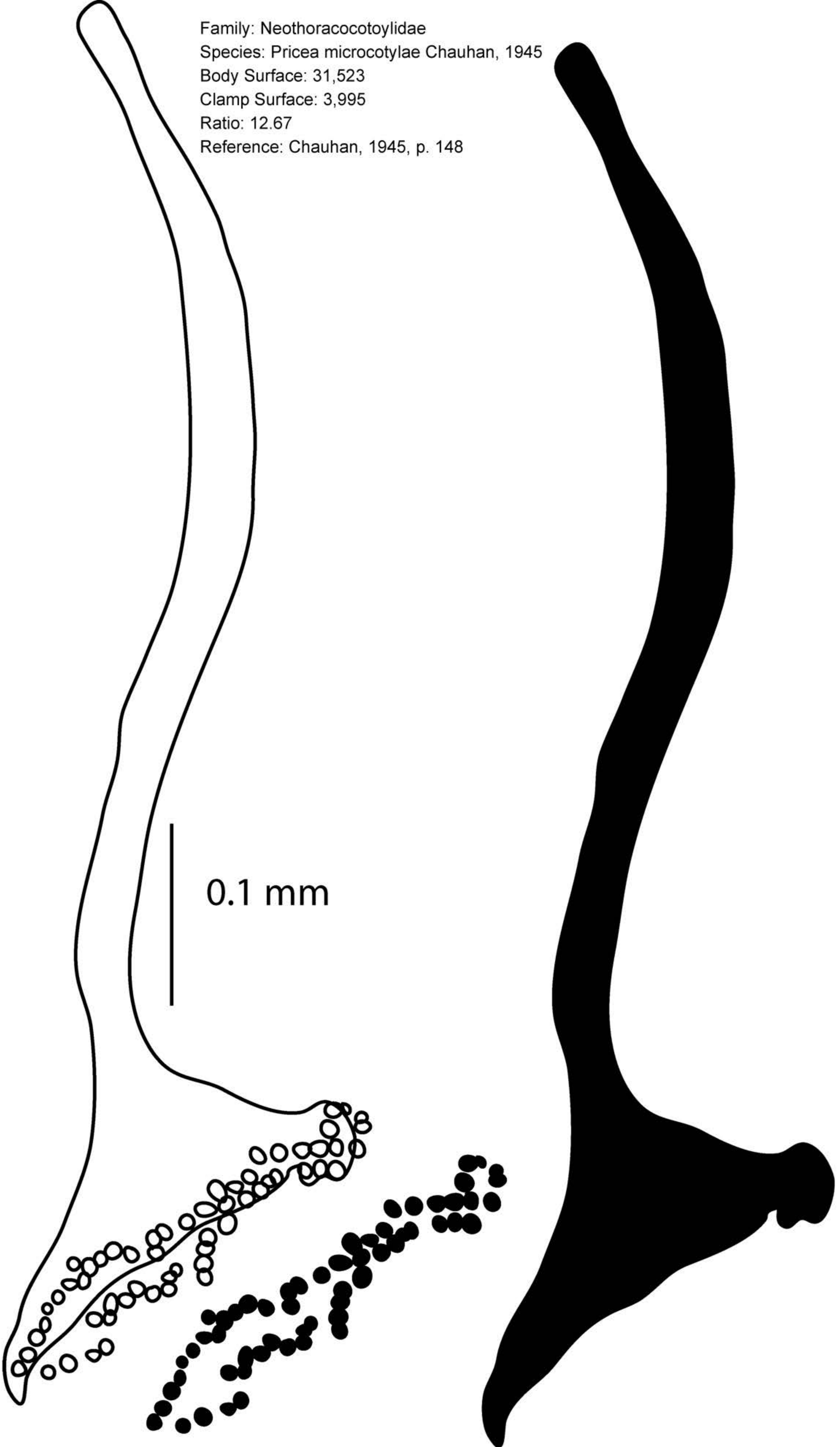
Family: Neothoracocotoylidae
Species: Pricea microcotylae Chauhan, 1945
Body Surface: 31,523
Clamp Surface: 3,995
Ratio: 12.67
Reference: Chauhan, 1945, p. 148
0.1 mm

Family: Neothoracocotoylidae
Species: Pricea minimae Chauhan, 1945
Body Surface: 796,183
Clamp Surface: 170,955
Ratio: 21.47
Reference: Chauhan, 1945, p. 146

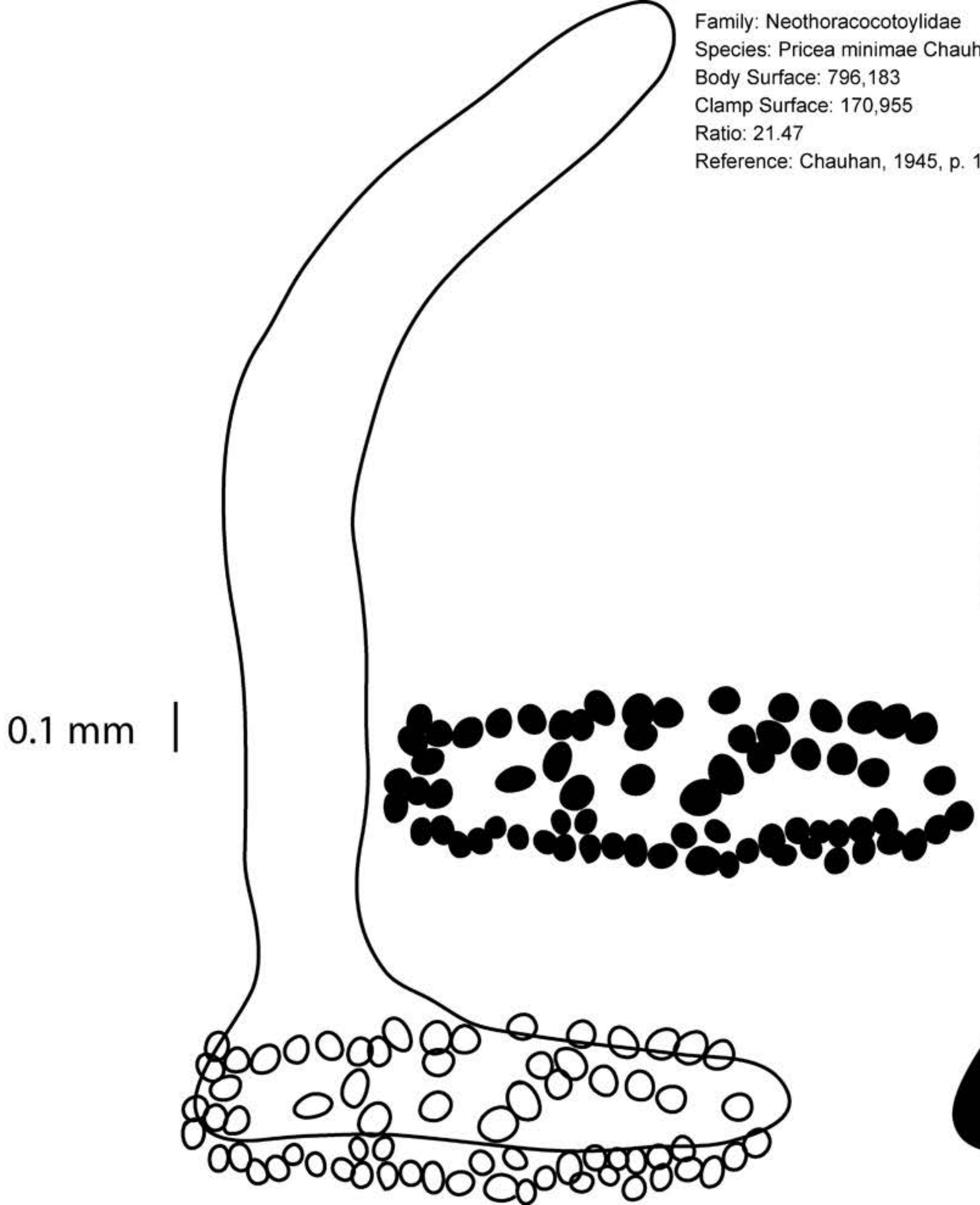

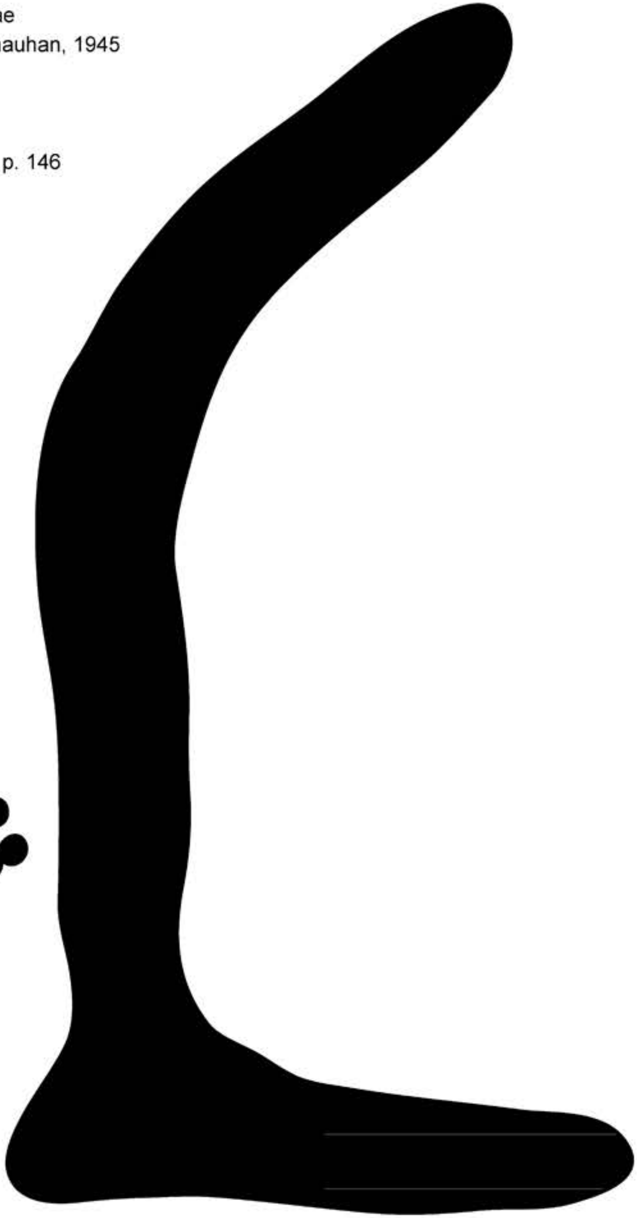

Family: Neothoracocotoylidae
Species: Pricea multae Chauhan, 1945
Body Surface: 3,561,945
clamp Surface: 371,996
Ratio: 10.44
Reference: Rohde, 1999, p. 173

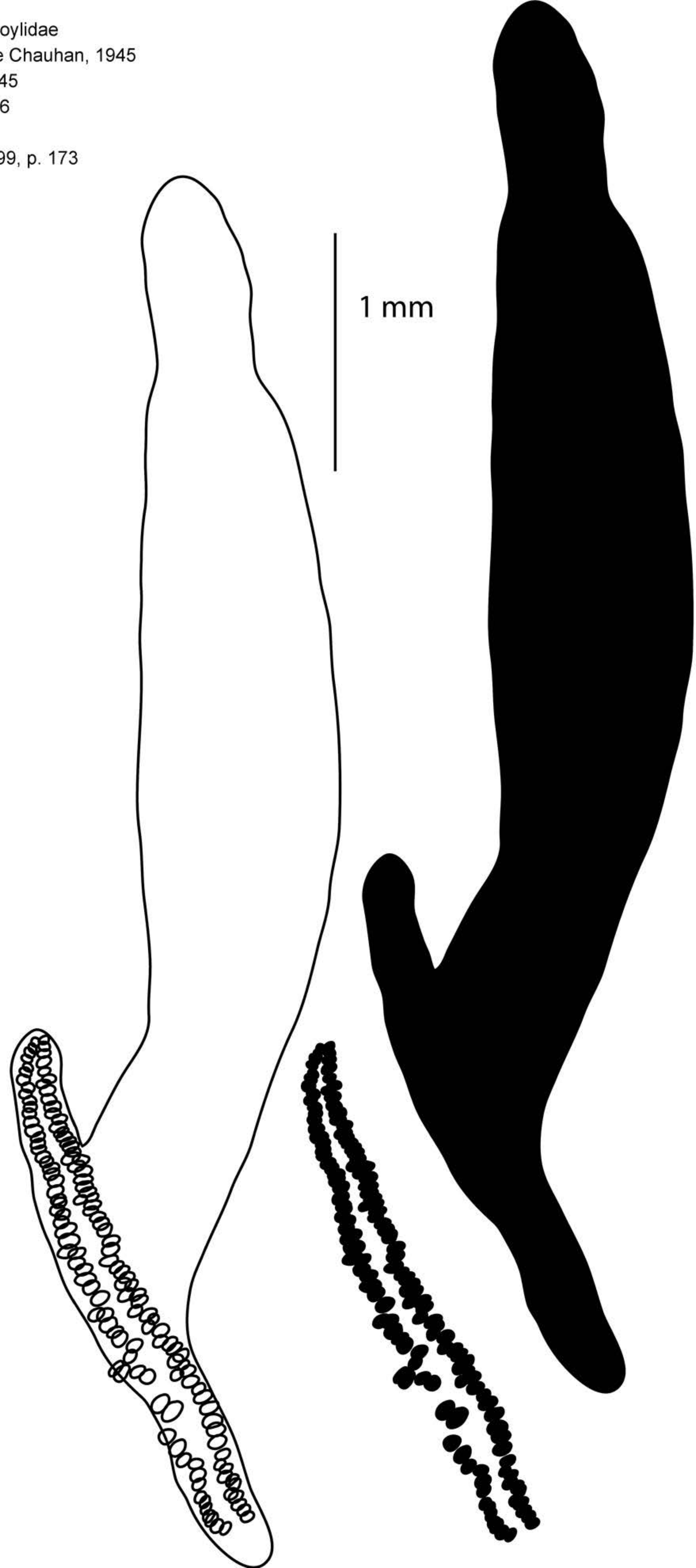

Family: Neothoracocotoylidae
Species: Pricea solandri Gupta & Channa, 1977
Body Surface: 29,180
Clamp Surface: 4,158
Ratio: 14.25
Reference: Pandey, 2008, p. 382

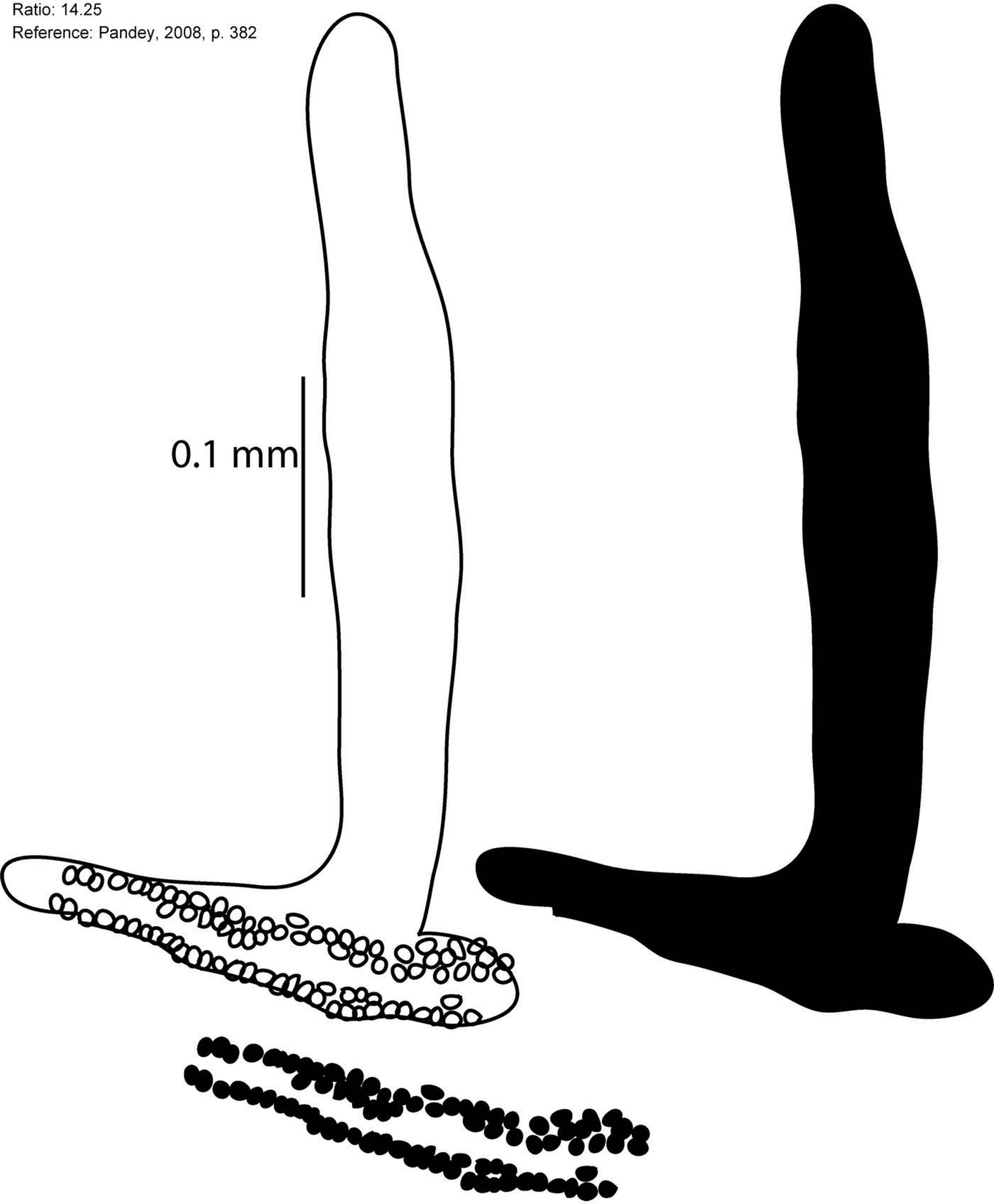

Family: Neothoracocotoylidae
Species: Pseudothoracocotyla ovalis
(Tripathi, 1956) Yamaguti, 1963
Body Surface: 1,104,406
Clamp Surface: 122,155
Ratio: 11.06
Reference: Hayward, 1999, p. 164

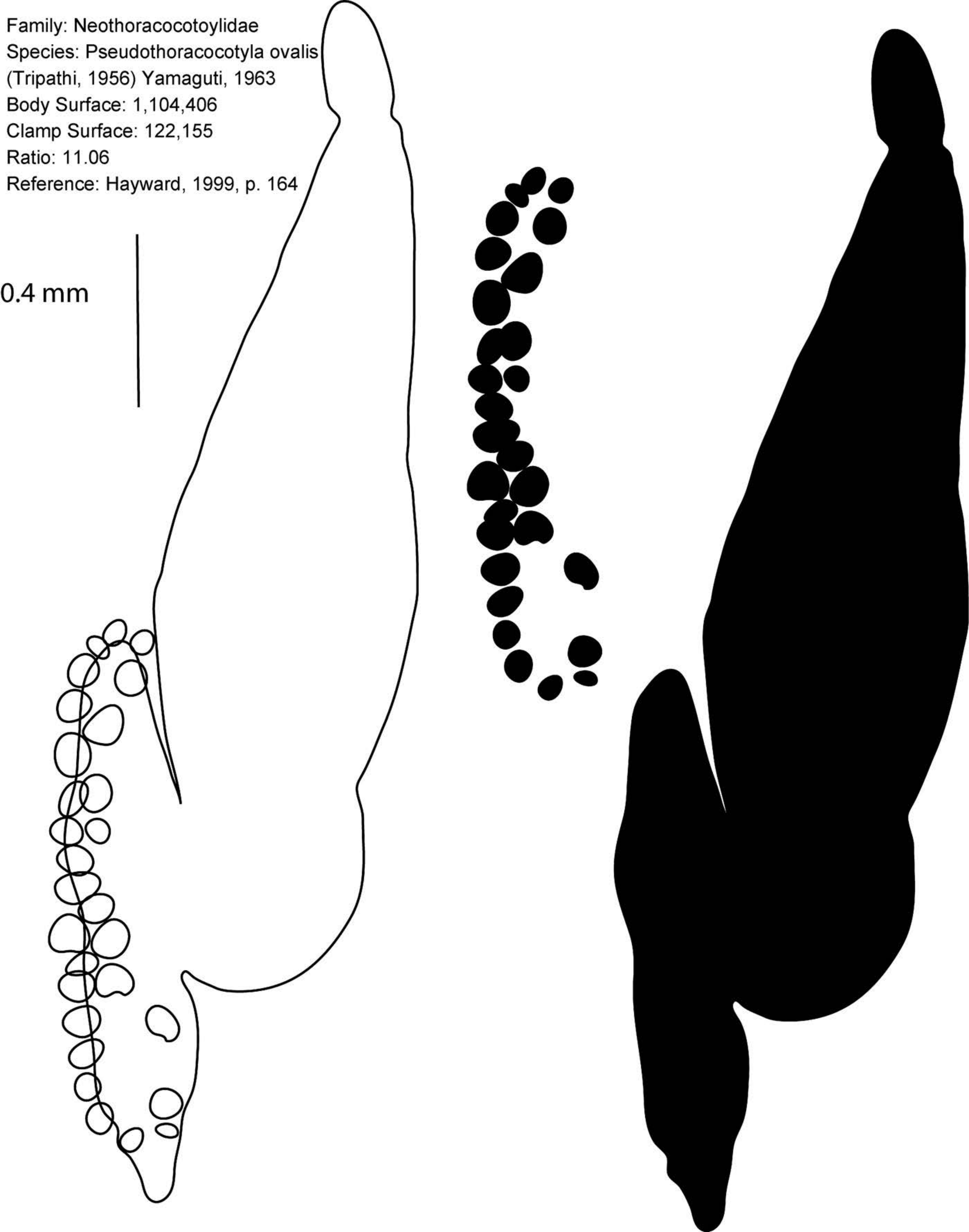

Family: Neothoracocotoylidae
Species: Pseudothoracocotyla whittingtoni Hayward & Rohde, 1999
Body Surface: 6,151,442
Clamp Surface: 2,086,478
Ratio: 33.92
Reference: Hayward, 1999, p. 167

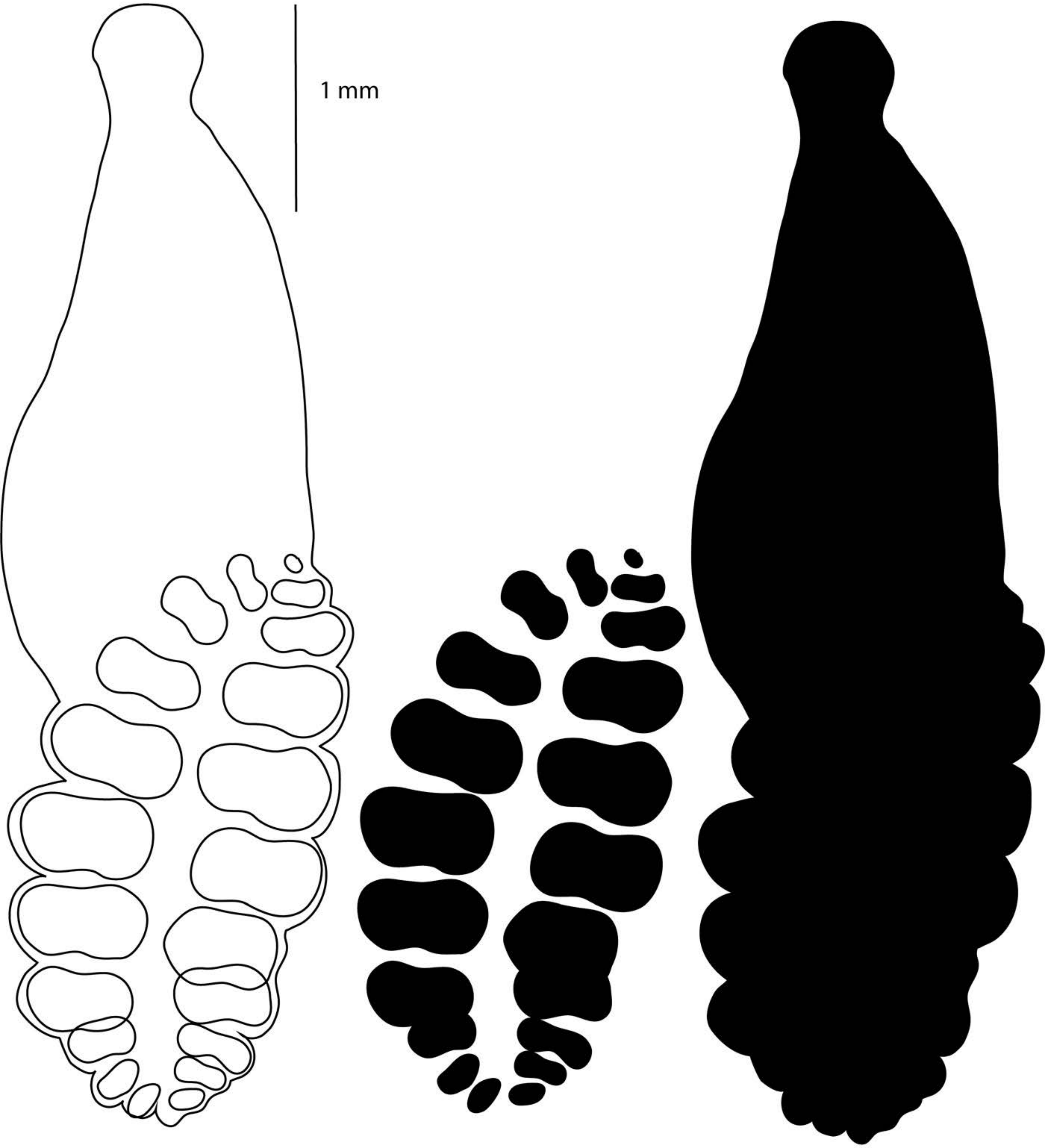

Family: Neothoracocotoylidae
Species: Scomberocotyle scomberomori (Koratha, 1955) Hargis, 1956
Body surface: 2837224
Clamp surface: 293771
Ratio: 10,35
Reference: Lebedev, 1986, p 89

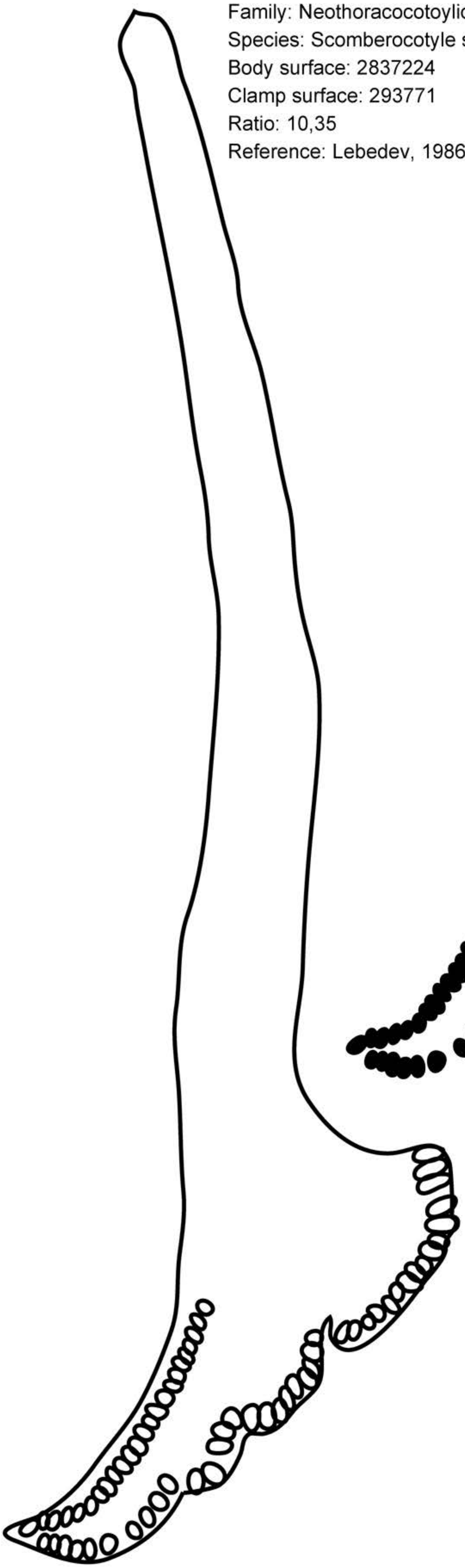

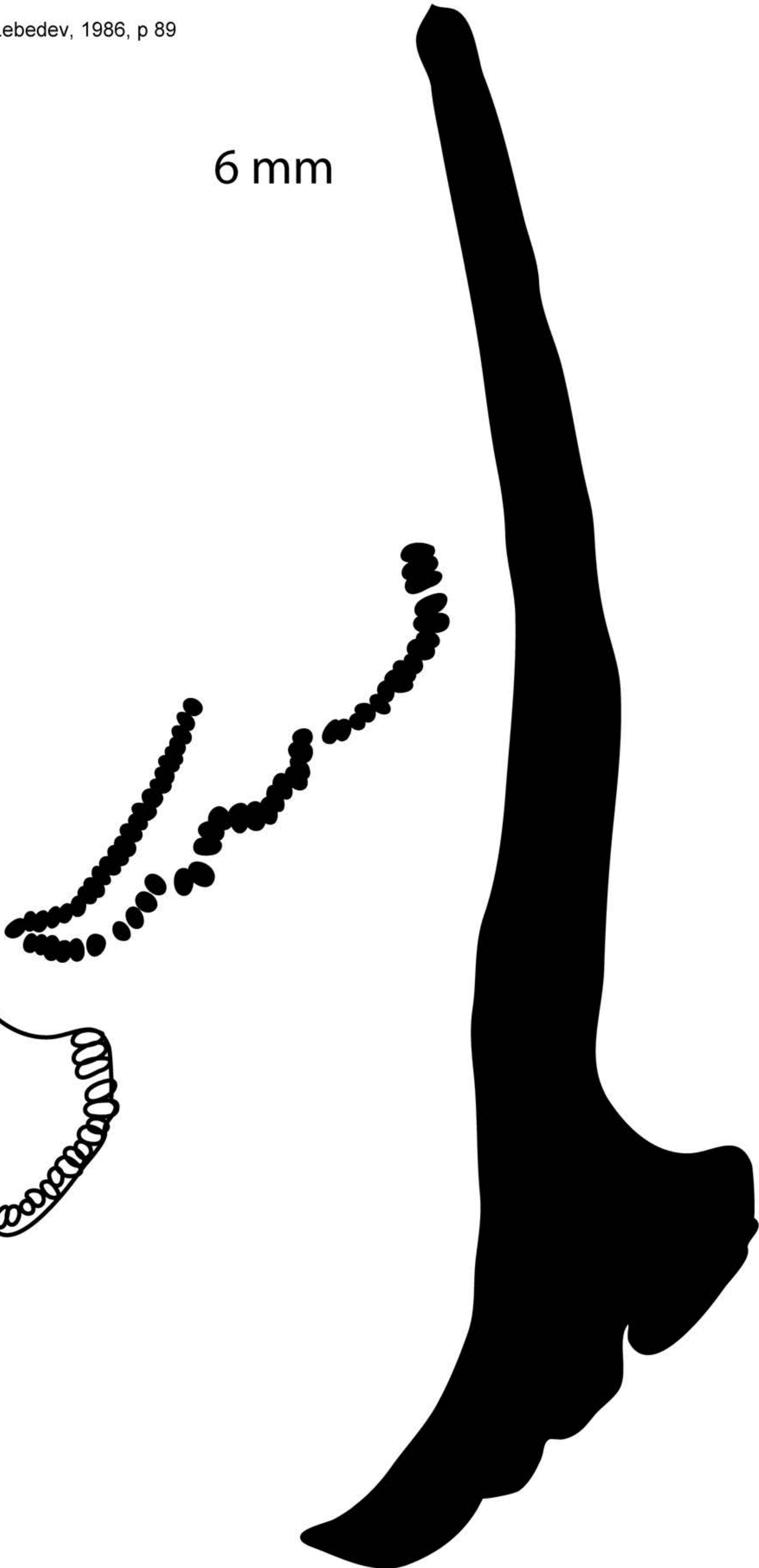

Family: Neothoracocotylidae
Species: Scomberomorocotyle munroi Rohde & Hayward, 1999
Body Surface: 640,210
Clamp Surface: 74,389
Ratio: 11.62
Reference: Rohde, 1999, p. 5

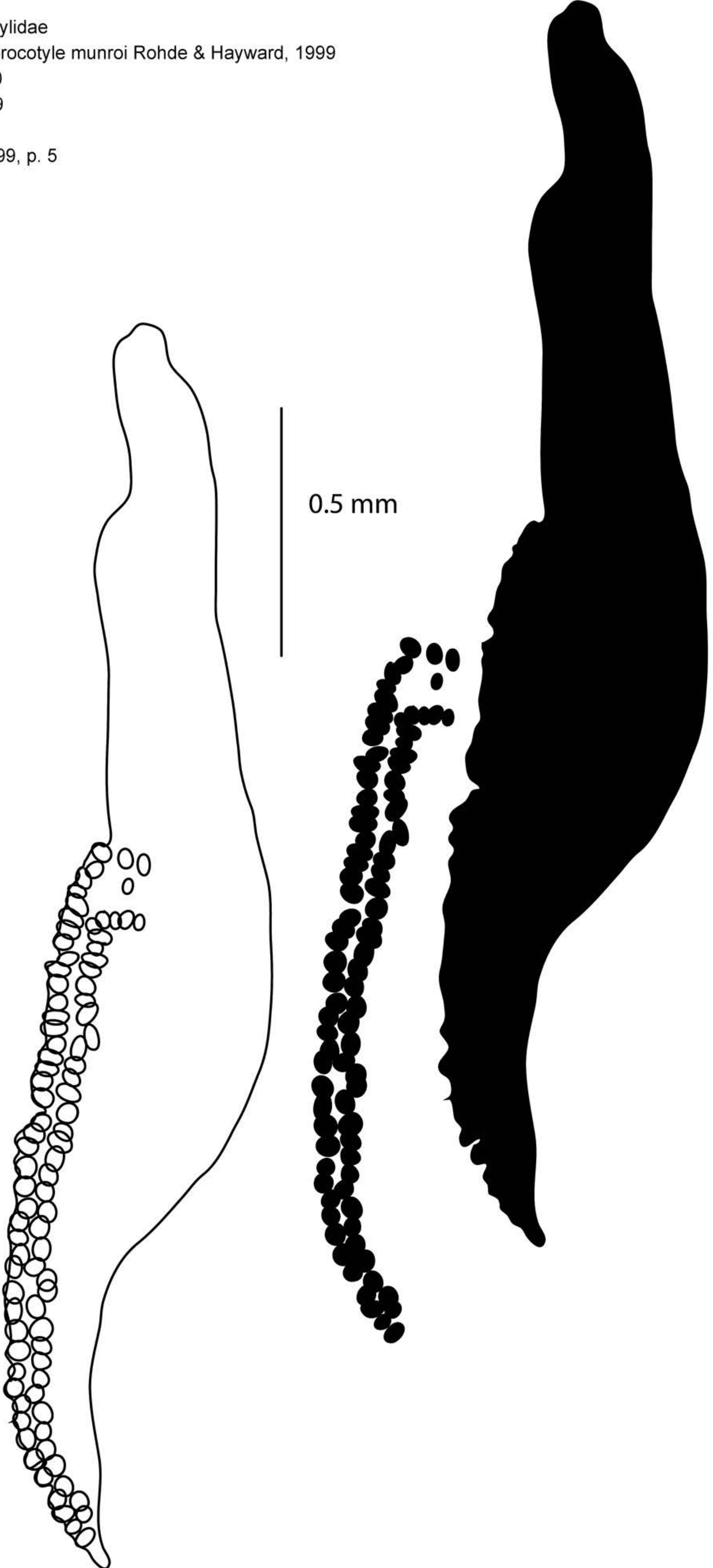

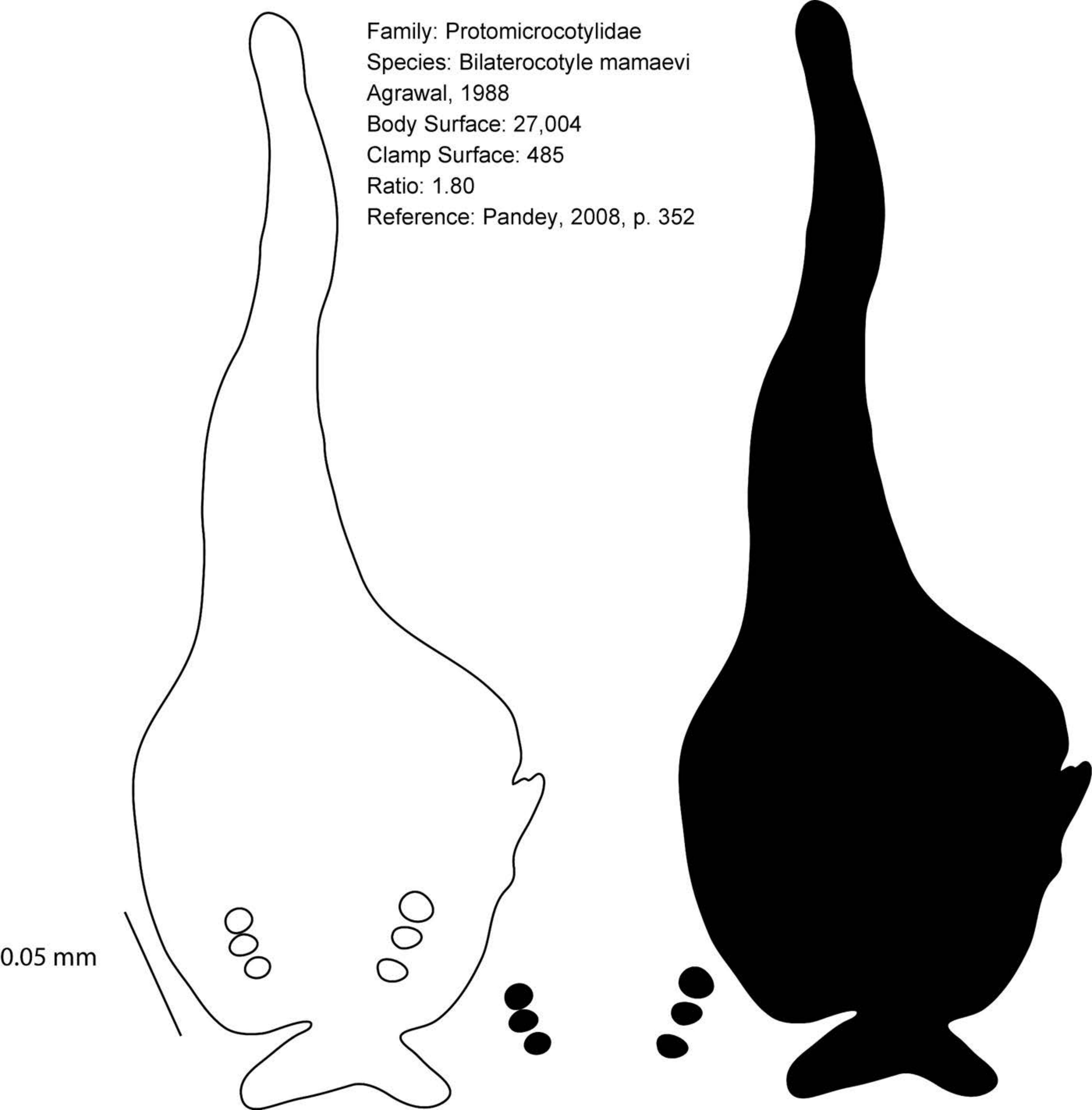

Family: Protomicrocotylidae
Species: Bilaterocotyle mamaevi
Agrawal, 1988
Body Surface: 27,004
Clamp Surface: 485
Ratio: 1.80
Reference: Pandey, 2008, p. 352
0.05 mm

Family: Protomicrocotylidae
Species: *Bilaterocotyle chirocentrosus* Chauhan, 1945
Body Surface: 670,131
Clamp Surface: 15,726
Ratio: 2.35
Reference: Chauhan, 1945, p. 138

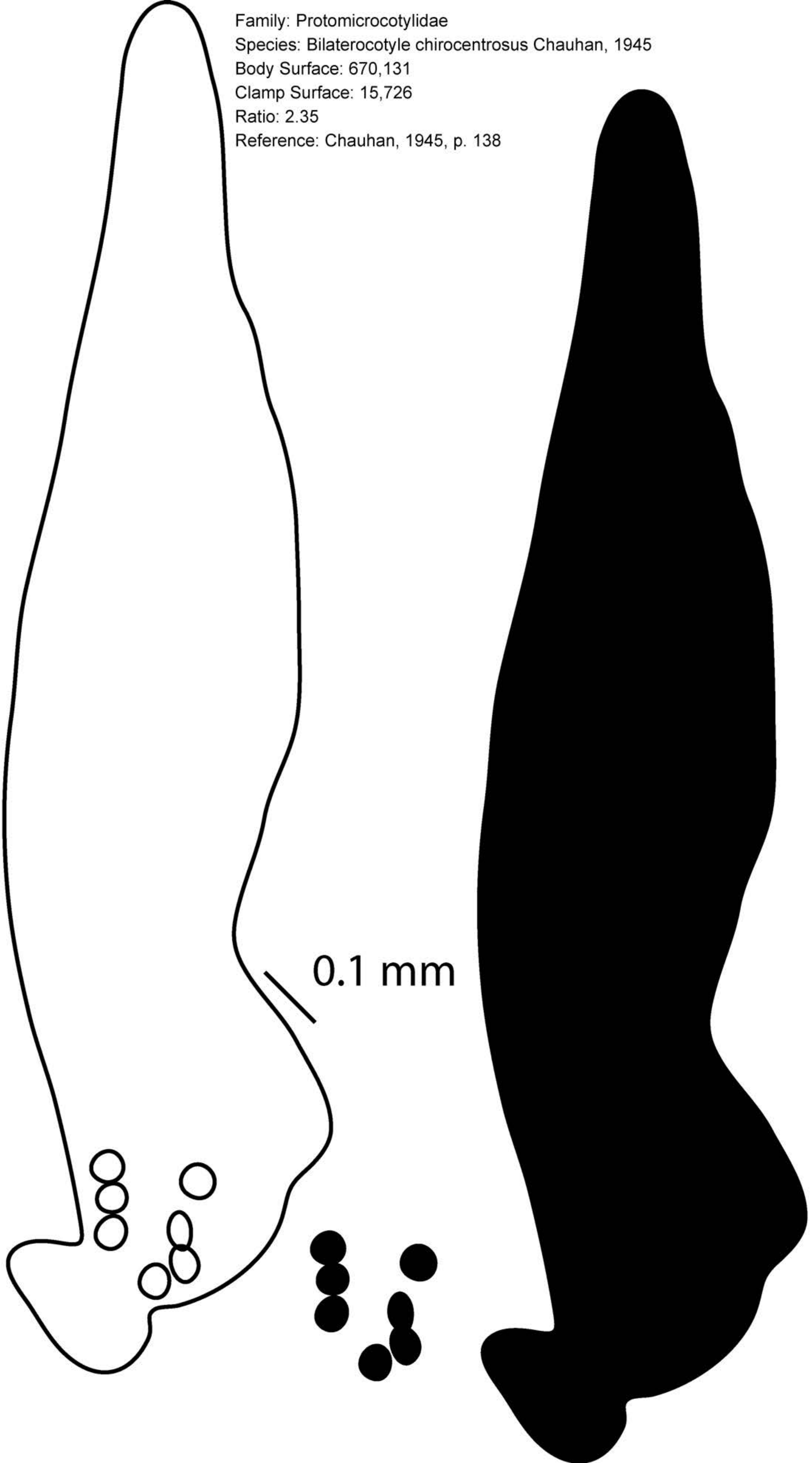

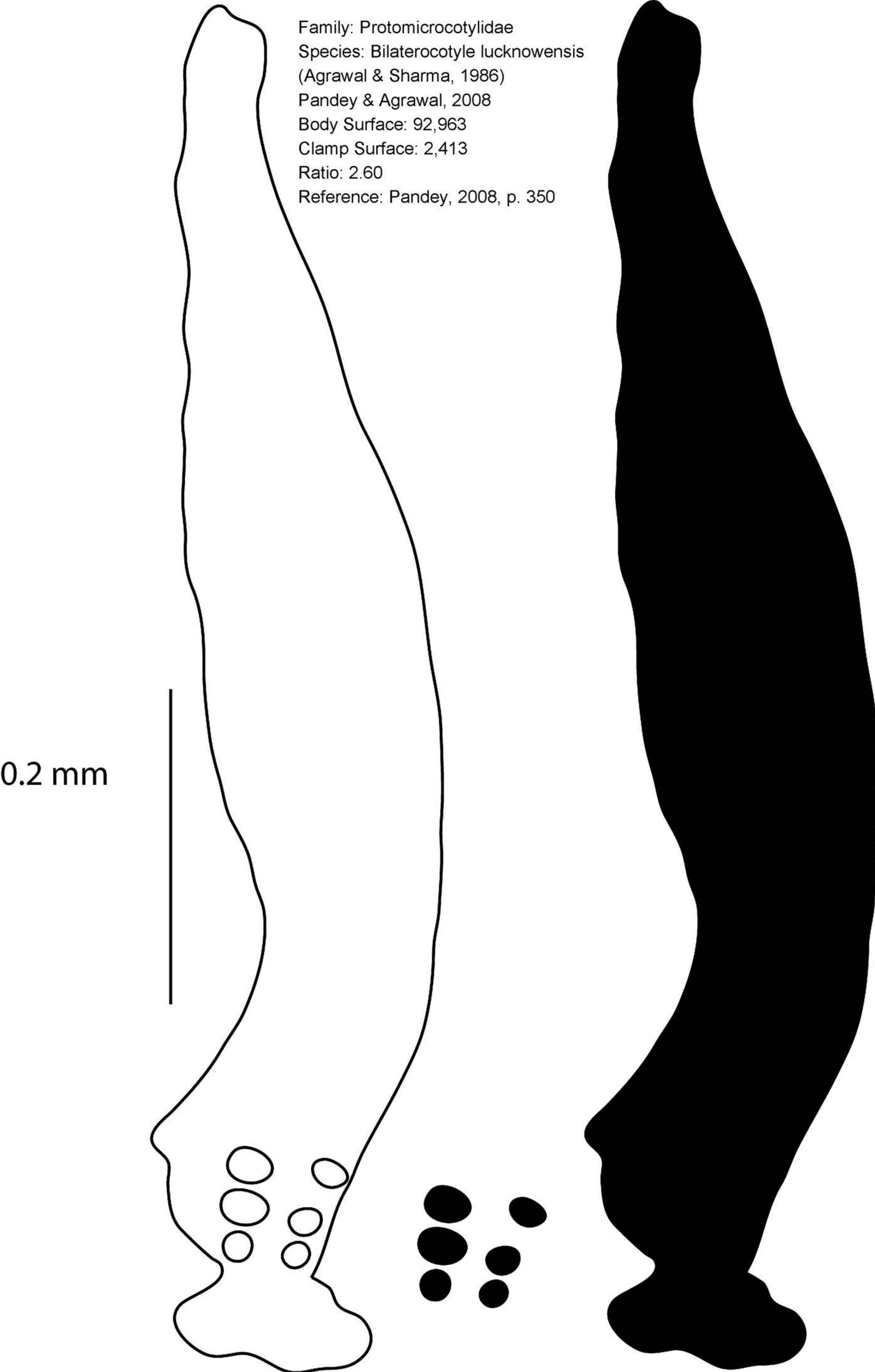
Family: Protomicrocotylidae
Species: Bilaterocotyle lucknowensis
(Agrawal & Sharma, 1986)
Pandey & Agrawal, 2008
Body Surface: 92,963
Clamp Surface: 2,413
Ratio: 2.60
Reference: Pandey, 2008, p. 350
0.2 mm

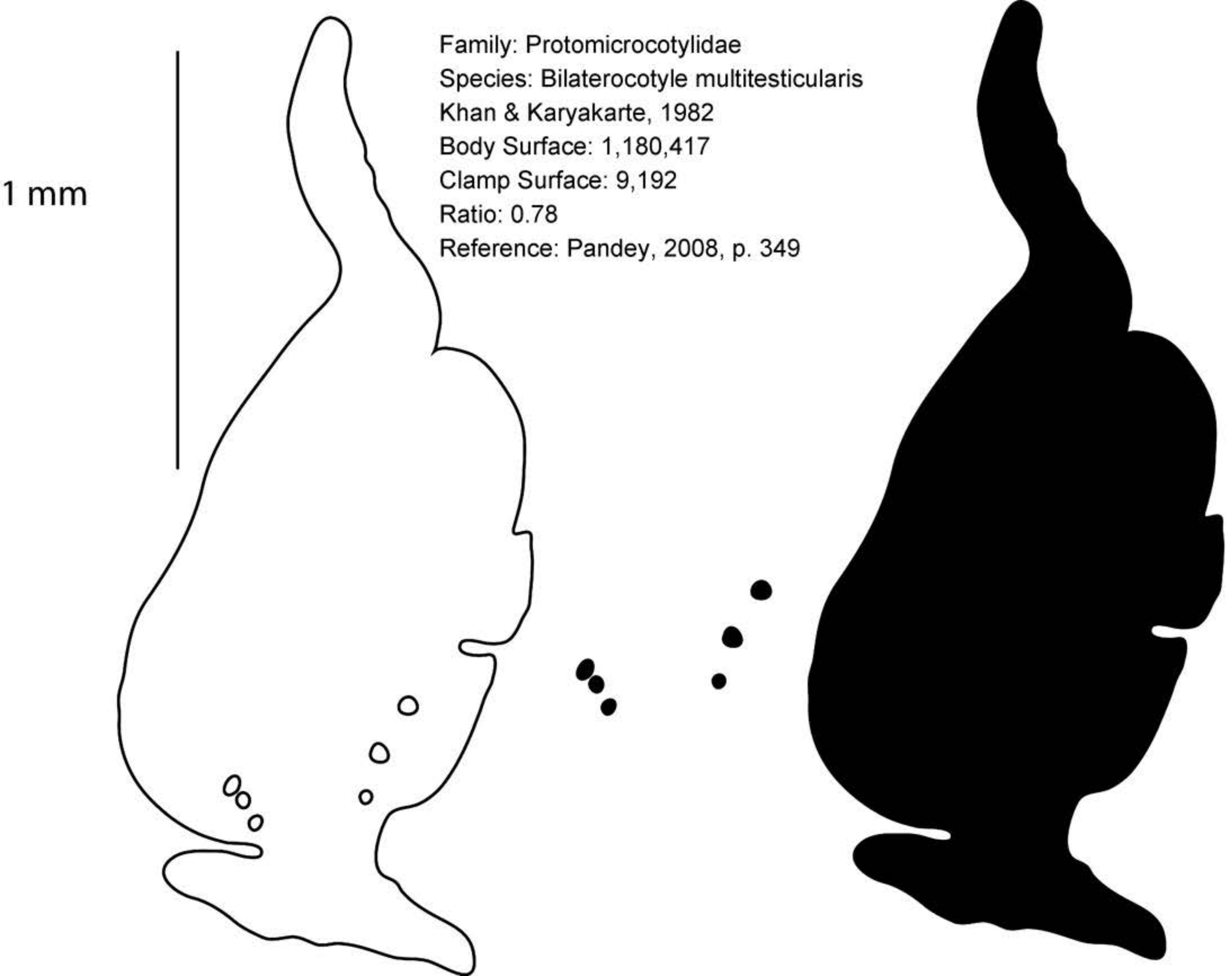
1 mm
Family: Protomicrocotylidae
Species: Bilaterocotyle multitesticularis
Khan & Karyakarte, 1982
Body Surface: 1,180,417
Clamp Surface: 9,192
Ratio: 0.78
Reference: Pandey, 2008, p. 349

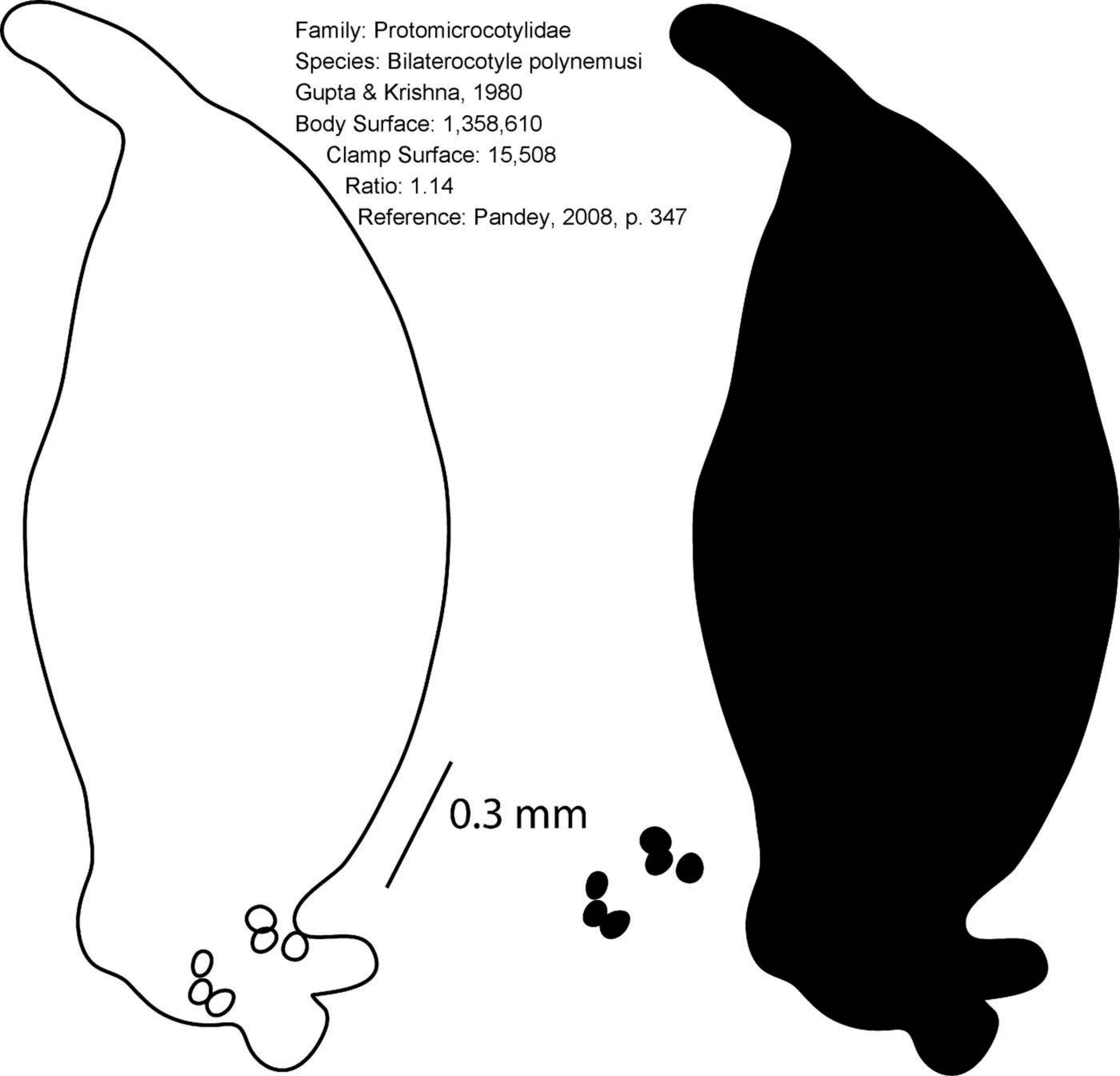

Family: Protomicrocotylidae
Species: Bilaterocotyle polynemusi
Gupta & Krishna, 1980
Body Surface: 1,358,610
Clamp Surface: 15,508
Ratio: 1.14
Reference: Pandey, 2008, p. 347
0.3 mm

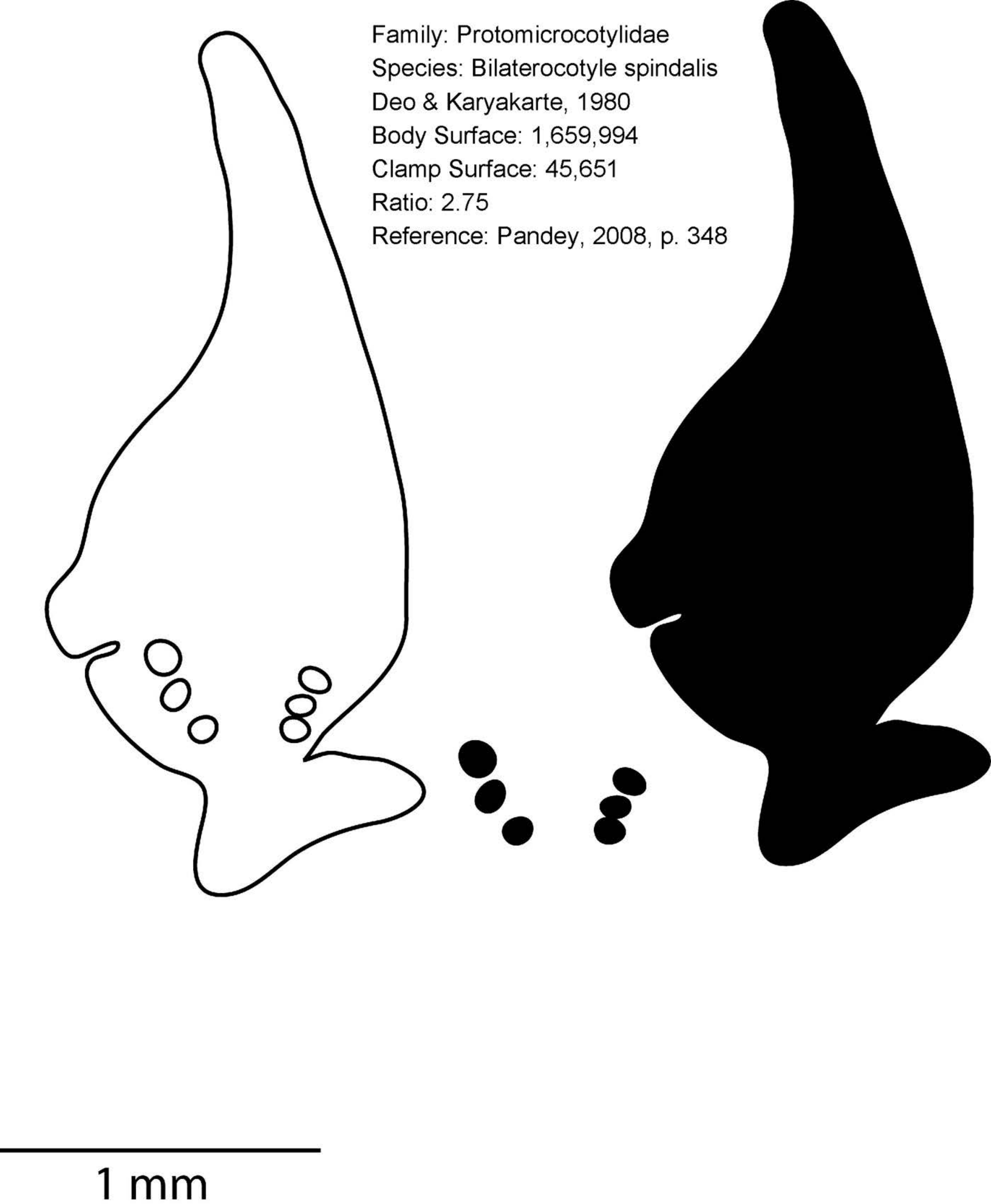
Family: Protomicrocotylidae
Species: Bilaterocotyle spindalis
Deo & Karyakarte, 1980
Body Surface: 1,659,994
Clamp Surface: 45,651
Ratio: 2.75
Reference: Pandey, 2008, p. 348
1 mm

Family: Protomicrocotylidae
Species: Bilaterocotyloides carangis Ramalingam, 1961
Body Surface: 1,135,204
Clamp Surface: 13,434
Ratio: 1.18
Reference: Lebedev, 1986, p. 114

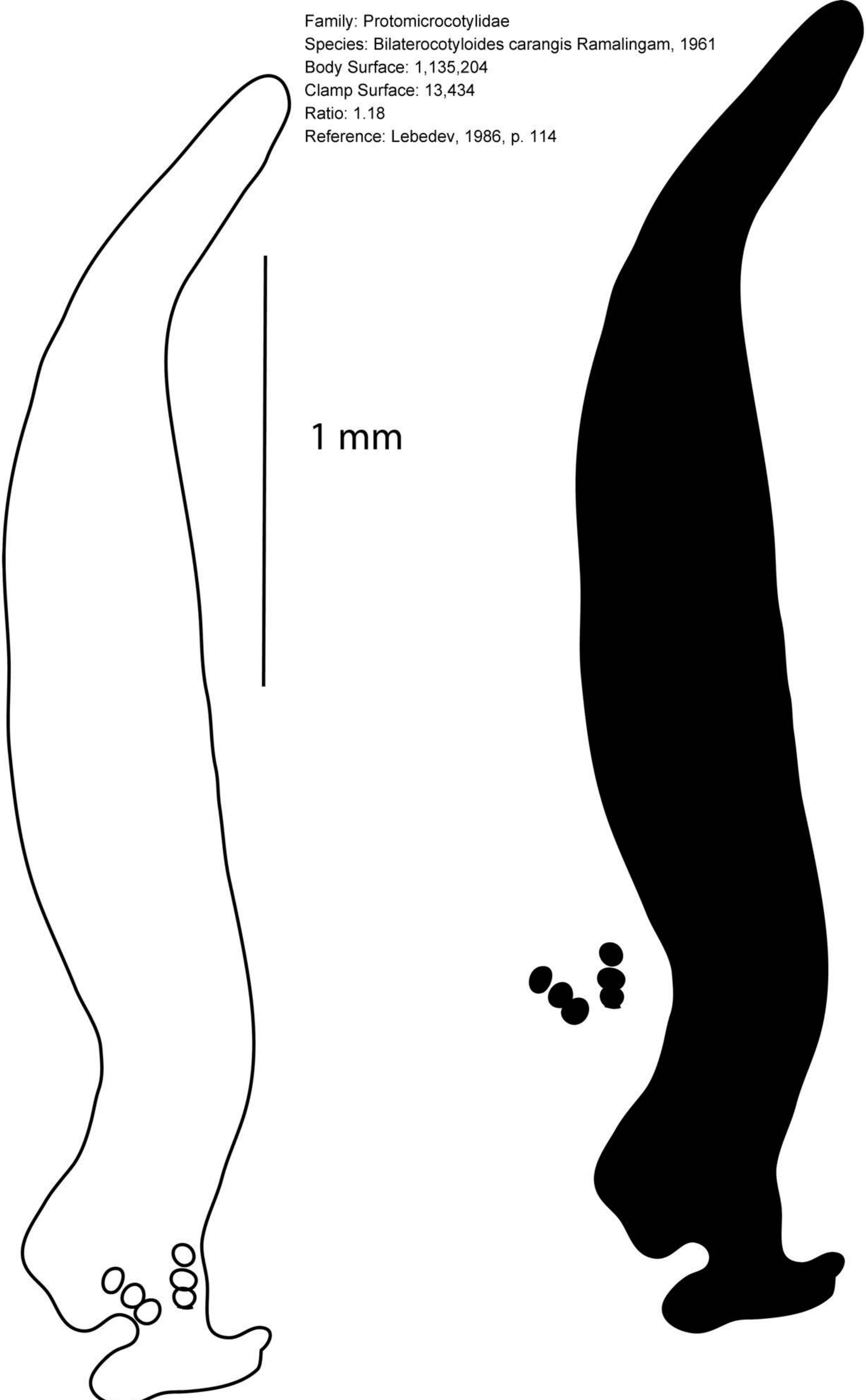

Family: Protomicrocotylidae
Species: Bilaterocotyloides madrasensis Radha, 1966
Body Surface: 441,228
Clamp Surface: 4,984
Ratio: 1.13
Reference: Lebedev, 1986, p. 116

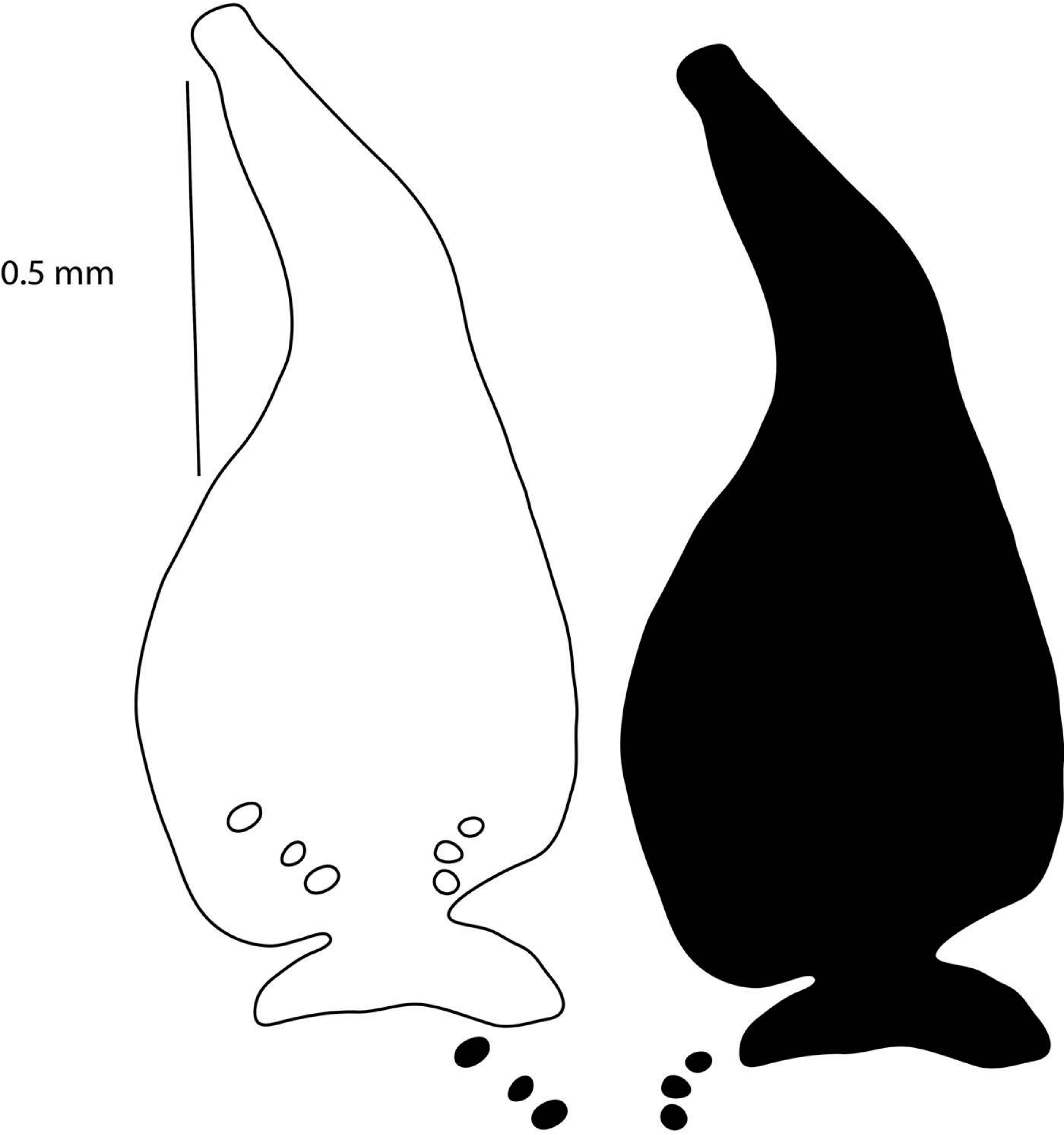

Family: Protomicrocotylidae

Species: Bilaterocotyloides novaeguineae (Rohde, 1977) Lebedev, 1986

Body Surface: 442,980

Clamp Surface: 11,411

Ratio: 2.58

Reference: Lebedev, 1986, p. 114

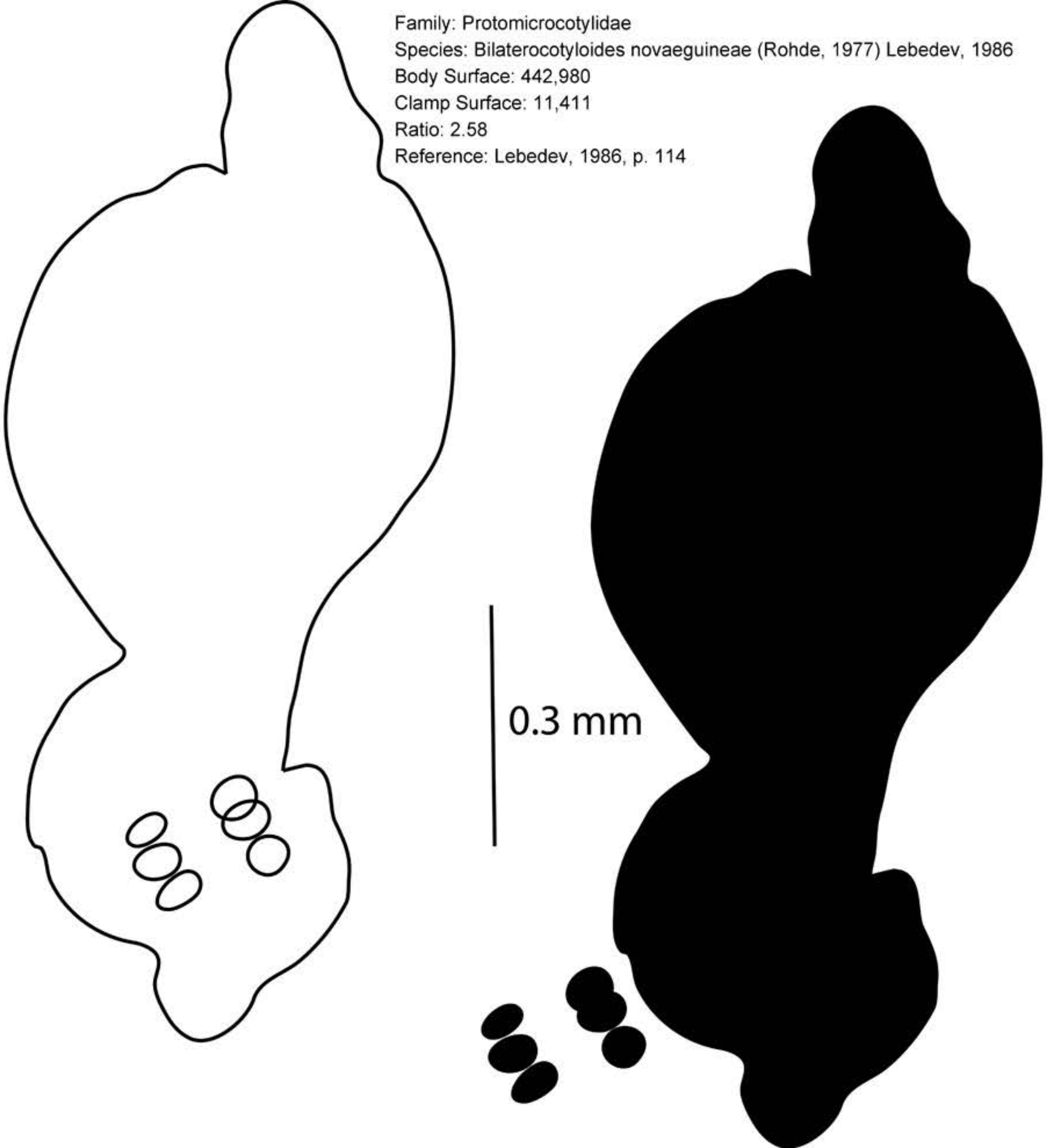

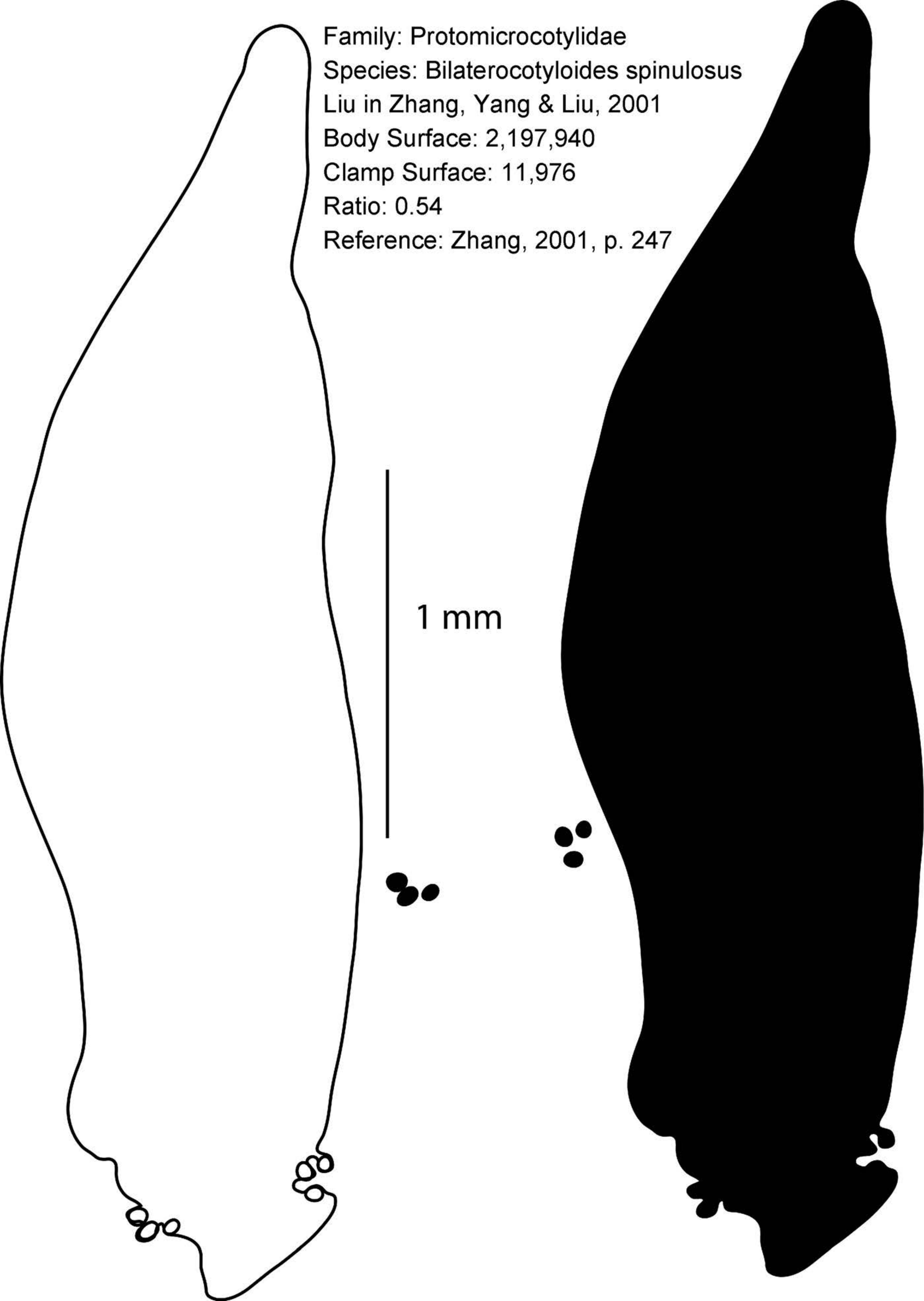
Family: Protomicrocotylidae
Species: Bilaterocotyloides spinulosus
Liu in Zhang, Yang & Liu, 2001
Body Surface: 2,197,940
Clamp Surface: 11,976
Ratio: 0.54
Reference: Zhang, 2001, p. 247
1 mm

Family: Protomicrocotylidae
Speceis: *Chauhanocotyle rottleri*
Khoche & Dad, 1975
Body Surface: 912,981
Clamp Surface: 13,257
Ratio: 1.45
Reference: Pandey, 2008, p. 356

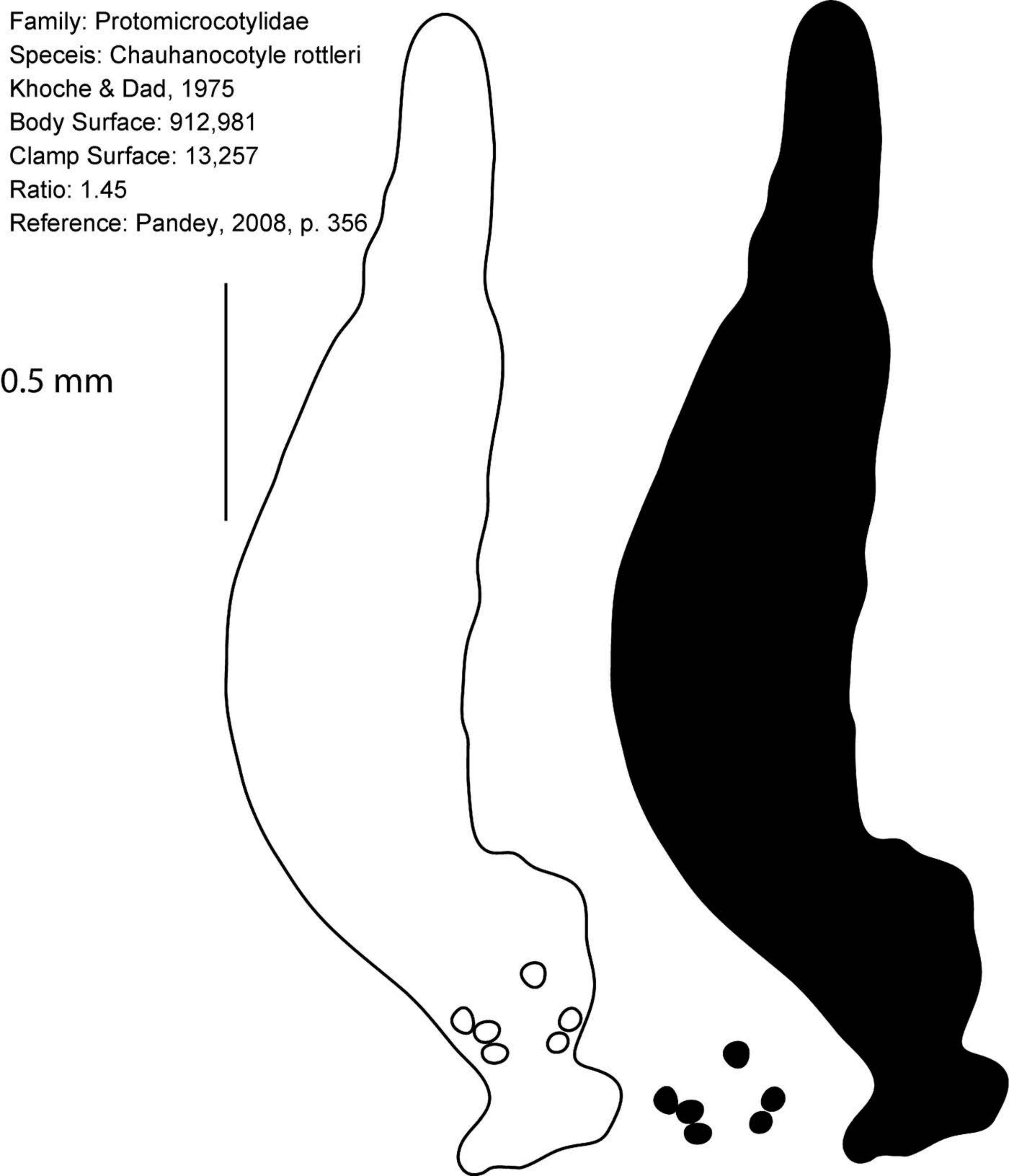

Family: Protomicrocotylidae
Species: Lethacotyle fijiensis Manter & Price, 1953
Body Surface: 2,788,607
Clamp Surface: 0
Ratio: 0
Reference: Lebedev, 1986, p. 117

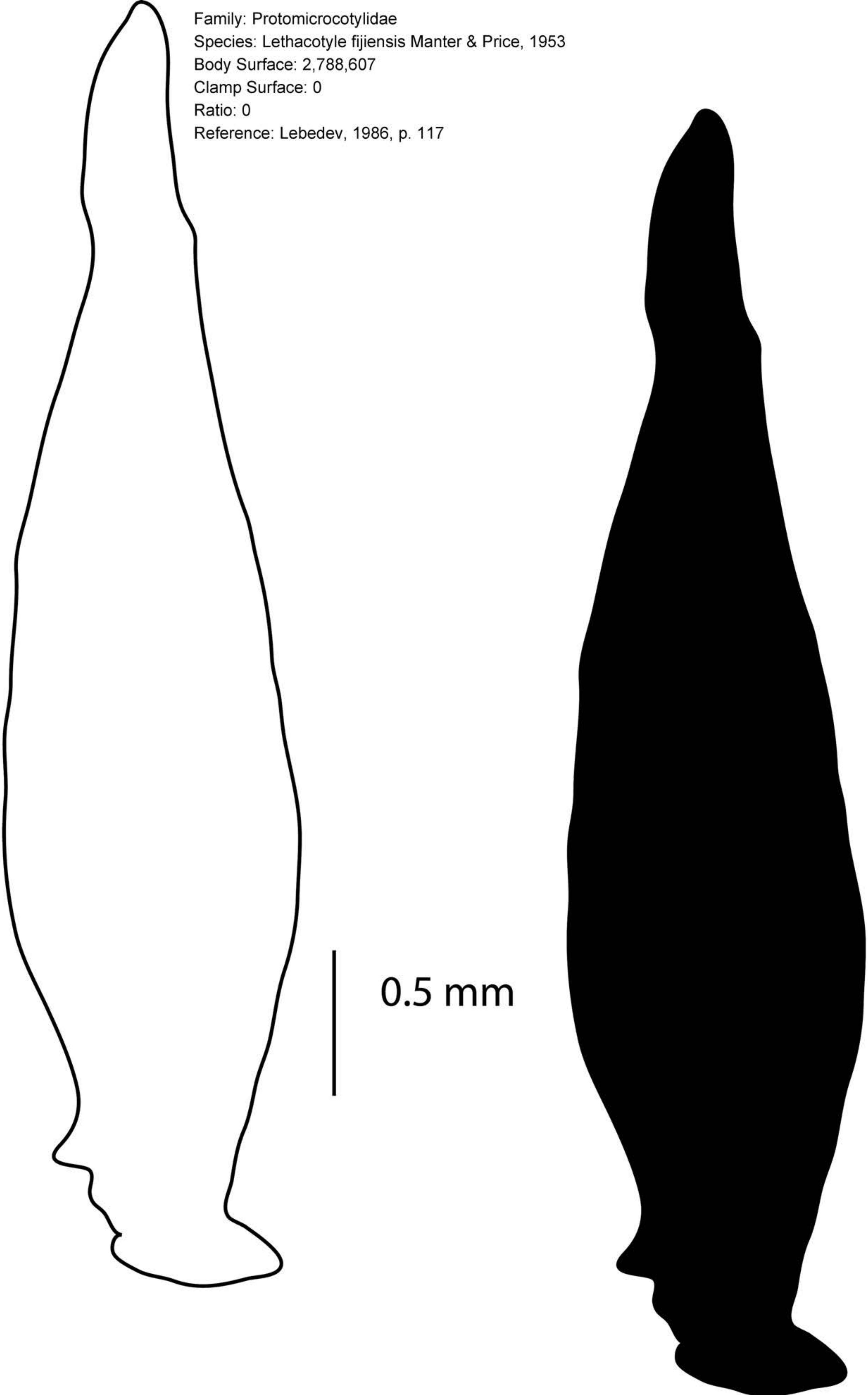

Family: Protomicrocotylidae
Species: Lethacotyle new species JNC3209A1
Body Surface: 2,562,639
Clamp Surface: 0
Ratio: 0

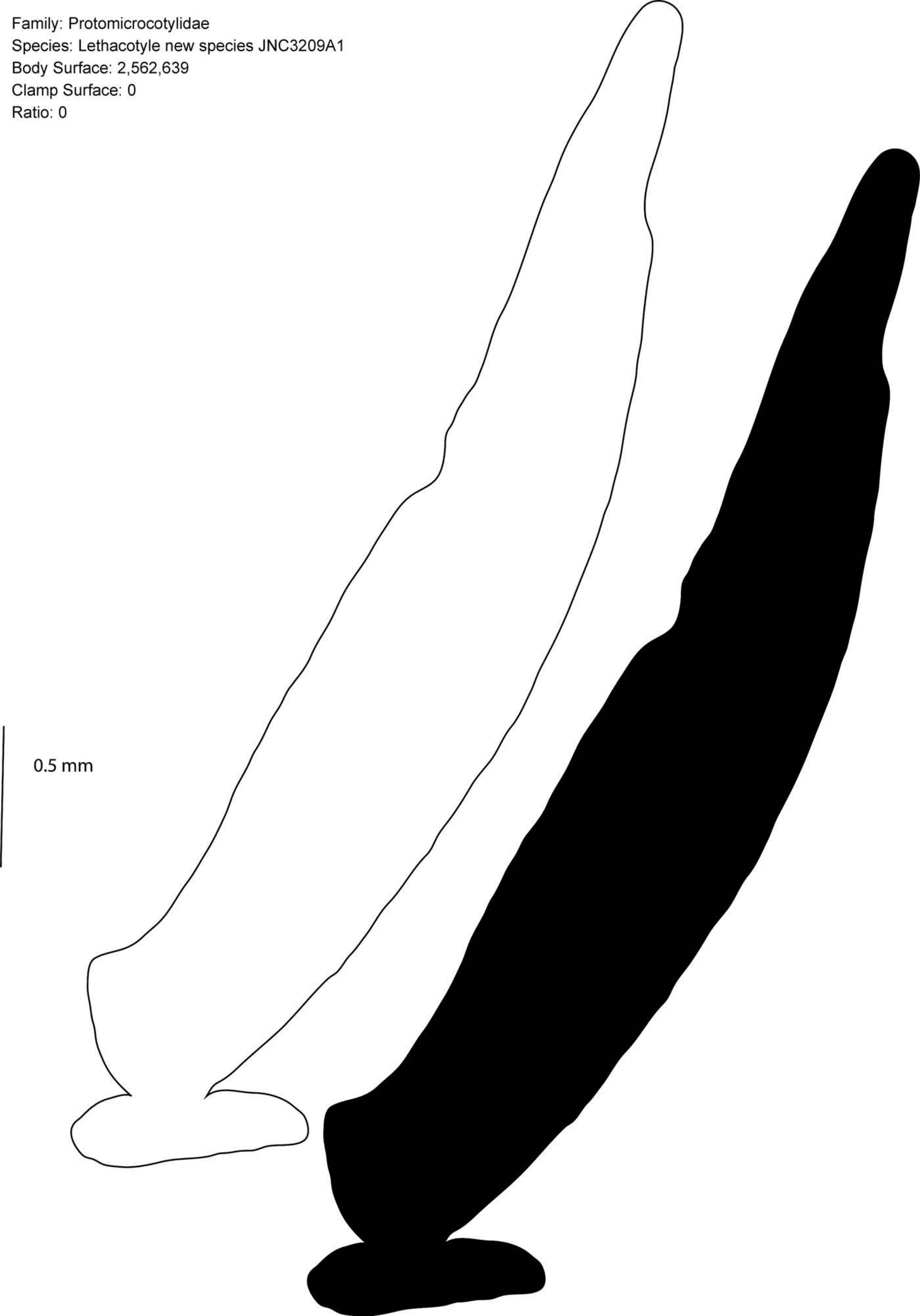

Family: Protomicrocotylidae

Species: *Neomicrocotyle carangis* Yamaguti, 1968

Body Surface: 4,287,184

Clamp Surface: 15,571

Ratio: 0.36

Reference: Lebedev, 1986, p. 110

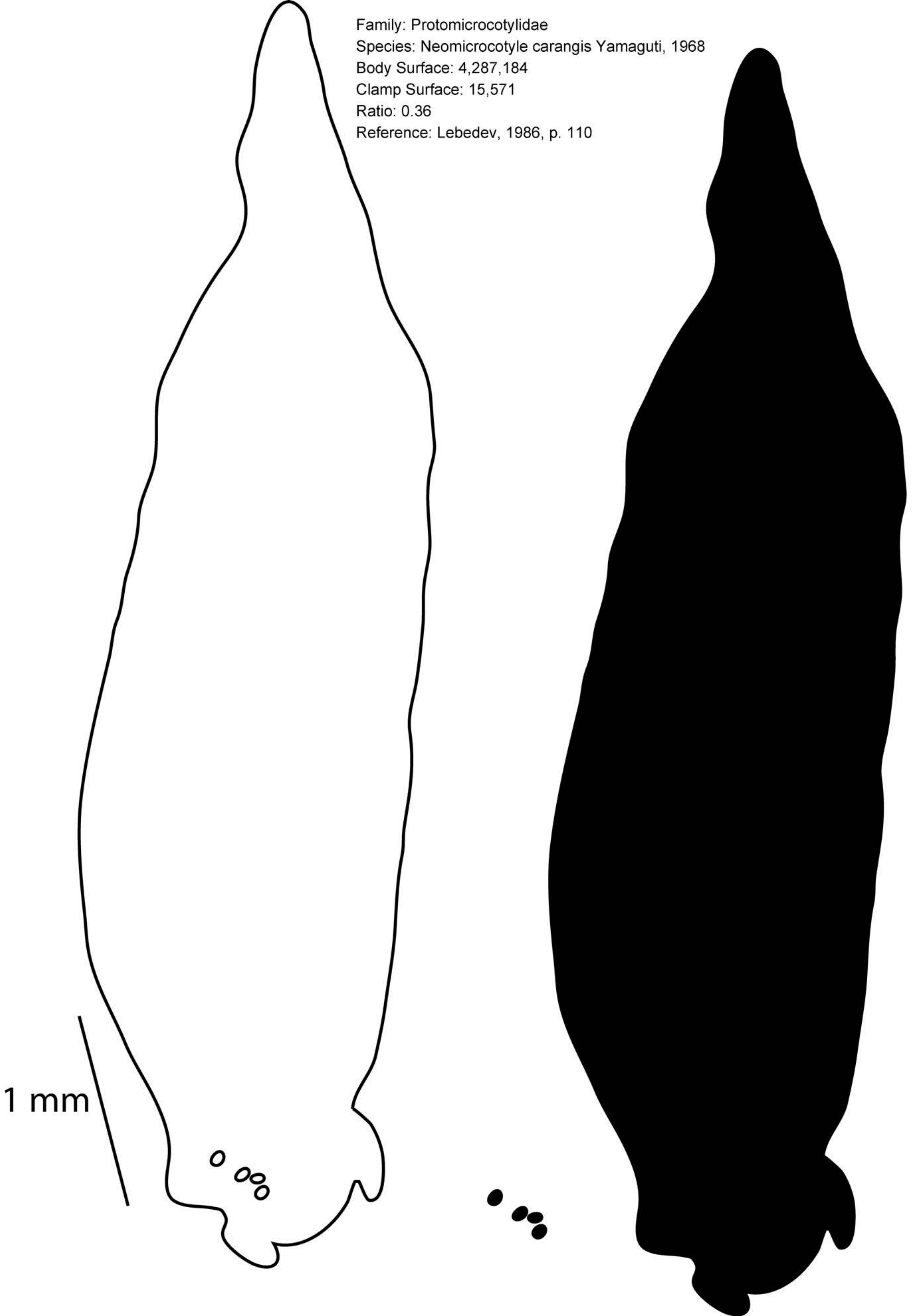

Family: Protomicrocotylidae
Species: Neomicrocotyle indica Ramalingam, 1960
Body Surface: 49,651
Clamp Surface: 232
Ratio: 0.47
Reference: Ramalingam, 1960, p. 375

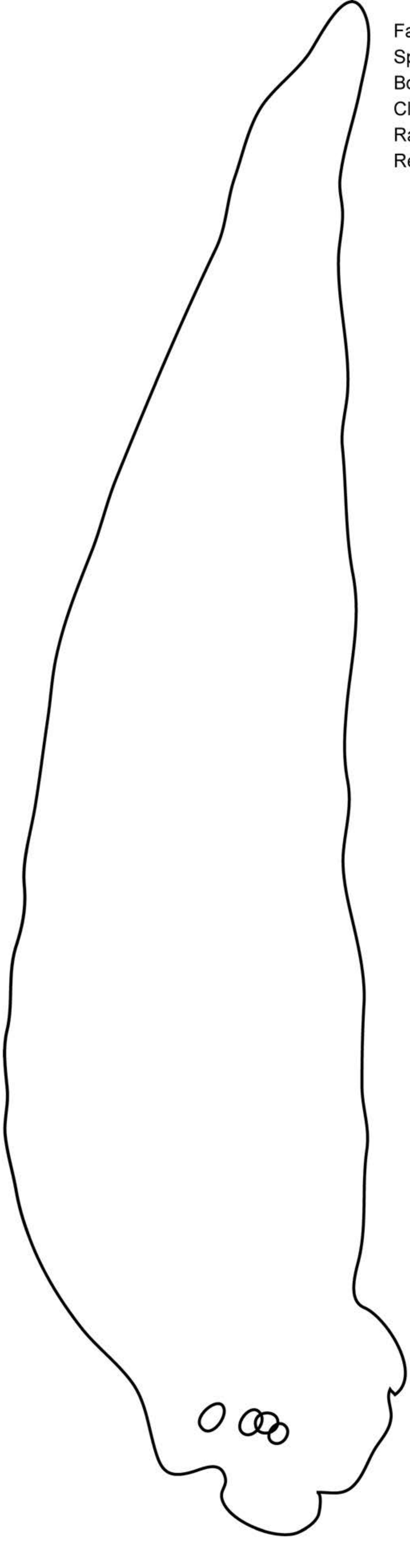

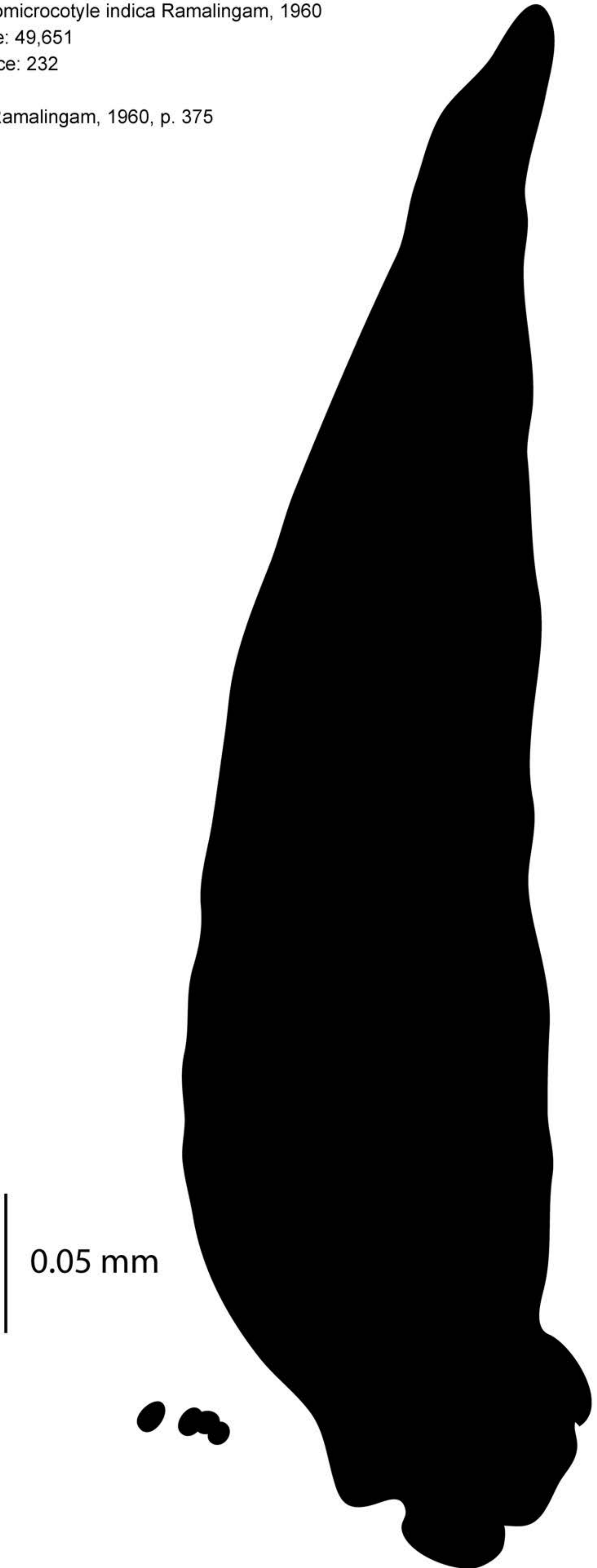

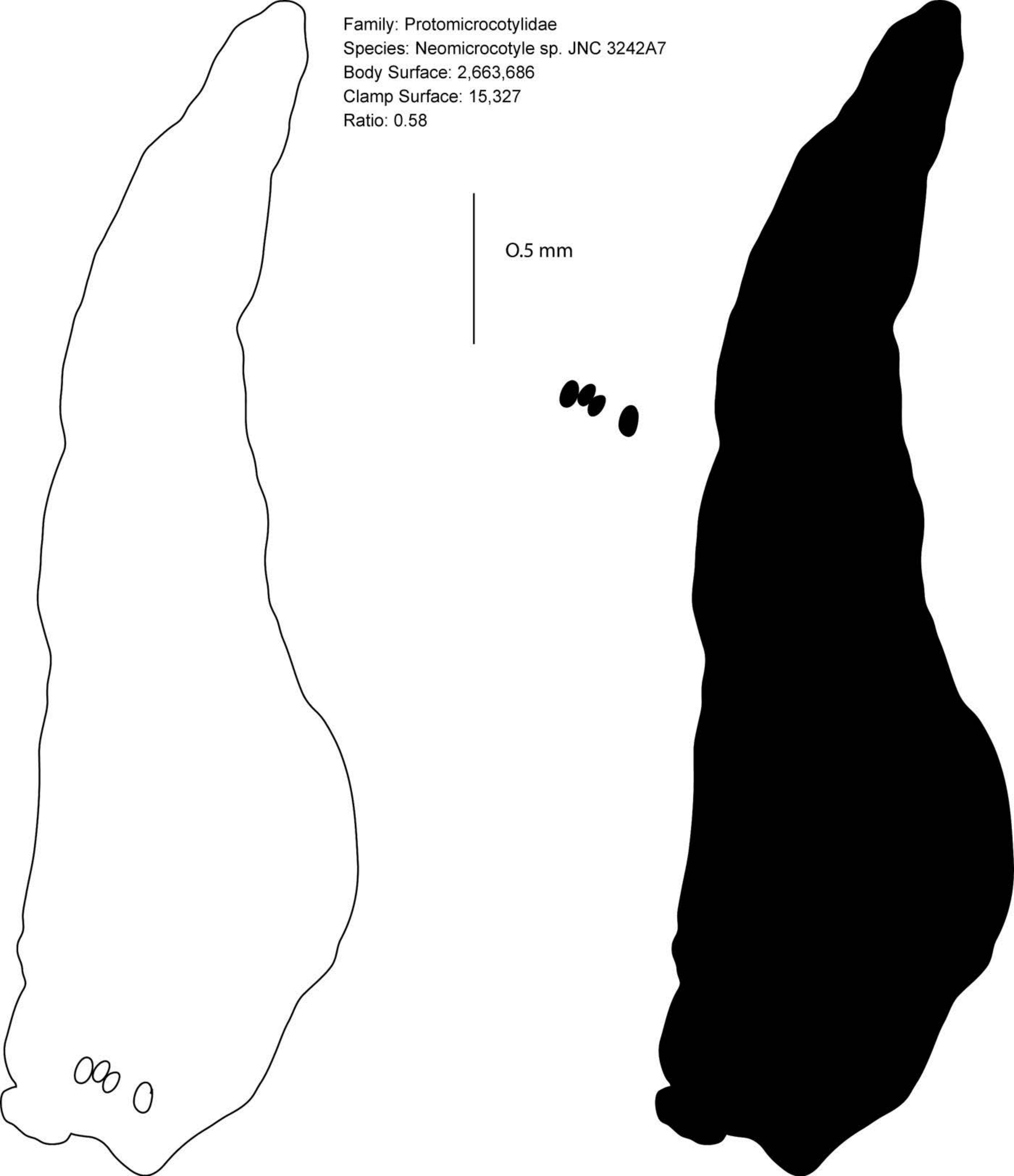
Family: Protomicrocotylidae
Species: Neomicrocotyle sp. JNC 3242A7
Body Surface: 2,663,686
Clamp Surface: 15,327
Ratio: 0.58
0.5 mm

Family: Protomicrocotylidae
Species: Neomicrocotyle unnithani Yamaguti, 1968
Body Surface: 2,019,641
Clamp Surface: 13,440
Ratio: 0.67
Reference: Unnithan, 1962, p. 344

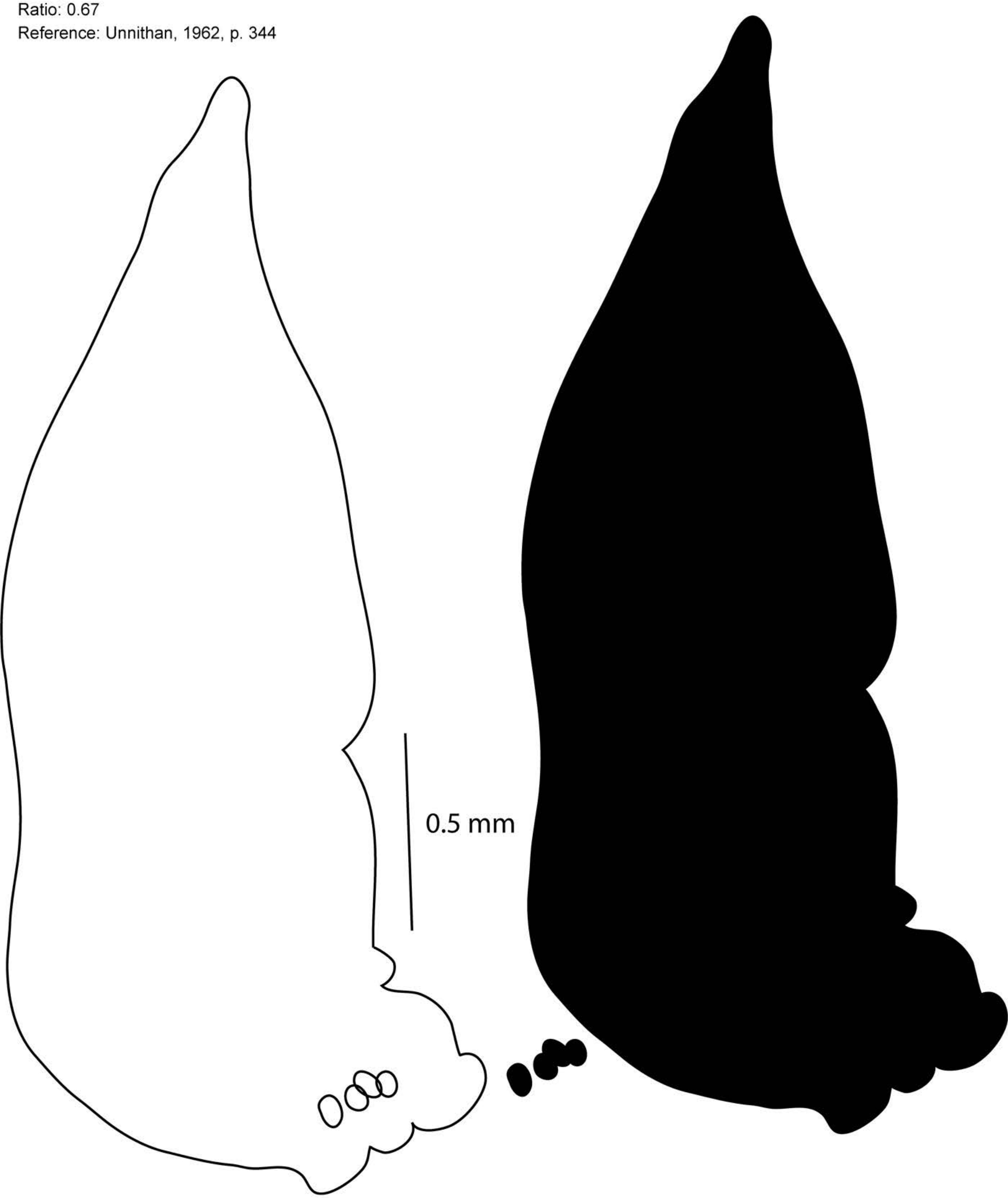

Family: Protomicrocotylidae
Species: Protomicrocotyle celebesensis Yamaguti, 1953
Body Surface: 1,791,383
Clamp Surface: 11,869
Ratio: 0.66
Reference: Yamaguti, 1953, p. 56

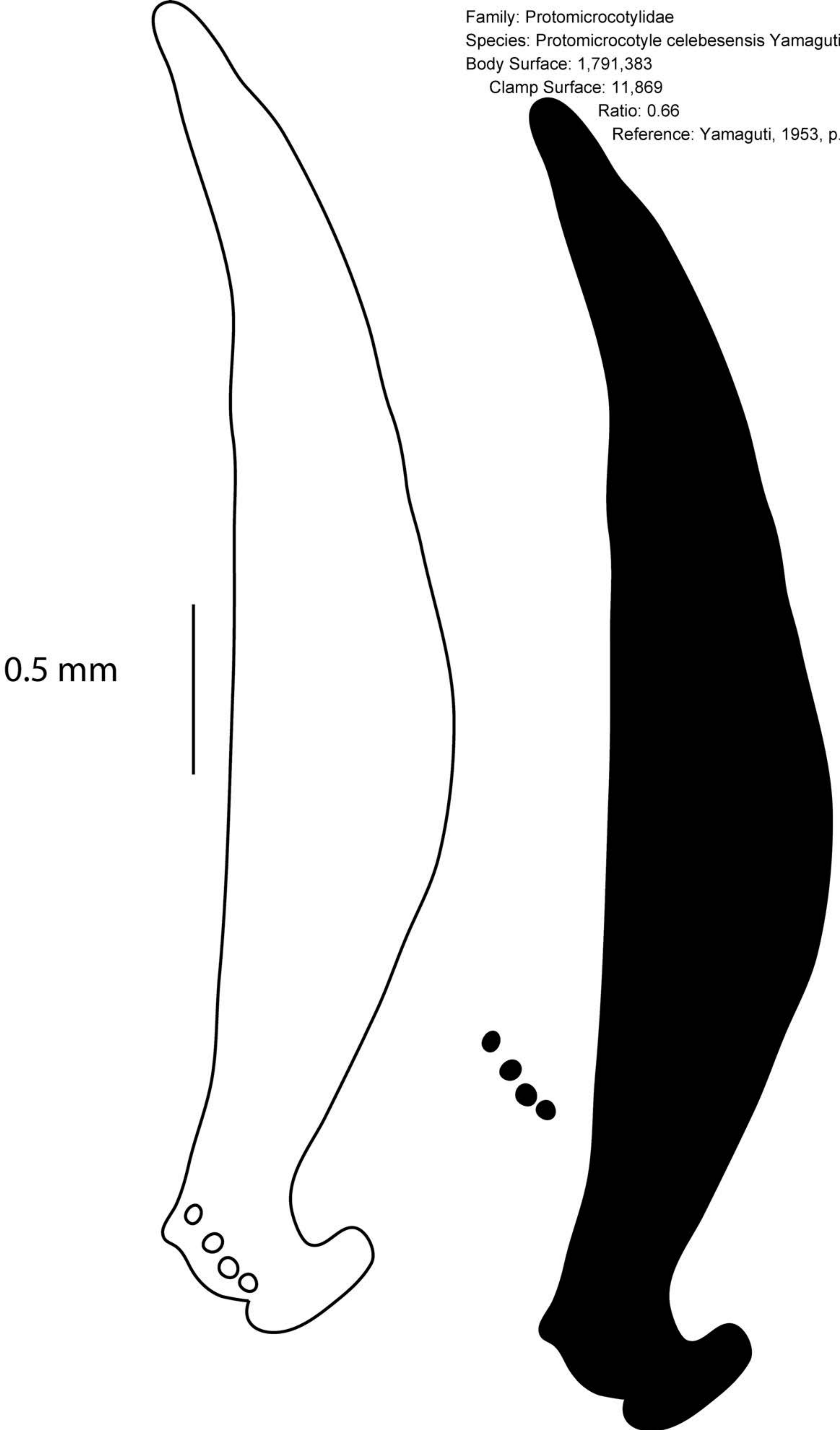

Family: Protomicrocotylidae
Species: Protomicrocotyle ivoriensis Wahl, 1972
Body Surface: 2,939,959 ; Clamp Surface: 7,682
Ratio: 0.26
Reference: Wahl, 1972, p. 324

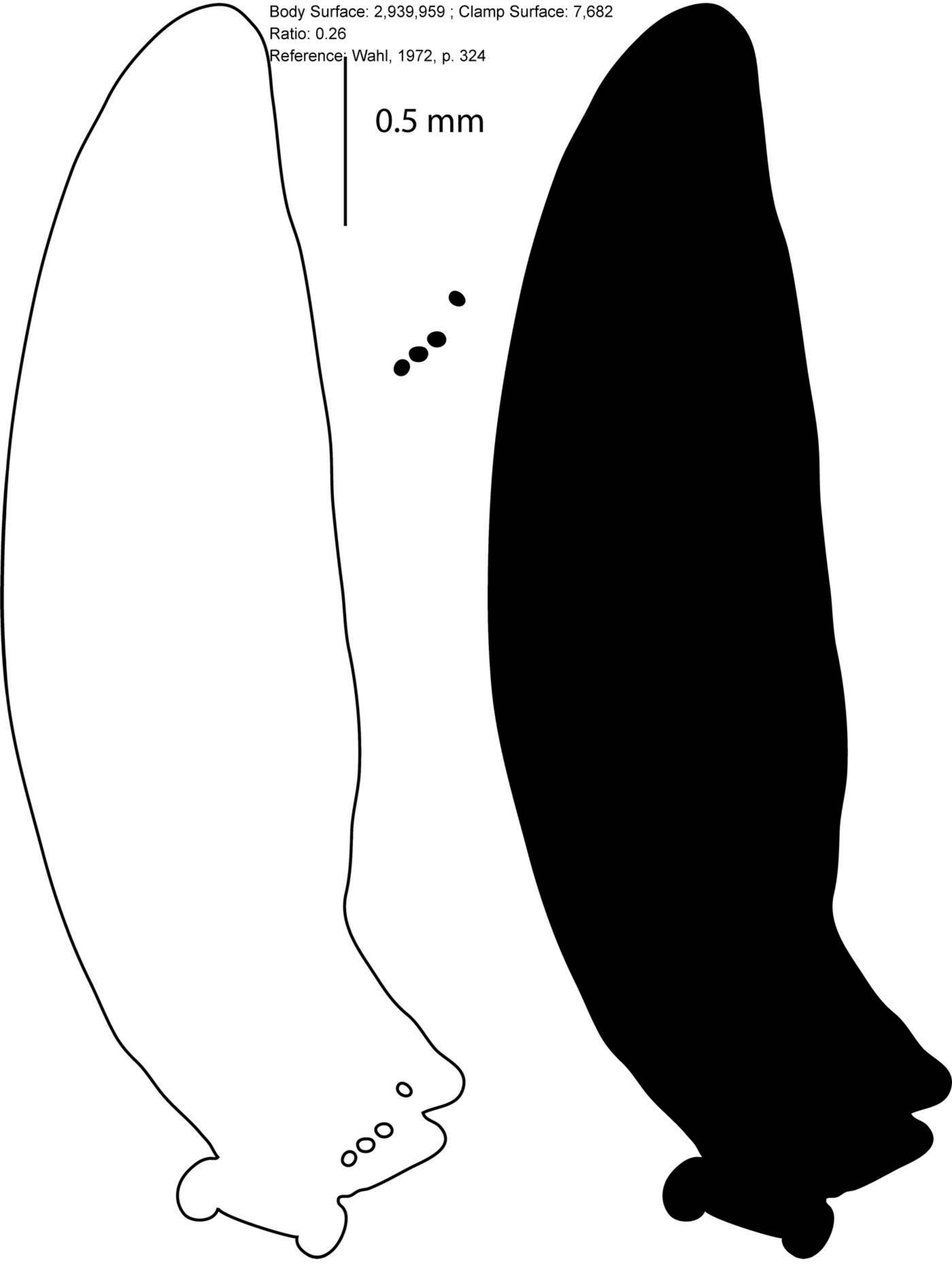

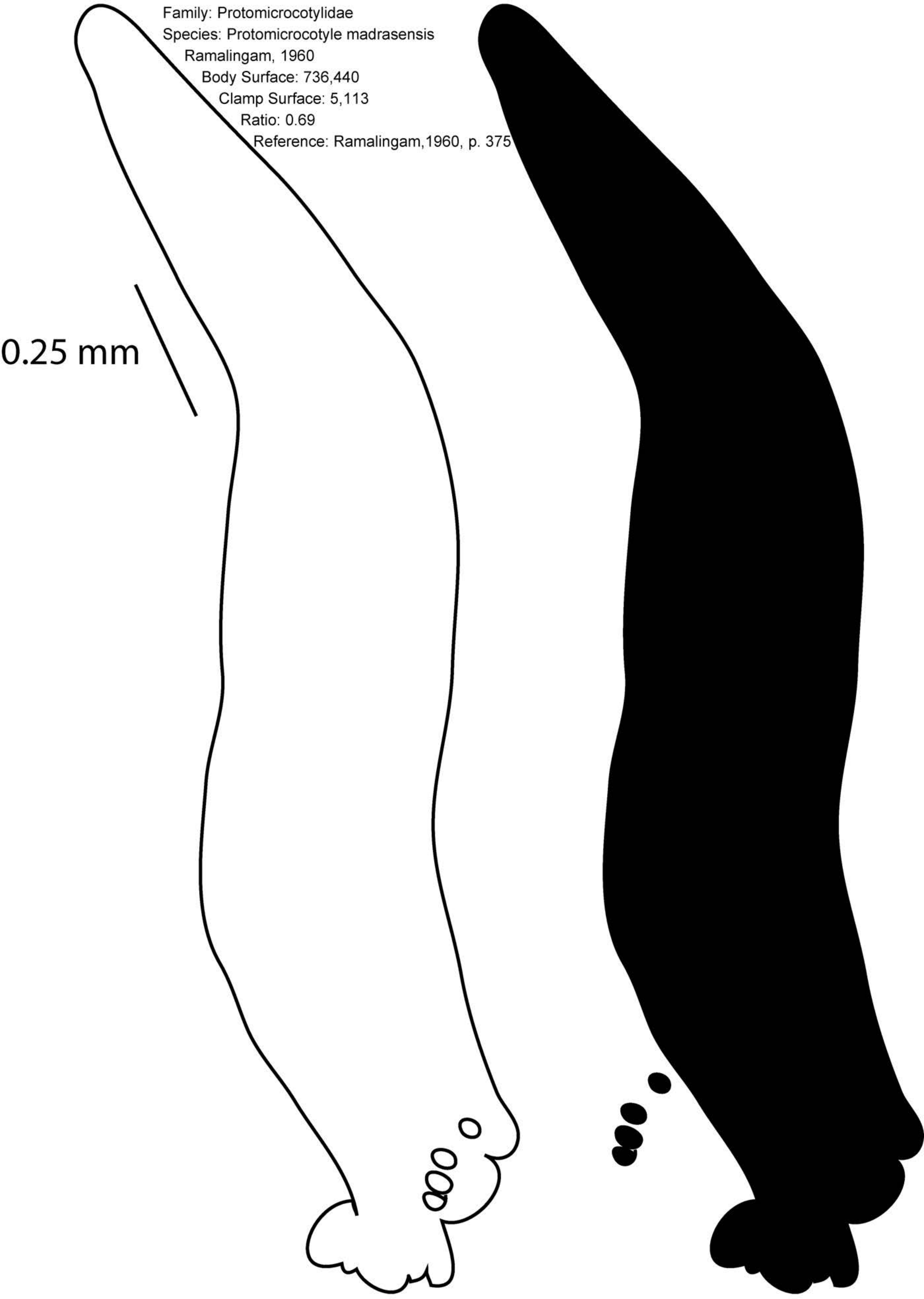
Family: Protomicrocotylidae
Species: Protomicrocotyle madrasensis
Ramalingam, 1960
Body Surface: 736,440
Clamp Surface: 5,113
Ratio: 0.69
Reference: Ramalingam,1960, p. 375
0.25 mm

Family: Protomicrocotylidae
Species: Protomicrocotyle mannarensis
Ramalingam, 1960
Body Surface: 1,934,754
Clamp Surface: 10,205
Ratio: 0.53
Reference: Ramalingam, 1960, p. 377

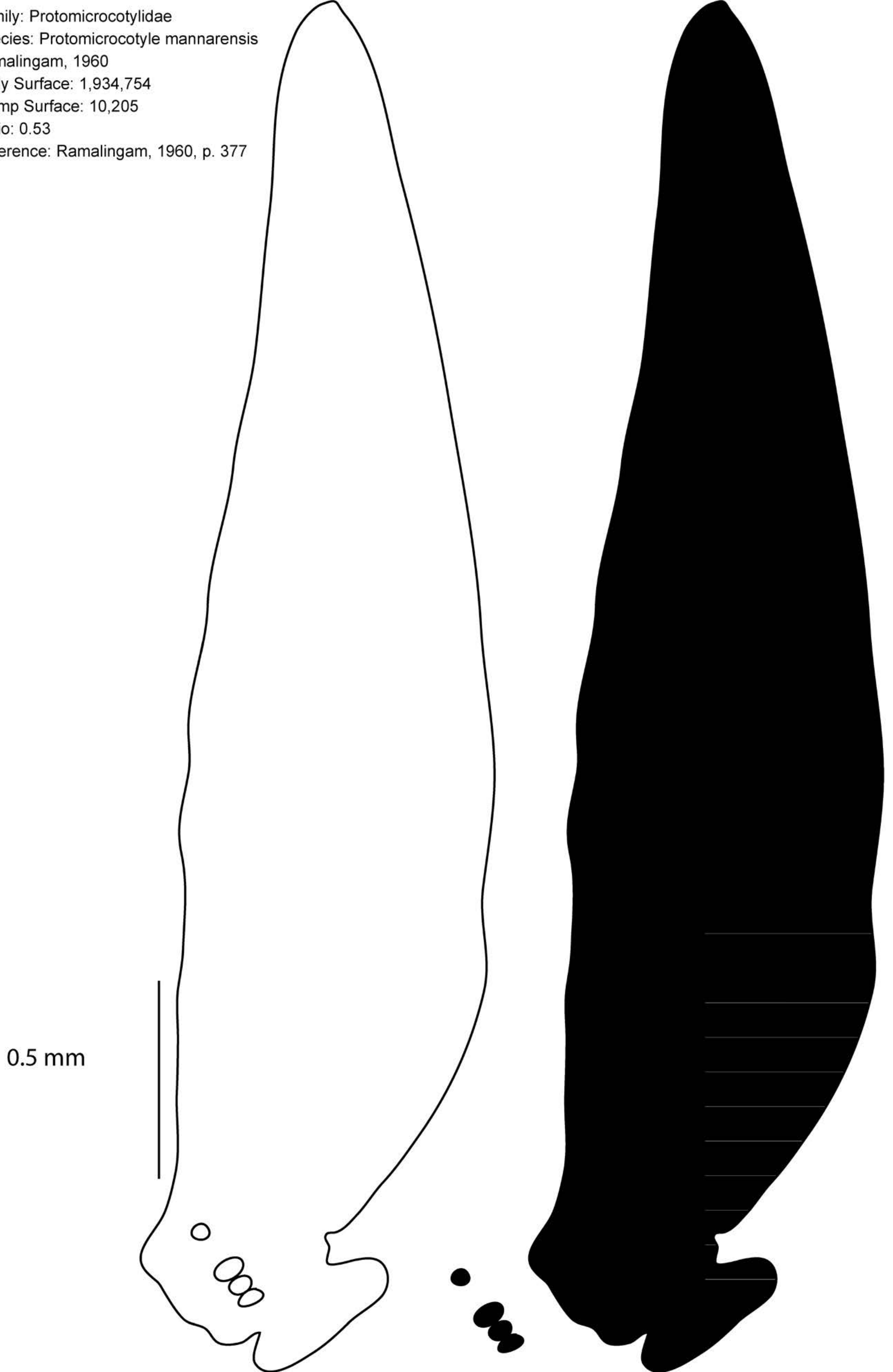

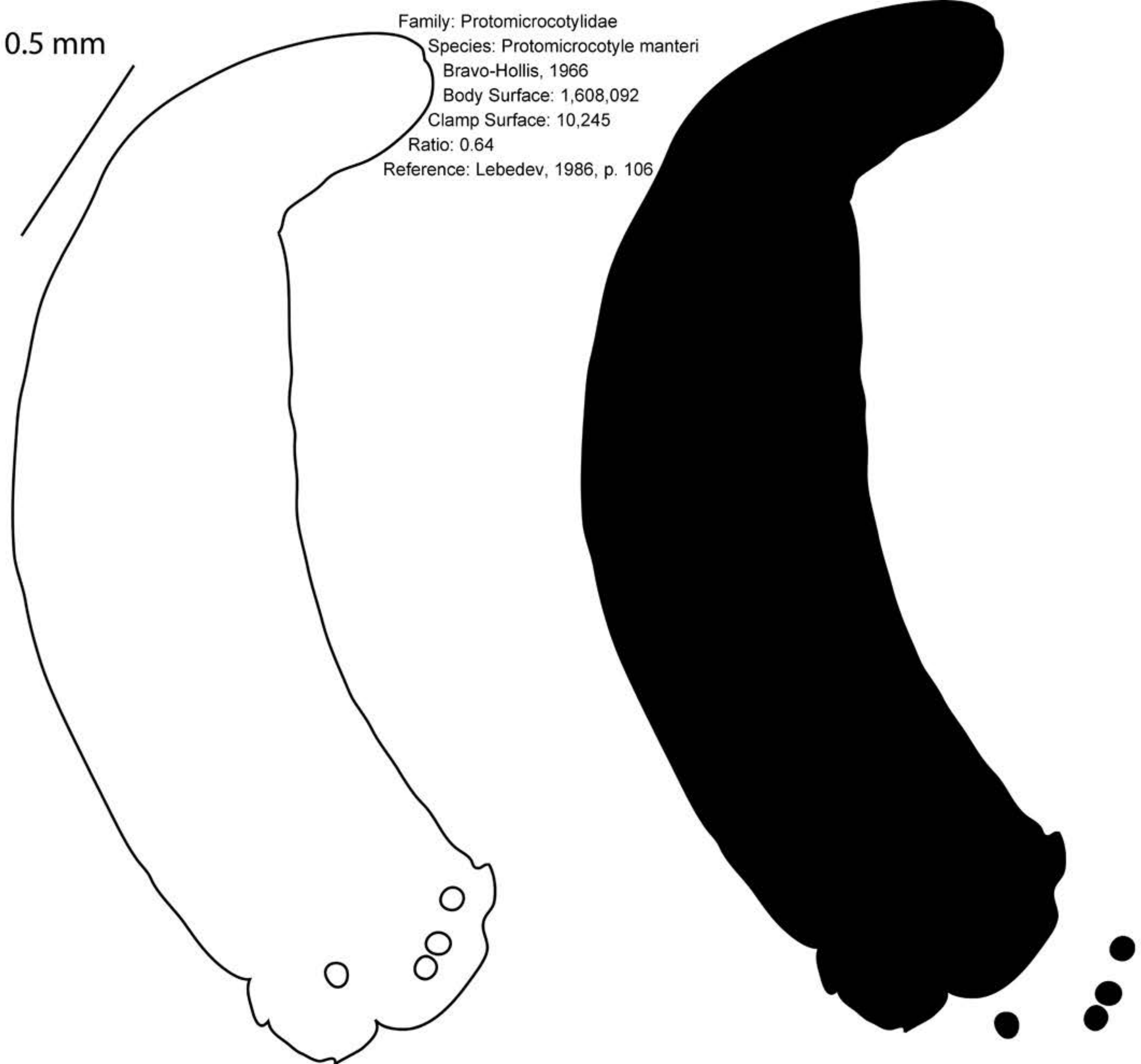
0.5 mm
Family: Protomicrocotylidae
Species: Protomicrocotyle manteri
Bravo-Hollis, 1966
Body Surface: 1,608,092
Clamp Surface: 10,245
Ratio: 0.64
Reference: Lebedev, 1986, p. 106

Family: Protomicrocotylidae
Species: Protomicrocotyle minuta Ramalingam, 1960
Body Surface: 334,808
Clamp Surface: 6,589
Ratio: 1.97
Reference: Ramalingam, 1960, p. 377

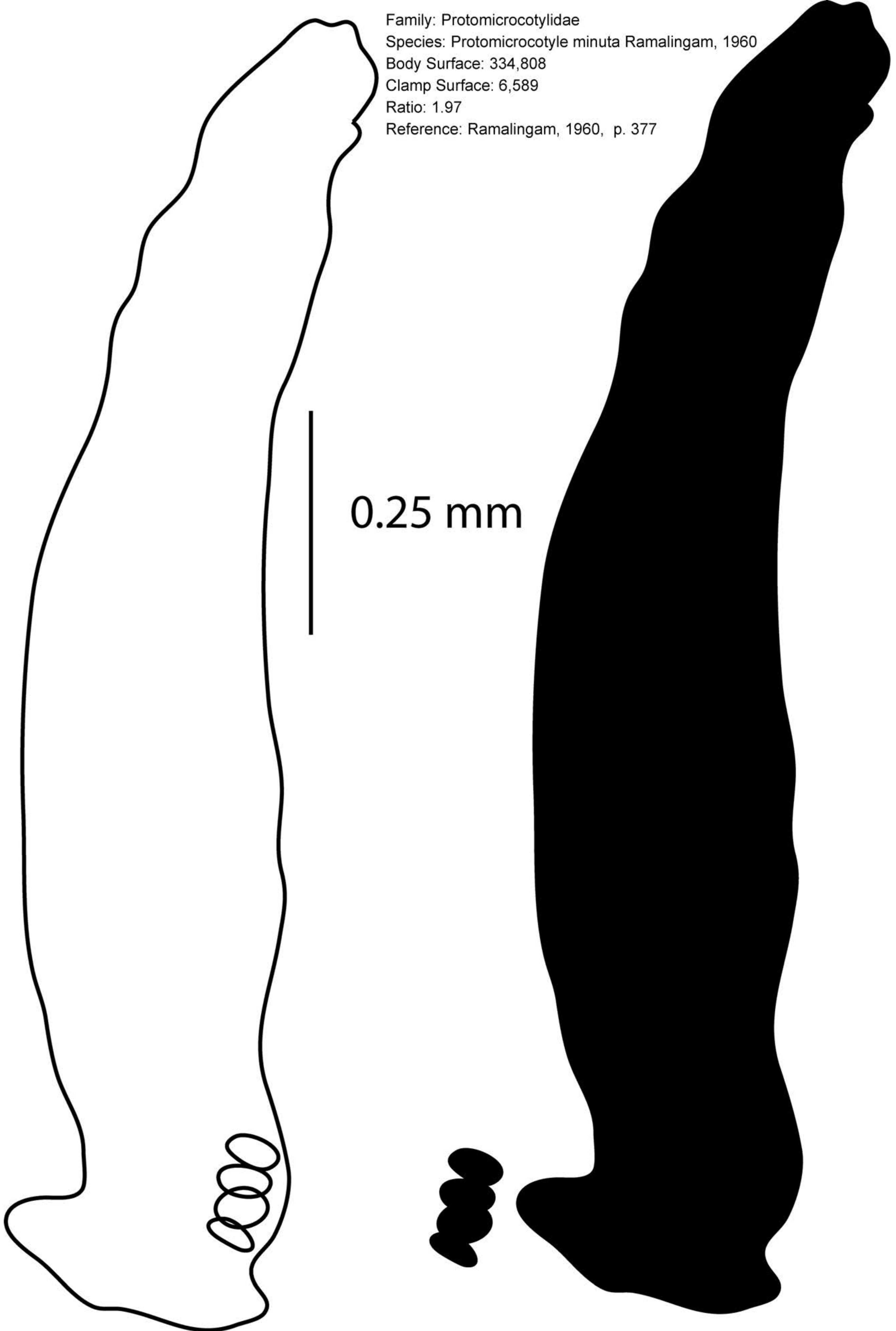

Family: Protomicrocotylidae
Species: Protomicrocotyle mirabilis (MacCallum, 1918) Johnston & Tiegs, 1922
Body Surface: 231,559
Clamp Surface: 7,854
Ratio: 3.39
Reference: Wahl, 1972, p. 321

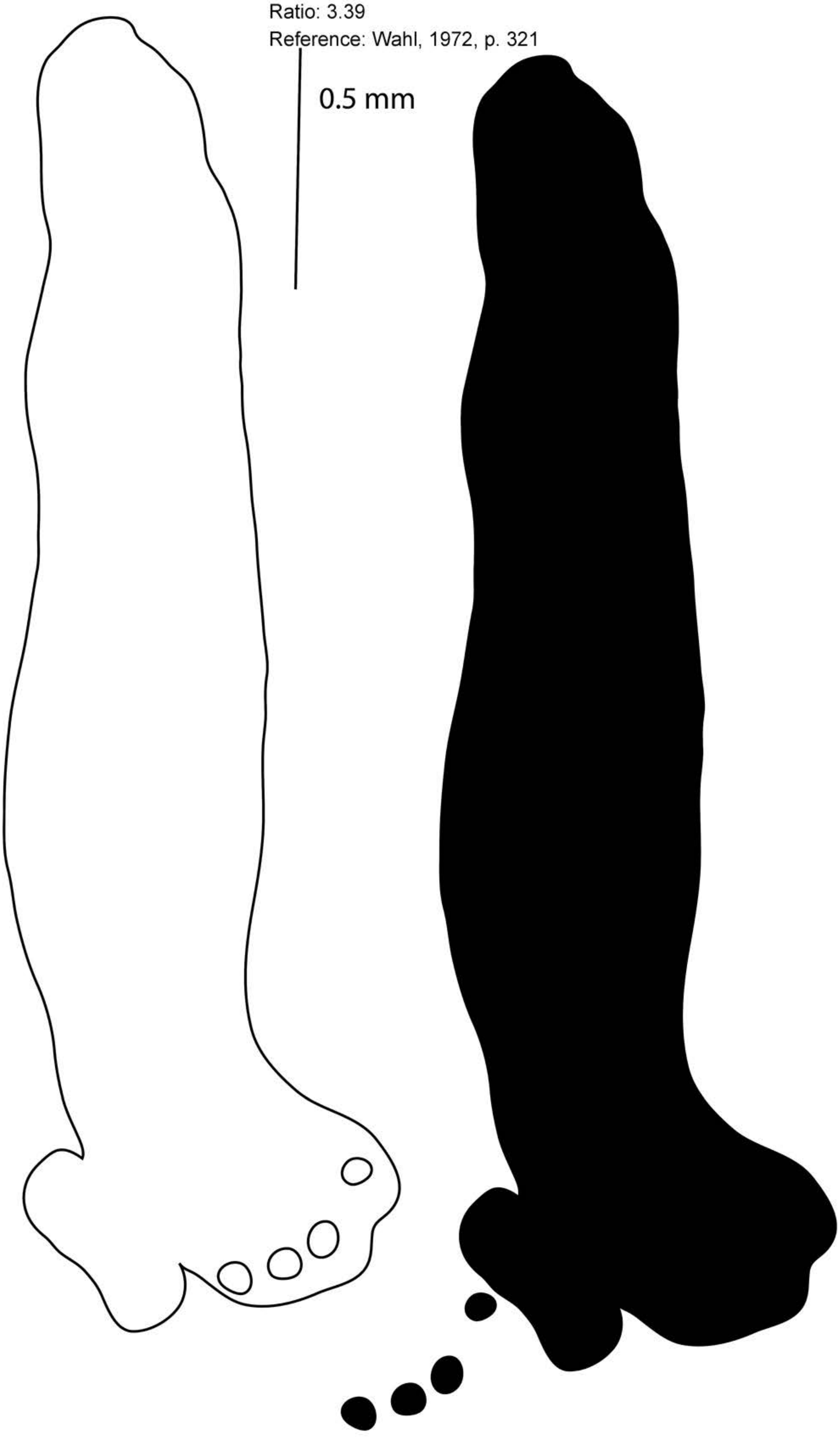

Family: Protomicrocotylidae
Species: Protomicrocotyle nayaritensis Bravo-Hollis, 1979
Body Surface: 7,317,320
Clamp Surface: 13,238
Ratio: 0.18
Reference: Bravo Hollis, 1979, p. 190

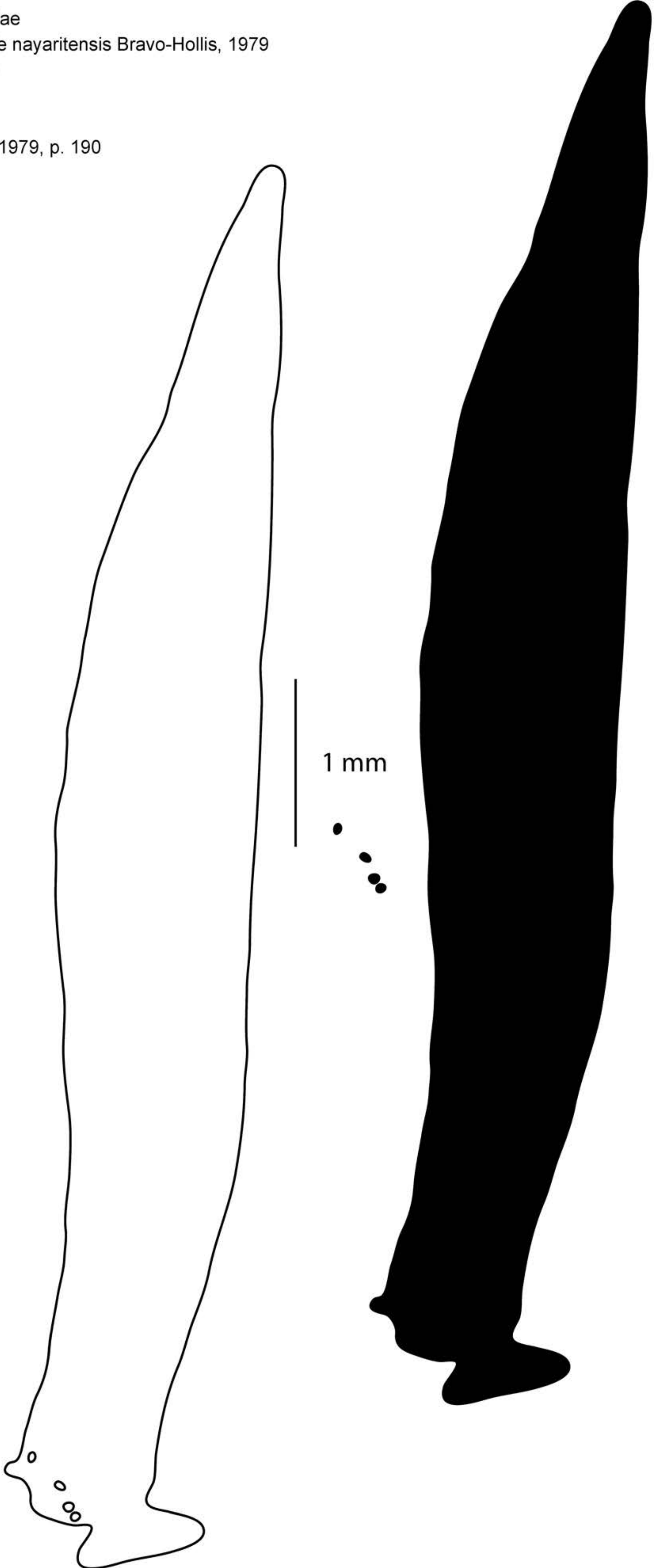

Family: Protomicrocotylidae
Species: Vallisiopsis contorta Subhapradha, 1951
Body Surface: 29,848
Clamp Surface: 281
Ratio: 0.94
Reference: Lebedev, 1986, p. 17

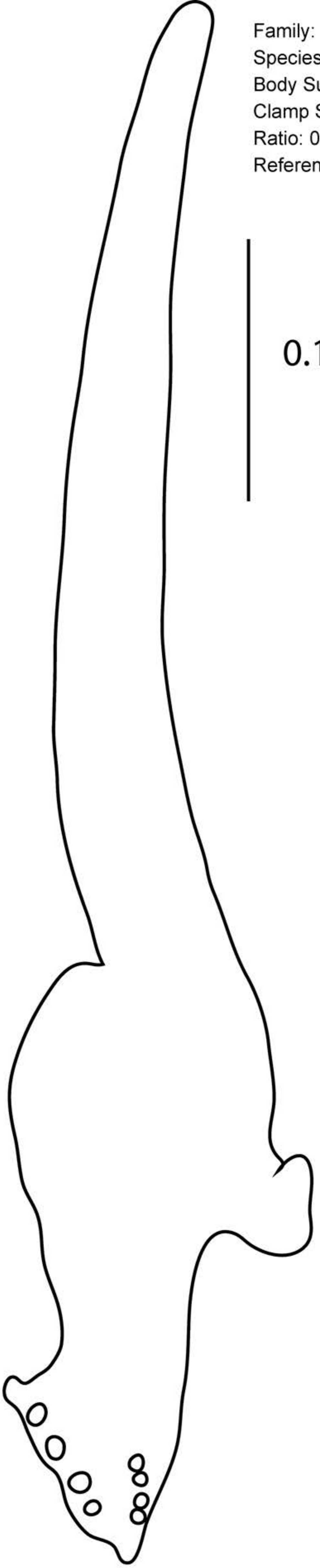

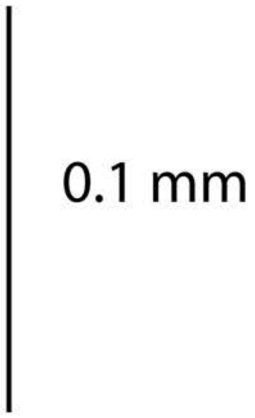

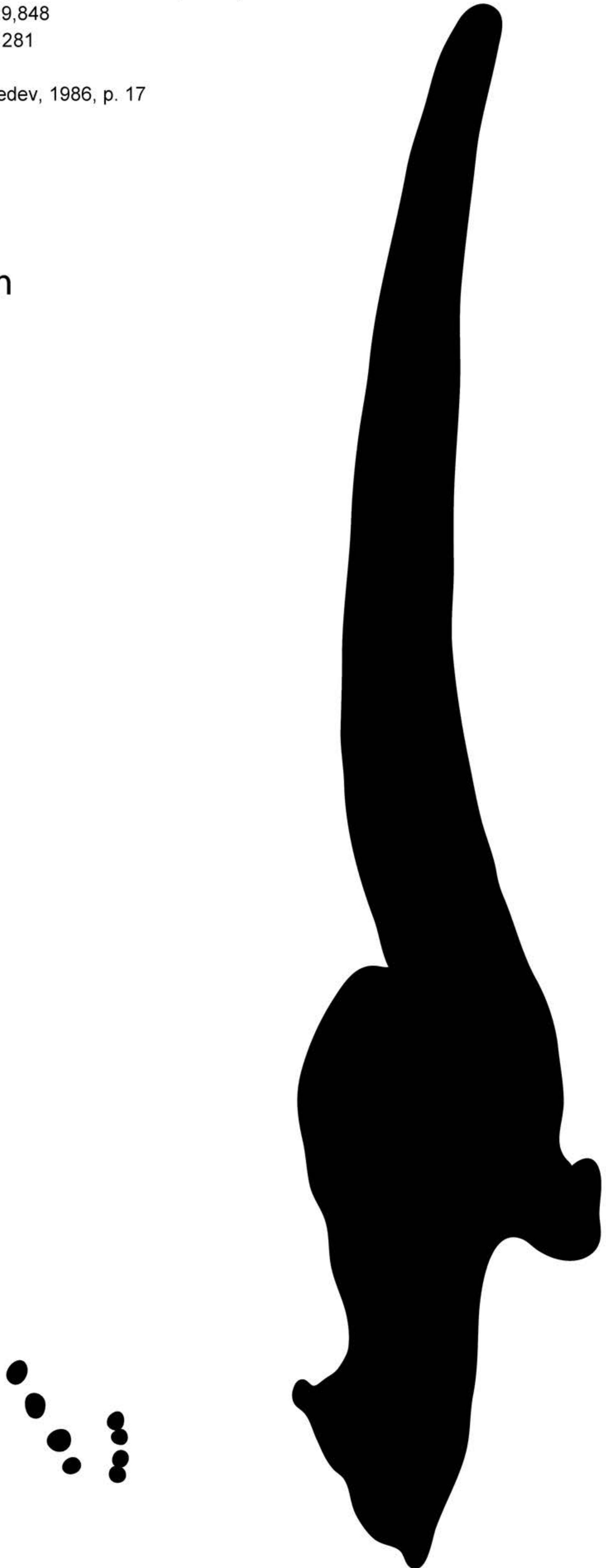

Family: Protomicrocotylidae
Species: Vallisiopsis sphyraenae Yamaguti, 1968
Body Surface: 6,746,717
Clamp Surface: 48,813
Ratio: 0.72
Reference: Yamaguti, 1968, p. 251

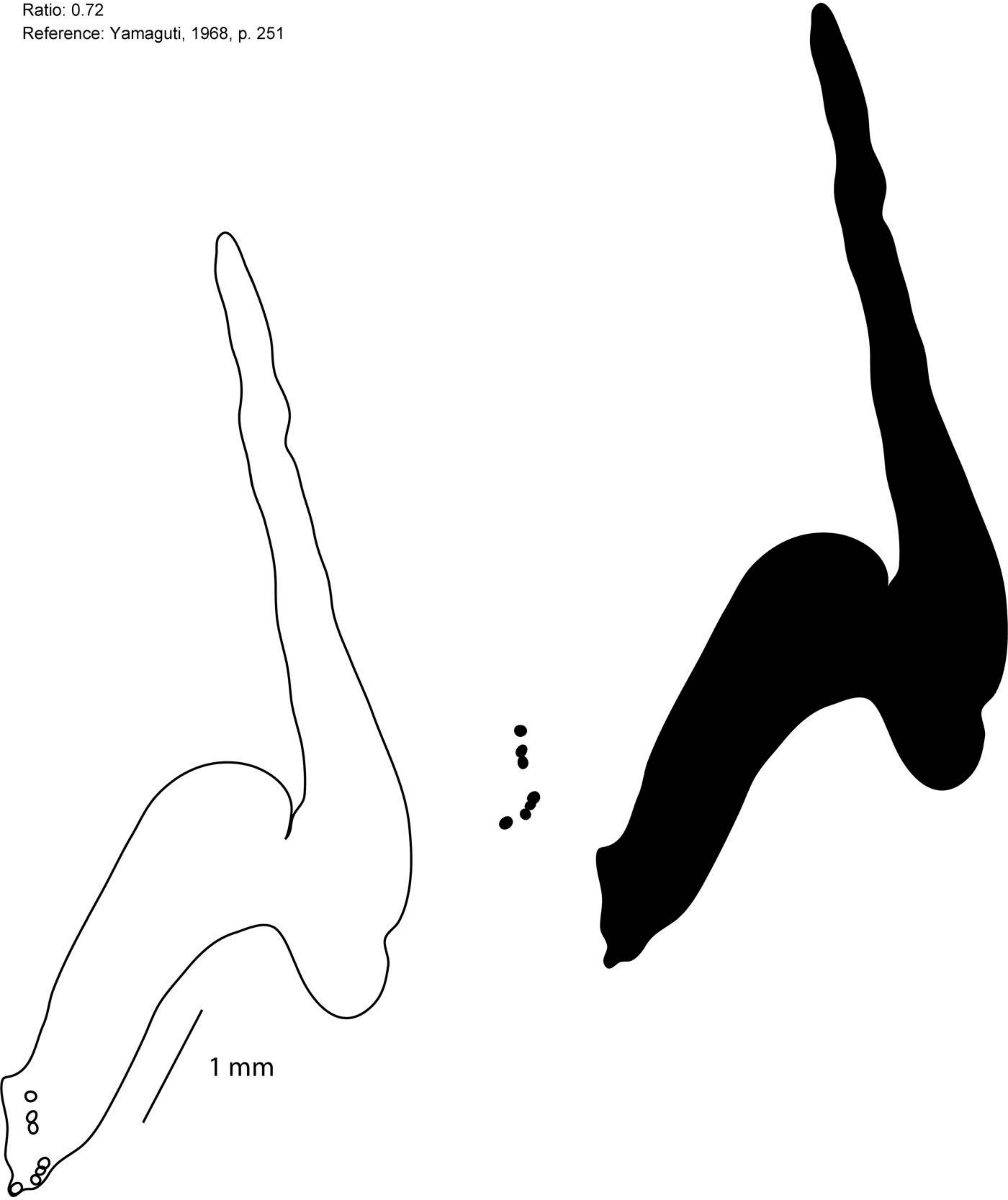

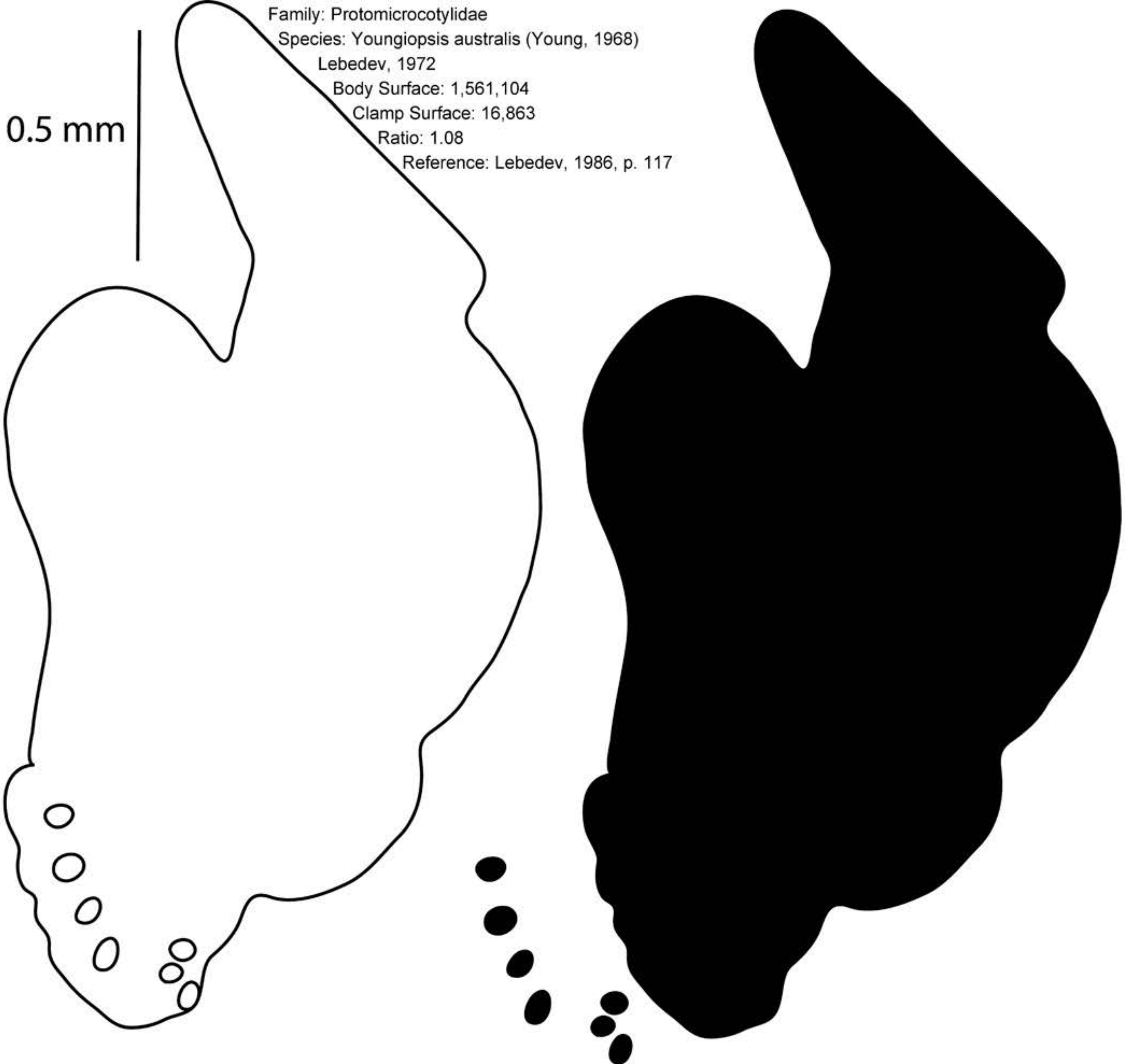
Family: Protomicrocotylidae
Species: Youngiopsis australis (Young, 1968)
Lebedev, 1972
Body Surface: 1,561,104
Clamp Surface: 16,863
Ratio: 1.08
Reference: Lebedev, 1986, p. 117
0.5 mm

Family: Pseudodiclidophoridae

Species: Allopseudodiclidophora opelu Yamaguti, 1965

Body Surface: 5,244,324 ; Clamp Surface: 20,471

Ratio: 0.39

Reference: Yamaguti, 1965, p. 73

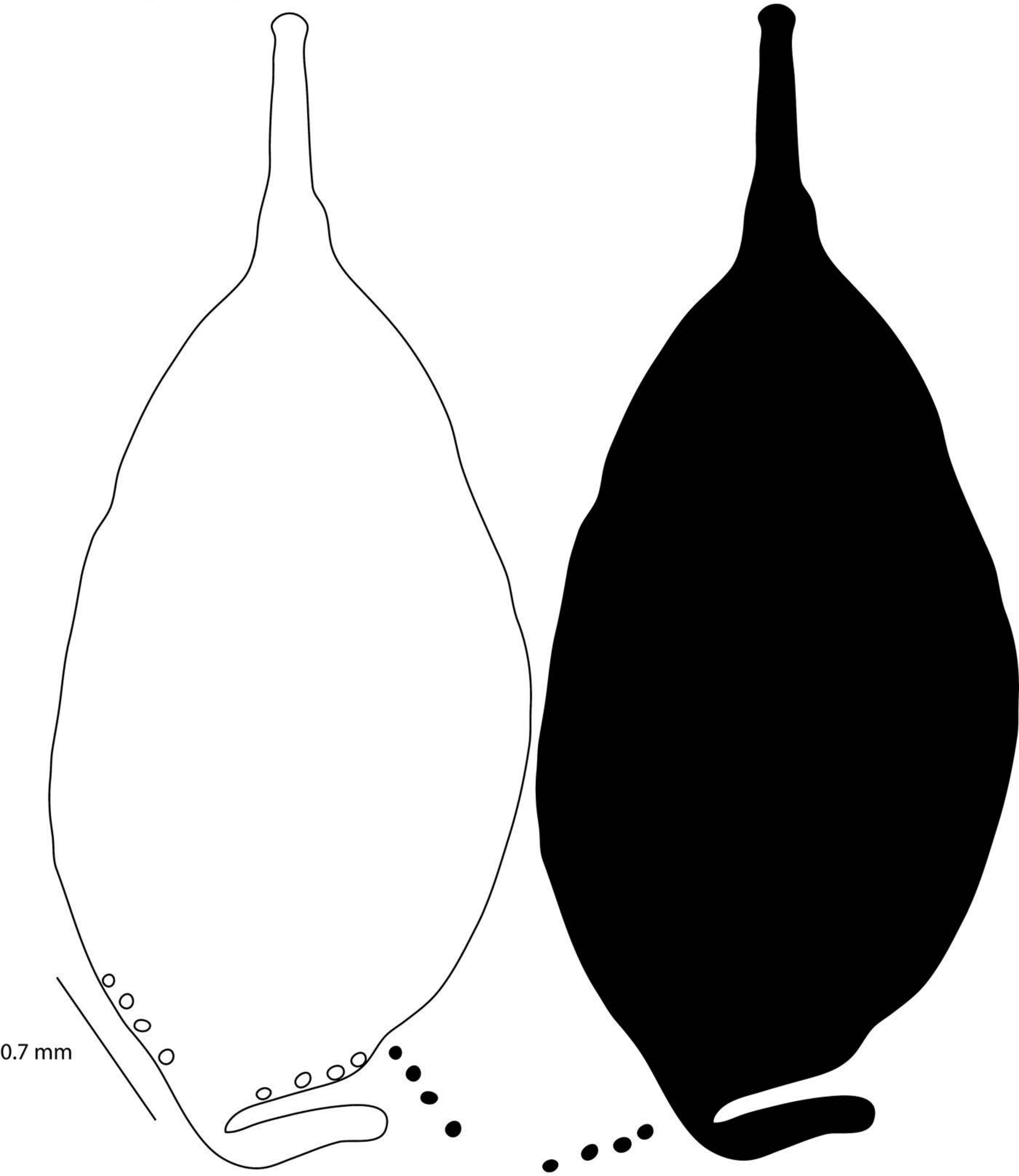

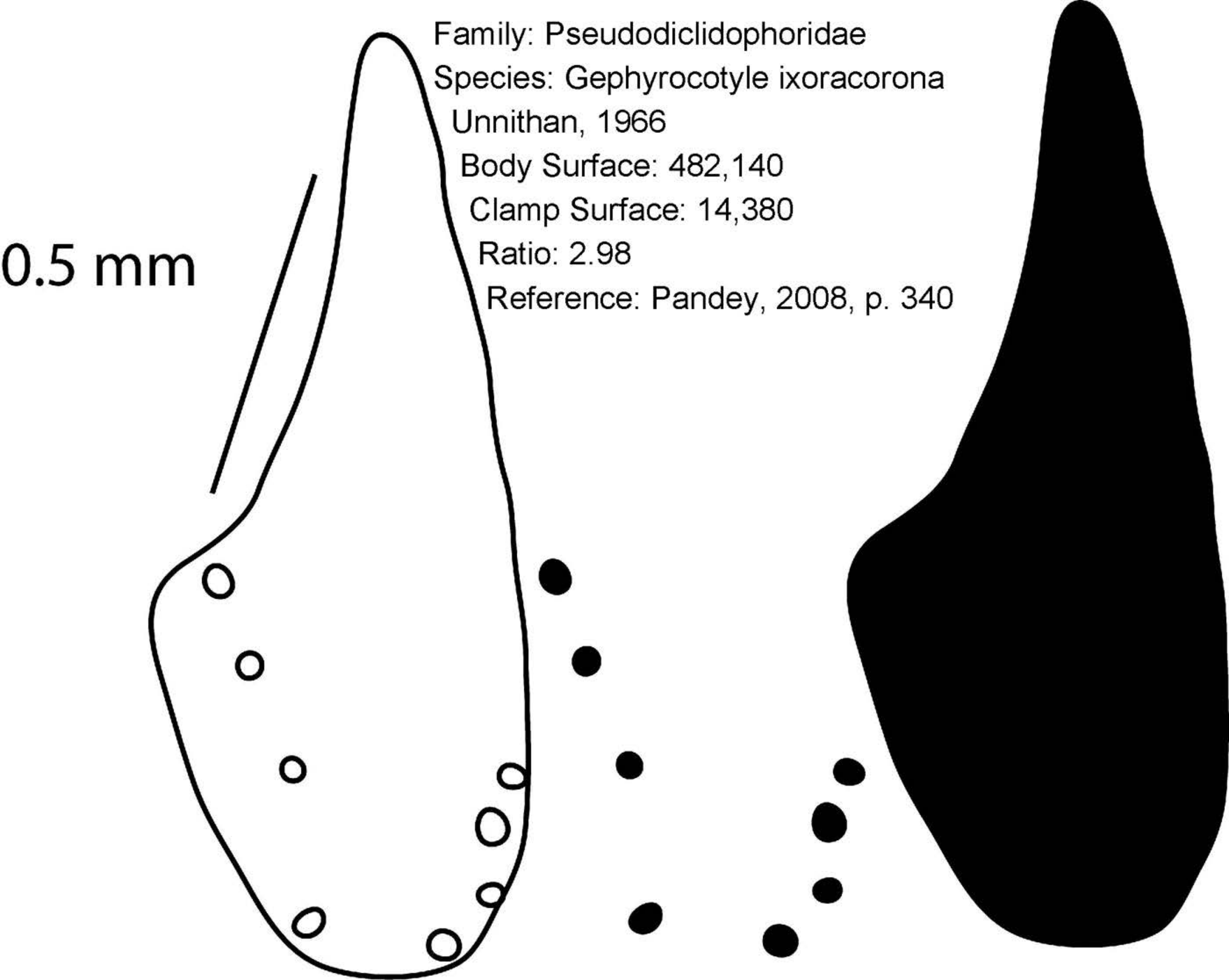

Family: Pseudodiclidophoridae
Species: Gephyrocotyle ixoracorona
Unnithan, 1966
Body Surface: 482,140
Clamp Surface: 14,380
Ratio: 2.98
Reference: Pandey, 2008, p. 340
0.5 mm

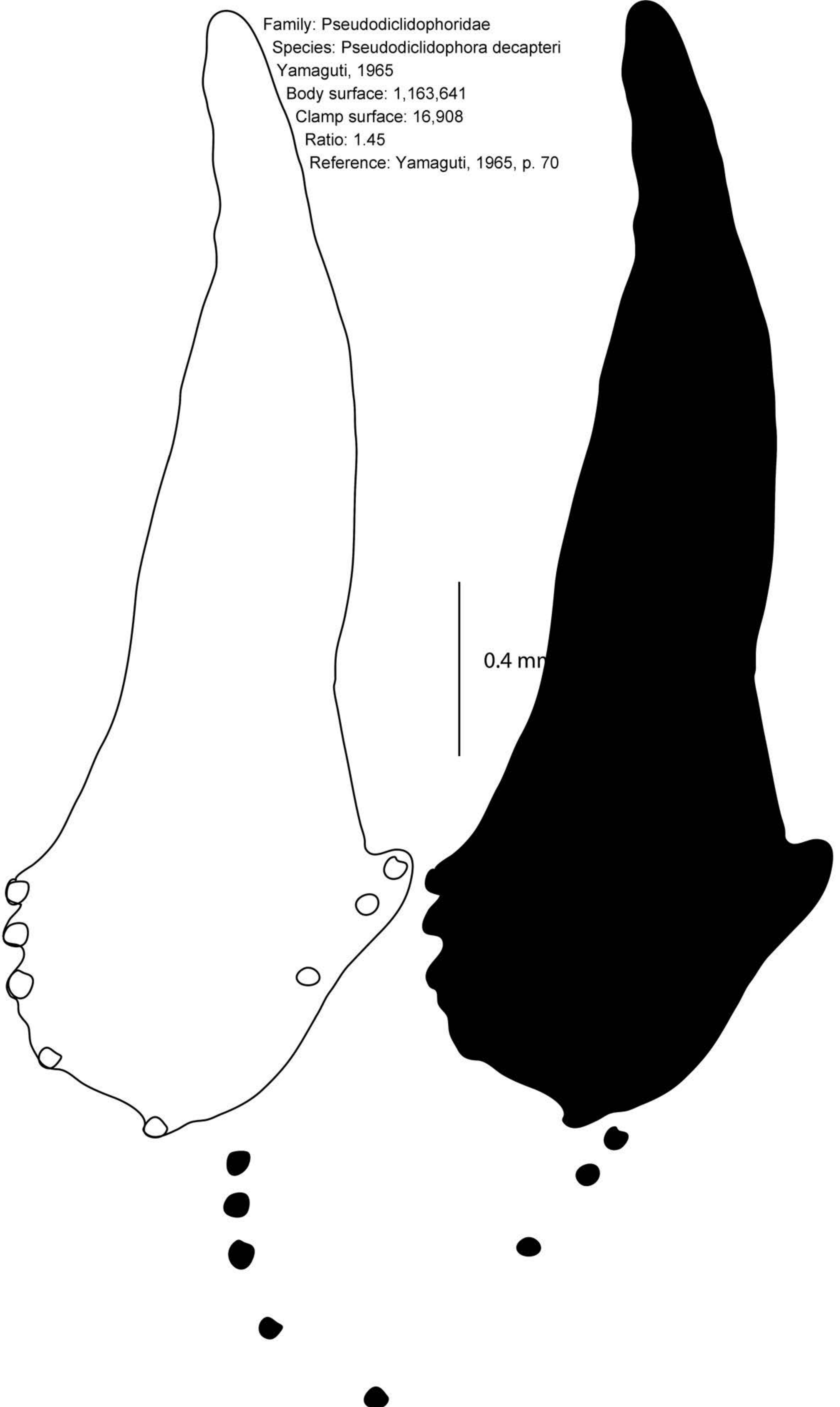
Family: Pseudodiclidophoridae
Species: Pseudodiclidophora decapteri
Yamaguti, 1965
Body surface: 1,163,641
Clamp surface: 16,908
Ratio: 1.45
Reference: Yamaguti, 1965, p. 70
0.4 mm

Family: Pseudodiclidophoridae
Species: Quilonella ventrosa Lebedev & Parukhin, 1970
Body Surface: 954,583; Clamp Surface: 13,250
Ratio: 1.39
Reference: Lebedev, 1986, p. 81

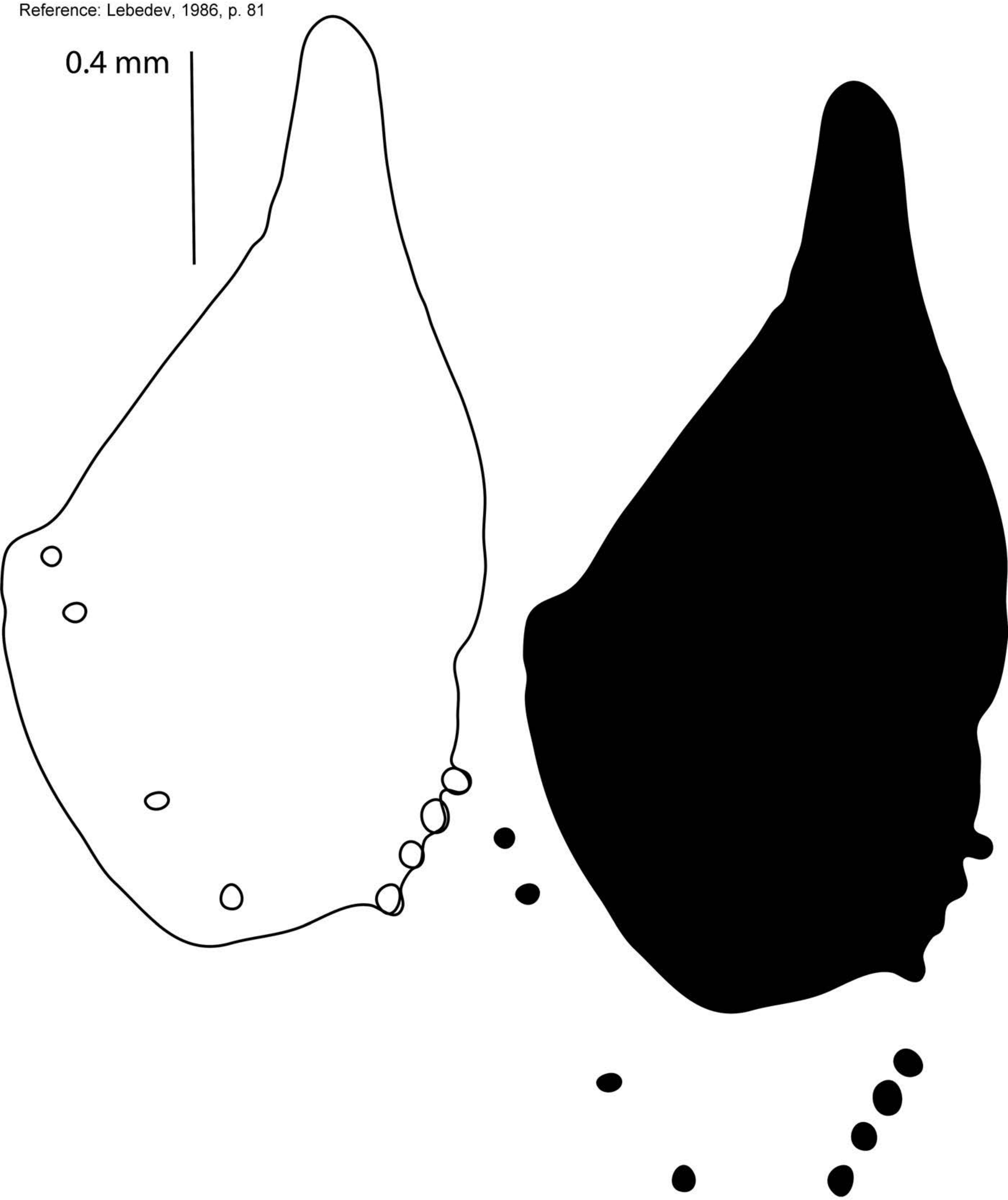

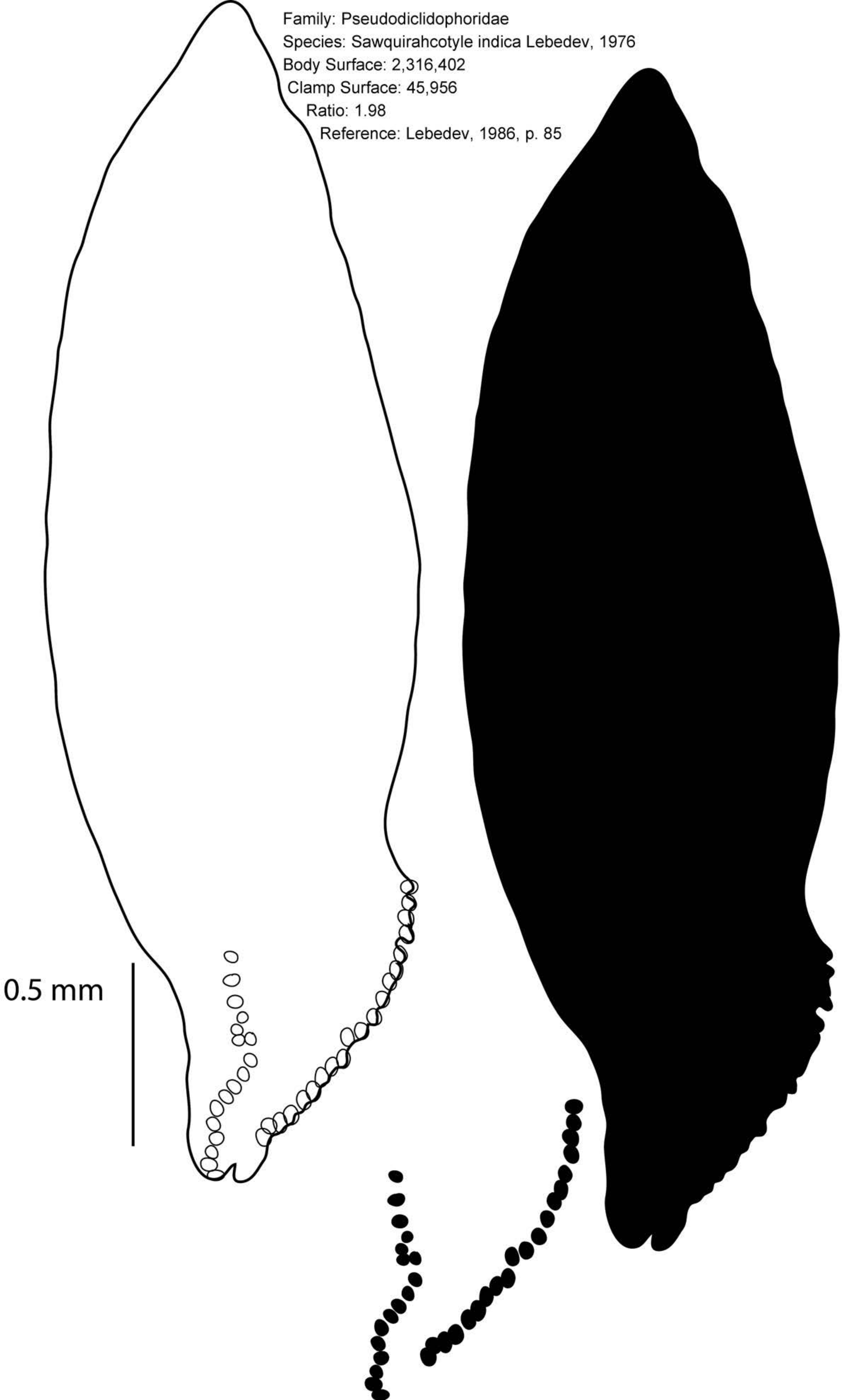
Family: Pseudodiclidophoridae
Species: Sawquirahcotyle indica Lebedev, 1976
Body Surface: 2,316,402
Clamp Surface: 45,956
Ratio: 1.98
Reference: Lebedev, 1986, p. 85
0.5 mm

Family: Pseudodiclidophoridae
Species: Winkenthughesia australis Robinson, 1961
Body Surface: 8,558,914
Clamp Surface: 244,842
Ratio: 2.86
Reference: Robinson, 1961, p. 261

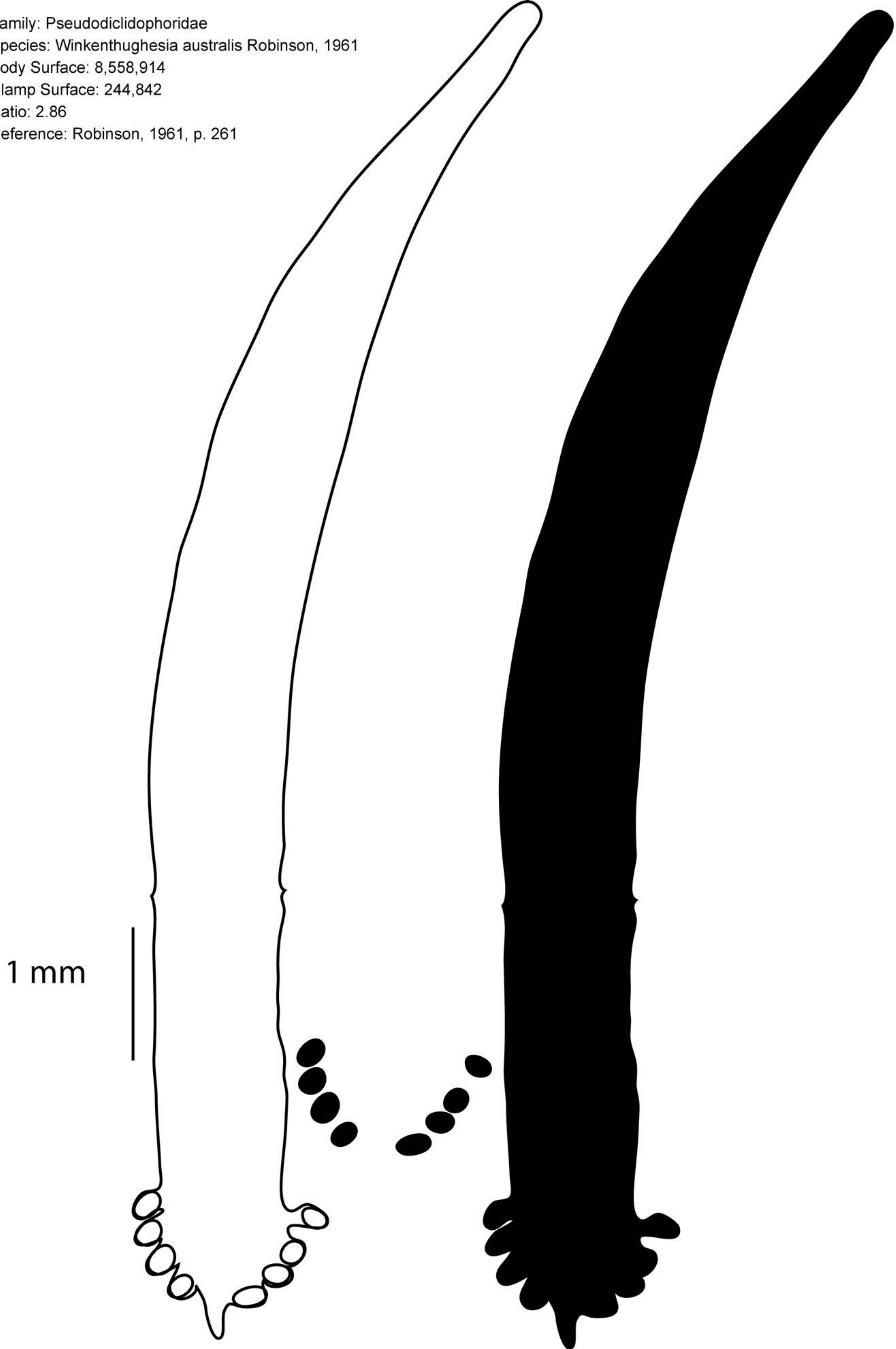

Supplement: File S1 — PDF of all figures and measurements of clamp and body surfaces. Total number of figures: 120. (PDF) [file pone.0079155.s001.pdf]
